# Computational discovery of binding mode of anti-TRBC1 antibody and predicted key amino acids of TRBC1

**DOI:** 10.1038/s41598-022-05742-6

**Published:** 2022-02-02

**Authors:** Jirakrit Saetang, Surasak Sangkhathat, Nawaphat Jangphattananont, Wannakorn Khopanlert, Jakrawadee Julamanee, Varomyalin Tipmanee

**Affiliations:** 1 Division of Surgery, Faculty of Medicine, Prince of Songkla University, Songkhla, 90110 Thailand; 2 International Center of Excellence in Seafood Science and Innovation, Faculty of Agro-Industry, Prince of Songkla University, Songkhla, 90110 Thailand; 3 EZ-Mol-Design Laboratory, Faculty of Medicine, Prince of Songkla University, Songkhla, 90110 Thailand; 4 Stem Cell Laboratory, Hematology Unit, Division of Internal Medicine, Faculty of Medicine, Prince of Songkla University, Songkhla, 90110 Thailand; 5 Division of Biomedical Sciences and Biomedical Engineering, Faculty of Medicine, Prince of Songkla University, Songkhla, 90110 Thailand

**Keywords:** Cancer, Computational biology and bioinformatics, Structural biology

## Abstract

Peripheral T-cell lymphoma (PTCL) is a type of non-Hodgkin lymphoma that progresses aggressively with poor survival rate. CAR T cell targeting T-cell receptor β-chain constant domains 1 (TRBC1) of malignant T cells has been developed recently by using JOVI.1 monoclonal antibody as a template. However, the mode of JOVI.1 binding is still unknown. This study aimed to investigate the molecular interaction between JOVI.1 antibody and TRBC1 by using computational methods and molecular docking. Therefore, the TRBC protein crystal structures (TRBC1 and TRBC2) as well as the sequences of JOVI.1 CDR were chosen as the starting materials. TRBC1 and TRBC2 epitopes were predicted, and molecular dynamic (MD) simulation was used to visualize the protein dynamic behavior. The structure of JOVI.1 antibody was also generated before the binding mode was predicted using molecular docking with an antibody mode. Epitope prediction suggested that the N3K4 region of TRBC1 may be a key to distinguish TRBC1 from TCBC2. MD simulation showed the major different surface conformation in this area between two TRBCs. The JOVI.1-TRBC1 structures with three binding modes demonstrated JOVI.1 interacted TRBC1 at N3K4 residues, with the predicted dissociation constant (K_d_) ranging from 1.5 × 10^8^ to 1.1 × 10^10^ M. The analysis demonstrated JOVI.1 needed D1 residues of TRBC1 for the interaction formation to N3K4 in all binding modes. In conclusion, we proposed the three binding modes of the JOVI.1 antibody to TRBC1 with the new key residue (D1) necessary for N3K4 interaction. This data was useful for JOVI.1 redesign to improve the PTCL-targeting CAR T cell.

## Introduction

Peripheral T-cell lymphoma (PTCL) is a highly aggressive hematologic malignancy with reported of less than 32% five-year survival rate^1^. Family background of hematologic malignancies, some skin conditions, celiac disease, smoking, and certain occupations are statistically often associated with PTCL development^2^. The combination chemotherapy regimens; for example, CHOP (cyclophosphamide, doxorubicin, vincristine, and prednisone) and CHOEP (etoposide, vincristine, doxorubicin, cyclophosphamide, and prednisone) are typically used as initial treatment for PTCL patients^3^. However, most of the patients relapse after treatment with standard chemotherapy, resulting in a poor survival outcome^4^. Therefore, the novel treatment modalities are needed to improve treatment responses and long-term survival outcomes.

Adoptive T-cell therapy has been investigated and currently applied to clinical practice, especially chimeric antigen receptor (CAR) T-cell therapy. Recently, the genetically modified-autologous CAR-T cells using single chain variable fragment (scFv) derived from monoclonal antibodies have been developed to specifically engage with target antigen on the tumor cell surface^5^. T-cell receptor β-chain constant domains 1 and 2 (TRBC1 and TRBC2) serve as one of the specific antigens recognizing markers for PTCL. Normal T-cell consists of both TRBC1 and TRBC2; however, the malignant T-cell contains only one either TRBC1 or TRBC2^6^. This feature will facilitate CAR T-cells to categorize malignant T-cells from normal T-cells. Recently, JOVI.1 clone of anti-TRBC1 monoclonal antibody has been studied and confirmed the specificity for TRBC1 recognition^6^. Although TRBC1 and TRBC2 shared somewhat similar protein sequences as well as three dimensional structures^7,8^, the previous report proposed that the alteration of asparagine (Asn) and lysine (Lys) of TRBC1 and TRBC2 would be the key of JOVI.1 selective binding. Up to date, the 3D structure of the JOVI.1 bound TRBC protein has not yet been reported, and how amino acid alteration affected the selectivity remained unknown. A lack of information regarding JOVI.1 binding mode towards TRBC1 and TRBC2 therefore became of interest. The atomistic understanding for the mechanistic action of how JOVI.1 antibody selectively interacts with TRBC1 is useful and able to facilitate the design of other more efficient and selective JOVI.1 antibodies.

To investigate the selective binding of JOVI.1 with the TRBC counterparts, the computational modeling approaches such as molecular docking and molecular dynamics simulation were introduced. These methods were proven to be successful in various molecular predictions such as drug-protein complexes^9–11^ and antibody design^12,13^. Molecular docking was generally used to generate the possible pose for the molecular binding between two entities based on docking score namely relative free binding energy or other ranking score types^14,15^, meanwhile, molecular dynamics simulation can fulfill the simulated effects due to surroundings such as temperature, pressure, solution ionic strength^11,16,17^, or even membrane environment^18,19^. In this study, we have performed computational modeling of TRBC1 and TRBC2 under dynamics conditions to visualize the effect of alternated Asn-Lys on the protein structure. We also investigated the JOVI.1-TRBC complex to propose its binding mode and binding selectivity via homology modeling and molecular docking.

## Results

### Epitope uniqueness of TRBC1 and TRBC2

**Figure 1:**
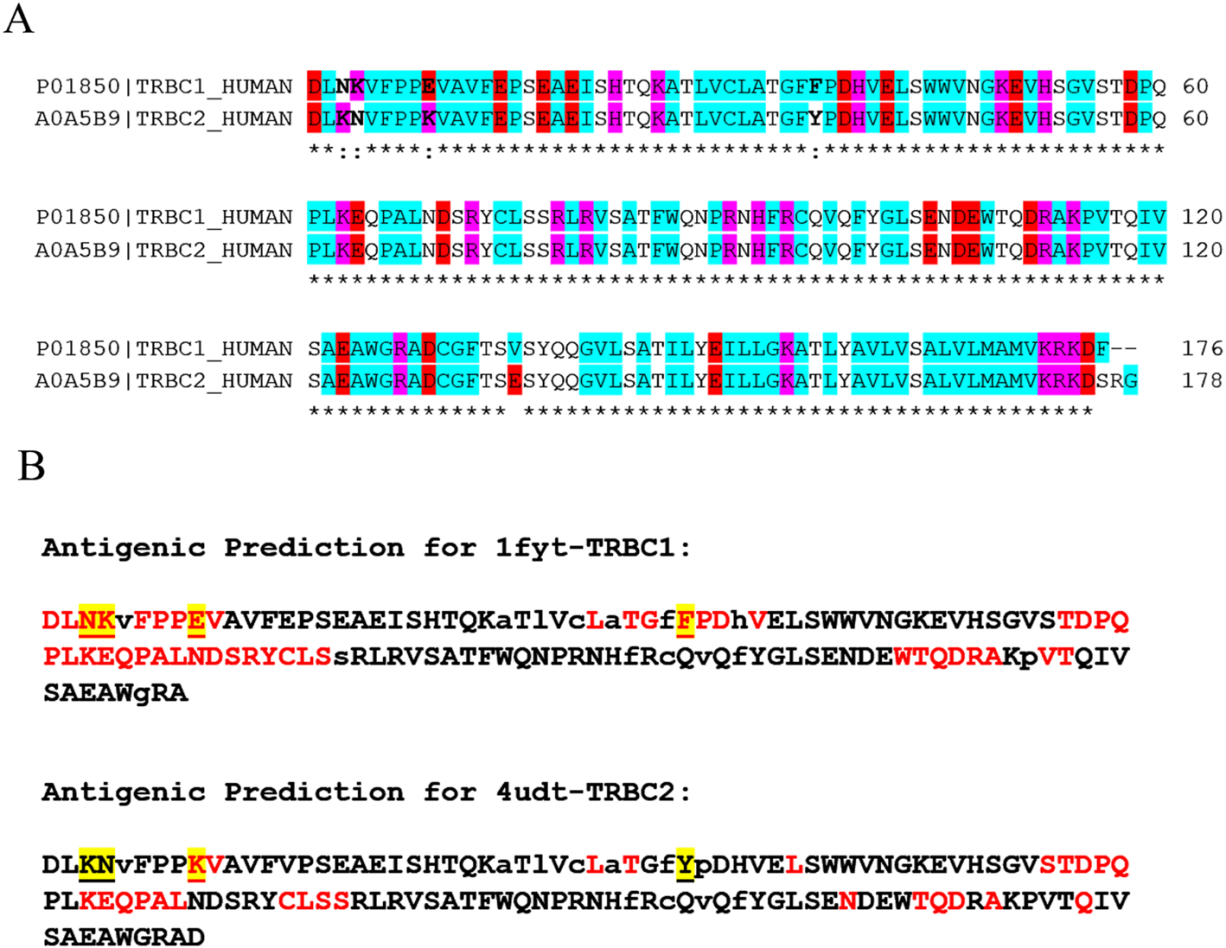
Sequence alignment and antigenic prediction of TRBC proteins. (**A**) Sequence alignment of both TRBC proteins. TRBC1 and TRBC2 sequences were taken from Uniprot ID P01850 and A0A5B9, respectively. The red, pink and light blue highlighted negatively charged, positively charged and non-polar amino acids. The asterisk (*) denoted the identical amino acid residues between TRBC proteins. (**B**) Antigenic prediction of both TRBC proteins. TRBC1 and TRBC2 sequences were taken from the crystal structure PDB code 1fyt and 4udt, respectively. The capital and lowercase letters indicated surface and buried residues. The red letter indicated epitope residues. The highlighted and underlined letters indicate unique residues.

Due to the specificity of JOVI.1 antibody towards only TRBC1, but not TRBC2, we tried to identify which TRBC1 antigenic determinant can be the selective residues for the antibody. TRBC1 and TRBC2 sequences showed that four amino acids are found to be conserved for each TRBC, including N3, K4, E9 and F36 for TRBC1, and K3, N4, K9 and Y36 for TRBC2 (Fig. 1A). Among these amino acids, K (lysine) and E (glutamic acid) are charged amino acids while N (asparagine) and Y (tyrosine) are neutral polar amino acid. In contrast, F (phenylalanine) is a non-polar aromatic amino acid. To identify possible epitopes for B cell of TRBC proteins, SEPPA 3.0 was used to determine the conformational discontinuous B cell epitope with the membrane protein and mouse immunity parameters. The analysis results demonstrated that N3K4 and F36P37D38 of TRBC1 could be categorized as epitopes for mouse B cell. Meanwhile, at the same position, K3N4 and Y36P37D38 of TRBC2 were not grouped as a target for mouse antibody (Fig. 1B). These results suggested that both two predicted epitopes may be selective regions for JOVI.1 antibody to distinguish TRBC1 from TRBC2.

### MD simulations of TRBC1 and TRBC2

**Figure 2:**
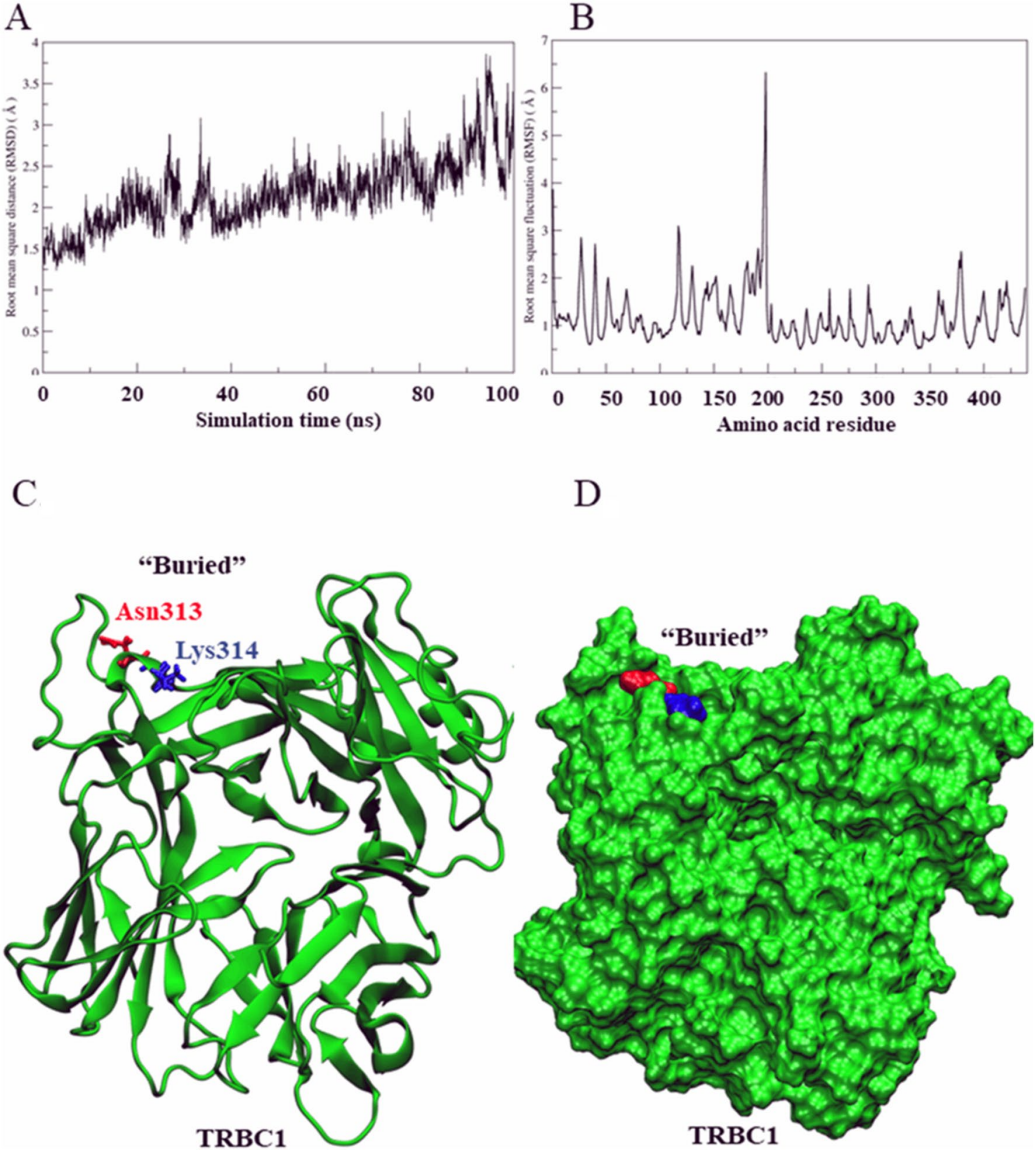
Simulated TRBC1 structure. (**A**) Root mean square distance (RMSD) of TRBC1. (**B**) Root mean square fluctuation (RMSF) of amino acid residues in TRBC1. (**C**) Positions of Asn313 (N3) and Lys314 (K4) in TRBC1. (**D**) The MD simulation suggested that the K4 sidechain was buried in the protein surface.

**Figure 3:**
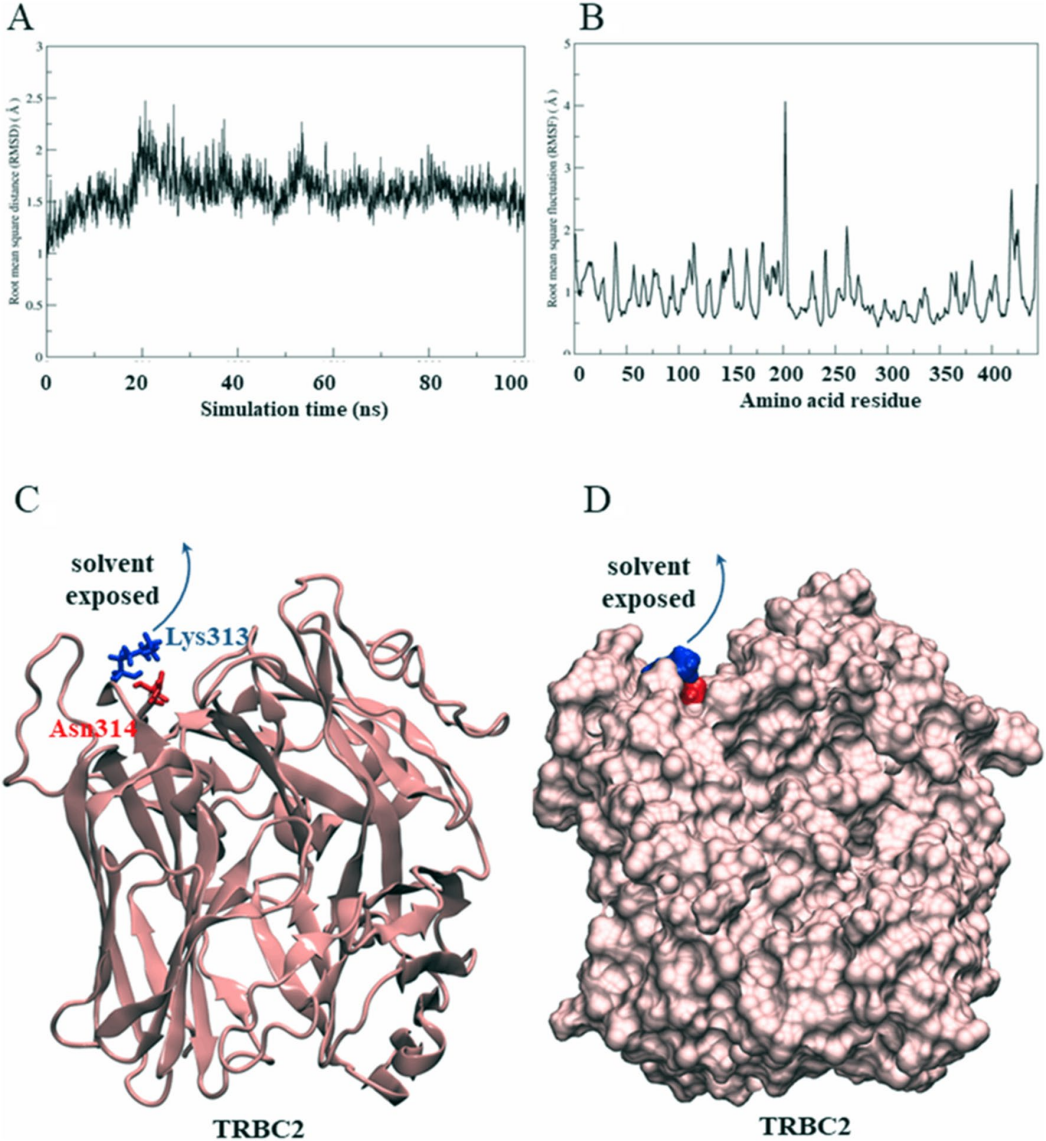
Simulated TRBC2 structure. (**A**) Root mean square distance (RMSD) of TRBC2. (**B**) Root mean square fluctuation (RMSF) of amino acid residues in TRBC2. (**C**) Positions of Asn313 (N3) and Lys314 (K4) in TRBC2. (**D**) The MD simulation suggested that the K4 sidechain came out from the protein surface and exposed to the aqueous environment.

In addition to crystal structure analysis, we also employed molecular dynamic (MD) simulation to visualize the difference of conformation for predicted epitope candidates. Both TRBC systems were stable throughout the simulation according to the root mean square distance (RMSD) plot (Figs. 2A and 3A). The flexibility of TRBC proteins was observed from root mean square fluctuation (RMSF). The result showed that an N-K alternation between two proteins indicated the orientation of lysine and asparagine. The MD simulation showed the 190th–200th residues in TRBC1 were more flexible than in TRBC2. Other regions remained similar in terms of flexibility (Figs. 2B and 3B). Moreover, we performed hydrogen bond analysis and considered H-bonds with higher than 80% occupancy were the conventional hydrogen bonds. The result showed that 27 conventional hydrogen bonds were found in TRBC1 structure while 39 conventional hydrogen bonds were discovered in TRBC2 (Supplementary information files 1 and 2). Up to this point, an N-K alternation could have an effect on structural flexibility of TRBC1 and TRBC2. Apart from the flexibility, N-K alternation of these TRBC proteins led to the conformation of lysine (K) sidechain. In TRBC1, lysine4 (K4) sidechain was found to be buried inside the protein surface (Fig. 2C,D), meanwhile in TRBC2, the equivalent lysine3 (K3) sidechain became a surface residue and solvent exposed (Fig. 3C,D).

### Predicted JOVI.1-antibody structure

**Figure 4:**
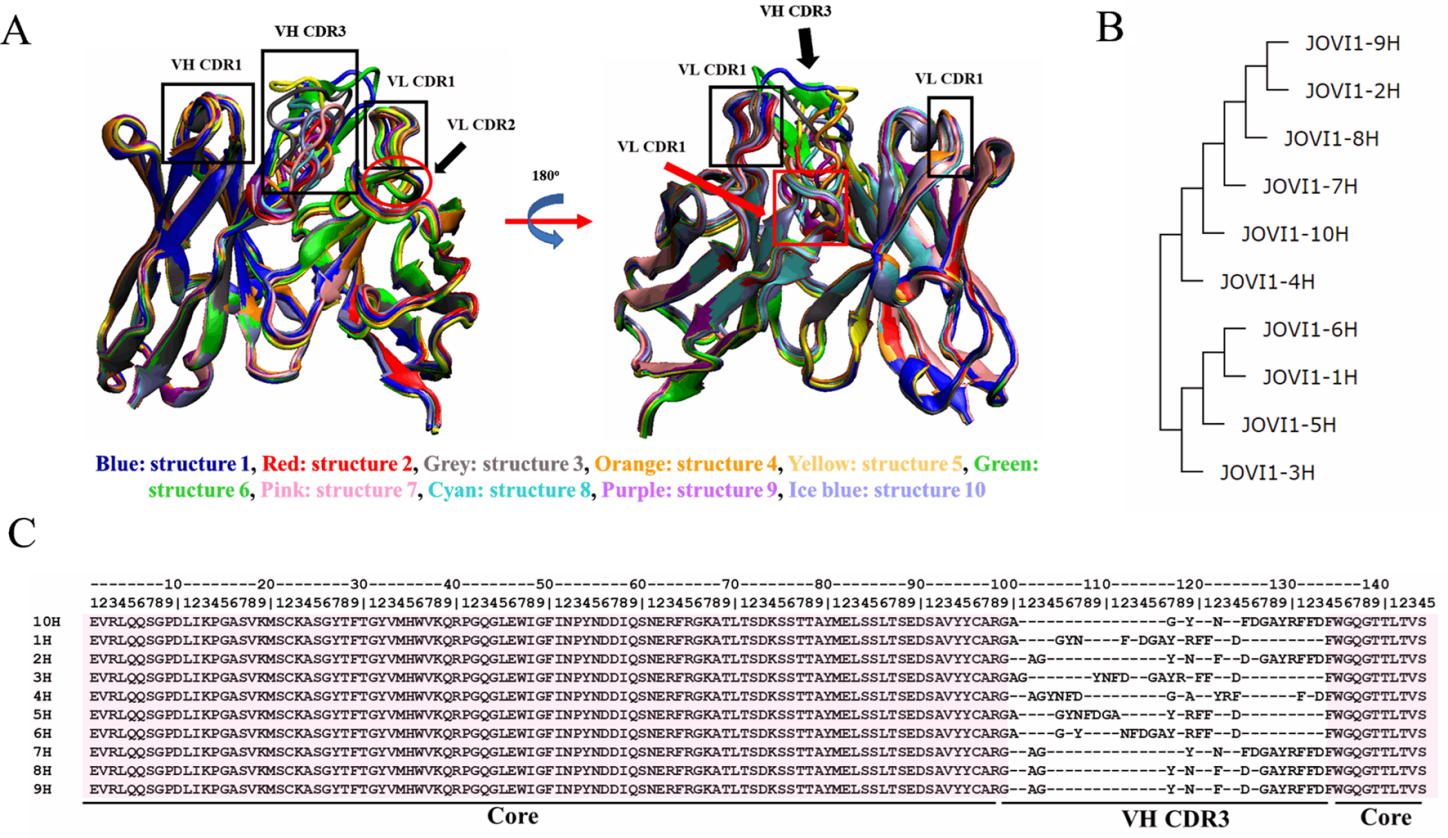
Predicted JOVI.1 antibody structure. (**A**) illustrated the structural alignment of ten proteins. The CDR regions from all ten conformers were identified. (**B**) showed phylogenetic tree of JOVI.1 predicted structures. (**C**) shower sequence alignment of all predicted structures, categorized into core structure, and VH CDR3 region.

Although many protocols for antibody modeling have been developed and benchmarked in the Antibody Modeling Assessment (AMA)^20^, RosettaAntibody is the only protocol that includes extensive conformational refinement focused in antibody degrees of freedom to create a structure with minimum energy that is appropriate for docking or design^21^. Therefore, this server was selected to predict the 3D structure of JOVI.1 scFv based on the sequence of six complementarity-determining regions. The framework sequence of variable region was used to identify the structure of known antibodies from Protein Data Bank with the structure of loops H1, H2, L1, L2, and L3. The CDR-H3 loop was modeled de novo to generate the ten models ranked by lowest energies. The structural alignment of the ribbon structures of ten JOVI.1 single-chain variable fragment models were illustrated in Fig. 4A). All CDR except CDR-H3 displayed the same pattern of domains and loops. Structural alignment among all ten predicted proteins investigated the similarity of CDR-H3 loop conformation of each structure. Two major clusters were found according to the structural phylogenetic tree of ten models based on CDR-H3 shape (Fig. 4B). Structure 1, 3, 5, and 6 were grouped into the same clusters with three levels of relationship while another one showed the separation of five layers of six models. The alignment also revealed the maximum pairwise residue distance of each residue of CDR-H3 more than (column with gaps) and within (no gaps) 4 angstroms (Fig. 4C).

### Molecular docking of predicted JOVI.1 single-chain variable fragments and TRBC1 protein

**Figure 5:**
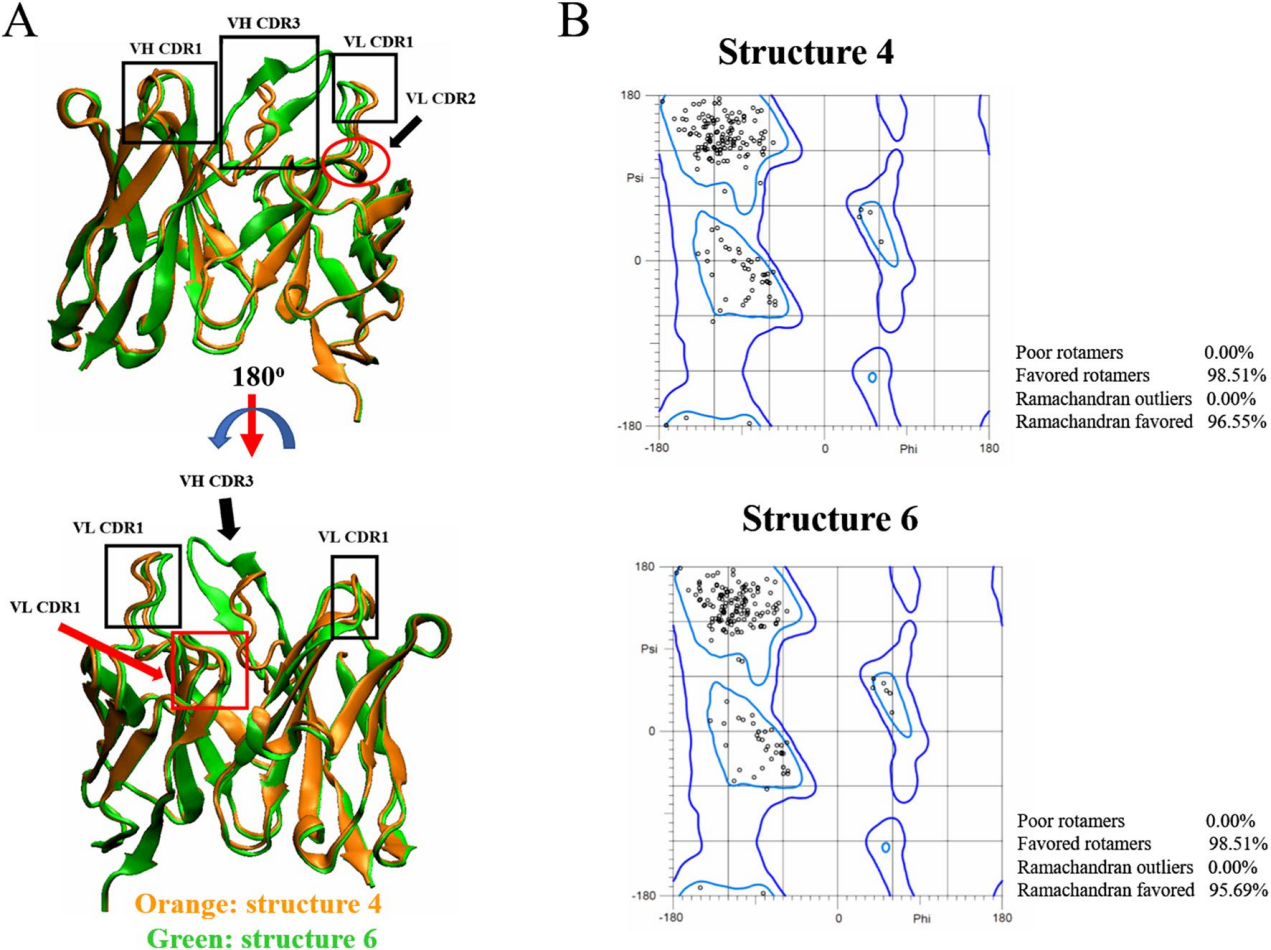
Structure analysis of selected JOVI.1-antibody structure. (**A**) Structural alignment of JOVI.1 structure 4 and structure 6, along with CDR region comparison. (**B**) Ramachandran plot of both structures. The plot indicated no outlier residue was found.

Ten models of JOVI.1 scFv were analyzed for TRBC1 interaction. Although all of these structures were ranked by the lowest energies of the CDR-H3 loop, we investigated the interaction of all structures by molecular docking based on the previous result described N4K5 (N3K4 in this work) of TRBC1 are the target for JOVI.1 antibody^6^. In addition to this report, the antigenic determinant prediction of TRBC1 mentioned earlier also demonstrated these two residues could be the selective region for antibodies to distinguish TRBC1 from TRBC2. After using antibody mode of Cluspro docking server, the results showed ten candidate clusters for each docking pair resulting in one hundred candidate clusters for ten models. We decided to develop the criteria to select the possible models that have the mode of interaction according to JOVI.1 antibody: (1) the candidate clusters must be the top three ranked by Cluspro weighted score, (2) the candidate clusters must have any interaction with N3K4 residues of TRBC1, (3) the three candidates will be selected according to predicted binding affinity (ΔG) provided by PRODIGY analysis, 4) the N3K4 interacting candidate clusters that have the members lower than 80% of the 1^st^ cluster ranked by Cluspro weighted score will be excluded. With these criteria, we found that structure 6 cluster 0, structure 4 cluster 1, and structure 4 cluster 0 were the top three candidate models of JOVI.1 scFv. Binding analysis by PRODIGY showed that structure 6 has the lowest binding affinity with the dG of -14.1 kcal/mol while structure 4 cluster 1 and 0 were the second and third lowest binding scores with -11.5 and -11.1 kcal/mol (Table 1). Structure 6 cluster 0 also showed the lowest predicted dissociation constant (K_d_) value (1.10 × 10^–10^ M at 37 °C) meanwhile structure 4 cluster 1 and 0 have K_d_ about 8.40 × 10^–9^ and 1.50 × 10^–8^ M at 37 °C (Table 1). The structure 6 and 4 were aligned (Fig. 5A) to investigate structural similarity. Ramachandran plot analysis by MolProbity^22^ showed that both JOVI.1 scFv models contained more than 98% and 95% of flavored rotamers and Ramachandran flavored protein geometry, respectively (Fig. 5B). No outlier was found from the structure. This suggested that angles of amino acids of both structures could be used for interaction study since it demonstrated the flavored empirical distribution.

**Table 1:** Binding analysis of predict JOVI.1 antibody with TRBC1.

	Cluster	Member	NK interaction	Predicted dissociation constant (K_d_) (M)	Relative binding energy (∆G) (kcal/mol)
Structure 1	0	132	No		
	1	77	No		
	2	54	No		
Structure 2	0	97	No		
	1	83	No		
	2	71	Yes	1.50 × 10^–6^	− 8.3
Structure 3	0	109	No		
	1	70	No		
	2	66	No		
Structure 4	0	65	Yes	1.50 × 10^–8^	− 11.1
	1	54	Yes	8.40 × 10^–9^	− 11.5
	2	51	No		
Structure 5	0	107	Yes	3.10 × 10^–7^	− 9.3
	1	54	No		
	2	44	No		
Structure 6	0	77	Yes	1.10 × 10^–10^	− 14.1
	1	76	No		
	2	63	No		
Structure 7	0	91	Yes	4.70 × 10^–7^	− 9.0
	1	87	No		
	2	50	Yes	3.50 × 10^–9^	− 12.0
Structure 8	0	90	No		
	1	70	No		
	2	57	No		
Structure 9	0	78	No		
	1	56	No		
	2	49	Yes	5.90 × 10^–8^	− 10.3
Structure 10	0	153	No		
	1	61	No		
	2	57	No

### Predicted JOVI.1 single-chain variable fragments with TRBC1

**Figure 6:**
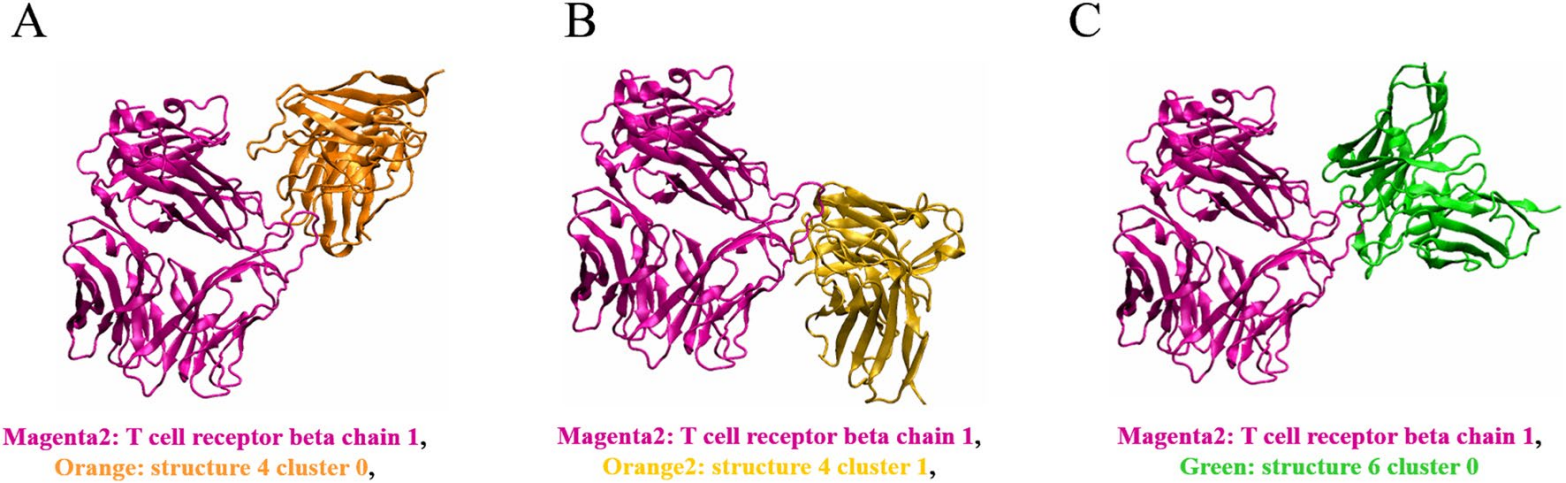
Predicted JOVI.1-TRBC1 complex structure. The three predicted complexes between JOVI.1 and TRBC1 were shown in (**A**), (**B**) and (**C**). TRBC1 structure was in magenta color.

To investigate the molecular insight of all three docking model candidates, MD simulation was then performed to mimic the dynamic behavior of the binding. We used five snapshots of MD simulation of each docking candidate at equilibrium state for analysis. By using PRODIGY, the interacting residues between JOVI.1 and TRBC1 of each snapshot were revealed. Table 2 showed the interacting residues that were found in 4/5 snapshots of the docked model. Structure 6 showed the highest frequency of chemical bonding of CDR with 31 interactions to 16 residues of TRBC1. The major difference of this structure is the bond between CDR-H2/H1 and R112 which is found only in structure 6 docking. The main interaction was observed in CDR-H2 forming 9 interactions while the second frequency was found in CDR-H3 with 8 bonding. Interestingly, the interaction of structure 4 of both clusters were found in CDR-H3 that formed 9 bonds and 6 bonds for cluster 0 and cluster 1 of structure 4, respectively. However, cluster 0 showed the higher number of TRBC1 residues to be formed the chemical bonds with 26 interactions while 10 interactions were observed in cluster 1 of structure 4. Moreover, the highest number of unique interactions was noticeable in cluster 0 model with seven residues while two specific interactions were found in cluster 1 of structure 4 (Table 2). Surprisingly, all docked models not only interacted with unique residues of TRBC1, K4 (Lys314), but also formed the chemical bonding to D1 (Fig. 6). This suggested that these residues of TRBC1 are important for JOVI.1 interaction.

**Table 2:** Predicted interacting residue of JOVI.1 with TRBC1.

Residues	Structure 4 cluster 0	Structure 4 cluster 1	Structure 6 cluster 0
ASP1	H1, H2, H3	H2	H1, H3
LEU2		H3	H3
ASN3		H2	H1, H2, H3
LYS4	H2, H3	H2	H1, H2, H3
PHE6	H3, L1,		H2,
PRO7	L1		
GLU9	L1		
THR33	L1,		
GLY34	L1,		
PHE36	H2		H2
GLU64	H3, L1		H2,
GLN65	L3		
LEU68	H2, H3		
ASN69	H2		
ASP70	H2, H3		H1, H2,
SER71			H2,
ARG72	H3, L1, L3		H2
ASN105		H3	
ASP106		H3	
GLU107		H3, L1	H3, L1, L2
TRP108		H3,	H3, L1
THR109		H3	H3, L1, L2, L3
GLN110	H3,		H3, L1
ASP111	H3, L2		L1, L3
ARG112			H2, L1

*H* Complementarity-determining regions heavy chain, *L* Complementarity-determining regions light chain.

Moreover, we calculated the relative binding energy of each docking structure after MD simulation. The result demonstrated that Structure 4 cluster 1 showed the lowest binding energy compared to other models (Table 3). This pair of JOVI.1 scFv and TRBC1 gave the ∆G value of -50.88 ± 0.43 and -32.25 ± 0.32 kcal/mol for MM/PBSA and MM/GBSA, respectively. Structure 6 cluster 0 displayed the second in rank of lowest binding energy with the values of -35.71 ± 0.84 and -30.92 ± 0.43 kcal/mol for MM/PBSA and MM/GBSA while structure 4 cluster 0 showed the highest value of ∆G (Table 3). Interestingly, the relative binding energy derived from MD simulation demonstrated the obvious different value when compared to ∆G calculated by PRODIGY server.

**Table 3:** Predicted ∆G of each interaction pair between predicted JOVI.1 structure and TRBC1.

Calculation methods	∆G of structure 4 cluster 0 (kcal/mol)	∆G of structure 4 cluster 1 (kcal/mol)	∆G of structure 6 cluster 0 (kcal/mol)
PRODIGY prediction	− 11.1	− 11.5	− 14.1
MM/PBSA prediction	− 32.77 ± 0.99	− 50.88 ± 0.43	− 35.71 ± 0.84
MM/GBSA prediction	− 22.59 ± 0.76	− 32.25 ± 0.32	− 30.92 ± 0.43

## Discussion

PTCL is a type of non-Hodgkin’s lymphoma accounting for 6–10% of all cases. This type of cancer originates from mature T cells or NK cells, and carries a poor prognosis^23^. As no gold standard for PTCL treatment was established, the combination of chemotherapeutic drugs, such as CHOP, is generally chosen for PTCL patients^24^. Unfortunately, patients showed unsatisfactory responses even when the new drugs have been administered^25^. Moreover, relapse is usually found although the autologous stem cell transplant may improve progression-free survival (PFS)^26^. New additional strategy apart from chemotherapeutic drugs is thus useful to improve the response rate of PTCL patients. Recently, CAR T cells for cancer treatment have been successfully translated to T cell malignancies^6^. The TRBC1 specific antibody (JOVI.1) was applied to generate a selective CAR T cell, binding only TRBC1 expressing malignant T cells, but not TRBC2-containing normal cells. The antibody clone has already been characterized and the sequence of CDR has well been documented. However, the basic interaction and specificity of the clone to TRBC1 are unknown.

Our study provided the first computational modeling to predict the binding mode of an anti-TRBC1 antibody clone (JOVI.1) toward the TRBC1. We used modeled scFv as a binding part of JOVI.1 antibody since many studies demonstrated this fragment was used as a representative structure for antibody-antigen interaction study. For example, Zhang et al. used scFv of monoclonal antibody against pefloxacin for interaction investigation^27^. Another study also used scFv for interaction discovery of antibody-antigen complexes for their anti-FGF2 3F12E7 monoclonal antibody both in vitro and in vivo^28^. Recently, a docking study was also applied to scFv to mimic the specific binding of the IgG1 format to membrane-bound CoV-2 spike protein^29^. Moreover, scFv format of the JOVI.1 antibody has been applied to the CAR T cell receptor successfully targeting the TRBC1 expressing cancer cell^6^. Despite only the algorithmic possible interaction demonstrated, the selection criteria of the candidate model were based on the previous experimental data^6,30^. First of all the antigenic determinant regions of both TRBC1 and TRBC2 were predicted to identify and confirm what region can be a target for mouse B cell which resulted in the generation of TRBC specific antibody. Interestingly, N3K4 region, previously reported as selective amino acids of TRBC1 for JOVI.1, was found to be an immunogenic sequence for mouse B cell receptor repertoire while K3N4 of TRBC2 was not immunogenic for mouse immunity. This emphasized the possibility of the N3K4 region in TRBC1 for the generation of the JOVI.1 clone since the antibody was from immunized mice^31^.

Secondly, we also performed the molecular dynamic (MD) simulation of TRBC1 and TRBC2 by using crystal structure as a template to visualize the natural behavior of the protein in aqueous environment^32^. A possible key factor for JOVI.1 selection would be the specific lysine-arginine (N-K) region. The surrounding site of N-K residues in TRBC1/2 was also speculated to be a factor of difference in lysine (K) orientation. In addition, the difference of the lysine sidechain orientation was observed from the crystal structure. From our study, the MD simulations showed the different conformation between N3K4 (TRBC1) and K3N4 (TRBC2). The observed lysine sidechain pose gave a hint that this lysine would play a key for TRBC1/2 antibody binding specificity. In addition, the evidence from epitope prediction and structural study of TRBC1 and TRBC2, together with the previously reported data, pointed out the possible role of these amino acids as a distinctive region for JOVI.1 binding.

Nowadays, because of the increasing computing power together with the expanded database of protein sequence and structure, the computational methods have been intensively used to predict structure of the protein with more accuracy^33^. In this study, we used RosettaAntibody3 for JOVI.1 structure prediction with known CDR sequence to generate template-based structure of several antibodies, subjected to interaction analysis and protein redesign^34,35^. Although most steps of algorithmic calculation were performed by homology modeling from known canonical structure of antibody, the challenge of this work is the prediction of CDR-H3 loop which showed a variety in length, sequence and structure due to V(D)J recombination and somatic hyper-mutation^36,37^. Fortunately, the TRBC1 specific target residues to JOVI.1 were revealed without the overall binding mode^6^. Moreover, the structural analysis of TRBC1 and TRBC2 also revealed the difference in flexibility and N3K4 surface region among both structures. The variation of flexibility may be due to the different numbers of conventional hydrogen found in both structures (SI.1). Therefore, we applied these reported data for structure selection after molecular docking, and also included the number of members in each cluster and predicted dissociation constant (K_d_) as the criteria. The higher number of members and K_d_ predicted the higher binding possibility and binding affinity^14,38,39^. As a result of the prediction, three candidates from two structures were acquired with K_d_ values ranging from 1.5 × 10^–8^ to 1.1 × 10^–10^ M. Interestingly, this range of K_d_ is close to the K_d_ from in vitro study (4.2 × 10^–10^ M)^6^. However, MD simulation of all three candidates showed the major different binding energy compared to values obtained from PRODIGY. This may be due to the presence of water molecules in the modeling system which may affect the binding force between JOVI.1 and TRBC1. In addition to K_d_ and binding energy calculation, Ramachandran plot indicated the acceptable phi and psi torsion angles of amino acids in modeled structures.

Finally, another key amino acid of JOVI.1 used for its specific interaction in all three binding modes of TRBC1 was revealed, D1. The obtained information could be applied for the JOVI.1 modification both as a target for antibody redesign or a residue that cannot be touched. In summary, we presented proposed molecular insight for JOVI.1-TRBC1 binding mode. Our modeling method was based on the experimental data as the concept for the structure selection. This may fulfill the gap of knowledge of this antibody that has been used as a prototype for CAR T cell development against PTCL, which threatens the lives of patients.

## Methods

### Sequence analysis and epitope prediction

The amino acid sequences of TRBC1 and TRBC2 were retrieved from The Universal Protein Resource (UniProt)^40^ and aligned together using the Clustal Omega program^41^. TRBC1(PDB: 1FYT) and TRBC2 (PDB: 4UDT) were retrieved from Protein Data Bank^42^. FoldX was chosen to perform structural mutation to prepare the structures of TRBC1 and TRBC2 from original sequence^43^. Epitope residue prediction of TRBC1 and TRBC2 proteins with its antibody from crystal structure was carried out using Spatial Epitope Prediction of Protein Antigens (SEPPA) 3.0^44^, relied on three parameters: propensity index avgr, relative ASA Aprefr and ratio of glycosylation triangles^44^. Subcellular localization of antigen was defined as membrane, and mouse was chosen as the species of the immune host.

### Structure preparation of JOVI.1 antibody

The predicted JOVI.1 antibody Fv region was generated using RosettaAntibody3 program in The Rosetta Online Server That Includes Everyone (ROSIE)^45^. Briefly, amino acid sequences of heavy chain (VH) and light chain (VL) variable domains of JOVI.1 antibody were uploaded onto RosettaAntibody application, which used a three-steps protocol to model the Fv region of the antibody^46^. Firstly, the amino acid sequence of VH and VH of JOVI.1 was subjected to the BLAST protocol to select the template from the crystal structure of antibody in Protein Data Bank^42^. The selected template of five complementarity-determining regions (CDRs; L1, L2, L3, H1 and H2) and the frameworks (FRL and FRH) were searched independently. In the second stage, all selected CDRs and framework regions were then grafted and optimized by minimizations, random torsional sampling and cyclic coordinate descent (CCD)^47^ resulting in the crude assembled antibody structure. Finally, the CDR-H3 loop was modeled de novo with the next-generation kinematic loop closure (KIC) algorithm in a low-resolution step^48^. VL/VH orientations, side chain and loop backbone were then optimized using a Rosetta protocol. The ten lowest scoring homology models of antibody were used for molecular docking.

### Molecular docking

A ClusPro web server with Antibody mode was used to screen the interaction between 10 JOVI.1 scFv region modeled structures, and TRBC1 or TRBC2. The interaction energy from billions of sampling conformations is calculated and the structures of the 1000 lowest energies will be grouped according to root-mean-square deviation (RMSD) to generate the clusters. The largest clusters are selected as the most likely models of the complex, which will be refined using energy minimization^14^. In the Antibody mode, ClusPro considers the asymmetric interaction between Phe-Leu residues in both antigen and antibody^39^ and also allows for the masking of the surface of the antibody except for the CDR residues^14^. The docking was performed by using 10 predicted structures of JOVI.1 antibody and the structure of TRBC1 and TRBC2 retrieved from Protein Data Bank^42^ including chain D and E of TRBC1 (PDB: 1FYT)^7^ and chain A and B of TRBC2 (PDB: 4UDT)^8^. All clusters were analyzed by PRODIGY (PROtein binDIng enerGY prediction)^49^ for dissociation constant (Kd), value of the binding affinity (ΔG) and interaction residues.

### Molecular dynamics simulation

The protonation state of TRBC1 or TRBC2 was considered based on the aforementioned crystal structure using PDB2PQR webserver^50^. Each ionizable amino acid in the complex structure was assigned at pH 7.0 based on AMBER20 force field^51^. Finally, all histidine was assigned as a singly protonated histidine (neutral charge) at epsilon-nitrogen position (HIE). Other ionizable amino acids were set as a default state at pH 7.0: aspartate (ASP) and glutamate (GLU) were -1e charged, while lysine (LYS) and arginine (ARG) were + 1e charged. The protein was solvated by TIP3P water from a distance of 14 Å. The complex was neutralized using 8 sodium ions, and 38 sodium chloride pairs were added to generate 0.10 M NaCl solution. The TRBC system consisted of approximately 21,000 waters, 46 sodium ions, and 38 chloride ions. The system was energetically minimized using 2,000 steepest descent steps and 1000 conjugated gradient steps, with a cutoff of 16 Å under a periodic boundary condition using the pmemd.cuda module. The minimized structure was taken for the canonical (NVT) equilibration at 310 K. The atomic position of the protein was restraint. The harmonic force constants were sequentially reduced as follows: 200, 100, 50, 20, and 10 kcal mol^-1^ Å^-2^. All nonbonded and electrostatic interactions were computed using a cutoff of 16 Å. All H–X bonds were constrained using SHAKE algorithm^52^. An NVT simulation with each respective force constant lasted 200 ps using a 1-fs time step. Finally, the NVT simulation yielded 1.0 ns. The temperature of 310 K was controlled using Langevin dynamics^53^. The last snapshot of the NVT ensemble was changed into the isobaric-isothermal (NPT) ensemble of 1.0 atm (1.013 Bar) and 310 K. Both temperature and pressure were regulated using Berendsen algorithm^54^. The 100 ns MD simulation was obtained using a time step of 2 fs for 50,000,000 steps. The 1000 equidistant snapshots were obtained from 100 ns MD trajectory. The first 500 MD snapshots were then discarded and the last 500 MD snapshots were acquired for structural analysis.

### MD trajectory analysis

The root mean square displacement (RMSD) was plotted from 1000 MD snapshots with respect to the starting coordinate. The RMSD computation was performed using Visual Molecular Dynamics (VMD) package^55^. The root mean square fluctuation (RMSF) of alpha carbon at the protein backbone was calculated from the last 500 MD snapshots. The visualization of the protein structure and protein–protein interaction was performed using VMD.

## Supplementary Information

Supplementary Information 1.

Supplementary Information 2.
