# Supplementary material for: Computational discovery of binding mode of anti-TRBC1 antibody and predicted key amino acids of TRBC1
Source: Sci Rep. 2022 Feb 2;12:1760. doi: 10.1038/s41598-022-05742-6 (PMC8810837; doi:10.1038/s41598-022-05742-6)
Supplement: Supplementary file 1 — Supplementary Information 1. [file 41598_2022_5742_MOESM1_ESM.docx]

**TRBC1-H-bond**

#Acceptor DonorH Donor Frames Frac AvgDist AvgAng

MET_250@O SER_245@HG SER_245@OG 976 0.9760 2.6998 163.4813

GLN_281@O TYR_285@HH TYR_285@OH 960 0.9600 2.7144 163.6766

ASP_84@O TYR_88@HH TYR_88@OH 945 0.9450 2.7301 165.3955

ASP_368@OD2 SER_386@HG SER_386@OG 941 0.9410 2.7096 162.9305

PHE_89@O TYR_35@H TYR_35@N 904 0.9040 2.8275 158.5372

GLU_63@O THR_76@H THR_76@N 880 0.8800 2.8377 162.3707

LEU_32@O TYR_49@H TYR_49@N 879 0.8790 2.8222 157.4276

TRP_230@O ILE_242@H ILE_242@N 879 0.8790 2.8452 159.8101

VAL_18@O THR_76@HG1 THR_76@OG1 863 0.8630 2.7304 157.6142

GLU_327@O SER_331@HG SER_331@OG 861 0.8610 2.7029 164.0311

GLU_329@O THR_333@HG1 THR_333@OG1 858 0.8580 2.7297 159.8399

LEU_286@O TYR_231@H TYR_231@N 853 0.8530 2.8409 159.6218

GLU_100@O SER_93@HG SER_93@OG 843 0.8430 2.6579 154.7297

ASP_234@OD2 ARG_232@HH21 ARG_232@NH2 842 0.8420 2.8130 162.1987

VAL_430@O VAL_407@H VAL_407@N 842 0.8420 2.8457 160.8197

SER_261@O ILE_273@H ILE_273@N 840 0.8400 2.8397 158.7209

PHE_345@O TYR_383@H TYR_383@N 833 0.8330 2.8468 161.9850

TYR_285@OH TYR_260@HH TYR_260@OH 828 0.8280 2.8170 162.9076

GLU_87@O GLN_37@H GLN_37@N 828 0.8280 2.8344 157.0594

SER_204@OG THR_304@HG1 THR_304@OG1 821 0.8210 2.7848 160.6291

ASP_152@O SER_177@HG SER_177@OG 819 0.8190 2.7043 162.3373

ASP_234@OD2 SER_283@HG SER_283@OG 811 0.8110 2.7124 163.1423

ARG_422@O TRP_418@HE1 TRP_418@NE1 808 0.8080 2.8192 155.6743

PHE_395@O ASN_401@HD22 ASN_401@ND2 806 0.8060 2.8378 160.6818

GLU_226@O ARG_264@HH12 ARG_264@NH1 804 0.8040 2.8263 153.2465

ALA_175@O CYX_134@H CYX_134@N 801 0.8010 2.8472 161.1534

TYR_124@O LEU_135@H LEU_135@N 795 0.7950 2.8553 159.4622

SER_387@O CYX_340@H CYX_340@N 793 0.7930 2.8552 158.1310

VAL_133@O LEU_126@H LEU_126@N 789 0.7890 2.8516 160.7511

THR_159@O SER_172@H SER_172@N 785 0.7850 2.8366 155.8883

VAL_339@O PHE_323@H PHE_323@N 784 0.7840 2.8385 159.9352

TRP_34@O LEU_46@H LEU_46@N 772 0.7720 2.8564 155.5531

LYS_267@O ARG_264@HH21 ARG_264@NH2 771 0.7710 2.8278 155.8718

ARG_404@O TRP_355@H TRP_355@N 769 0.7690 2.8409 155.8636

LEU_75@O LEU_20@H LEU_20@N 769 0.7690 2.8556 160.0132

GLN_37@OE1 GLN_233@HE21 GLN_233@NE2 765 0.7650 2.8361 161.7677

LEU_385@O ALA_342@H ALA_342@N 763 0.7630 2.8702 162.6102

PHE_73@O TRP_34@HE1 TRP_34@NE1 760 0.7600 2.8491 159.7518

ASP_368@OD1 ARG_388@HH22 ARG_388@NH2 759 0.7590 2.8159 154.2564

ARG_264@O LYS_249@H LYS_249@N 759 0.7590 2.8377 157.7641

LEU_389@O LEU_338@H LEU_338@N 753 0.7530 2.8501 156.1378

ALA_434@O PHE_403@H PHE_403@N 747 0.7470 2.8606 160.4163

LEU_217@O LEU_272@H LEU_272@N 747 0.7470 2.8623 158.3879

TYR_285@O THR_304@H THR_304@N 746 0.7460 2.8466 152.8514

PHE_139@O ASN_171@HD22 ASN_171@ND2 744 0.7440 2.8494 163.3293

SER_387@OG TRP_354@HE1 TRP_354@NE1 743 0.7430 2.8667 158.7777

SER_353@O GLN_406@H GLN_406@N 740 0.7400 2.8676 160.8558

GLU_252@O PHE_244@H PHE_244@N 738 0.7380 2.8714 161.1608

SER_366@O ARG_388@H ARG_388@N 732 0.7320 2.8318 153.5142

SER_170@O LEU_161@H LEU_161@N 732 0.7320 2.8378 155.3310

ASP_162@OD1 ARG_164@HH21 ARG_164@NH2 729 0.7290 2.7768 158.4672

LEU_135@O TYR_124@H TYR_124@N 729 0.7290 2.8719 160.3425

SER_302@O GLN_202@HE22 GLN_202@NE2 728 0.7280 2.8442 163.1610

VAL_315@O ARG_422@HH12 ARG_422@NH1 724 0.7240 2.8425 158.0690

PRO_425@O LEU_412@H LEU_412@N 723 0.7230 2.8575 160.8456

GLN_428@O PHE_409@H PHE_409@N 723 0.7230 2.8648 158.5626

LYS_359@O VAL_356@H VAL_356@N 722 0.7220 2.8599 160.8030

HIE_81@O ASP_84@H ASP_84@N 719 0.7190 2.8581 159.9767

THR_304@O TYR_285@H TYR_285@N 719 0.7190 2.8611 159.7730

LEU_20@O LEU_75@H LEU_75@N 704 0.7040 2.8415 157.1563

TYR_88@O GLN_5@HE21 GLN_5@NE2 703 0.7030 2.8542 160.4464

ALA_336@O VAL_391@H VAL_391@N 703 0.7030 2.8631 162.5696

GLY_364@O ARG_390@H ARG_390@N 698 0.6980 2.8539 162.1023

THR_108@O TYR_88@H TYR_88@N 694 0.6940 2.8633 160.7056

GLU_329@OE2 ARG_390@HH21 ARG_390@NH2 685 0.6850 2.7897 151.6782

VAL_160@O SER_366@HG SER_366@OG 684 0.6840 2.7135 157.0229

GLN_44@O VAL_36@H VAL_36@N 684 0.6840 2.8765 157.9823

CYX_90@O GLY_105@H GLY_105@N 681 0.6810 2.8557 155.9034

GLU_329@OE1 THR_337@H THR_337@N 680 0.6800 2.8019 153.5572

ALA_86@O LEU_110@H LEU_110@N 680 0.6800 2.8696 160.4249

ALA_432@O CYX_405@H CYX_405@N 679 0.6790 2.8786 161.2143

PHE_136@O ALA_173@H ALA_173@N 678 0.6780 2.8714 153.7290

ALA_173@O PHE_136@H PHE_136@N 678 0.6780 2.8742 160.2337

LEU_272@O LEU_217@H LEU_217@N 676 0.6760 2.8630 157.5636

ALA_91@O PHE_33@H PHE_33@N 672 0.6720 2.8669 157.1240

VAL_36@O GLN_44@H GLN_44@N 671 0.6710 2.8684 158.3106

ASP_234@OD1 ARG_232@HE ARG_232@NE 667 0.6670 2.8350 153.6081

ASN_23@O THR_4@H THR_4@N 662 0.6620 2.8631 159.2848

THR_201@O VAL_220@H VAL_220@N 661 0.6610 2.8802 160.3160

GLU_351@O GLN_408@H GLN_408@N 658 0.6580 2.8661 161.9316

GLU_226@OE1 SER_291@HG SER_291@OG 652 0.6520 2.6328 164.7144

GLU_374@O TYR_154@HH TYR_154@OH 652 0.6520 2.7348 160.6970

ARG_382@O GLU_374@H GLU_374@N 650 0.6500 2.8482 157.5833

PHE_244@O GLU_252@H GLU_252@N 649 0.6490 2.8748 157.1484

ASP_368@OD2 SER_386@H SER_386@N 647 0.6470 2.8392 152.4228

CYX_287@O GLY_301@H GLY_301@N 646 0.6460 2.8588 159.0821

ASN_227@O SER_290@H SER_290@N 646 0.6460 2.8637 151.6126

VAL_391@O ALA_336@H ALA_336@N 645 0.6450 2.8534 155.2597

TRP_176@O TYR_154@H TYR_154@N 643 0.6430 2.8716 159.3750

TYR_35@O PHE_89@H PHE_89@N 643 0.6430 2.8737 163.1229

PHE_33@O ALA_91@H ALA_91@N 640 0.6400 2.8560 152.0908

ILE_192@O ASN_144@H ASN_144@N 637 0.6370 2.8511 159.2974

ILE_113@O GLU_14@H GLU_14@N 636 0.6360 2.8589 156.1624

PHE_403@O ALA_434@H ALA_434@N 634 0.6340 2.8676 155.3223

VAL_215@O LEU_274@H LEU_274@N 630 0.6300 2.8764 162.0750

GLU_327@O SER_331@H SER_331@N 629 0.6290 2.8557 156.3713

SER_172@O THR_159@H THR_159@N 628 0.6280 2.8665 159.4010

SER_384@O LEU_372@H LEU_372@N 626 0.6260 2.8636 159.5582

TYR_154@O TRP_176@H TRP_176@N 622 0.6220 2.8796 155.4535

THR_299@O SER_289@H SER_289@N 622 0.6220 2.8808 154.1550

THR_76@O GLU_63@H GLU_63@N 618 0.6180 2.8817 159.5847

GLU_319@O THR_343@H THR_343@N 617 0.6170 2.8719 159.1445

LEU_338@O LEU_389@H LEU_389@N 616 0.6160 2.8748 160.2244

ASN_401@OD1 ASN_357@HD21 ASN_357@ND2 615 0.6150 2.8590 160.8719

SER_245@O MET_228@H MET_228@N 615 0.6150 2.8823 155.2408

VAL_12@O ILE_113@H ILE_113@N 614 0.6140 2.8688 157.0999

THR_343@O GLU_319@H GLU_319@N 613 0.6130 2.8712 159.4815

ALA_16@O ALA_80@H ALA_80@N 612 0.6120 2.8688 161.2467

SER_289@O THR_299@H THR_299@N 604 0.6040 2.8809 158.3964

GLU_268@O GLN_221@H GLN_221@N 604 0.6040 2.8833 156.9666

ALA_80@O GLY_15@H GLY_15@N 599 0.5990 2.8495 158.2839

ASN_357@OD1 HIE_402@H HIE_402@N 595 0.5950 2.8728 158.5896

GLU_329@OE2 THR_337@HG1 THR_337@OG1 594 0.5940 2.7301 151.3318

PHE_182@O SER_132@HG SER_132@OG 588 0.5880 2.7000 163.1347

SER_72@O LYS_67@H LYS_67@N 587 0.5870 2.8767 160.1764

PHE_316@O PHE_346@H PHE_346@N 587 0.5870 2.8882 157.8695

PHE_346@O PHE_316@H PHE_316@N 585 0.5850 2.8793 161.6255

ASN_99@OD1 SER_95@HG SER_95@OG 577 0.5770 2.7207 160.3172

THR_103@O VAL_92@H VAL_92@N 577 0.5770 2.8835 155.7152

ALA_393@O GLN_397@H GLN_397@N 576 0.5760 2.8582 157.7576

ALA_277@O GLY_212@H GLY_212@N 574 0.5740 2.8686 159.1750

TRP_355@O ARG_404@H ARG_404@N 573 0.5730 2.8636 157.8701

VAL_56@O ALA_64@H ALA_64@N 573 0.5730 2.8739 152.9209

ARG_232@O ARG_240@H ARG_240@N 573 0.5730 2.8755 159.5686

LEU_47@O TRP_34@H TRP_34@N 572 0.5720 2.8781 155.9454

ALA_288@O PHE_229@H PHE_229@N 572 0.5720 2.8872 158.3520

LEU_372@O SER_384@H SER_384@N 571 0.5710 2.8655 152.5229

ALA_342@O LEU_385@H LEU_385@N 567 0.5670 2.8785 157.8726

MET_228@O SER_245@H SER_245@N 566 0.5660 2.8658 151.9079

GLY_7@O THR_108@HG1 THR_108@OG1 564 0.5640 2.7349 161.8667

GLU_329@O THR_333@H THR_333@N 564 0.5640 2.8689 152.8287

ARG_240@O ARG_232@H ARG_232@N 562 0.5620 2.8792 162.4522

ASP_162@OD2 ARG_164@HE ARG_164@NE 559 0.5590 2.8417 158.3257

VAL_28@O SER_26@HG SER_26@OG 558 0.5580 2.7170 159.3559

LYS_209@O VAL_309@H VAL_309@N 555 0.5550 2.8833 158.8685

THR_54@O PHE_66@H PHE_66@N 553 0.5530 2.8805 161.2270

CYX_219@O PHE_270@H PHE_270@N 551 0.5510 2.8914 156.3310

THR_4@O ASN_23@H ASN_23@N 546 0.5460 2.8881 158.3578

LEU_207@O THR_307@H THR_307@N 545 0.5450 2.8864 159.9044

SER_8@O ARG_109@H ARG_109@N 544 0.5440 2.8785 161.7174

ASP_167@OD1 ARG_164@HH12 ARG_164@NH1 543 0.5430 2.7881 156.3647

LYS_77@O VAL_18@H VAL_18@N 542 0.5420 2.8762 162.3872

THR_282@OG1 VAL_308@H VAL_308@N 539 0.5390 2.8919 162.7601

CYX_184@O PHE_188@H PHE_188@N 537 0.5370 2.8402 159.9178

GLN_406@O SER_353@H SER_353@N 536 0.5360 2.8913 157.7479

ASN_189@OD1 SER_191@HG SER_191@OG 535 0.5350 2.6861 163.0927

ASN_280@O ARG_232@HH12 ARG_232@NH1 535 0.5350 2.8407 154.8697

ARG_305@O LEU_207@H LEU_207@N 535 0.5350 2.8747 156.1446

PHE_270@O CYX_219@H CYX_219@N 535 0.5350 2.8875 158.4537

ASP_368@OD1 ARG_388@HH11 ARG_388@NH1 532 0.5320 2.8041 151.8904

TRP_396@O ASN_398@H ASN_398@N 532 0.5320 2.8753 144.8356

THR_337@OG1 ARG_390@HE ARG_390@NE 530 0.5300 2.8857 157.3844

SER_278@O GLN_281@H GLN_281@N 528 0.5280 2.8863 157.0557

ALA_321@O LEU_341@H LEU_341@N 522 0.5220 2.8949 159.9595

ASP_234@OD1 ARG_240@HH21 ARG_240@NH2 521 0.5210 2.7883 158.6870

THR_367@OG1 SER_387@HG SER_387@OG 519 0.5190 2.7942 156.0660

ASN_401@O GLY_436@H GLY_436@N 518 0.5180 2.8718 157.9660

GLN_420@OE1 ARG_422@HH21 ARG_422@NH2 509 0.5090 2.8146 150.9815

ARG_109@O VAL_10@H VAL_10@N 508 0.5080 2.8740 155.5900

ASP_416@OD1 SER_413@HG SER_413@OG 505 0.5050 2.6516 164.6258

ASP_138@OD1 ARG_390@HH12 ARG_390@NH1 498 0.4980 2.7965 160.3232

GLU_226@O ARG_264@HH22 ARG_264@NH2 497 0.4970 2.8522 148.0118

TRP_396@O ARG_437@H ARG_437@N 496 0.4960 2.8807 156.4709

CYX_134@O ALA_175@H ALA_175@N 492 0.4920 2.8874 158.7018

GLN_408@O GLU_351@H GLU_351@N 488 0.4880 2.8873 155.3627

VAL_407@O VAL_430@H VAL_430@N 488 0.4880 2.8950 160.7426

ILE_242@O GLY_254@H GLY_254@N 487 0.4870 2.8712 151.0891

ASN_189@O ILE_192@H ILE_192@N 486 0.4860 2.8759 155.5338

ALA_64@O VAL_56@H VAL_56@N 481 0.4810 2.8893 161.0779

TYR_88@O THR_108@H THR_108@N 479 0.4790 2.8705 151.4531

ARG_205@O ARG_305@H ARG_305@N 478 0.4780 2.8827 161.6602

THR_279@O THR_282@HG1 THR_282@OG1 476 0.4760 2.7972 157.5807

ASN_171@O THR_137@HG1 THR_137@OG1 474 0.4740 2.7751 159.3008

GLU_329@OE1 THR_337@HG1 THR_337@OG1 473 0.4730 2.7937 157.4577

LEU_207@O HIE_349@HE2 HIE_349@NE2 471 0.4710 2.8702 155.7836

ASP_138@OD1 SER_170@HG SER_170@OG 470 0.4700 2.6289 163.5176

ILE_273@O SER_261@H SER_261@N 469 0.4690 2.8766 153.0809

GLU_70@O THR_71@HG1 THR_71@OG1 457 0.4570 2.6905 160.9548

SER_290@O ASN_227@H ASN_227@N 456 0.4560 2.8976 156.1186

HIE_225@ND1 SER_289@HG SER_289@OG 455 0.4550 2.8264 159.3013

ASP_140@OD1 SER_141@HG SER_141@OG 454 0.4540 2.6809 164.6228

ALA_183@O ASN_186@H ASN_186@N 452 0.4520 2.8798 154.8073

GLN_233@OE1 GLN_37@HE21 GLN_37@NE2 447 0.4470 2.8662 163.1047

ASP_140@OD2 SER_141@HG SER_141@OG 442 0.4420 2.6798 164.6921

TYR_296@O TYR_35@HH TYR_35@OH 438 0.4380 2.7875 161.0979

ARG_127@O GLN_125@HE22 GLN_125@NE2 437 0.4370 2.8639 156.7876

SER_245@OG ARG_264@HH11 ARG_264@NH1 437 0.4370 2.8869 158.7127

PRO_347@O HIE_349@H HIE_349@N 436 0.4360 2.8632 145.0022

VAL_220@O THR_201@H THR_201@N 431 0.4310 2.8959 157.0660

ASP_416@OD2 SER_413@HG SER_413@OG 429 0.4290 2.6559 164.6139

PHE_62@O GLY_58@H GLY_58@N 419 0.4190 2.8634 152.9330

GLU_351@OE1 TYR_410@HH TYR_410@OH 417 0.4170 2.6872 159.7928

VAL_92@O THR_103@H THR_103@N 414 0.4140 2.8996 158.9283

ASP_138@OD2 SER_170@HG SER_170@OG 412 0.4120 2.6369 164.4720

ASP_234@OD1 ARG_240@HH22 ARG_240@NH2 408 0.4080 2.7576 159.4185

MET_284@O GLN_233@H GLN_233@N 405 0.4050 2.8533 150.4745

SER_2@O SER_25@H SER_25@N 405 0.4050 2.8695 158.0584

THR_156@OG1 VAL_174@H VAL_174@N 405 0.4050 2.9039 160.4659

GLU_100@O GLU_94@H GLU_94@N 403 0.4030 2.8955 160.6784

THR_137@O ALA_122@H ALA_122@N 400 0.4000 2.8944 161.2406

GLU_195@OE1 THR_197@HG1 THR_197@OG1 395 0.3950 2.6853 163.7055

ASP_138@OD2 ARG_390@HH12 ARG_390@NH1 395 0.3950 2.8055 162.0446

ASP_84@OD1 HIE_81@H HIE_81@N 393 0.3930 2.8525 158.5411

THR_71@O TYR_24@H TYR_24@N 391 0.3910 2.8896 154.8540

ILE_155@O SER_148@H SER_148@N 391 0.3910 2.8942 156.2953

GLN_41@O GLN_37@HE22 GLN_37@NE2 390 0.3900 2.8460 160.5502

SER_148@OG ILE_155@H ILE_155@N 389 0.3890 2.8845 156.5392

CYX_340@O SER_387@H SER_387@N 389 0.3890 2.8900 155.6719

SER_381@OG GLN_375@H GLN_375@N 389 0.3890 2.8979 153.6306

SER_83@O TYR_38@HH TYR_38@OH 381 0.3810 2.7421 163.3733

LEU_241@O ASP_255@H ASP_255@N 377 0.3770 2.8142 147.5793

CYX_405@O ALA_432@H ALA_432@N 377 0.3770 2.9112 156.2971

TYR_243@O TRP_230@H TRP_230@N 373 0.3730 2.9022 157.8116

ASN_189@OD1 SER_191@H SER_191@N 372 0.3720 2.8832 154.3761

GLU_351@OE1 ARG_205@HH22 ARG_205@NH2 371 0.3710 2.8099 159.3981

THR_108@OG1 GLN_5@HE22 GLN_5@NE2 369 0.3690 2.8668 154.1681

ARG_21@O LEU_6@H LEU_6@N 367 0.3670 2.8978 161.8169

GLU_351@OE2 ARG_205@HH12 ARG_205@NH1 366 0.3660 2.8063 160.4889

LYS_267@O GLN_221@HE22 GLN_221@NE2 361 0.3610 2.8621 149.3048

GLY_238@O ARG_240@HH21 ARG_240@NH2 359 0.3590 2.8100 158.4640

ALA_328@O HIE_332@H HIE_332@N 359 0.3590 2.8841 159.3688

HIE_402@O ASN_357@H ASN_357@N 359 0.3590 2.8942 161.1474

SER_177@O SER_132@H SER_132@N 357 0.3570 2.8862 157.4524

LEU_341@O ALA_321@H ALA_321@N 357 0.3570 2.8912 154.2629

PHE_409@O GLN_428@H GLN_428@N 356 0.3560 2.9011 157.8262

VAL_174@O THR_156@H THR_156@N 354 0.3540 2.9043 160.3981

ASP_247@OD2 VAL_248@H VAL_248@N 353 0.3530 2.8249 146.2237

ASP_416@OD1 SER_413@H SER_413@N 351 0.3510 2.8378 154.8752

VAL_174@O THR_156@HG1 THR_156@OG1 349 0.3490 2.7163 156.9175

GLU_351@OE2 TYR_410@HH TYR_410@OH 345 0.3450 2.6938 160.0544

GLN_334@O ALA_393@H ALA_393@N 345 0.3450 2.8787 160.4849

MET_163@O PHE_168@H PHE_168@N 344 0.3440 2.8984 161.8958

GLU_351@OE2 TYR_206@HH TYR_206@OH 339 0.3390 2.7166 163.0010

ILE_192@O THR_143@HG1 THR_143@OG1 339 0.3390 2.7228 155.7489

ASN_398@OD1 ARG_400@H ARG_400@N 338 0.3380 2.8995 157.3779

SER_13@O ALA_16@H ALA_16@N 337 0.3370 2.9013 158.5594

SER_353@OG GLN_406@HE22 GLN_406@NE2 336 0.3360 2.8837 158.7533

ASP_348@OD2 ARG_305@HH22 ARG_305@NH2 335 0.3350 2.7904 157.3330

ASN_189@O ILE_193@H ILE_193@N 333 0.3330 2.8839 157.4892

LYS_67@O SER_72@H SER_72@N 329 0.3290 2.8985 158.1194

SER_132@O SER_177@H SER_177@N 329 0.3290 2.9010 153.7594

ASP_157@O THR_156@HG1 THR_156@OG1 328 0.3280 2.8009 154.9072

ASP_162@OD1 ARG_164@HE ARG_164@NE 327 0.3270 2.8517 153.0078

GLN_221@OE1 MET_223@H MET_223@N 326 0.3260 2.8846 156.4162

ASP_157@OD1 LYS_158@H LYS_158@N 324 0.3240 2.8060 146.8811

GLU_195@OE1 ASP_196@H ASP_196@N 324 0.3240 2.8467 154.3611

ASP_157@OD2 LYS_158@H LYS_158@N 322 0.3220 2.8115 146.7756

ASP_234@O GLY_238@H GLY_238@N 315 0.3150 2.8752 155.2399

ALA_438@OXT GLN_397@HE22 GLN_397@NE2 314 0.3140 2.8349 161.2070

HIE_349@O TYR_410@H TYR_410@N 314 0.3140 2.8986 162.4341

GLN_406@OE1 ARG_404@HE ARG_404@NE 313 0.3130 2.8365 155.5820

VAL_309@O THR_211@H THR_211@N 312 0.3120 2.8648 146.4123

ASP_348@OD2 ARG_305@HH12 ARG_305@NH1 310 0.3100 2.8291 154.2662

GLY_344@O ARG_382@HE ARG_382@NE 309 0.3090 2.8251 158.3020

ASP_247@OD1 VAL_248@H VAL_248@N 308 0.3080 2.8274 145.5051

GLU_351@OE1 TYR_206@HH TYR_206@OH 307 0.3070 2.6955 163.1630

GLU_218@OE2 ARG_269@HH21 ARG_269@NH2 307 0.3070 2.7999 158.5460

ALA_122@O THR_137@H THR_137@N 306 0.3060 2.9100 157.1764

LEU_306@O SER_283@H SER_283@N 305 0.3050 2.9159 154.1323

ASP_84@OD2 SER_83@HG SER_83@OG 304 0.3040 2.6999 163.2571

VAL_10@O THR_111@HG1 THR_111@OG1 303 0.3030 2.8164 154.2387

GLY_344@O ARG_382@HH11 ARG_382@NH1 301 0.3010 2.8274 162.1405

SER_291@O ASN_99@HD22 ASN_99@ND2 301 0.3010 2.8580 157.5965

LEU_294@O ASN_99@HD22 ASN_99@ND2 297 0.2970 2.8488 160.4695

GLU_351@OE1 ARG_205@HH12 ARG_205@NH1 295 0.2950 2.8014 159.8075

PHE_229@O ALA_288@H ALA_288@N 291 0.2910 2.9059 152.5482

GLU_351@OE2 ARG_205@HH22 ARG_205@NH2 290 0.2900 2.8038 159.2712

PHE_168@O MET_163@H MET_163@N 290 0.2900 2.8864 154.1299

GLY_259@O GLU_275@H GLU_275@N 288 0.2880 2.8781 155.9915

SER_204@O GLN_202@HE21 GLN_202@NE2 287 0.2870 2.8312 152.7010

ASN_60@O LYS_77@HZ2 LYS_77@NZ 284 0.2840 2.8013 153.7316

THR_279@O THR_282@H THR_282@N 283 0.2830 2.9040 154.5731

ARG_164@O ASP_167@H ASP_167@N 280 0.2800 2.8872 153.6786

ASP_138@O ASP_120@H ASP_120@N 279 0.2790 2.8655 159.1900

VAL_10@O THR_111@H THR_111@N 279 0.2790 2.8787 149.7763

LYS_424@O VAL_426@H VAL_426@N 278 0.2780 2.8435 142.7419

ARG_388@O SER_366@H SER_366@N 277 0.2770 2.8886 153.7860

PRO_114@O ASN_171@HD21 ASN_171@ND2 276 0.2760 2.8711 156.7588

ASP_416@OD2 SER_413@H SER_413@N 275 0.2750 2.8456 154.8664

ASP_138@OD2 ARG_390@HH22 ARG_390@NH2 273 0.2730 2.8370 158.7649

VAL_426@O GLN_428@HE22 GLN_428@NE2 272 0.2720 2.8545 163.5668

ASP_84@OD2 HIE_81@H HIE_81@N 272 0.2720 2.8585 159.1011

THR_159@OG1 ARG_388@HH21 ARG_388@NH2 271 0.2710 2.8632 148.3046

GLU_195@OE1 THR_197@H THR_197@N 271 0.2710 2.8691 160.8055

GLU_268@OE1 GLU_268@H GLU_268@N 269 0.2690 2.8388 150.7943

SER_366@OG ARG_388@HE ARG_388@NE 268 0.2680 2.8957 157.6650

SER_95@O GLY_98@H GLY_98@N 268 0.2680 2.9091 155.9628

LEU_312@O VAL_315@H VAL_315@N 265 0.2650 2.9056 160.8476

GLU_252@OE2 LYS_253@H LYS_253@N 264 0.2640 2.8521 153.5976

ASN_186@OD1 ALA_183@H ALA_183@N 264 0.2640 2.8804 157.8036

SER_263@O SER_271@H SER_271@N 262 0.2620 2.9008 159.5032

GLU_218@OE1 ARG_269@HH21 ARG_269@NH2 261 0.2610 2.7954 157.1840

GLU_324@OE2 GLU_324@H GLU_324@N 261 0.2610 2.8440 152.5532

TYR_231@O LEU_286@H LEU_286@N 261 0.2610 2.9135 160.7542

GLU_218@OE1 ARG_269@HE ARG_269@NE 259 0.2590 2.8360 156.4255

ASP_167@OD2 ARG_164@HH12 ARG_164@NH1 258 0.2580 2.7889 155.4316

LEU_126@O ARG_127@HE ARG_127@NE 258 0.2580 2.8024 156.1111

SER_177@O LYS_179@H LYS_179@N 258 0.2580 2.8830 145.6367

ASN_313@O ARG_422@HH22 ARG_422@NH2 257 0.2570 2.8035 149.4373

GLN_221@OE1 HIE_225@H HIE_225@N 256 0.2560 2.9023 161.5534

SER_392@O TRP_396@H TRP_396@N 256 0.2560 2.9087 157.2200

ASP_311@O LYS_314@H LYS_314@N 255 0.2550 2.9063 159.9473

ALA_183@O ALA_187@H ALA_187@N 254 0.2540 2.8637 157.9573

SER_366@O SER_387@HG SER_387@OG 253 0.2530 2.7282 148.4105

ASN_280@O ARG_232@HH22 ARG_232@NH2 253 0.2530 2.8615 150.3852

GLU_324@OE1 GLU_324@H GLU_324@N 252 0.2520 2.8357 152.4972

GLU_70@OE2 SER_69@HG SER_69@OG 250 0.2500 2.6397 165.1737

PHE_409@O THR_427@HG1 THR_427@OG1 248 0.2480 2.7063 158.7080

ASP_138@OD1 ARG_390@HH22 ARG_390@NH2 248 0.2480 2.8347 153.9724

GLN_5@OE1 CYX_90@H CYX_90@N 248 0.2480 2.9017 160.4035

MET_163@O MET_166@H MET_166@N 247 0.2470 2.8929 154.9946

ASP_348@O VAL_350@H VAL_350@N 246 0.2460 2.8768 144.4227

SER_283@O LEU_306@H LEU_306@N 246 0.2460 2.9031 156.8102

ALA_52@O TYR_49@HH TYR_49@OH 245 0.2450 2.7867 164.9753

ASP_348@OD1 ARG_305@HH12 ARG_305@NH1 244 0.2440 2.8280 153.9939

VAL_356@O LYS_359@H LYS_359@N 244 0.2440 2.9069 158.8272

GLU_213@O ALA_277@H ALA_277@N 243 0.2430 2.8624 163.1437

GLN_5@OE1 GLY_107@H GLY_107@N 243 0.2430 2.8842 145.6509

GLU_252@OE1 LYS_253@H LYS_253@N 242 0.2420 2.8671 153.9367

LYS_68@O THR_71@H THR_71@N 242 0.2420 2.8996 153.7465

GLU_327@OE2 ARG_437@HH12 ARG_437@NH1 241 0.2410 2.7911 160.0655

CYX_22@O PHE_73@H PHE_73@N 241 0.2410 2.8921 156.8890

ASN_60@O LYS_77@HZ1 LYS_77@NZ 240 0.2400 2.8095 152.8994

TYR_49@O LEU_32@H LEU_32@N 240 0.2400 2.9103 162.3656

ASP_162@OD2 ARG_164@HH21 ARG_164@NH2 239 0.2390 2.7814 158.2203

GLU_70@OE1 SER_69@HG SER_69@OG 238 0.2380 2.6472 164.7863

THR_307@O LYS_209@H LYS_209@N 238 0.2380 2.8995 157.9411

GLU_310@OE2 THR_211@HG1 THR_211@OG1 231 0.2310 2.7001 164.6578

ASP_348@OD1 ARG_305@HH22 ARG_305@NH2 231 0.2310 2.8000 158.4356

TRP_354@O VAL_361@H VAL_361@N 230 0.2300 2.8906 157.8947

GLN_375@O LEU_378@H LEU_378@N 225 0.2250 2.9057 160.5046

GLY_297@O SER_290@HG SER_290@OG 224 0.2240 2.7632 159.0821

SER_392@O PHE_395@H PHE_395@N 224 0.2240 2.8972 149.1971

GLU_226@OE2 LYS_267@HZ3 LYS_267@NZ 221 0.2210 2.7746 160.5960

GLU_226@OE2 LYS_267@HZ2 LYS_267@NZ 221 0.2210 2.7817 159.3172

GLU_327@OE1 ARG_437@HH22 ARG_437@NH2 221 0.2210 2.7964 160.1506

VAL_315@O ARG_422@HH22 ARG_422@NH2 220 0.2200 2.8641 149.2971

GLU_226@OE2 LYS_267@HZ1 LYS_267@NZ 218 0.2180 2.7848 160.9225

ASP_84@OD1 LYS_77@HZ2 LYS_77@NZ 218 0.2180 2.7963 155.8193

ILE_155@O SER_148@HG SER_148@OG 215 0.2150 2.7417 158.6495

GLU_327@OE1 ARG_437@HH12 ARG_437@NH1 215 0.2150 2.7844 158.1611

GLU_310@OE1 THR_211@HG1 THR_211@OG1 214 0.2140 2.7029 165.7097

ILE_330@O GLN_334@H GLN_334@N 214 0.2140 2.8819 147.3781

ASP_84@OD2 LYS_77@HZ3 LYS_77@NZ 213 0.2130 2.8020 154.2627

THR_111@O VAL_12@H VAL_12@N 210 0.2100 2.9074 155.9812

ASP_84@OD1 LYS_77@HZ3 LYS_77@NZ 209 0.2090 2.8096 153.4481

ASN_60@O LYS_77@HZ3 LYS_77@NZ 209 0.2090 2.8164 153.2131

HIE_74@O GLU_65@H GLU_65@N 209 0.2090 2.8824 158.4228

GLU_268@OE2 GLU_268@H GLU_268@N 208 0.2080 2.8535 152.3094

GLU_213@O VAL_215@H VAL_215@N 208 0.2080 2.8914 144.1109

GLU_327@OE2 ARG_437@HH22 ARG_437@NH2 207 0.2070 2.8004 159.8197

PHE_97@O GLY_297@H GLY_297@N 207 0.2070 2.8520 149.7077

ALA_438@O GLN_397@HE22 GLN_397@NE2 206 0.2060 2.8276 161.1150

GLY_259@O GLN_281@HE21 GLN_281@NE2 206 0.2060 2.8679 154.9848

ASP_84@OD1 LYS_77@HZ1 LYS_77@NZ 202 0.2020 2.8008 156.3716

THR_111@OG1 SER_11@HG SER_11@OG 201 0.2010 2.7969 156.1595

GLN_397@OE1 ARG_437@HE ARG_437@NE 201 0.2010 2.8517 153.6677

GLN_37@O GLU_87@H GLU_87@N 201 0.2010 2.9070 161.1237

ASP_167@OD1 ARG_164@HH22 ARG_164@NH2 200 0.2000 2.8083 151.2098

GLU_226@OE1 GLU_226@H GLU_226@N 200 0.2000 2.8621 146.0543

SER_180@O PHE_182@H PHE_182@N 199 0.1990 2.8508 144.6752

TYR_38@O GLN_41@H GLN_41@N 199 0.1990 2.8977 156.1517

GLU_218@OE2 ARG_269@HE ARG_269@NE 197 0.1970 2.8298 156.2443

ARG_127@O VAL_133@H VAL_133@N 196 0.1960 2.8978 157.2510

ASN_171@O ASP_138@H ASP_138@N 195 0.1950 2.8787 148.7008

GLY_259@O SER_276@H SER_276@N 192 0.1920 2.8876 154.1855

PHE_270@O TRP_230@HE1 TRP_230@NE1 192 0.1920 2.8932 161.6392

SER_95@OG PHE_97@H PHE_97@N 189 0.1890 2.9172 153.4982

ASP_84@OD1 SER_83@HG SER_83@OG 188 0.1880 2.7206 163.0619

ASP_150@OD1 ASP_152@H ASP_152@N 188 0.1880 2.8721 160.2395

HIE_81@ND1 MET_82@H MET_82@N 188 0.1880 2.9040 148.4309

GLU_87@OE1 ARG_109@HE ARG_109@NE 186 0.1860 2.8037 159.8344

ASP_138@OD1 THR_137@HG1 THR_137@OG1 183 0.1830 2.7211 161.5752

GLN_420@OE1 ARG_422@HE ARG_422@NE 183 0.1830 2.8516 148.0192

LEU_412@O THR_427@H THR_427@N 183 0.1830 2.8581 148.8970

ASN_40@O GLY_303@H GLY_303@N 180 0.1800 2.8721 159.8491

GLY_107@O THR_108@HG1 THR_108@OG1 178 0.1780 2.7498 156.7362

THR_299@O THR_299@HG1 THR_299@OG1 178 0.1780 2.8399 141.9591

ASP_222@OD1 LYS_199@H2 LYS_199@N 177 0.1770 2.8116 157.9666

ASN_398@O ASN_401@H ASN_401@N 177 0.1770 2.9119 159.5770

PRO_96@O LYS_48@HZ1 LYS_48@NZ 176 0.1760 2.8359 157.7779

THR_159@OG1 SER_172@HG SER_172@OG 175 0.1750 2.8139 153.6702

SER_170@OG ASN_171@H ASN_171@N 174 0.1740 2.7851 139.1073

TYR_298@OH LEU_102@H LEU_102@N 172 0.1720 2.9195 157.2333

LYS_101@O THR_103@HG1 THR_103@OG1 171 0.1710 2.7897 158.7625

SER_191@O ASN_144@HD22 ASN_144@ND2 171 0.1710 2.8552 158.6767

LEU_241@O ILE_256@H ILE_256@N 171 0.1710 2.9158 160.4748

ASP_234@O LEU_237@H LEU_237@N 169 0.1690 2.9175 153.1488

GLN_406@OE1 ARG_404@HH11 ARG_404@NH1 168 0.1680 2.8260 156.7632

GLU_226@OE2 SER_291@HG SER_291@OG 167 0.1670 2.6382 166.8707

ASP_222@OD1 LYS_199@H3 LYS_199@N 166 0.1660 2.8028 157.4105

ASP_152@OD2 SER_151@HG SER_151@OG 165 0.1650 2.7016 163.0872

GLU_87@OE1 ARG_109@HH21 ARG_109@NH2 165 0.1650 2.8137 154.4682

ALA_342@O SER_384@HG SER_384@OG 164 0.1640 2.7286 160.9758

GLU_87@OE2 ARG_109@HE ARG_109@NE 163 0.1630 2.8126 157.8889

PRO_257@O LYS_253@HZ3 LYS_253@NZ 163 0.1630 2.8190 155.9871

ASP_140@OD1 ILE_116@H ILE_116@N 163 0.1630 2.8691 161.7465

SER_25@O SER_2@H SER_2@N 163 0.1630 2.8747 154.1320

LYS_67@O GLU_70@H GLU_70@N 163 0.1630 2.9105 153.5312

ASP_138@OD2 THR_137@HG1 THR_137@OG1 162 0.1620 2.7509 162.8825

SER_289@O THR_299@HG1 THR_299@OG1 162 0.1620 2.8250 156.0244

SER_271@O SER_263@H SER_263@N 162 0.1620 2.9065 153.4291

HIE_74@ND1 CYX_22@H CYX_22@N 162 0.1620 2.9384 158.5816

ASP_222@OD2 LYS_199@H2 LYS_199@N 161 0.1610 2.8151 156.9730

ASP_311@OD2 ASN_313@HD22 ASN_313@ND2 161 0.1610 2.8242 157.3207

ALA_185@O ASN_189@HD22 ASN_189@ND2 161 0.1610 2.8669 161.9042

ASP_255@OD1 LEU_241@H LEU_241@N 160 0.1600 2.8677 161.7882

ASP_247@O MET_250@H MET_250@N 160 0.1600 2.8898 152.9961

ASP_416@OD2 LYS_209@HZ2 LYS_209@NZ 159 0.1590 2.8102 157.0212

GLU_87@OE2 ARG_109@HH21 ARG_109@NH2 159 0.1590 2.8273 157.5284

GLN_147@OE1 ASP_157@H ASP_157@N 159 0.1590 2.8796 156.7742

GLU_70@O SER_72@HG SER_72@OG 158 0.1580 2.7281 161.0940

ASP_380@OD1 LYS_314@HZ2 LYS_314@NZ 157 0.1570 2.8036 156.3850

ILE_193@O GLU_195@H GLU_195@N 156 0.1560 2.8733 142.6868

SER_93@OG SER_95@H SER_95@N 156 0.1560 2.9230 156.7258

GLU_310@OE2 GLU_310@H GLU_310@N 155 0.1550 2.8306 151.9472

ASP_224@OD1 LYS_267@HZ1 LYS_267@NZ 154 0.1540 2.8086 158.9815

GLY_238@O ASP_234@H ASP_234@N 154 0.1540 2.9078 159.8441

ASP_224@OD1 LYS_267@HZ2 LYS_267@NZ 153 0.1530 2.8058 158.1599

PRO_96@O LYS_48@HZ2 LYS_48@NZ 153 0.1530 2.8264 157.0303

GLN_202@OE1 SER_204@H SER_204@N 153 0.1530 2.8862 154.3111

GLN_281@OE1 SER_278@H SER_278@N 153 0.1530 2.8868 156.3825

ASP_416@OD2 LYS_209@HZ1 LYS_209@NZ 152 0.1520 2.8055 159.4855

ASP_222@OD1 LYS_199@H1 LYS_199@N 149 0.1490 2.8004 156.0570

GLU_324@OE2 ARG_127@HH22 ARG_127@NH2 149 0.1490 2.8029 156.3676

ASP_84@OD2 LYS_77@HZ2 LYS_77@NZ 149 0.1490 2.8155 152.4468

PRO_96@O LYS_48@HZ3 LYS_48@NZ 149 0.1490 2.8261 157.3017

ASP_255@OD2 LEU_241@H LEU_241@N 149 0.1490 2.8593 163.4529

GLU_258@OE2 GLY_259@H GLY_259@N 147 0.1470 2.8559 156.0050

ASP_380@OD2 LYS_314@HZ3 LYS_314@NZ 146 0.1460 2.8171 156.3725

ASP_234@OD1 ARG_240@HH12 ARG_240@NH1 146 0.1460 2.8264 147.7776

GLU_213@OE2 LYS_214@H LYS_214@N 146 0.1460 2.8548 151.4642

ASP_311@OD2 ASN_313@H ASN_313@N 145 0.1450 2.8933 160.9889

LYS_179@O SER_132@HG SER_132@OG 144 0.1440 2.7482 160.7418

ASP_380@OD2 LYS_314@HZ2 LYS_314@NZ 143 0.1430 2.8320 155.3785

ASP_150@OD2 ASP_152@H ASP_152@N 142 0.1420 2.8730 160.0496

ASP_84@OD2 LYS_77@HZ1 LYS_77@NZ 141 0.1410 2.7952 153.5120

PRO_257@O LYS_253@HZ1 LYS_253@NZ 141 0.1410 2.8243 155.1989

GLU_258@OE1 GLY_259@H GLY_259@N 141 0.1410 2.8654 155.4714

ASP_416@O LYS_424@HZ1 LYS_424@NZ 140 0.1400 2.7977 149.8526

ASP_224@OD2 LYS_267@HZ3 LYS_267@NZ 140 0.1400 2.8070 159.3829

ASP_224@OD2 LYS_267@HZ2 LYS_267@NZ 140 0.1400 2.8076 158.5685

PRO_371@O GLN_147@HE22 GLN_147@NE2 139 0.1390 2.8690 153.4820

ASP_311@OD1 ASN_313@H ASN_313@N 139 0.1390 2.8940 161.5002

ASP_224@OD1 LYS_267@HZ3 LYS_267@NZ 138 0.1380 2.8029 157.4136

ASP_380@OD1 LYS_314@HZ3 LYS_314@NZ 138 0.1380 2.8222 155.3149

ASP_311@OD1 ASN_313@HD22 ASN_313@ND2 138 0.1380 2.8347 155.9096

ASP_416@O LYS_424@HZ2 LYS_424@NZ 137 0.1370 2.7942 150.2270

PRO_371@O GLN_147@HE21 GLN_147@NE2 136 0.1360 2.8798 155.4758

TYR_285@O GLN_202@HE21 GLN_202@NE2 136 0.1360 2.8905 150.1460

SER_326@O GLU_329@H GLU_329@N 136 0.1360 2.9000 149.4231

ASP_222@OD2 LYS_199@H1 LYS_199@N 135 0.1350 2.8140 156.4080

SER_128@O SER_180@HG SER_180@OG 134 0.1340 2.7047 165.2770

ASP_222@OD2 LYS_199@H3 LYS_199@N 134 0.1340 2.8168 159.2513

GLN_125@OE1 CYX_184@H CYX_184@N 133 0.1330 2.8646 153.3098

GLN_202@O SER_204@H SER_204@N 132 0.1320 2.8914 143.6437

GLU_213@OE1 LYS_214@H LYS_214@N 131 0.1310 2.8538 153.9096

ASP_150@OD1 VAL_153@H VAL_153@N 130 0.1300 2.8542 160.4004

SER_386@O ASP_368@H ASP_368@N 130 0.1300 2.9299 163.0749

GLU_265@OE2 SER_263@HG SER_263@OG 129 0.1290 2.6596 163.4321

GLU_374@OE1 TYR_154@HH TYR_154@OH 129 0.1290 2.6664 164.9284

ASN_280@OD1 SER_278@HG SER_278@OG 129 0.1290 2.7000 161.2488

LEU_217@O SER_271@HG SER_271@OG 129 0.1290 2.7411 160.0800

ASP_416@OD1 LYS_209@HZ2 LYS_209@NZ 129 0.1290 2.8000 159.7157

ASP_416@O LYS_424@HZ3 LYS_424@NZ 129 0.1290 2.8133 149.7902

GLN_420@O ARG_422@H ARG_422@N 129 0.1290 2.8516 146.3280

PRO_257@O LYS_253@HZ2 LYS_253@NZ 128 0.1280 2.8188 155.8389

PHE_182@O GLN_125@HE21 GLN_125@NE2 127 0.1270 2.8797 154.3154

ASP_224@OD2 LYS_267@HZ1 LYS_267@NZ 125 0.1250 2.8033 158.2647

CYX_184@O ALA_187@H ALA_187@N 125 0.1250 2.9124 155.9967

PRO_121@O THR_197@HG1 THR_197@OG1 123 0.1230 2.7479 162.8392

ASP_130@OD1 LYS_131@H LYS_131@N 122 0.1220 2.8383 150.6894

GLU_360@OE1 GLU_360@H GLU_360@N 122 0.1220 2.8464 149.1595

GLN_406@OE1 ARG_404@HH21 ARG_404@NH2 122 0.1220 2.8557 146.7273

GLU_324@OE1 ARG_127@HH22 ARG_127@NH2 121 0.1210 2.8058 156.1867

SER_129@O LYS_131@H LYS_131@N 121 0.1210 2.8121 148.2614

ASP_150@OD1 SER_151@H SER_151@N 121 0.1210 2.8161 146.3901

ASN_40@OD1 ARG_205@HH21 ARG_205@NH2 121 0.1210 2.8257 156.8887

GLU_310@OE1 GLU_310@H GLU_310@N 120 0.1200 2.8228 148.4536

GLU_14@O ASN_115@HD21 ASN_115@ND2 120 0.1200 2.8414 154.6893

HIE_349@ND1 ARG_305@HH21 ARG_305@NH2 118 0.1180 2.9068 150.2714

GLU_433@OE2 HIE_402@HE2 HIE_402@NE2 115 0.1150 2.8199 157.7081

ASP_150@OD2 SER_151@H SER_151@N 114 0.1140 2.8281 147.0244

SER_165@OG SER_363@HG SER_363@OG 113 0.1130 2.7701 159.2871

ASN_23@OD1 THR_4@HG1 THR_4@OG1 112 0.1120 2.7503 151.3333

GLU_360@OE2 GLU_360@H GLU_360@N 112 0.1120 2.8355 149.8200

ASP_152@OD1 SER_151@HG SER_151@OG 111 0.1110 2.6930 162.1069

ASN_40@OD1 SER_302@HG SER_302@OG 111 0.1110 2.7388 158.6862

ASP_130@O SER_132@HG SER_132@OG 111 0.1110 2.7869 162.8347

ASP_167@OD2 ARG_164@HH22 ARG_164@NH2 111 0.1110 2.8030 151.2597

ARG_400@O ARG_400@HH11 ARG_400@NH1 111 0.1110 2.8413 164.7987

GLU_417@OE2 TRP_418@H TRP_418@N 111 0.1110 2.8831 153.4405

GLU_87@OE1 ARG_109@HH11 ARG_109@NH1 108 0.1080 2.7790 159.4665

ASP_380@OD2 LYS_314@HZ1 LYS_314@NZ 108 0.1080 2.8036 156.5321

GLY_259@O GLN_281@HE22 GLN_281@NE2 108 0.1080 2.8407 154.7722

ASN_40@OD1 ARG_305@HH11 ARG_305@NH1 107 0.1070 2.8122 155.7138

TRP_435@O ARG_437@HH11 ARG_437@NH1 107 0.1070 2.8231 155.2479

GLN_375@OE1 ALA_377@H ALA_377@N 107 0.1070 2.8706 157.0589

GLU_433@OE1 HIE_402@HE2 HIE_402@NE2 106 0.1060 2.8095 158.4091

TYR_383@O GLY_344@H GLY_344@N 106 0.1060 2.8379 145.0005

GLN_397@OE1 ARG_437@HH21 ARG_437@NH2 105 0.1050 2.8514 148.1161

GLU_324@OE1 ARG_127@HH21 ARG_127@NH2 104 0.1040 2.7875 160.1593

ASP_416@OD1 LYS_209@HZ1 LYS_209@NZ 104 0.1040 2.8127 157.8494

GLU_14@OE2 LYS_169@HZ2 LYS_169@NZ 103 0.1030 2.7963 157.6910

SER_392@OG THR_394@HG1 THR_394@OG1 103 0.1030 2.8035 158.2599

ASP_416@OD1 LYS_209@HZ3 LYS_209@NZ 103 0.1030 2.8056 159.4585

GLU_324@OE2 ARG_127@HH21 ARG_127@NH2 102 0.1020 2.7736 158.7745

GLU_14@OE2 LYS_169@HZ3 LYS_169@NZ 102 0.1020 2.7994 157.4617

GLU_213@OE2 ARG_210@HH21 ARG_210@NH2 102 0.1020 2.8046 155.8861

GLU_218@OE1 ARG_269@HH11 ARG_269@NH1 102 0.1020 2.8073 159.9941

GLU_65@OE2 THR_54@HG1 THR_54@OG1 101 0.1010 2.6314 162.5982

GLU_275@OE2 LYS_214@HZ1 LYS_214@NZ 101 0.1010 2.7994 157.7408

ASP_368@OD1 ARG_388@HH12 ARG_388@NH1 101 0.1010 2.8155 150.3884

ASP_234@OD1 ARG_240@HE ARG_240@NE 101 0.1010 2.8414 145.9703

ASP_152@OD1 ASN_178@HD21 ASN_178@ND2 101 0.1010 2.8490 160.8901

SER_413@OG ASN_415@H ASN_415@N 101 0.1010 2.9088 148.3327

GLU_265@OE1 SER_263@HG SER_263@OG 100 0.1000 2.6566 164.6188

GLU_275@OE1 LYS_214@HZ1 LYS_214@NZ 100 0.1000 2.7840 157.8681

TYR_38@OH ASN_60@HD22 ASN_60@ND2 100 0.1000 2.8744 154.7215

SER_132@OG GLN_125@HE22 GLN_125@NE2 100 0.1000 2.8926 150.1528

GLU_275@OE2 LYS_214@HZ2 LYS_214@NZ 99 0.0990 2.7865 158.4460

GLU_319@OE2 VAL_320@H VAL_320@N 99 0.0990 2.8793 153.0572

ASP_380@OD1 LYS_314@HZ1 LYS_314@NZ 98 0.0980 2.8107 153.9109

LEU_294@O TYR_296@H TYR_296@N 98 0.0980 2.8541 143.1406

ASP_416@OD2 LYS_209@HZ3 LYS_209@NZ 97 0.0970 2.8056 157.5421

ASP_150@OD2 VAL_153@H VAL_153@N 97 0.0970 2.8618 161.3577

HIE_362@O VAL_365@H VAL_365@N 97 0.0970 2.9138 157.2190

ASP_140@OD2 ILE_116@H ILE_116@N 96 0.0960 2.8711 160.3286

GLY_15@O SER_79@HG SER_79@OG 95 0.0950 2.7904 159.1962

ASP_138@OD2 LYS_335@HZ1 LYS_335@NZ 95 0.0950 2.7947 157.9050

PRO_121@O THR_197@H THR_197@N 95 0.0950 2.8336 155.7700

GLU_14@O ASN_115@HD22 ASN_115@ND2 95 0.0950 2.8507 159.3409

SER_276@O GLN_281@HE22 GLN_281@NE2 94 0.0940 2.8572 156.0388

VAL_248@O MET_250@H MET_250@N 94 0.0940 2.8683 148.4600

SER_326@O ILE_330@H ILE_330@N 94 0.0940 2.9249 166.0184

GLU_329@OE2 LYS_335@HZ3 LYS_335@NZ 93 0.0930 2.7866 160.3036

GLU_213@OE1 ARG_210@HH21 ARG_210@NH2 93 0.0930 2.7958 156.6881

ASP_130@OD2 LYS_131@H LYS_131@N 93 0.0930 2.8455 149.5878

GLU_14@OE2 ASN_115@H ASN_115@N 93 0.0930 2.8671 164.2105

GLU_275@OE2 LYS_214@HZ3 LYS_214@NZ 92 0.0920 2.7912 156.7353

ILE_59@O PHE_62@H PHE_62@N 92 0.0920 2.9362 163.5345

GLU_14@OE2 LYS_169@HZ1 LYS_169@NZ 91 0.0910 2.7889 158.3221

ASN_23@OD1 THR_71@HG1 THR_71@OG1 90 0.0900 2.7669 151.2005

GLU_324@OE1 ARG_127@HH12 ARG_127@NH1 90 0.0900 2.8126 155.0052

GLU_100@OE1 LYS_101@H LYS_101@N 90 0.0900 2.8654 149.4207

VAL_133@O ARG_127@H ARG_127@N 89 0.0890 2.9257 157.9039

GLU_275@OE1 LYS_214@HZ3 LYS_214@NZ 88 0.0880 2.7856 156.5099

HIE_81@ND1 SER_83@HG SER_83@OG 88 0.0880 2.8121 165.0839

PHE_104@O GLN_1@HE22 GLN_1@NE2 88 0.0880 2.8513 156.5278

GLU_319@OE1 VAL_320@H VAL_320@N 88 0.0880 2.8692 152.5407

THR_394@OG1 SER_392@HG SER_392@OG 87 0.0870 2.7846 152.2523

GLU_65@OE1 THR_54@HG1 THR_54@OG1 86 0.0860 2.6755 163.6255

GLU_329@OE2 LYS_335@HZ1 LYS_335@NZ 86 0.0860 2.7865 158.9298

GLU_324@OE2 ARG_127@HH12 ARG_127@NH1 86 0.0860 2.8186 156.3444

SER_283@OG MET_284@H MET_284@N 86 0.0860 2.8345 138.7490

ASP_138@OD2 LYS_335@HZ2 LYS_335@NZ 85 0.0850 2.7990 158.9854

LEU_237@O ARG_240@HE ARG_240@NE 84 0.0840 2.8603 152.3279

ASP_138@OD2 LYS_335@HZ3 LYS_335@NZ 83 0.0830 2.7959 157.6105

ASP_84@OD2 ASN_60@HD21 ASN_60@ND2 83 0.0830 2.8081 156.4436

ASP_181@O ASN_186@HD21 ASN_186@ND2 83 0.0830 2.8668 159.1233

SER_363@OG SER_165@H SER_165@N 83 0.0830 2.9080 156.0855

ASP_162@OD1 MET_163@H MET_163@N 82 0.0820 2.7880 141.6617

VAL_145@O LYS_158@HZ1 LYS_158@NZ 82 0.0820 2.8310 151.1964

PHE_323@O VAL_339@H VAL_339@N 82 0.0820 2.9071 162.1248

VAL_18@O LYS_77@H LYS_77@N 82 0.0820 2.9248 154.2914

THR_333@O LYS_335@HZ1 LYS_335@NZ 81 0.0810 2.8224 155.6835

GLY_98@O GLU_100@H GLU_100@N 81 0.0810 2.8634 144.1011

PRO_39@O GLN_41@HE22 GLN_41@NE2 81 0.0810 2.8703 160.9458

GLU_417@OE1 TRP_418@H TRP_418@N 81 0.0810 2.8740 151.5775

SER_291@O LEU_294@H LEU_294@N 81 0.0810 2.8948 158.1329

TYR_31@O SER_93@H SER_93@N 81 0.0810 2.9071 154.3543

GLU_433@OE2 ARG_404@HH21 ARG_404@NH2 80 0.0800 2.7921 156.0856

GLU_433@OE1 ARG_404@HH21 ARG_404@NH2 80 0.0800 2.8281 157.6931

VAL_3@O GLN_1@HE22 GLN_1@NE2 80 0.0800 2.8517 158.2555

THR_333@OG1 LYS_335@HZ2 LYS_335@NZ 80 0.0800 2.8717 159.9462

GLU_329@OE2 LYS_335@HZ2 LYS_335@NZ 79 0.0790 2.7750 160.1745

GLU_70@O ASN_23@HD21 ASN_23@ND2 79 0.0790 2.8693 156.7177

GLU_374@OE2 TYR_154@HH TYR_154@OH 78 0.0780 2.6738 163.2671

GLU_310@OE2 LYS_314@HZ2 LYS_314@NZ 78 0.0780 2.8003 157.9129

ASP_130@OD2 SER_129@HG SER_129@OG 77 0.0770 2.6790 164.0405

SER_172@O THR_159@HG1 THR_159@OG1 77 0.0770 2.7967 154.6567

GLU_14@O SER_13@HG SER_13@OG 76 0.0760 2.7821 164.7662

GLU_329@OE2 ARG_390@HE ARG_390@NE 76 0.0760 2.8562 147.0265

GLU_265@OE2 GLU_265@H GLU_265@N 76 0.0760 2.8590 153.0270

SER_386@OG ARG_388@HH11 ARG_388@NH1 76 0.0760 2.8707 146.8019

SER_366@OG ARG_388@HH21 ARG_388@NH2 76 0.0760 2.8940 152.5410

SER_326@OG ALA_328@H ALA_328@N 76 0.0760 2.9401 152.3423

LEU_412@O THR_427@HG1 THR_427@OG1 75 0.0750 2.8295 162.7812

PRO_399@O TRP_435@HE1 TRP_435@NE1 75 0.0750 2.8463 153.1242

SER_180@OG ASP_130@H ASP_130@N 75 0.0750 2.8885 155.5113

ASP_130@OD1 ARG_127@HH12 ARG_127@NH1 74 0.0740 2.7709 160.2299

THR_137@OG1 ARG_390@HH22 ARG_390@NH2 74 0.0740 2.8805 150.0938

SER_263@O SER_271@HG SER_271@OG 73 0.0730 2.8004 147.3731

LYS_131@O SER_129@H SER_129@N 73 0.0730 2.8923 154.7410

PHE_136@O SER_172@HG SER_172@OG 72 0.0720 2.8120 154.5867

THR_333@OG1 LYS_335@H LYS_335@N 72 0.0720 2.9035 154.0197

GLU_14@OE1 LYS_169@HZ2 LYS_169@NZ 71 0.0710 2.7869 159.3479

GLU_275@OE1 LYS_214@HZ2 LYS_214@NZ 71 0.0710 2.7956 158.4846

SER_129@O SER_180@HG SER_180@OG 69 0.0690 2.6969 163.1352

ASP_130@OD1 SER_129@HG SER_129@OG 69 0.0690 2.6979 163.8267

THR_111@O SER_11@HG SER_11@OG 69 0.0690 2.7830 157.7581

ASP_348@OD2 ASP_348@H ASP_348@N 69 0.0690 2.8050 138.7457

GLU_310@OE1 LYS_314@HZ1 LYS_314@NZ 69 0.0690 2.8088 156.7795

GLU_70@OE1 LYS_67@HZ2 LYS_67@NZ 69 0.0690 2.8237 156.1548

LEU_161@O SER_170@H SER_170@N 69 0.0690 2.8639 149.9928

THR_333@OG1 LYS_335@HZ3 LYS_335@NZ 69 0.0690 2.8681 155.8678

SER_353@OG TRP_355@HE1 TRP_355@NE1 69 0.0690 2.8878 151.9811

ASN_99@O SER_290@HG SER_290@OG 68 0.0680 2.7834 153.9477

VAL_145@O LYS_158@HZ2 LYS_158@NZ 68 0.0680 2.8047 150.5939

GLU_414@OE2 LYS_424@HZ3 LYS_424@NZ 68 0.0680 2.8058 160.9691

THR_50@OG1 SER_51@HG SER_51@OG 68 0.0680 2.8275 165.1292

SER_413@O ASP_416@H ASP_416@N 68 0.0680 2.9160 155.0214

GLU_265@OE1 LYS_249@HZ1 LYS_249@NZ 67 0.0670 2.8070 157.0243

GLU_265@OE1 LYS_249@HZ3 LYS_249@NZ 67 0.0670 2.8082 154.3570

GLU_265@OE2 LYS_249@HZ3 LYS_249@NZ 67 0.0670 2.8164 155.2051

ASN_186@O ASN_189@HD22 ASN_189@ND2 67 0.0670 2.8558 161.1245

LYS_266@O ARG_264@HE ARG_264@NE 67 0.0670 2.9033 145.0223

GLU_433@OE2 SER_431@HG SER_431@OG 66 0.0660 2.6700 163.0019

SER_413@O LYS_424@HZ3 LYS_424@NZ 66 0.0660 2.8150 152.9058

GLU_100@OE2 LYS_101@H LYS_101@N 66 0.0660 2.8695 150.1312

LYS_179@O SER_132@H SER_132@N 66 0.0660 2.8773 158.6516

THR_103@OG1 GLN_1@HE21 GLN_1@NE2 65 0.0650 2.8616 160.9445

THR_71@O ASN_23@HD22 ASN_23@ND2 63 0.0630 2.8693 155.3050

MET_163@O ASP_167@H ASP_167@N 63 0.0630 2.8720 148.4928

TRP_354@O GLN_406@HE21 GLN_406@NE2 63 0.0630 2.8999 154.5832

ALA_438@O ARG_437@HH11 ARG_437@NH1 62 0.0620 2.7840 161.0851

GLN_281@OE1 SER_278@HG SER_278@OG 61 0.0610 2.7186 159.2981

GLU_226@OE1 LYS_267@HZ3 LYS_267@NZ 61 0.0610 2.7967 159.3600

LYS_199@O ASP_222@H ASP_222@N 61 0.0610 2.9111 157.2975

GLU_63@OE1 LYS_57@HZ1 LYS_57@NZ 60 0.0600 2.7976 155.6749

GLU_70@OE2 LYS_67@HZ3 LYS_67@NZ 60 0.0600 2.8097 156.6092

GLU_70@OE2 LYS_67@HZ2 LYS_67@NZ 60 0.0600 2.8205 156.2933

SER_413@O LYS_424@HZ1 LYS_424@NZ 60 0.0600 2.8275 151.2135

ASP_157@OD2 LYS_158@HZ2 LYS_158@NZ 59 0.0590 2.7971 156.5029

ASP_196@O PHE_198@H PHE_198@N 59 0.0590 2.8490 144.6179

ASP_162@O GLY_364@H GLY_364@N 59 0.0590 2.8906 146.1730

GLN_44@OE1 TYR_38@HH TYR_38@OH 58 0.0580 2.7422 165.1918

GLU_63@OE1 LYS_57@HZ3 LYS_57@NZ 58 0.0580 2.7861 158.4742

GLU_14@OE1 LYS_169@HZ3 LYS_169@NZ 58 0.0580 2.7948 158.4640

THR_71@O THR_71@HG1 THR_71@OG1 58 0.0580 2.7987 140.7602

SER_276@O GLN_281@HE21 GLN_281@NE2 58 0.0580 2.8551 152.1477

GLN_125@O SER_326@HG SER_326@OG 57 0.0570 2.7762 155.0633

SER_290@O ASN_227@HD22 ASN_227@ND2 57 0.0570 2.8675 156.4256

ASN_313@OD1 GLN_420@HE22 GLN_420@NE2 57 0.0570 2.8678 161.6640

GLY_303@O ARG_205@HH11 ARG_205@NH1 57 0.0570 2.8706 154.6488

GLN_1@O SER_25@HG SER_25@OG 56 0.0560 2.7072 160.1105

GLU_414@OE2 LYS_424@HZ2 LYS_424@NZ 56 0.0560 2.7956 159.3877

GLU_265@OE2 LYS_249@HZ1 LYS_249@NZ 56 0.0560 2.7962 156.1473

GLU_213@OE2 ARG_210@HE ARG_210@NE 56 0.0560 2.8483 156.2272

LEU_274@O SER_276@H SER_276@N 56 0.0560 2.8597 145.4495

GLN_117@OE1 LYS_169@H LYS_169@N 56 0.0560 2.8935 161.4435

SER_172@OG ARG_388@HE ARG_388@NE 56 0.0560 2.9042 150.2532

HIE_81@ND1 SER_83@H SER_83@N 56 0.0560 2.9193 163.5592

GLU_310@OE1 LYS_314@HZ2 LYS_314@NZ 55 0.0550 2.7816 158.4633

GLU_324@OE1 ARG_437@HH22 ARG_437@NH2 55 0.0550 2.8116 156.1000

GLU_310@OE2 LYS_314@HZ3 LYS_314@NZ 55 0.0550 2.8213 157.9363

THR_333@O LYS_335@HZ3 LYS_335@NZ 55 0.0550 2.8240 155.5307

ASP_120@O ASN_118@HD22 ASN_118@ND2 55 0.0550 2.8270 153.9509

SER_413@O LYS_424@HZ2 LYS_424@NZ 55 0.0550 2.8325 153.6108

GLU_213@OE1 ARG_210@HE ARG_210@NE 55 0.0550 2.8413 156.5566

GLU_414@OE2 LYS_424@HZ1 LYS_424@NZ 54 0.0540 2.7853 160.6463

GLU_63@OE2 LYS_57@HZ3 LYS_57@NZ 54 0.0540 2.7871 157.9275

GLU_70@OE1 LYS_67@HZ3 LYS_67@NZ 54 0.0540 2.7880 157.3498

GLU_218@OE2 ARG_269@HH11 ARG_269@NH1 54 0.0540 2.7943 157.5492

GLU_226@OE1 LYS_267@HZ1 LYS_267@NZ 54 0.0540 2.8024 160.6400

GLU_63@OE2 LYS_57@HZ1 LYS_57@NZ 54 0.0540 2.8059 157.1398

ASP_130@OD2 ARG_127@HH22 ARG_127@NH2 54 0.0540 2.8632 155.3249

ASP_234@OD2 ARG_232@HE ARG_232@NE 54 0.0540 2.8706 149.0163

SER_392@OG THR_394@H THR_394@N 54 0.0540 2.9307 153.8119

GLU_310@OE2 LYS_314@HZ1 LYS_314@NZ 53 0.0530 2.8062 157.4288

LYS_131@O ARG_127@HH11 ARG_127@NH1 53 0.0530 2.8469 153.8549

GLN_41@OE1 GLY_42@H GLY_42@N 53 0.0530 2.8653 154.1279

GLU_195@OE2 ASP_196@H ASP_196@N 53 0.0530 2.8762 151.0278

GLN_1@OE1 SER_26@HG SER_26@OG 52 0.0520 2.7234 160.7366

GLU_70@OE2 LYS_67@HZ1 LYS_67@NZ 52 0.0520 2.7961 154.0635

ARG_269@O ARG_264@HE ARG_264@NE 52 0.0520 2.8872 142.3089

GLN_233@O MET_284@H MET_284@N 52 0.0520 2.9321 154.8246

GLU_414@OE1 LYS_424@HZ3 LYS_424@NZ 51 0.0510 2.7902 159.5577

GLU_14@OE1 LYS_169@HZ1 LYS_169@NZ 51 0.0510 2.7987 156.7282

GLU_265@OE2 LYS_249@HZ2 LYS_249@NZ 51 0.0510 2.8020 153.0089

VAL_160@O SER_172@HG SER_172@OG 51 0.0510 2.8510 149.5376

SER_366@O ARG_388@HH12 ARG_388@NH1 51 0.0510 2.8518 158.4204

GLN_147@OE1 GLN_370@HE22 GLN_370@NE2 51 0.0510 2.8527 161.3258

SER_172@OG SER_366@HG SER_366@OG 51 0.0510 2.8543 150.8060

THR_292@O ASN_99@HD21 ASN_99@ND2 51 0.0510 2.8806 148.5863

GLU_218@O SER_203@H SER_203@N 51 0.0510 2.8940 151.7457

GLU_414@OE1 LYS_424@HZ1 LYS_424@NZ 50 0.0500 2.7996 159.2299

ASP_196@OD2 ASN_190@HD22 ASN_190@ND2 50 0.0500 2.8155 160.0993

GLU_265@OE1 LYS_249@HZ2 LYS_249@NZ 49 0.0490 2.7844 156.6343

GLU_226@OE1 LYS_267@HZ2 LYS_267@NZ 49 0.0490 2.7849 156.1616

GLU_324@OE2 ARG_437@HH22 ARG_437@NH2 49 0.0490 2.8030 160.2107

THR_333@O LYS_335@HZ2 LYS_335@NZ 49 0.0490 2.8401 153.5814

THR_211@O ARG_210@HH11 ARG_210@NH1 49 0.0490 2.8528 154.8473

GLY_344@O ARG_382@HH21 ARG_382@NH2 49 0.0490 2.8677 144.3600

THR_333@OG1 LYS_335@HZ1 LYS_335@NZ 49 0.0490 2.8790 159.0985

GLU_275@OE2 SER_261@HG SER_261@OG 48 0.0480 2.6526 163.0063

ASP_157@OD2 LYS_158@HZ1 LYS_158@NZ 48 0.0480 2.7763 158.6555

GLU_70@OE1 LYS_67@HZ1 LYS_67@NZ 48 0.0480 2.8169 155.6756

ASP_152@OD2 ASN_178@HD21 ASN_178@ND2 48 0.0480 2.8413 162.7937

GLY_238@O ARG_240@HE ARG_240@NE 48 0.0480 2.8704 148.6522

ASN_23@OD1 SER_72@HG SER_72@OG 47 0.0470 2.7302 163.4708

ASP_130@OD2 ARG_127@HH12 ARG_127@NH1 47 0.0470 2.7973 156.5830

ASP_196@OD1 ASN_190@HD22 ASN_190@ND2 47 0.0470 2.8140 162.7263

ASN_40@OD1 ARG_305@HE ARG_305@NE 47 0.0470 2.8788 148.4489

ASP_157@OD1 LYS_158@HZ1 LYS_158@NZ 46 0.0460 2.7828 156.7615

VAL_145@O LYS_158@HZ3 LYS_158@NZ 46 0.0460 2.7960 150.7075

ASP_152@OD2 VAL_153@H VAL_153@N 46 0.0460 2.8229 146.5024

GLU_417@O ARG_210@HH22 ARG_210@NH2 46 0.0460 2.8340 154.7148

ASP_157@OD2 GLN_370@HE22 GLN_370@NE2 46 0.0460 2.8447 165.8007

GLU_433@OE2 ARG_404@HE ARG_404@NE 46 0.0460 2.8607 152.7572

GLU_195@OE2 THR_197@HG1 THR_197@OG1 45 0.0450 2.6844 161.4248

ASN_40@O SER_302@HG SER_302@OG 45 0.0450 2.7076 160.2810

GLU_63@OE1 LYS_57@HZ2 LYS_57@NZ 45 0.0450 2.7990 158.4382

GLU_275@O LYS_214@HZ2 LYS_214@NZ 45 0.0450 2.8160 151.7208

GLU_265@OE1 GLU_265@H GLU_265@N 45 0.0450 2.8446 153.1854

ASP_157@OD1 LYS_158@HZ3 LYS_158@NZ 44 0.0440 2.7910 154.5959

GLU_268@OE1 LYS_266@HZ1 LYS_266@NZ 44 0.0440 2.8118 157.7886

GLU_14@OE1 ASN_115@HD22 ASN_115@ND2 44 0.0440 2.8407 155.4873

VAL_426@O GLY_411@H GLY_411@N 44 0.0440 2.8547 153.1793

LYS_67@O THR_71@H THR_71@N 44 0.0440 2.8569 143.8984

ASN_189@OD1 ASN_190@H ASN_190@N 44 0.0440 2.8761 147.7095

SER_170@OG ASP_138@H ASP_138@N 44 0.0440 2.9045 140.6295

GLU_275@OE1 SER_261@HG SER_261@OG 43 0.0430 2.6631 164.9333

ALA_288@O SER_289@HG SER_289@OG 43 0.0430 2.8093 159.4281

ASN_115@O LYS_169@HZ1 LYS_169@NZ 43 0.0430 2.8299 150.6534

TYR_383@O PHE_345@H PHE_345@N 43 0.0430 2.9115 150.4995

SER_278@OG ASN_280@H ASN_280@N 43 0.0430 2.9170 153.9489

ASN_178@OD1 LYS_131@HZ1 LYS_131@NZ 42 0.0420 2.8235 151.2433

ASP_247@O ARG_264@HH11 ARG_264@NH1 42 0.0420 2.8768 147.6892

GLN_375@OE1 SER_151@HG SER_151@OG 41 0.0410 2.7072 162.9003

ASP_157@OD2 LYS_158@HZ3 LYS_158@NZ 41 0.0410 2.7754 154.5759

ASP_348@OD1 ASP_348@H ASP_348@N 41 0.0410 2.7962 138.1813

LEU_378@O LYS_373@HZ2 LYS_373@NZ 41 0.0410 2.7984 154.5242

ASP_152@OD1 VAL_153@H VAL_153@N 41 0.0410 2.8220 148.1799

GLN_221@OE1 ASP_224@H ASP_224@N 41 0.0410 2.8465 143.5606

GLU_433@OE1 SER_431@HG SER_431@OG 40 0.0400 2.6773 163.1134

ASP_152@OD1 LYS_179@HZ2 LYS_179@NZ 40 0.0400 2.7617 157.2692

ASP_181@OD1 LYS_179@HZ1 LYS_179@NZ 40 0.0400 2.7816 154.2261

GLU_327@OE2 ARG_437@HH21 ARG_437@NH2 40 0.0400 2.7981 155.7618

GLU_414@OE1 LYS_424@HZ2 LYS_424@NZ 40 0.0400 2.8026 158.3139

ASP_181@OD2 LYS_179@HZ1 LYS_179@NZ 40 0.0400 2.8085 155.8080

PHE_104@O GLN_1@HE21 GLN_1@NE2 40 0.0400 2.8211 155.9526

SER_291@OG THR_292@H THR_292@N 40 0.0400 2.8345 138.9507

GLU_14@OE1 ASN_115@H ASN_115@N 40 0.0400 2.8988 164.8882

ASP_157@OD1 LYS_158@HZ2 LYS_158@NZ 39 0.0390 2.7824 158.5138

GLU_275@O LYS_214@HZ1 LYS_214@NZ 39 0.0390 2.8057 151.9658

GLU_226@OE2 GLU_226@H GLU_226@N 39 0.0390 2.8665 147.7416

GLN_147@OE1 GLN_147@H GLN_147@N 38 0.0380 2.8329 153.9024

ASP_167@O GLN_117@HE21 GLN_117@NE2 38 0.0380 2.8337 157.9941

THR_137@OG1 ASP_138@H ASP_138@N 38 0.0380 2.8361 138.3407

ASP_234@OD1 ARG_232@HH21 ARG_232@NH2 38 0.0380 2.8530 145.4363

ASP_138@OD1 ASN_118@HD22 ASN_118@ND2 38 0.0380 2.8534 162.5511

ASP_162@O ARG_164@H ARG_164@N 38 0.0380 2.8844 143.1703

GLU_218@OE1 SER_203@HG SER_203@OG 37 0.0370 2.6516 166.4603

GLU_70@OE1 SER_72@HG SER_72@OG 37 0.0370 2.6663 165.4703

ASP_181@OD1 LYS_179@HZ2 LYS_179@NZ 37 0.0370 2.7512 158.2307

SER_276@O SER_278@HG SER_278@OG 37 0.0370 2.7755 157.3790

ASP_181@OD1 LYS_179@HZ3 LYS_179@NZ 37 0.0370 2.7805 155.5536

ASN_178@OD1 LYS_131@HZ2 LYS_131@NZ 37 0.0370 2.8190 153.5465

ASP_138@OD2 ASN_118@HD22 ASN_118@ND2 37 0.0370 2.8242 161.6491

ASP_84@O ALA_86@H ALA_86@N 37 0.0370 2.8367 142.5100

ASN_313@O VAL_315@H VAL_315@N 37 0.0370 2.8626 146.2565

PRO_235@O LEU_237@H LEU_237@N 37 0.0370 2.8724 143.2463

GLU_65@O HIE_74@H HIE_74@N 37 0.0370 2.9032 155.5556

ALA_53@O TYR_49@HH TYR_49@OH 36 0.0360 2.7141 165.7015

ASN_144@O SER_146@HG SER_146@OG 36 0.0360 2.7936 158.9504

ASP_84@OD1 ASN_60@HD21 ASN_60@ND2 36 0.0360 2.7999 153.9116

GLU_310@OE1 LYS_314@HZ3 LYS_314@NZ 36 0.0360 2.8010 159.7517

PRO_295@O LYS_48@HZ3 LYS_48@NZ 36 0.0360 2.8146 155.0403

LEU_102@O TYR_231@HH TYR_231@OH 36 0.0360 2.8235 153.1258

GLU_417@OE2 GLU_417@H GLU_417@N 36 0.0360 2.8277 149.9467

SER_261@OG VAL_262@H VAL_262@N 36 0.0360 2.8528 138.9514

ASP_130@OD1 ARG_127@HH22 ARG_127@NH2 36 0.0360 2.8644 154.5921

HIE_225@O GLN_221@HE21 GLN_221@NE2 36 0.0360 2.8670 155.6695

GLU_417@OE1 GLU_417@H GLU_417@N 36 0.0360 2.8730 150.9990

ILE_273@O GLU_275@H GLU_275@N 36 0.0360 2.8856 141.3783

THR_337@OG1 ARG_390@HH21 ARG_390@NH2 36 0.0360 2.9150 144.6657

SER_2@O SER_25@HG SER_25@OG 35 0.0350 2.7634 157.9490

GLU_327@OE1 ARG_437@HH21 ARG_437@NH2 35 0.0350 2.7768 156.3002

GLU_374@OE1 ASN_178@HD21 ASN_178@ND2 35 0.0350 2.8301 159.8766

GLU_324@OE2 ARG_127@HE ARG_127@NE 35 0.0350 2.8454 157.3955

GLU_324@OE1 ARG_127@HE ARG_127@NE 35 0.0350 2.8458 154.6677

GLY_42@O GLN_37@HE22 GLN_37@NE2 35 0.0350 2.8505 161.8801

SER_363@OG ARG_164@H ARG_164@N 35 0.0350 2.8885 153.8102

LYS_179@O PHE_182@H PHE_182@N 35 0.0350 2.9078 159.7390

SER_203@OG GLU_218@H GLU_218@N 35 0.0350 2.9128 159.4220

ASP_157@OD2 SER_146@HG SER_146@OG 34 0.0340 2.7127 163.9387

GLU_268@OE2 LYS_266@HZ2 LYS_266@NZ 34 0.0340 2.7742 158.8908

VAL_309@O THR_211@HG1 THR_211@OG1 34 0.0340 2.7748 160.4456

ASN_178@OD1 LYS_131@HZ3 LYS_131@NZ 34 0.0340 2.8023 153.5820

GLU_433@OE1 ARG_404@HE ARG_404@NE 34 0.0340 2.8357 153.6759

ASN_115@OD1 LYS_169@HZ2 LYS_169@NZ 34 0.0340 2.8461 151.1117

TRP_435@O ARG_437@HE ARG_437@NE 34 0.0340 2.8623 155.8748

GLN_370@OE1 GLN_147@HE21 GLN_147@NE2 34 0.0340 2.8818 158.3892

SER_291@OG GLY_293@H GLY_293@N 34 0.0340 2.9040 156.9120

MET_82@O ALA_85@H ALA_85@N 34 0.0340 2.9136 159.8583

SER_177@OG LYS_179@H LYS_179@N 34 0.0340 2.9223 163.1628

GLU_218@OE2 SER_271@HG SER_271@OG 33 0.0330 2.6454 164.6843

GLU_417@OE2 ARG_210@HH22 ARG_210@NH2 33 0.0330 2.7675 156.3888

GLU_417@OE2 ARG_210@HH21 ARG_210@NH2 33 0.0330 2.7985 157.8050

GLU_275@O LYS_214@HZ3 LYS_214@NZ 33 0.0330 2.8052 152.7646

ASP_162@OD2 MET_163@H MET_163@N 33 0.0330 2.8055 142.9176

SER_51@OG THR_50@HG1 THR_50@OG1 33 0.0330 2.8141 161.7468

THR_211@O ARG_210@HE ARG_210@NE 33 0.0330 2.8311 155.5703

THR_201@OG1 GLN_202@H GLN_202@N 33 0.0330 2.8320 138.1965

GLU_433@OE2 GLU_433@H GLU_433@N 33 0.0330 2.8534 145.8634

GLU_327@OE1 GLU_327@H GLU_327@N 33 0.0330 2.8682 149.5288

SER_93@O SER_95@H SER_95@N 33 0.0330 2.9045 142.2946

HIE_9@ND1 ARG_109@HE ARG_109@NE 33 0.0330 2.9101 152.4510

ARG_210@O GLU_213@H GLU_213@N 33 0.0330 2.9246 157.1612

LEU_46@O ILE_59@H ILE_59@N 33 0.0330 2.9481 157.3710

ASN_227@O SER_289@HG SER_289@OG 32 0.0320 2.7476 154.4349

THR_159@O SER_172@HG SER_172@OG 32 0.0320 2.7800 159.4540

GLU_268@OE1 LYS_266@HZ2 LYS_266@NZ 32 0.0320 2.7916 158.9772

GLU_94@OE1 LYS_101@HZ1 LYS_101@NZ 32 0.0320 2.8193 156.0864

GLU_319@O THR_343@HG1 THR_343@OG1 32 0.0320 2.8225 155.0851

PRO_295@O LYS_48@HZ2 LYS_48@NZ 32 0.0320 2.8257 155.3352

ASN_115@OD1 LYS_169@HZ1 LYS_169@NZ 32 0.0320 2.8302 151.1756

GLY_42@O GLN_44@HE22 GLN_44@NE2 32 0.0320 2.8346 154.9727

ASN_379@O LYS_373@HZ1 LYS_373@NZ 32 0.0320 2.8387 152.1990

ARG_127@O SER_132@HG SER_132@OG 31 0.0310 2.7256 159.6718

SER_79@O SER_79@HG SER_79@OG 31 0.0310 2.7573 139.4828

GLU_63@OE2 LYS_57@HZ2 LYS_57@NZ 31 0.0310 2.7694 153.1561

LEU_372@O SER_384@HG SER_384@OG 31 0.0310 2.7793 157.0061

SER_366@OG THR_159@HG1 THR_159@OG1 31 0.0310 2.7866 158.2177

MET_284@O SER_283@HG SER_283@OG 31 0.0310 2.8018 158.8065

GLU_414@OE1 GLU_414@H GLU_414@N 31 0.0310 2.8377 149.9190

LEU_378@O LYS_373@HZ1 LYS_373@NZ 31 0.0310 2.8513 153.4182

GLU_414@OE2 GLU_414@H GLU_414@N 31 0.0310 2.8696 149.1254

LEU_126@O ARG_127@HH21 ARG_127@NH2 31 0.0310 2.8733 142.6234

THR_111@OG1 HIE_9@HE2 HIE_9@NE2 31 0.0310 2.8915 158.8415

ASP_152@OD1 LYS_179@HZ3 LYS_179@NZ 30 0.0300 2.8155 155.4250

GLU_360@OE1 TRP_355@HE1 TRP_355@NE1 30 0.0300 2.8345 159.3998

ASP_380@OD1 ASN_379@HD22 ASN_379@ND2 30 0.0300 2.8377 158.9980

TYR_260@OH ARG_232@HH11 ARG_232@NH1 30 0.0300 2.8862 152.2369

SER_13@OG ASN_115@HD22 ASN_115@ND2 30 0.0300 2.8922 157.0199

PHE_409@O GLY_411@H GLY_411@N 30 0.0300 2.9171 144.4935

LYS_266@O ARG_269@H ARG_269@N 30 0.0300 2.9370 163.1556

ASP_157@OD1 SER_146@HG SER_146@OG 29 0.0290 2.7081 164.0096

SER_381@OG ARG_382@H ARG_382@N 29 0.0290 2.8021 138.9431

ASP_181@OD2 LYS_179@HZ2 LYS_179@NZ 29 0.0290 2.8267 157.8094

TYR_260@O TYR_243@HH TYR_243@OH 29 0.0290 2.8351 159.3450

ASP_120@O ASN_118@HD21 ASN_118@ND2 29 0.0290 2.8352 153.6596

GLU_14@OE2 ASN_115@HD22 ASN_115@ND2 29 0.0290 2.8514 159.0030

ASN_115@OD1 LYS_169@HZ3 LYS_169@NZ 29 0.0290 2.8701 153.2085

PRO_257@O TYR_260@H TYR_260@N 29 0.0290 2.9141 155.8581

GLU_374@OE1 SER_151@HG SER_151@OG 28 0.0280 2.6333 165.1756

GLU_414@O LYS_424@HZ2 LYS_424@NZ 28 0.0280 2.8097 145.6699

ASP_152@OD1 LYS_179@HZ1 LYS_179@NZ 28 0.0280 2.8143 161.0908

LEU_378@O LYS_373@HZ3 LYS_373@NZ 28 0.0280 2.8279 154.1255

SER_290@OG SER_291@H SER_291@N 28 0.0280 2.8296 137.0037

GLU_360@OE2 TRP_355@HE1 TRP_355@NE1 28 0.0280 2.8372 153.8453

SER_51@O ALA_53@H ALA_53@N 28 0.0280 2.8554 144.6993

SER_132@OG GLN_125@HE21 GLN_125@NE2 28 0.0280 2.8686 148.6934

GLN_428@OE1 ILE_429@H ILE_429@N 28 0.0280 2.9112 150.3133

GLU_87@OE2 ARG_109@HH11 ARG_109@NH1 27 0.0270 2.7704 160.0151

GLU_70@OE1 HIE_74@HE2 HIE_74@NE2 27 0.0270 2.8126 154.4471

PRO_295@O LYS_48@HZ1 LYS_48@NZ 27 0.0270 2.8244 153.6041

SER_386@OG ARG_388@HH12 ARG_388@NH1 27 0.0270 2.8557 147.8574

GLU_195@OE2 THR_197@H THR_197@N 27 0.0270 2.8602 159.7106

THR_156@O THR_156@HG1 THR_156@OG1 27 0.0270 2.8625 143.8086

SER_27@OG GLN_1@HE21 GLN_1@NE2 27 0.0270 2.8846 162.0125

ASN_189@OD1 ILE_192@H ILE_192@N 27 0.0270 2.8984 155.7861

LEU_294@O GLY_297@H GLY_297@N 27 0.0270 2.9015 155.1062

GLU_218@OE1 SER_271@HG SER_271@OG 26 0.0260 2.6202 164.3771

ASP_196@OD1 THR_197@HG1 THR_197@OG1 26 0.0260 2.7295 164.1602

GLU_417@OE2 ARG_210@HH12 ARG_210@NH1 26 0.0260 2.8101 149.4075

GLU_94@OE1 GLN_1@HE21 GLN_1@NE2 26 0.0260 2.8151 159.3569

GLU_414@OE2 ASN_415@HD22 ASN_415@ND2 26 0.0260 2.8154 163.3767

VAL_262@O LYS_251@HZ2 LYS_251@NZ 26 0.0260 2.8228 152.6766

ASP_157@OD1 ARG_109@HH22 ARG_109@NH2 26 0.0260 2.8326 157.0242

GLU_65@OE2 LYS_67@HZ1 LYS_67@NZ 26 0.0260 2.8401 152.0224

SER_141@O THR_143@H THR_143@N 26 0.0260 2.8523 145.3041

GLN_281@OE1 ALA_277@H ALA_277@N 26 0.0260 2.8554 149.1055

GLN_44@OE1 GLN_41@HE22 GLN_41@NE2 26 0.0260 2.8689 161.0696

GLU_329@O HIE_332@H HIE_332@N 26 0.0260 2.9210 147.7883

ASN_190@O SER_191@HG SER_191@OG 25 0.0250 2.6377 153.7797

VAL_153@O SER_148@HG SER_148@OG 25 0.0250 2.7585 163.7295

GLN_202@OE1 SER_204@HG SER_204@OG 25 0.0250 2.7837 153.6038

GLU_100@OE2 LYS_101@HZ2 LYS_101@NZ 25 0.0250 2.7942 155.8779

TYR_24@O THR_71@HG1 THR_71@OG1 25 0.0250 2.7951 157.0030

ASP_152@OD2 LYS_179@HZ2 LYS_179@NZ 25 0.0250 2.7969 155.4979

GLU_327@OE2 GLU_327@H GLU_327@N 25 0.0250 2.8277 152.0263

GLN_147@O LYS_149@HZ3 LYS_149@NZ 25 0.0250 2.8394 159.9088

ASP_181@OD1 ASP_181@H ASP_181@N 25 0.0250 2.8655 138.7313

ASP_157@OD2 GLN_370@HE21 GLN_370@NE2 25 0.0250 2.8714 157.0199

PRO_376@O LEU_378@H LEU_378@N 25 0.0250 2.8743 144.5530

SER_278@OG ASN_280@HD22 ASN_280@ND2 25 0.0250 2.8856 157.0427

LEU_110@O ALA_86@H ALA_86@N 25 0.0250 2.9104 143.4993

LYS_267@O ARG_264@HE ARG_264@NE 25 0.0250 2.9105 144.5002

SER_283@OG GLN_233@H GLN_233@N 25 0.0250 2.9445 150.5325

GLU_360@OE2 SER_353@HG SER_353@OG 24 0.0240 2.6438 164.2051

SER_129@OG ASP_130@H ASP_130@N 24 0.0240 2.8032 139.4234

ASP_152@OD2 LYS_179@HZ3 LYS_179@NZ 24 0.0240 2.8055 156.2266

ASP_181@OD2 LYS_179@HZ3 LYS_179@NZ 24 0.0240 2.8063 152.2754

GLU_414@OE1 ASN_415@HD22 ASN_415@ND2 24 0.0240 2.8068 159.0536

GLU_94@OE1 GLN_1@HE22 GLN_1@NE2 24 0.0240 2.8209 160.1642

ASP_120@O HIE_332@HE2 HIE_332@NE2 24 0.0240 2.8597 156.9507

ARG_305@O HIE_349@HE2 HIE_349@NE2 24 0.0240 2.8601 143.9106

THR_4@OG1 GLN_1@HE22 GLN_1@NE2 24 0.0240 2.8733 158.9613

GLU_94@OE1 LYS_101@HZ2 LYS_101@NZ 23 0.0230 2.7672 155.7653

GLU_414@O LYS_424@HZ3 LYS_424@NZ 23 0.0230 2.7897 146.1344

GLU_94@OE1 LYS_101@HZ3 LYS_101@NZ 23 0.0230 2.8116 156.9441

GLU_324@OE2 ARG_437@HH12 ARG_437@NH1 23 0.0230 2.8254 156.1690

GLN_406@OE1 ARG_404@HH12 ARG_404@NH1 23 0.0230 2.8314 150.4299

ASN_115@O LYS_169@HZ3 LYS_169@NZ 23 0.0230 2.8463 153.4596

ASN_40@OD1 ARG_205@HE ARG_205@NE 23 0.0230 2.8631 150.0825

ASP_157@OD1 GLN_370@HE21 GLN_370@NE2 23 0.0230 2.8752 159.1935

GLU_374@OE1 GLY_344@H GLY_344@N 23 0.0230 2.8822 151.0545

HIE_225@O ARG_264@HH22 ARG_264@NH2 23 0.0230 2.8878 144.0388

TYR_154@OH ASN_178@HD21 ASN_178@ND2 23 0.0230 2.8928 155.3200

GLU_268@OE2 LYS_266@HZ1 LYS_266@NZ 22 0.0220 2.7834 153.6698

ASP_380@OD2 ASN_379@HD22 ASN_379@ND2 22 0.0220 2.8197 161.0154

GLU_195@OE1 ASN_190@HD22 ASN_190@ND2 22 0.0220 2.8350 157.9093

ALA_438@OXT ARG_437@HH11 ARG_437@NH1 22 0.0220 2.8365 157.7949

GLU_94@OE2 GLN_1@HE22 GLN_1@NE2 22 0.0220 2.8469 161.4642

GLU_195@OE2 ASN_190@HD22 ASN_190@ND2 22 0.0220 2.8485 163.6323

ASP_421@OD1 ASP_421@H ASP_421@N 22 0.0220 2.8487 138.8097

SER_290@O GLU_226@H GLU_226@N 22 0.0220 2.8551 149.9001

ASP_138@O ASN_118@HD22 ASN_118@ND2 22 0.0220 2.8836 159.1510

SER_93@O GLN_1@HE21 GLN_1@NE2 22 0.0220 2.8848 154.2241

THR_304@O LEU_306@H LEU_306@N 22 0.0220 2.8942 140.5175

GLU_65@OE1 LYS_67@HZ3 LYS_67@NZ 21 0.0210 2.7724 158.0298

GLU_268@OE2 LYS_266@HZ3 LYS_266@NZ 21 0.0210 2.7731 161.0870

GLU_65@OE1 LYS_67@HZ1 LYS_67@NZ 21 0.0210 2.7789 149.6266

SER_271@OG SER_263@HG SER_263@OG 21 0.0210 2.7827 159.7067

ASP_152@OD2 LYS_179@HZ1 LYS_179@NZ 21 0.0210 2.8020 156.2701

SER_25@OG THR_4@HG1 THR_4@OG1 21 0.0210 2.8104 156.1370

ASP_421@OD2 ASP_421@H ASP_421@N 21 0.0210 2.8159 138.7653

SER_129@O ARG_127@HH11 ARG_127@NH1 21 0.0210 2.8218 159.5983

ASP_416@OD2 ARG_210@HH21 ARG_210@NH2 21 0.0210 2.8295 160.4409

GLU_360@OE1 HIE_362@HE2 HIE_362@NE2 21 0.0210 2.8382 157.0575

GLU_417@OE1 ARG_210@HH21 ARG_210@NH2 21 0.0210 2.8432 153.8893

GLY_303@O ASN_40@HD21 ASN_40@ND2 21 0.0210 2.8455 150.7071

GLN_370@O GLN_147@HE21 GLN_147@NE2 21 0.0210 2.8734 147.5562

GLU_70@OE2 ASN_23@HD21 ASN_23@ND2 21 0.0210 2.8927 161.3167

GLU_65@OE2 LYS_67@HZ2 LYS_67@NZ 20 0.0200 2.7779 156.5444

GLU_324@OE1 ARG_437@HH12 ARG_437@NH1 20 0.0200 2.7960 158.8750

SER_2@OG VAL_3@H VAL_3@N 20 0.0200 2.8084 138.5743

THR_143@O LYS_158@HZ3 LYS_158@NZ 20 0.0200 2.8108 153.5826

ASP_130@OD1 LYS_131@HZ2 LYS_131@NZ 20 0.0200 2.8259 154.7990

THR_427@O THR_427@HG1 THR_427@OG1 20 0.0200 2.8352 140.7532

ASP_348@O ARG_305@HH22 ARG_305@NH2 20 0.0200 2.8399 148.5215

GLN_44@OE1 GLN_41@HE21 GLN_41@NE2 20 0.0200 2.8440 154.4085

GLU_258@OE2 GLU_258@H GLU_258@N 20 0.0200 2.8595 148.6111

GLU_310@O ARG_210@HE ARG_210@NE 20 0.0200 2.8675 153.9887

VAL_262@O LYS_251@HZ1 LYS_251@NZ 20 0.0200 2.8847 153.6821

SER_13@OG ASN_115@HD21 ASN_115@ND2 20 0.0200 2.9030 159.3172

TRP_34@O LEU_47@H LEU_47@N 20 0.0200 2.9474 162.1774

GLU_268@OE1 LYS_266@HZ3 LYS_266@NZ 19 0.0190 2.7571 156.7676

GLY_212@O SER_276@HG SER_276@OG 19 0.0190 2.7608 155.6926

VAL_356@O GLY_358@H GLY_358@N 19 0.0190 2.7639 149.8039

ASP_311@OD2 LEU_312@H LEU_312@N 19 0.0190 2.8089 143.4784

GLU_414@O LYS_424@HZ1 LYS_424@NZ 19 0.0190 2.8151 154.7429

GLN_147@O LYS_149@HZ2 LYS_149@NZ 19 0.0190 2.8206 161.7430

GLU_417@O ARG_210@HH12 ARG_210@NH1 19 0.0190 2.8245 153.1822

ASN_115@OD1 ASN_115@H ASN_115@N 19 0.0190 2.8384 138.2124

GLU_433@OE1 GLU_433@H GLU_433@N 19 0.0190 2.8448 146.6518

ASN_401@OD1 ARG_400@HE ARG_400@NE 19 0.0190 2.8462 153.2337

ALA_393@O TRP_396@H TRP_396@N 19 0.0190 2.8610 145.9541

GLU_417@OE1 ARG_210@HE ARG_210@NE 19 0.0190 2.8625 157.4034

GLU_70@O ASN_23@HD22 ASN_23@ND2 19 0.0190 2.8900 144.9683

GLU_374@OE2 SER_384@HG SER_384@OG 18 0.0180 2.6987 156.0232

ASP_196@OD2 THR_197@HG1 THR_197@OG1 18 0.0180 2.7402 161.6249

GLU_94@OE2 LYS_101@HZ1 LYS_101@NZ 18 0.0180 2.7673 160.2878

ASP_157@OD2 ARG_109@HH22 ARG_109@NH2 18 0.0180 2.7893 157.8146

ASN_23@O THR_4@HG1 THR_4@OG1 18 0.0180 2.7952 153.8387

GLU_100@OE2 LYS_101@HZ1 LYS_101@NZ 18 0.0180 2.7989 155.7415

GLU_94@OE2 LYS_101@HZ2 LYS_101@NZ 18 0.0180 2.8031 157.7750

ASP_130@OD2 LYS_131@HZ2 LYS_131@NZ 18 0.0180 2.8153 158.0214

ASP_181@OD2 ASP_181@H ASP_181@N 18 0.0180 2.8232 138.9956

THR_108@O THR_108@HG1 THR_108@OG1 18 0.0180 2.8406 141.3015

THR_143@O LYS_158@HZ2 LYS_158@NZ 18 0.0180 2.8417 153.4349

SER_331@O GLN_334@HE21 GLN_334@NE2 18 0.0180 2.8430 153.2006

ASP_120@OD2 HIE_332@HE2 HIE_332@NE2 18 0.0180 2.8453 155.4904

ASN_178@OD1 LYS_179@HZ3 LYS_179@NZ 18 0.0180 2.8534 154.8421

ASP_140@O ASN_171@HD22 ASN_171@ND2 18 0.0180 2.8572 163.0697

GLU_360@OE1 GLN_406@HE21 GLN_406@NE2 18 0.0180 2.8710 155.1884

GLN_1@OE1 SER_2@H SER_2@N 18 0.0180 2.8745 144.7967

THR_211@OG1 ARG_210@HH21 ARG_210@NH2 18 0.0180 2.8746 153.7849

SER_132@OG SER_129@H SER_129@N 18 0.0180 2.8898 145.9363

GLU_374@OE2 SER_151@HG SER_151@OG 17 0.0170 2.7065 162.2625

PRO_78@O SER_79@HG SER_79@OG 17 0.0170 2.7348 157.6534

SER_263@OG SER_271@HG SER_271@OG 17 0.0170 2.7718 153.4671

GLU_100@OE2 LYS_101@HZ3 LYS_101@NZ 17 0.0170 2.7722 156.6210

ASN_115@O LYS_169@HZ2 LYS_169@NZ 17 0.0170 2.7896 150.8379

GLY_212@O LYS_214@HZ3 LYS_214@NZ 17 0.0170 2.7915 156.4312

ILE_116@O ASN_115@HD22 ASN_115@ND2 17 0.0170 2.8109 162.9769

GLU_374@OE2 GLY_344@H GLY_344@N 17 0.0170 2.8412 151.6170

GLU_417@OE1 ARG_210@HH12 ARG_210@NH1 17 0.0170 2.8497 155.0245

VAL_262@O LYS_251@HZ3 LYS_251@NZ 17 0.0170 2.8606 155.6332

GLU_195@O GLN_142@HE21 GLN_142@NE2 17 0.0170 2.8627 158.5963

ASN_379@O LYS_373@HZ2 LYS_373@NZ 17 0.0170 2.8635 155.4729

ASP_421@OD2 ARG_422@H ARG_422@N 17 0.0170 2.8649 140.3790

SER_177@OG LYS_179@HZ3 LYS_179@NZ 17 0.0170 2.8960 155.9355

THR_394@O GLN_397@H GLN_397@N 17 0.0170 2.9010 147.5824

SER_392@OG PHE_395@H PHE_395@N 17 0.0170 2.9084 155.6081

THR_427@OG1 GLY_411@H GLY_411@N 17 0.0170 2.9167 156.5783

SER_26@OG VAL_28@H VAL_28@N 17 0.0170 2.9308 161.3220

ARG_382@O SER_381@HG SER_381@OG 16 0.0160 2.7106 166.3868

GLU_265@OE1 ARG_269@HH12 ARG_269@NH1 16 0.0160 2.7424 153.0645

TYR_298@O TYR_35@HH TYR_35@OH 16 0.0160 2.7492 153.7311

GLU_94@OE2 GLN_1@HE21 GLN_1@NE2 16 0.0160 2.8217 155.9268

ASP_120@OD1 HIE_332@HE2 HIE_332@NE2 16 0.0160 2.8220 153.7092

ASP_130@OD2 LYS_131@HZ1 LYS_131@NZ 16 0.0160 2.8243 152.9541

SER_79@O LYS_77@HZ2 LYS_77@NZ 16 0.0160 2.8274 154.2114

ASP_167@O ARG_164@HH22 ARG_164@NH2 16 0.0160 2.8402 148.4712

GLU_268@OE1 ARG_269@HH11 ARG_269@NH1 16 0.0160 2.8523 154.3199

ASN_398@OD1 ARG_400@HH11 ARG_400@NH1 16 0.0160 2.8554 158.2071

GLU_327@OE2 ARG_437@HE ARG_437@NE 16 0.0160 2.8663 151.2440

TYR_296@O TYR_298@H TYR_298@N 16 0.0160 2.8679 145.5651

GLU_258@OE1 GLU_258@H GLU_258@N 16 0.0160 2.8812 148.3637

VAL_160@O THR_159@HG1 THR_159@OG1 16 0.0160 2.8871 157.4521

VAL_308@O THR_279@HG1 THR_279@OG1 15 0.0150 2.7573 166.0664

HIE_9@ND1 SER_8@HG SER_8@OG 15 0.0150 2.7813 162.0421

GLU_65@OE2 LYS_67@HZ3 LYS_67@NZ 15 0.0150 2.7879 158.5824

GLU_100@OE1 LYS_101@HZ1 LYS_101@NZ 15 0.0150 2.7933 160.0430

GLU_100@OE1 LYS_101@HZ2 LYS_101@NZ 15 0.0150 2.8039 154.1544

CYX_22@O SER_72@HG SER_72@OG 15 0.0150 2.8082 155.1972

TYR_49@OH SER_51@HG SER_51@OG 15 0.0150 2.8126 162.8192

ASP_368@OD1 ARG_388@HH21 ARG_388@NH2 15 0.0150 2.8168 150.5957

ARG_109@O THR_108@HG1 THR_108@OG1 15 0.0150 2.8229 156.8080

ILE_193@O SER_191@H SER_191@N 15 0.0150 2.8377 151.4626

ASP_130@OD1 LYS_131@HZ3 LYS_131@NZ 15 0.0150 2.8425 162.1287

GLU_360@OE2 HIE_362@HE2 HIE_362@NE2 15 0.0150 2.8490 153.5845

SER_51@OG ALA_52@H ALA_52@N 15 0.0150 2.8551 139.8808

PHE_139@O ASN_171@H ASN_171@N 15 0.0150 2.8564 153.7225

ASN_398@OD1 ARG_400@HE ARG_400@NE 15 0.0150 2.8621 150.6856

TYR_24@O SER_26@H SER_26@N 15 0.0150 2.8639 144.5908

GLU_310@O ARG_210@HH21 ARG_210@NH2 15 0.0150 2.8777 149.9502

GLU_414@O ASP_416@H ASP_416@N 15 0.0150 2.8855 143.9095

ASP_167@O LYS_169@H LYS_169@N 15 0.0150 2.9146 142.1299

SER_151@OG TYR_154@HH TYR_154@OH 14 0.0140 2.7868 159.3030

SER_146@OG GLN_147@H GLN_147@N 14 0.0140 2.8020 138.3520

SER_26@OG SER_2@HG SER_2@OG 14 0.0140 2.8253 160.5985

ASP_311@OD2 ARG_210@HH12 ARG_210@NH1 14 0.0140 2.8396 158.9397

THR_143@O LYS_158@HZ1 LYS_158@NZ 14 0.0140 2.8398 151.6340

THR_292@O LEU_294@H LEU_294@N 14 0.0140 2.8658 141.5700

TYR_38@O GLY_42@H GLY_42@N 14 0.0140 2.8806 155.0895

ALA_187@O ASN_189@H ASN_189@N 14 0.0140 2.8959 146.4977

HIE_81@ND1 LYS_77@HZ3 LYS_77@NZ 14 0.0140 2.9024 152.2630

THR_427@OG1 GLN_408@HE22 GLN_408@NE2 14 0.0140 2.9211 161.4663

THR_143@OG1 VAL_145@H VAL_145@N 14 0.0140 2.9236 162.2553

HIE_9@ND1 ARG_109@HH21 ARG_109@NH2 14 0.0140 2.9490 147.3794

GLU_360@OE1 SER_353@HG SER_353@OG 13 0.0130 2.6203 168.4754

GLU_374@O SER_151@HG SER_151@OG 13 0.0130 2.7229 160.9926

GLU_100@OE1 LYS_101@HZ3 LYS_101@NZ 13 0.0130 2.7509 153.4003

GLN_1@OE1 THR_103@HG1 THR_103@OG1 13 0.0130 2.7566 160.7162

ASP_311@OD2 ARG_210@HH21 ARG_210@NH2 13 0.0130 2.7854 156.4513

LEU_6@O ARG_21@HH21 ARG_21@NH2 13 0.0130 2.7944 158.7464

GLU_433@OE2 ARG_404@HH11 ARG_404@NH1 13 0.0130 2.8099 155.4440

THR_197@O THR_197@HG1 THR_197@OG1 13 0.0130 2.8145 140.2626

GLU_65@OE1 LYS_67@HZ2 LYS_67@NZ 13 0.0130 2.8194 157.8359

TRP_435@O ARG_437@HH21 ARG_437@NH2 13 0.0130 2.8221 160.8851

GLU_268@OE2 ARG_269@HH11 ARG_269@NH1 13 0.0130 2.8261 157.5697

SER_128@OG SER_129@H SER_129@N 13 0.0130 2.8321 137.7315

ASN_189@O SER_191@H SER_191@N 13 0.0130 2.8345 143.3010

ASN_23@OD1 ARG_21@HE ARG_21@NE 13 0.0130 2.8418 157.6819

GLN_41@OE1 GLN_44@HE22 GLN_44@NE2 13 0.0130 2.8528 155.3578

PHE_198@O VAL_123@H VAL_123@N 13 0.0130 2.8556 165.0942

ASN_379@O LYS_373@HZ3 LYS_373@NZ 13 0.0130 2.8582 150.1630

SER_79@O LYS_77@HZ3 LYS_77@NZ 13 0.0130 2.8640 154.2361

THR_211@O GLU_213@H GLU_213@N 13 0.0130 2.8872 141.8546

ASN_190@O ILE_192@H ILE_192@N 13 0.0130 2.8888 144.6666

THR_111@OG1 LYS_158@HZ3 LYS_158@NZ 13 0.0130 2.8914 158.6480

GLN_420@OE1 ARG_422@H ARG_422@N 13 0.0130 2.8998 163.7494

SER_2@OG SER_25@H SER_25@N 13 0.0130 2.9011 156.5868

ASN_118@OD1 ASP_120@H ASP_120@N 13 0.0130 2.9013 158.7681

MET_250@O TYR_246@H TYR_246@N 13 0.0130 2.9290 151.6190

GLU_218@OE2 SER_203@HG SER_203@OG 12 0.0120 2.6356 170.0805

GLU_275@OE1 SER_276@HG SER_276@OG 12 0.0120 2.7355 161.3876

ASP_222@OD1 LYS_199@HZ3 LYS_199@NZ 12 0.0120 2.7550 158.8823

GLN_281@O SER_283@HG SER_283@OG 12 0.0120 2.7795 157.1987

ASP_222@OD2 LYS_199@HZ1 LYS_199@NZ 12 0.0120 2.7842 157.5409

SER_51@O TYR_49@HH TYR_49@OH 12 0.0120 2.7877 150.4567

SER_363@OG SER_165@HG SER_165@OG 12 0.0120 2.8037 158.3952

LEU_43@O TYR_296@HH TYR_296@OH 12 0.0120 2.8037 154.1185

GLU_265@OE2 ARG_269@HH12 ARG_269@NH1 12 0.0120 2.8140 153.8530

THR_4@O THR_4@HG1 THR_4@OG1 12 0.0120 2.8194 142.4215

SER_27@O TYR_24@HH TYR_24@OH 12 0.0120 2.8289 164.6864

GLU_195@O ASN_190@HD22 ASN_190@ND2 12 0.0120 2.8339 166.3665

GLU_374@OE2 ASN_178@HD21 ASN_178@ND2 12 0.0120 2.8454 161.5816

THR_292@O THR_292@HG1 THR_292@OG1 12 0.0120 2.8508 140.2398

ASN_189@OD1 ASN_189@H ASN_189@N 12 0.0120 2.8529 141.1834

ASN_398@O ARG_400@H ARG_400@N 12 0.0120 2.8638 145.5007

SER_177@OG LYS_179@HZ2 LYS_179@NZ 12 0.0120 2.8655 155.8646

THR_71@OG1 ASN_23@HD22 ASN_23@ND2 12 0.0120 2.8727 147.7610

TYR_24@O ASN_23@HD22 ASN_23@ND2 12 0.0120 2.8733 163.4593

ASN_190@OD1 GLU_195@H GLU_195@N 12 0.0120 2.8763 162.4742

SER_128@OG GLN_125@HE22 GLN_125@NE2 12 0.0120 2.8907 158.7905

SER_177@OG LYS_179@HZ1 LYS_179@NZ 12 0.0120 2.8917 157.4505

SER_326@OG GLU_329@H GLU_329@N 12 0.0120 2.8953 154.7357

PHE_97@O ASN_99@H ASN_99@N 12 0.0120 2.9059 139.5423

GLN_1@OE1 VAL_3@H VAL_3@N 12 0.0120 2.9136 162.0023

TYR_410@OH ARG_205@HH12 ARG_205@NH1 12 0.0120 2.9137 155.3011

ASN_379@O SER_381@H SER_381@N 12 0.0120 2.9187 146.7336

SER_413@OG ASP_416@H ASP_416@N 12 0.0120 2.9485 152.0248

THR_50@O SER_51@HG SER_51@OG 11 0.0110 2.6759 148.0189

CYX_405@O SER_431@HG SER_431@OG 11 0.0110 2.7175 156.3443

GLU_417@OE1 ARG_210@HH22 ARG_210@NH2 11 0.0110 2.7300 157.7829

ASN_415@OD1 LYS_209@HZ1 LYS_209@NZ 11 0.0110 2.7816 149.9954

GLU_351@OE1 ARG_205@HH21 ARG_205@NH2 11 0.0110 2.7961 156.0007

LEU_161@O SER_170@HG SER_170@OG 11 0.0110 2.7969 159.4994

ASN_313@O ARG_422@HH12 ARG_422@NH1 11 0.0110 2.8031 149.4775

GLN_41@O SER_302@HG SER_302@OG 11 0.0110 2.8072 159.1091

ASP_348@OD2 ARG_305@HH11 ARG_305@NH1 11 0.0110 2.8112 155.5139

PRO_194@O ASP_196@H ASP_196@N 11 0.0110 2.8141 145.1926

ARG_400@O ASN_357@HD21 ASN_357@ND2 11 0.0110 2.8275 164.3851

SER_141@O THR_143@HG1 THR_143@OG1 11 0.0110 2.8340 156.3753

GLU_310@O THR_211@HG1 THR_211@OG1 11 0.0110 2.8345 157.9172

ASN_23@OD1 TYR_24@H TYR_24@N 11 0.0110 2.8407 144.9433

THR_76@OG1 LYS_77@H LYS_77@N 11 0.0110 2.8435 137.9173

SER_79@O LYS_77@HZ1 LYS_77@NZ 11 0.0110 2.8483 154.0952

GLU_417@OE2 ARG_210@HE ARG_210@NE 11 0.0110 2.8543 152.7088

ASN_401@OD1 ARG_400@HH21 ARG_400@NH2 11 0.0110 2.8681 145.9810

GLU_327@OE1 ARG_437@HE ARG_437@NE 11 0.0110 2.8685 157.7270

ASN_280@OD1 ASN_280@H ASN_280@N 11 0.0110 2.8693 139.8115

ARG_205@O ARG_205@HH11 ARG_205@NH1 11 0.0110 2.8730 159.8063

GLN_375@OE1 LEU_378@H LEU_378@N 11 0.0110 2.8775 156.3813

GLN_5@O GLY_7@H GLY_7@N 11 0.0110 2.8786 144.3773

ILE_330@O GLN_334@HE22 GLN_334@NE2 11 0.0110 2.8801 157.5207

SER_72@OG HIE_74@HE2 HIE_74@NE2 11 0.0110 2.8911 147.4598

ARG_205@O ASN_40@HD21 ASN_40@ND2 11 0.0110 2.9020 156.3605

SER_271@OG SER_263@H SER_263@N 11 0.0110 2.9233 144.4575

GLN_1@OE1 SER_27@H SER_27@N 11 0.0110 2.9259 157.3593

TYR_38@OH ASN_60@HD21 ASN_60@ND2 11 0.0110 2.9273 157.0891

GLN_1@OE1 SER_25@HG SER_25@OG 10 0.0100 2.6845 158.3984

ASN_313@OD1 ARG_422@HH22 ARG_422@NH2 10 0.0100 2.7605 153.4877

GLN_41@OE1 SER_302@HG SER_302@OG 10 0.0100 2.7610 159.8739

ASP_196@OD1 THR_197@H THR_197@N 10 0.0100 2.7749 151.0638

ASP_311@OD1 ARG_210@HH12 ARG_210@NH1 10 0.0100 2.7791 150.5867

SER_203@O SER_203@HG SER_203@OG 10 0.0100 2.7867 139.2355

ASP_130@OD2 ASP_130@H ASP_130@N 10 0.0100 2.8034 139.9553

ASP_311@OD1 LEU_312@H LEU_312@N 10 0.0100 2.8097 142.4988

GLY_259@O SER_276@HG SER_276@OG 10 0.0100 2.8127 158.3089

ASP_157@OD1 ARG_109@HH12 ARG_109@NH1 10 0.0100 2.8176 157.1272

LYS_251@O TYR_246@HH TYR_246@OH 10 0.0100 2.8302 156.6948

GLU_310@OE1 ASN_379@HD21 ASN_379@ND2 10 0.0100 2.8484 165.6832

GLN_420@OE1 ASP_421@H ASP_421@N 10 0.0100 2.8526 147.4691

ASN_401@OD1 ARG_400@HH11 ARG_400@NH1 10 0.0100 2.8583 158.9127

THR_307@OG1 HIE_349@HE2 HIE_349@NE2 10 0.0100 2.8647 147.1735

ALA_183@O ASN_186@HD22 ASN_186@ND2 10 0.0100 2.8693 160.3951

SER_95@OG ASN_99@H ASN_99@N 10 0.0100 2.9039 149.3492

GLU_329@O GLN_334@H GLN_334@N 10 0.0100 2.9097 160.3051

SER_302@OG GLN_202@HE22 GLN_202@NE2 10 0.0100 2.9136 160.2174

THR_299@OG1 SER_289@HG SER_289@OG 10 0.0100 2.9167 142.5116

GLN_420@O TRP_418@HE1 TRP_418@NE1 10 0.0100 2.9280 140.7845

SER_384@OG GLY_344@H GLY_344@N 10 0.0100 2.9342 155.4997

TYR_296@OH LEU_45@H LEU_45@N 10 0.0100 2.9353 158.1526

THR_54@O VAL_56@H VAL_56@N 10 0.0100 2.9430 143.8669

GLU_226@OE1 THR_292@HG1 THR_292@OG1 9 0.0090 2.6290 167.0972

ASN_379@O TYR_383@HH TYR_383@OH 9 0.0090 2.7108 164.1378

GLU_275@OE2 SER_276@HG SER_276@OG 9 0.0090 2.7186 161.5767

GLU_213@OE1 ARG_210@HH12 ARG_210@NH1 9 0.0090 2.7659 157.0886

GLU_360@OE2 GLN_406@HE21 GLN_406@NE2 9 0.0090 2.7865 154.5909

GLN_281@OE1 SER_276@HG SER_276@OG 9 0.0090 2.7881 155.1964

LYS_249@O LYS_251@HZ3 LYS_251@NZ 9 0.0090 2.8055 152.2199

LEU_19@O ARG_21@HH11 ARG_21@NH1 9 0.0090 2.8057 150.9884

ASN_178@OD1 LYS_179@HZ2 LYS_179@NZ 9 0.0090 2.8073 154.2351

ASN_178@OD1 LYS_179@HZ1 LYS_179@NZ 9 0.0090 2.8077 148.8283

ASP_311@OD1 LYS_314@HZ1 LYS_314@NZ 9 0.0090 2.8095 158.4167

PRO_376@O LYS_373@HZ1 LYS_373@NZ 9 0.0090 2.8167 150.4741

ASP_181@O LYS_179@HZ3 LYS_179@NZ 9 0.0090 2.8211 159.1264

SER_25@O SER_25@HG SER_25@OG 9 0.0090 2.8255 139.7736

ASP_311@OD2 ARG_210@HH22 ARG_210@NH2 9 0.0090 2.8297 150.4807

SER_204@OG ARG_205@H ARG_205@N 9 0.0090 2.8340 138.3269

GLY_107@O THR_106@HG1 THR_106@OG1 9 0.0090 2.8395 163.7006

ASP_130@OD2 LYS_131@HZ3 LYS_131@NZ 9 0.0090 2.8641 158.4848

ALA_328@O SER_331@H SER_331@N 9 0.0090 2.8649 143.2488

SER_151@O GLN_375@HE21 GLN_375@NE2 9 0.0090 2.8655 160.6749

SER_431@OG ARG_404@HH21 ARG_404@NH2 9 0.0090 2.8702 141.7232

THR_50@OG1 LYS_48@HZ3 LYS_48@NZ 9 0.0090 2.8756 152.3483

ASP_152@O ASN_178@H ASN_178@N 9 0.0090 2.8769 153.3752

LEU_110@O ARG_109@HH11 ARG_109@NH1 9 0.0090 2.8789 148.2764

ASN_398@OD1 ASN_401@H ASN_401@N 9 0.0090 2.9093 149.9662

GLU_310@OE1 ASP_311@H ASP_311@N 9 0.0090 2.9125 148.0131

LEU_378@O SER_381@H SER_381@N 9 0.0090 2.9240 155.1879

SER_151@OG ALA_377@H ALA_377@N 9 0.0090 2.9266 150.5499

GLU_374@OE1 SER_384@HG SER_384@OG 8 0.0080 2.6758 155.1820

ASN_313@OD1 THR_419@HG1 THR_419@OG1 8 0.0080 2.7195 156.9477

ILE_113@O SER_13@HG SER_13@OG 8 0.0080 2.7506 146.8331

GLU_195@OE1 GLN_142@HE22 GLN_142@NE2 8 0.0080 2.7615 157.3832

ASP_421@OD1 ARG_422@HH21 ARG_422@NH2 8 0.0080 2.7702 159.2103

SER_276@OG SER_278@HG SER_278@OG 8 0.0080 2.7837 158.3043

LYS_373@O TYR_154@HH TYR_154@OH 8 0.0080 2.7849 167.0365

ASP_130@OD1 LYS_131@HZ1 LYS_131@NZ 8 0.0080 2.8074 158.9089

ASP_130@O SER_180@HG SER_180@OG 8 0.0080 2.8088 161.6394

ASP_416@O TRP_418@H TRP_418@N 8 0.0080 2.8120 144.9544

SER_276@O SER_278@H SER_278@N 8 0.0080 2.8175 145.2515

ASP_162@OD1 LYS_169@HZ1 LYS_169@NZ 8 0.0080 2.8191 156.3668

ASN_115@OD1 GLN_117@HE22 GLN_117@NE2 8 0.0080 2.8233 161.9889

THR_159@OG1 SER_366@HG SER_366@OG 8 0.0080 2.8292 149.1398

ASN_313@OD1 ARG_422@HH12 ARG_422@NH1 8 0.0080 2.8306 155.1150

GLU_265@OE1 LYS_266@HZ2 LYS_266@NZ 8 0.0080 2.8328 155.7862

ASP_167@O GLN_117@HE22 GLN_117@NE2 8 0.0080 2.8395 165.0916

ASP_140@OD2 SER_141@H SER_141@N 8 0.0080 2.8443 146.4913

GLU_94@OE2 LYS_101@HZ3 LYS_101@NZ 8 0.0080 2.8479 153.4937

SER_128@O ARG_127@HH11 ARG_127@NH1 8 0.0080 2.8484 151.1861

LYS_179@O ASP_181@H ASP_181@N 8 0.0080 2.8484 142.4570

ALA_85@O ARG_109@HH12 ARG_109@NH1 8 0.0080 2.8490 149.4857

ASN_190@OD1 ASN_190@H ASN_190@N 8 0.0080 2.8504 137.3024

ASP_150@OD1 LYS_149@H LYS_149@N 8 0.0080 2.8553 153.8774

PRO_114@O ILE_116@H ILE_116@N 8 0.0080 2.8602 140.2140

GLU_226@OE1 THR_292@H THR_292@N 8 0.0080 2.8619 156.6776

ASP_421@OD1 ARG_422@H ARG_422@N 8 0.0080 2.8673 140.0928

ASP_380@OD2 SER_381@H SER_381@N 8 0.0080 2.8726 145.0658

ASN_415@OD1 LYS_209@HZ2 LYS_209@NZ 8 0.0080 2.8730 151.7512

LYS_77@O SER_79@H SER_79@N 8 0.0080 2.8749 143.8175

SER_25@OG GLN_1@HE21 GLN_1@NE2 8 0.0080 2.8994 159.3859

ILE_429@O GLN_428@HE22 GLN_428@NE2 8 0.0080 2.9065 155.2613

LEU_274@O VAL_215@H VAL_215@N 8 0.0080 2.9146 155.5349

SER_129@OG LYS_131@H LYS_131@N 8 0.0080 2.9374 161.9199

GLU_70@OE2 SER_72@HG SER_72@OG 7 0.0070 2.7105 160.9971

SER_180@O SER_132@HG SER_132@OG 7 0.0070 2.7176 162.1289

SER_278@OG SER_276@HG SER_276@OG 7 0.0070 2.7180 164.3170

ASP_222@OD1 LYS_199@HZ2 LYS_199@NZ 7 0.0070 2.7410 153.2770

GLU_252@OE1 LYS_253@HZ3 LYS_253@NZ 7 0.0070 2.7432 151.9748

SER_11@O SER_11@HG SER_11@OG 7 0.0070 2.7465 141.1797

ASP_157@OD2 ARG_109@HH12 ARG_109@NH1 7 0.0070 2.7594 156.4118

ASP_167@OD1 LYS_169@HZ3 LYS_169@NZ 7 0.0070 2.7622 157.2696

PHE_104@O SER_2@HG SER_2@OG 7 0.0070 2.7886 158.3255

GLU_265@OE2 LYS_266@HZ2 LYS_266@NZ 7 0.0070 2.7923 152.2921

GLU_360@OE2 LYS_359@HZ3 LYS_359@NZ 7 0.0070 2.8031 153.0788

PRO_399@O ARG_400@HH11 ARG_400@NH1 7 0.0070 2.8040 155.6929

SER_25@O SER_2@HG SER_2@OG 7 0.0070 2.8057 153.8527

SER_353@O GLN_406@HE22 GLN_406@NE2 7 0.0070 2.8079 155.2536

ASP_150@O SER_148@HG SER_148@OG 7 0.0070 2.8148 151.2970

ASP_162@OD1 LYS_169@HZ2 LYS_169@NZ 7 0.0070 2.8166 158.1458

ASP_222@OD2 LYS_199@HZ3 LYS_199@NZ 7 0.0070 2.8194 158.3330

ASN_415@OD1 LYS_209@HZ3 LYS_209@NZ 7 0.0070 2.8224 148.1058

ASP_167@O LYS_169@HZ1 LYS_169@NZ 7 0.0070 2.8259 155.6949

ASP_311@OD1 ARG_210@HH21 ARG_210@NH2 7 0.0070 2.8265 147.9514

ASP_311@OD2 ARG_210@HE ARG_210@NE 7 0.0070 2.8377 150.1340

ASP_157@OD1 GLN_370@HE22 GLN_370@NE2 7 0.0070 2.8384 166.3441

PHE_73@O CYX_22@H CYX_22@N 7 0.0070 2.8387 143.0070

SER_25@O GLN_1@HE22 GLN_1@NE2 7 0.0070 2.8440 164.0296

VAL_3@O GLN_1@HE21 GLN_1@NE2 7 0.0070 2.8441 153.9955

LEU_55@O LYS_57@HZ3 LYS_57@NZ 7 0.0070 2.8501 161.3695

TYR_243@OH LYS_251@HZ1 LYS_251@NZ 7 0.0070 2.8505 151.3201

LYS_249@O LYS_251@HZ1 LYS_251@NZ 7 0.0070 2.8549 155.9688

SER_413@OG ASN_415@HD22 ASN_415@ND2 7 0.0070 2.8654 150.0959

GLN_406@O SER_353@HG SER_353@OG 7 0.0070 2.8664 149.9812

PRO_194@O GLN_142@HE22 GLN_142@NE2 7 0.0070 2.8670 153.8479

ASP_130@OD2 ARG_127@HH21 ARG_127@NH2 7 0.0070 2.8673 164.7606

LEU_45@O GLN_44@HE22 GLN_44@NE2 7 0.0070 2.8675 159.1557

GLU_360@OE2 LYS_359@HZ2 LYS_359@NZ 7 0.0070 2.8687 152.8452

SER_25@O GLN_1@H2 GLN_1@N 7 0.0070 2.8698 152.7429

SER_25@OG SER_2@H SER_2@N 7 0.0070 2.8714 143.3495

GLN_44@OE1 LEU_45@H LEU_45@N 7 0.0070 2.8747 151.5832

SER_290@OG ASN_227@HD22 ASN_227@ND2 7 0.0070 2.8779 157.9644

GLN_41@O LEU_43@H LEU_43@N 7 0.0070 2.8791 143.0972

SER_148@O GLN_147@HE22 GLN_147@NE2 7 0.0070 2.8818 160.2270

PRO_39@O GLN_41@H GLN_41@N 7 0.0070 2.8820 146.3325

GLN_1@O VAL_3@H VAL_3@N 7 0.0070 2.8855 146.0955

SER_141@OG GLN_142@HE22 GLN_142@NE2 7 0.0070 2.8856 166.0952

ASP_181@OD1 ASN_186@HD21 ASN_186@ND2 7 0.0070 2.8918 151.6610

THR_143@O VAL_145@H VAL_145@N 7 0.0070 2.8994 143.2120

PHE_66@O LYS_68@H LYS_68@N 7 0.0070 2.9005 143.7957

THR_343@O THR_343@HG1 THR_343@OG1 7 0.0070 2.9039 138.6169

ASN_115@OD1 ILE_116@H ILE_116@N 7 0.0070 2.9085 138.1848

HIE_74@ND1 SER_72@HG SER_72@OG 7 0.0070 2.9090 152.3396

GLU_14@OE1 MET_82@H MET_82@N 7 0.0070 2.9176 152.7475

SER_151@O ASN_178@HD21 ASN_178@ND2 7 0.0070 2.9182 150.0896

SER_93@OG GLU_100@H GLU_100@N 7 0.0070 2.9569 153.1535

ASP_181@O SER_177@HG SER_177@OG 6 0.0060 2.6896 161.6763

ASP_120@OD1 THR_197@HG1 THR_197@OG1 6 0.0060 2.6986 162.8220

ASP_140@O SER_13@HG SER_13@OG 6 0.0060 2.7132 162.7979

SER_93@OG SER_95@HG SER_95@OG 6 0.0060 2.7229 156.8900

SER_51@O SER_51@HG SER_51@OG 6 0.0060 2.7272 143.8793

SER_276@O SER_276@HG SER_276@OG 6 0.0060 2.7390 140.6191

GLU_213@OE2 ARG_210@HH22 ARG_210@NH2 6 0.0060 2.7446 150.4576

SER_151@O TYR_154@HH TYR_154@OH 6 0.0060 2.7484 148.4902

ASP_162@OD2 LYS_169@HZ1 LYS_169@NZ 6 0.0060 2.7701 160.1118

GLN_147@OE1 LYS_149@HZ1 LYS_149@NZ 6 0.0060 2.7761 153.8579

THR_50@O THR_50@HG1 THR_50@OG1 6 0.0060 2.7800 138.9989

SER_151@O SER_151@HG SER_151@OG 6 0.0060 2.7803 140.1628

GLN_375@O ALA_377@H ALA_377@N 6 0.0060 2.7832 151.1157

GLN_41@OE1 LYS_199@HZ1 LYS_199@NZ 6 0.0060 2.7863 158.3296

ASP_130@OD1 ARG_127@HH21 ARG_127@NH2 6 0.0060 2.7953 153.9188

ASP_348@OD1 ARG_305@HH11 ARG_305@NH1 6 0.0060 2.8037 157.7406

HIE_225@O LYS_267@HZ1 LYS_267@NZ 6 0.0060 2.8055 153.4781

ASP_196@OD2 THR_197@H THR_197@N 6 0.0060 2.8082 147.5582

SER_331@O GLN_334@HE22 GLN_334@NE2 6 0.0060 2.8122 148.2160

ASP_421@OD2 ARG_422@HE ARG_422@NE 6 0.0060 2.8151 158.5404

GLN_370@OE1 GLN_147@HE22 GLN_147@NE2 6 0.0060 2.8203 159.7081

GLU_258@OE1 LYS_253@HZ2 LYS_253@NZ 6 0.0060 2.8253 150.2862

ASN_178@OD1 ASN_178@H ASN_178@N 6 0.0060 2.8272 137.9201

PRO_371@O GLN_370@HE22 GLN_370@NE2 6 0.0060 2.8369 159.9802

THR_282@O ARG_232@HH22 ARG_232@NH2 6 0.0060 2.8374 144.2853

SER_132@O SER_177@HG SER_177@OG 6 0.0060 2.8384 160.1512

ASP_181@O LYS_179@HZ1 LYS_179@NZ 6 0.0060 2.8402 160.4467

ASP_181@O ALA_183@H ALA_183@N 6 0.0060 2.8423 145.8434

ASN_227@OD1 MET_228@H MET_228@N 6 0.0060 2.8429 139.3031

ASN_280@OD1 ARG_232@HH12 ARG_232@NH1 6 0.0060 2.8444 158.3673

ASP_311@OD1 ASN_313@HD21 ASN_313@ND2 6 0.0060 2.8455 153.2669

GLY_212@O LYS_214@HZ2 LYS_214@NZ 6 0.0060 2.8467 153.1873

SER_13@O GLY_15@H GLY_15@N 6 0.0060 2.8520 150.9761

PHE_168@O SER_170@H SER_170@N 6 0.0060 2.8522 146.6360

GLN_420@OE1 ASN_313@HD22 ASN_313@ND2 6 0.0060 2.8525 162.6445

ASP_167@OD1 LYS_169@HZ1 LYS_169@NZ 6 0.0060 2.8550 154.5615

THR_103@OG1 PHE_104@H PHE_104@N 6 0.0060 2.8596 136.6909

THR_211@O THR_211@HG1 THR_211@OG1 6 0.0060 2.8709 143.8854

GLU_360@OE1 ARG_404@HH22 ARG_404@NH2 6 0.0060 2.8726 148.9923

THR_419@OG1 ARG_210@HH12 ARG_210@NH1 6 0.0060 2.8765 152.2977

ILE_193@O ASN_190@HD22 ASN_190@ND2 6 0.0060 2.8767 160.2473

LEU_19@O ARG_21@HE ARG_21@NE 6 0.0060 2.8775 147.8349

SER_191@OG ASN_189@HD22 ASN_189@ND2 6 0.0060 2.8794 152.8958

ALA_438@OXT GLN_397@HE21 GLN_397@NE2 6 0.0060 2.8795 165.3784

GLU_351@OE2 ARG_205@HE ARG_205@NE 6 0.0060 2.8798 163.2549

GLU_351@OE1 LEU_352@H LEU_352@N 6 0.0060 2.8811 150.3151

ASN_171@OD1 SER_172@H SER_172@N 6 0.0060 2.8822 142.5522

VAL_3@O THR_4@HG1 THR_4@OG1 6 0.0060 2.8827 149.9425

SER_151@OG GLN_375@HE21 GLN_375@NE2 6 0.0060 2.8840 156.7768

ASP_234@OD1 SER_283@HG SER_283@OG 6 0.0060 2.8900 146.9228

GLU_195@OE2 GLN_142@HE22 GLN_142@NE2 6 0.0060 2.8967 158.4690

GLN_428@OE1 VAL_426@H VAL_426@N 6 0.0060 2.9009 156.4501

TYR_410@OH GLN_408@HE22 GLN_408@NE2 6 0.0060 2.9016 149.1754

SER_25@OG GLN_1@H3 GLN_1@N 6 0.0060 2.9060 152.5904

SER_170@OG ARG_390@HH12 ARG_390@NH1 6 0.0060 2.9126 143.1625

LEU_352@O THR_367@HG1 THR_367@OG1 6 0.0060 2.9274 150.8350

ALA_438@O GLN_397@HE21 GLN_397@NE2 6 0.0060 2.9345 161.4266

GLN_406@NE2 ARG_404@HH21 ARG_404@NH2 6 0.0060 2.9380 159.3446

SER_95@OG GLY_98@H GLY_98@N 6 0.0060 2.9640 149.6769

SER_25@O SER_26@HG SER_26@OG 5 0.0050 2.6944 153.1261

GLN_233@O SER_283@HG SER_283@OG 5 0.0050 2.7088 152.8743

GLN_1@OE1 THR_4@HG1 THR_4@OG1 5 0.0050 2.7098 160.8037

ASP_167@O LYS_169@HZ2 LYS_169@NZ 5 0.0050 2.7254 151.0902

GLU_14@O SER_79@HG SER_79@OG 5 0.0050 2.7264 154.8385

GLU_213@OE1 LYS_209@HZ1 LYS_209@NZ 5 0.0050 2.7406 155.3653

GLU_360@OE2 ARG_404@HH22 ARG_404@NH2 5 0.0050 2.7413 158.3822

GLN_44@OE1 TYR_296@HH TYR_296@OH 5 0.0050 2.7445 155.6271

GLU_213@OE2 ARG_210@HH12 ARG_210@NH1 5 0.0050 2.7446 146.1730

GLU_360@OE1 LYS_359@HZ2 LYS_359@NZ 5 0.0050 2.7591 154.6652

GLY_411@O THR_427@HG1 THR_427@OG1 5 0.0050 2.7749 144.1466

VAL_350@O TYR_206@HH TYR_206@OH 5 0.0050 2.7804 149.3947

TYR_383@O SER_384@HG SER_384@OG 5 0.0050 2.7820 142.1443

GLU_275@O SER_276@HG SER_276@OG 5 0.0050 2.7853 154.9337

VAL_208@O LYS_209@HZ3 LYS_209@NZ 5 0.0050 2.7959 160.4333

ALA_85@O ARG_109@HH21 ARG_109@NH2 5 0.0050 2.7984 157.7540

ASP_421@OD1 GLN_420@HE21 GLN_420@NE2 5 0.0050 2.7990 149.9155

GLU_14@OE1 ASN_115@HD21 ASN_115@ND2 5 0.0050 2.8015 154.2813

ASP_150@O ASP_152@H ASP_152@N 5 0.0050 2.8039 140.2370

GLU_265@OE2 LYS_266@HZ1 LYS_266@NZ 5 0.0050 2.8060 156.9456

LEU_55@O LYS_57@HZ2 LYS_57@NZ 5 0.0050 2.8072 154.8928

GLN_406@OE1 ARG_404@HH22 ARG_404@NH2 5 0.0050 2.8107 149.3504

SER_381@O LYS_373@HZ2 LYS_373@NZ 5 0.0050 2.8152 155.3897

GLU_360@O LYS_359@HZ3 LYS_359@NZ 5 0.0050 2.8157 156.5662

THR_419@O THR_419@HG1 THR_419@OG1 5 0.0050 2.8158 137.1149

GLN_147@O LYS_149@HZ1 LYS_149@NZ 5 0.0050 2.8178 153.5082

ASN_23@OD1 ARG_21@HH21 ARG_21@NH2 5 0.0050 2.8228 149.3300

GLU_374@OE2 ARG_382@HH11 ARG_382@NH1 5 0.0050 2.8281 155.1541

ASP_181@O LYS_179@HZ2 LYS_179@NZ 5 0.0050 2.8287 154.7892

SER_203@OG SER_204@HG SER_204@OG 5 0.0050 2.8308 166.1326

ASN_313@O ARG_422@HH21 ARG_422@NH2 5 0.0050 2.8322 142.0732

LYS_249@O LYS_251@HZ2 LYS_251@NZ 5 0.0050 2.8358 158.3340

CYX_22@O ASN_23@HD22 ASN_23@ND2 5 0.0050 2.8359 151.3364

SER_129@O ARG_127@HH21 ARG_127@NH2 5 0.0050 2.8397 150.9417

ASP_157@OD2 HIE_9@HE2 HIE_9@NE2 5 0.0050 2.8399 159.8407

GLU_360@OE1 LYS_359@HZ1 LYS_359@NZ 5 0.0050 2.8414 152.8392

ARG_127@O GLN_125@HE21 GLN_125@NE2 5 0.0050 2.8445 149.0192

ASP_196@OD1 ASP_196@H ASP_196@N 5 0.0050 2.8454 138.2531

HIE_332@O GLN_334@HE22 GLN_334@NE2 5 0.0050 2.8466 156.6778

GLU_433@OE1 ARG_404@HH11 ARG_404@NH1 5 0.0050 2.8494 155.5707

SER_25@OG GLN_1@H1 GLN_1@N 5 0.0050 2.8513 152.0707

ASN_313@OD1 ARG_422@HH21 ARG_422@NH2 5 0.0050 2.8553 156.8424

ASP_311@OD1 LYS_314@HZ3 LYS_314@NZ 5 0.0050 2.8571 164.8093

SER_2@O THR_4@HG1 THR_4@OG1 5 0.0050 2.8620 154.7660

THR_211@O ARG_210@HH21 ARG_210@NH2 5 0.0050 2.8687 143.8270

GLN_117@OE1 ASN_115@HD22 ASN_115@ND2 5 0.0050 2.8748 159.8923

SER_148@OG ASP_150@H ASP_150@N 5 0.0050 2.8757 163.5403

SER_191@OG ASN_189@HD21 ASN_189@ND2 5 0.0050 2.8792 147.9570

THR_307@OG1 ARG_305@HH22 ARG_305@NH2 5 0.0050 2.8805 153.0620

SER_8@OG ARG_109@HH21 ARG_109@NH2 5 0.0050 2.8809 147.6432

HIE_74@ND1 ARG_21@HH11 ARG_21@NH1 5 0.0050 2.8835 151.2441

VAL_322@O ARG_127@HH22 ARG_127@NH2 5 0.0050 2.8872 154.0090

LEU_274@O ALA_277@H ALA_277@N 5 0.0050 2.8872 146.8152

GLU_258@OE2 LYS_253@HZ2 LYS_253@NZ 5 0.0050 2.8874 152.8163

THR_143@OG1 LYS_158@HZ3 LYS_158@NZ 5 0.0050 2.8878 153.8525

SER_26@OG GLN_1@HE22 GLN_1@NE2 5 0.0050 2.8882 151.8468

THR_419@O ASN_313@HD21 ASN_313@ND2 5 0.0050 2.8886 155.9501

TYR_243@OH LYS_251@HZ2 LYS_251@NZ 5 0.0050 2.8953 161.9651

SER_69@OG LYS_67@HZ2 LYS_67@NZ 5 0.0050 2.9012 159.3626

SER_11@OG LYS_158@HZ2 LYS_158@NZ 5 0.0050 2.9099 161.1460

TYR_410@OH ARG_205@HH22 ARG_205@NH2 5 0.0050 2.9135 157.9325

ASN_401@ND2 ASN_398@HD22 ASN_398@ND2 5 0.0050 2.9197 151.1054

ALA_393@O GLN_397@HE22 GLN_397@NE2 5 0.0050 2.9303 165.3026

THR_419@OG1 ASN_313@HD21 ASN_313@ND2 5 0.0050 2.9311 164.0296

SER_283@OG ARG_232@HE ARG_232@NE 5 0.0050 2.9414 147.4167

SER_51@OG ALA_53@H ALA_53@N 5 0.0050 2.9639 161.6259

GLN_375@O SER_381@HG SER_381@OG 4 0.0040 2.6309 164.8337

PHE_198@O THR_197@HG1 THR_197@OG1 4 0.0040 2.6649 162.2678

HIE_225@ND1 SER_291@HG SER_291@OG 4 0.0040 2.6879 160.1859

ASN_115@OD1 SER_13@HG SER_13@OG 4 0.0040 2.7035 150.6919

ASP_380@OD2 LYS_373@HZ2 LYS_373@NZ 4 0.0040 2.7145 155.4244

GLU_265@OE1 LYS_266@HZ3 LYS_266@NZ 4 0.0040 2.7172 147.3256

ASP_167@OD2 LYS_169@HZ1 LYS_169@NZ 4 0.0040 2.7195 152.5472

THR_282@O SER_283@HG SER_283@OG 4 0.0040 2.7447 151.4913

GLN_281@OE1 ARG_232@HH12 ARG_232@NH1 4 0.0040 2.7538 142.2361

GLY_212@O LYS_214@HZ1 LYS_214@NZ 4 0.0040 2.7584 157.6247

SER_177@OG ASN_178@H ASN_178@N 4 0.0040 2.7662 138.0896

SER_27@O LYS_68@HZ3 LYS_68@NZ 4 0.0040 2.7760 145.7229

GLN_1@NE2 THR_4@HG1 THR_4@OG1 4 0.0040 2.7778 158.5262

ASP_150@OD1 GLN_375@HE22 GLN_375@NE2 4 0.0040 2.7789 169.8659

ASP_157@O THR_159@HG1 THR_159@OG1 4 0.0040 2.7859 147.3028

ASP_196@OD2 ASP_196@H ASP_196@N 4 0.0040 2.7871 137.3636

GLU_258@OE2 LYS_253@HZ1 LYS_253@NZ 4 0.0040 2.7899 149.8980

ASP_162@OD1 LYS_169@HZ3 LYS_169@NZ 4 0.0040 2.7906 153.3941

ASP_380@OD2 ASP_380@H ASP_380@N 4 0.0040 2.7924 138.1075

ASP_140@O LYS_158@HZ1 LYS_158@NZ 4 0.0040 2.7955 166.9471

ASP_222@OD2 LYS_199@HZ2 LYS_199@NZ 4 0.0040 2.7963 160.3391

ALA_53@O SER_51@HG SER_51@OG 4 0.0040 2.8005 163.0155

ARG_127@O SER_129@HG SER_129@OG 4 0.0040 2.8009 158.4957

SER_302@OG GLY_303@H GLY_303@N 4 0.0040 2.8022 139.8164

ASP_421@OD2 ARG_422@HH21 ARG_422@NH2 4 0.0040 2.8038 152.5174

ALA_438@OXT ARG_437@HE ARG_437@NE 4 0.0040 2.8122 163.7171

PRO_369@O ARG_109@HH12 ARG_109@NH1 4 0.0040 2.8140 151.9376

SER_128@O ARG_127@HH21 ARG_127@NH2 4 0.0040 2.8141 161.8756

SER_291@O GLY_293@H GLY_293@N 4 0.0040 2.8141 147.2884

ASP_421@OD2 GLN_420@HE22 GLN_420@NE2 4 0.0040 2.8159 156.9649

GLU_310@O ARG_210@HH11 ARG_210@NH1 4 0.0040 2.8178 160.9206

GLU_226@OE2 THR_292@HG1 THR_292@OG1 4 0.0040 2.8180 166.3977

SER_283@O ARG_305@HH21 ARG_305@NH2 4 0.0040 2.8182 155.3356

GLN_117@OE1 ASN_115@HD21 ASN_115@ND2 4 0.0040 2.8198 159.6625

ASN_115@OD1 GLN_117@HE21 GLN_117@NE2 4 0.0040 2.8222 161.3528

GLU_310@OE2 ASN_379@HD21 ASN_379@ND2 4 0.0040 2.8222 171.2286

GLU_351@OE1 ARG_205@HE ARG_205@NE 4 0.0040 2.8222 147.7049

ASP_311@OD1 ARG_210@HH22 ARG_210@NH2 4 0.0040 2.8231 146.2459

ASP_380@OD1 ASP_380@H ASP_380@N 4 0.0040 2.8276 138.4313

SER_27@OG VAL_28@H VAL_28@N 4 0.0040 2.8279 141.9766

THR_4@OG1 SER_25@HG SER_25@OG 4 0.0040 2.8282 161.4914

GLN_37@OE1 GLN_233@HE22 GLN_233@NE2 4 0.0040 2.8325 160.1440

ASN_23@OD1 SER_25@HG SER_25@OG 4 0.0040 2.8353 152.7933

GLU_374@OE2 GLN_375@HE22 GLN_375@NE2 4 0.0040 2.8366 169.5167

SER_151@O VAL_153@H VAL_153@N 4 0.0040 2.8383 151.5490

SER_69@OG LYS_67@HZ1 LYS_67@NZ 4 0.0040 2.8399 151.8153

GLU_218@OE2 SER_203@H SER_203@N 4 0.0040 2.8401 154.3169

GLN_147@OE1 LYS_149@HZ3 LYS_149@NZ 4 0.0040 2.8410 158.5494

GLU_265@O LYS_249@HZ1 LYS_249@NZ 4 0.0040 2.8413 150.7485

ASP_255@OD2 ASP_255@H ASP_255@N 4 0.0040 2.8416 139.5064

ASN_415@O LYS_209@HZ1 LYS_209@NZ 4 0.0040 2.8451 150.4702

SER_69@OG LYS_67@HZ3 LYS_67@NZ 4 0.0040 2.8468 146.1110

ASP_157@OD1 GLN_147@H GLN_147@N 4 0.0040 2.8475 157.5171

ASN_118@OD1 GLN_117@HE22 GLN_117@NE2 4 0.0040 2.8482 164.2570

SER_25@O GLN_1@H1 GLN_1@N 4 0.0040 2.8483 161.0998

THR_419@OG1 ARG_210@HH22 ARG_210@NH2 4 0.0040 2.8532 142.5348

GLN_117@O GLN_117@HE22 GLN_117@NE2 4 0.0040 2.8611 148.1142

GLU_14@O ALA_80@H ALA_80@N 4 0.0040 2.8633 155.8168

GLY_107@O SER_8@HG SER_8@OG 4 0.0040 2.8646 158.7674

ASP_150@OD2 LYS_149@H LYS_149@N 4 0.0040 2.8704 160.1484

GLN_147@OE1 LYS_149@HZ2 LYS_149@NZ 4 0.0040 2.8727 149.1936

VAL_208@O LYS_209@HZ1 LYS_209@NZ 4 0.0040 2.8734 153.7578

SER_27@O LYS_68@HZ2 LYS_68@NZ 4 0.0040 2.8758 151.9022

THR_279@OG1 ASN_379@HD21 ASN_379@ND2 4 0.0040 2.8797 154.7193

GLN_117@OE1 GLN_117@H GLN_117@N 4 0.0040 2.8823 150.4352

GLN_117@OE1 ASN_118@HD21 ASN_118@ND2 4 0.0040 2.8871 151.8993

THR_419@OG1 GLN_420@HE22 GLN_420@NE2 4 0.0040 2.8903 151.5282

THR_197@O ASN_190@HD22 ASN_190@ND2 4 0.0040 2.8916 150.4965

GLU_327@O ILE_330@H ILE_330@N 4 0.0040 2.8923 142.9312

ASN_379@OD1 LYS_314@HZ3 LYS_314@NZ 4 0.0040 2.8945 157.4548

SER_413@O ASN_415@H ASN_415@N 4 0.0040 2.8958 144.9374

SER_25@OG GLN_1@HE22 GLN_1@NE2 4 0.0040 2.8996 159.4177

SER_276@OG LYS_214@HZ3 LYS_214@NZ 4 0.0040 2.9018 151.8685

PRO_369@O GLN_370@HE22 GLN_370@NE2 4 0.0040 2.9022 153.0889

GLY_436@O ARG_437@HH11 ARG_437@NH1 4 0.0040 2.9022 156.9929

GLY_358@O TRP_355@HE1 TRP_355@NE1 4 0.0040 2.9035 137.3710

SER_172@OG THR_159@HG1 THR_159@OG1 4 0.0040 2.9062 140.6955

SER_51@OG THR_50@H THR_50@N 4 0.0040 2.9064 155.2230

TRP_355@O GLY_358@H GLY_358@N 4 0.0040 2.9107 157.8553

ASP_181@O ASN_186@HD22 ASN_186@ND2 4 0.0040 2.9135 158.2519

GLN_142@OE1 GLU_195@H GLU_195@N 4 0.0040 2.9211 151.9520

SER_146@O SER_146@HG SER_146@OG 4 0.0040 2.9228 138.3568

THR_4@OG1 GLN_1@HE21 GLN_1@NE2 4 0.0040 2.9237 162.3654

LYS_131@O ASP_130@H ASP_130@N 4 0.0040 2.9289 164.8867

SER_11@OG LYS_158@HZ1 LYS_158@NZ 4 0.0040 2.9304 143.5683

VAL_92@O THR_103@HG1 THR_103@OG1 4 0.0040 2.9326 150.4134

ASN_118@O ASP_120@H ASP_120@N 4 0.0040 2.9333 142.8946

SER_291@OG GLU_226@H GLU_226@N 4 0.0040 2.9460 162.7872

ASN_40@ND2 ARG_305@HE ARG_305@NE 4 0.0040 2.9477 158.1069

THR_211@OG1 ARG_210@HH11 ARG_210@NH1 4 0.0040 2.9481 159.5905

SER_13@OG GLY_15@H GLY_15@N 4 0.0040 2.9572 164.5988

GLN_420@NE2 ARG_422@HE ARG_422@NE 4 0.0040 2.9591 158.9001

THR_343@O PHE_345@H PHE_345@N 4 0.0040 2.9597 138.9219

ASP_138@OD2 TYR_124@HH TYR_124@OH 3 0.0030 2.6804 157.4349

ASP_150@OD1 SER_151@HG SER_151@OG 3 0.0030 2.6882 158.6631

ASP_120@OD2 THR_197@HG1 THR_197@OG1 3 0.0030 2.7003 168.0380

ALA_183@O ALA_185@H ALA_185@N 3 0.0030 2.7127 146.1988

GLU_319@OE1 THR_343@HG1 THR_343@OG1 3 0.0030 2.7193 161.2843

ALA_328@O SER_331@HG SER_331@OG 3 0.0030 2.7237 159.4529

GLU_213@OE1 ARG_210@HH22 ARG_210@NH2 3 0.0030 2.7259 142.7994

ALA_16@O SER_79@H SER_79@N 3 0.0030 2.7483 141.8793

SER_291@O SER_290@HG SER_290@OG 3 0.0030 2.7553 156.4136

HIE_225@O LYS_267@HZ2 LYS_267@NZ 3 0.0030 2.7557 153.2485

SER_26@OG SER_27@H SER_27@N 3 0.0030 2.7570 138.9585

GLU_258@OE1 LYS_253@HZ1 LYS_253@NZ 3 0.0030 2.7609 148.2928

SER_381@O LYS_373@HZ3 LYS_373@NZ 3 0.0030 2.7616 145.5272

GLU_258@OE1 LYS_253@HZ3 LYS_253@NZ 3 0.0030 2.7620 159.5890

SER_366@O ARG_388@HE ARG_388@NE 3 0.0030 2.7625 145.2804

GLU_360@OE1 LYS_359@HZ3 LYS_359@NZ 3 0.0030 2.7825 154.1907

ASN_115@O ASN_115@HD22 ASN_115@ND2 3 0.0030 2.7933 153.8002

ASN_379@O LYS_314@HZ2 LYS_314@NZ 3 0.0030 2.7978 147.5417

ASP_84@OD2 ASN_60@HD22 ASN_60@ND2 3 0.0030 2.7980 157.1674

GLN_370@O GLN_147@HE22 GLN_147@NE2 3 0.0030 2.8023 159.0569

GLU_324@OE1 ARG_437@HH21 ARG_437@NH2 3 0.0030 2.8036 156.5433

ASN_190@OD1 SER_191@H SER_191@N 3 0.0030 2.8055 143.3589

LYS_179@O SER_177@HG SER_177@OG 3 0.0030 2.8070 155.5497

GLN_334@O SER_392@HG SER_392@OG 3 0.0030 2.8077 141.3013

ASN_190@O ASN_190@HD22 ASN_190@ND2 3 0.0030 2.8089 146.7897

THR_307@OG1 ARG_305@HH21 ARG_305@NH2 3 0.0030 2.8102 150.2863

ASN_415@O ASN_415@HD22 ASN_415@ND2 3 0.0030 2.8118 147.9423

ASP_150@OD2 SER_151@HG SER_151@OG 3 0.0030 2.8121 154.7966

ASP_157@OD1 HIE_9@HE2 HIE_9@NE2 3 0.0030 2.8127 154.4649

SER_93@O GLN_1@HE22 GLN_1@NE2 3 0.0030 2.8133 151.0496

ASP_222@OD1 ASP_222@H ASP_222@N 3 0.0030 2.8156 140.4599

ASN_415@OD1 ASN_415@H ASN_415@N 3 0.0030 2.8164 139.5447

ASP_167@OD1 LYS_169@HZ2 LYS_169@NZ 3 0.0030 2.8171 156.7088

GLU_360@OE2 LYS_359@HZ1 LYS_359@NZ 3 0.0030 2.8178 160.5033

ASP_181@OD2 PHE_182@H PHE_182@N 3 0.0030 2.8206 142.9261

LYS_131@O SER_129@HG SER_129@OG 3 0.0030 2.8215 143.9684

PHE_198@OXT HIE_332@HE2 HIE_332@NE2 3 0.0030 2.8218 168.9006

GLU_195@OE2 GLU_195@H GLU_195@N 3 0.0030 2.8247 147.0903

ASP_222@OD2 ASP_222@H ASP_222@N 3 0.0030 2.8277 140.0576

ASP_311@OD1 LYS_314@HZ2 LYS_314@NZ 3 0.0030 2.8288 158.4088

ILE_273@O SER_261@HG SER_261@OG 3 0.0030 2.8293 153.4583

GLU_213@OE1 LYS_209@HZ2 LYS_209@NZ 3 0.0030 2.8293 158.5050

GLN_375@OE1 SER_381@HG SER_381@OG 3 0.0030 2.8298 161.5939

ASN_60@OD1 LYS_77@HZ3 LYS_77@NZ 3 0.0030 2.8309 145.9448

TYR_296@OH THR_299@HG1 THR_299@OG1 3 0.0030 2.8313 164.6554

ASP_152@OD2 ASP_152@H ASP_152@N 3 0.0030 2.8328 141.1394

ASP_140@O LYS_158@HZ3 LYS_158@NZ 3 0.0030 2.8336 165.3296

ASP_130@O SER_132@H SER_132@N 3 0.0030 2.8344 137.8701

ASP_130@OD1 ASP_130@H ASP_130@N 3 0.0030 2.8362 136.2034

GLU_218@OE1 SER_203@H SER_203@N 3 0.0030 2.8418 161.2385

GLU_265@OE1 LYS_266@HZ1 LYS_266@NZ 3 0.0030 2.8493 156.4736

ASN_357@O LYS_359@HZ3 LYS_359@NZ 3 0.0030 2.8515 157.2852

ASN_379@OD1 ASN_379@H ASN_379@N 3 0.0030 2.8524 137.2737

ASN_357@O LYS_359@HZ2 LYS_359@NZ 3 0.0030 2.8548 153.0943

LEU_55@O LYS_57@HZ1 LYS_57@NZ 3 0.0030 2.8554 162.5748

HIE_225@O LYS_267@HZ3 LYS_267@NZ 3 0.0030 2.8566 148.0545

PRO_376@O LYS_373@HZ3 LYS_373@NZ 3 0.0030 2.8571 138.9489

GLU_252@OE1 LYS_253@HZ2 LYS_253@NZ 3 0.0030 2.8601 163.9582

SER_384@OG TRP_176@HE1 TRP_176@NE1 3 0.0030 2.8623 147.4451

THR_307@O THR_307@HG1 THR_307@OG1 3 0.0030 2.8633 142.3135

VAL_262@O SER_263@HG SER_263@OG 3 0.0030 2.8636 151.7455

GLN_370@OE1 ARG_109@HH12 ARG_109@NH1 3 0.0030 2.8661 155.5760

GLU_351@OE2 ARG_205@HH21 ARG_205@NH2 3 0.0030 2.8706 151.5956

TYR_38@O GLN_37@HE22 GLN_37@NE2 3 0.0030 2.8741 160.7250

TYR_231@OH TYR_298@HH TYR_298@OH 3 0.0030 2.8748 146.9185

SER_353@OG TRP_354@H TRP_354@N 3 0.0030 2.8776 138.3537

SER_302@OG THR_201@HG1 THR_201@OG1 3 0.0030 2.8781 140.7810

THR_50@OG1 LYS_48@HZ1 LYS_48@NZ 3 0.0030 2.8820 151.7005

ARG_305@O THR_304@HG1 THR_304@OG1 3 0.0030 2.8871 168.0052

GLU_14@OE2 ASN_115@HD21 ASN_115@ND2 3 0.0030 2.8873 148.5843

ASP_224@O LYS_267@HZ1 LYS_267@NZ 3 0.0030 2.8875 138.9644

GLU_265@O LYS_249@HZ2 LYS_249@NZ 3 0.0030 2.8890 155.3278

SER_79@OG HIE_81@HE2 HIE_81@NE2 3 0.0030 2.8905 146.6880

GLN_117@OE1 ASN_118@HD22 ASN_118@ND2 3 0.0030 2.8955 166.5205

GLY_42@O TYR_38@H TYR_38@N 3 0.0030 2.8962 153.0435

THR_103@OG1 GLN_1@HE22 GLN_1@NE2 3 0.0030 2.8973 144.0772

ASP_380@OD1 SER_381@H SER_381@N 3 0.0030 2.8986 147.2968

GLN_41@OE1 TYR_38@H TYR_38@N 3 0.0030 2.9058 168.5343

SER_93@O TYR_31@H TYR_31@N 3 0.0030 2.9087 161.8033

SER_261@OG LYS_251@HZ1 LYS_251@NZ 3 0.0030 2.9087 159.3439

LYS_131@O ARG_127@HE ARG_127@NE 3 0.0030 2.9140 141.6593

LEU_102@O THR_103@HG1 THR_103@OG1 3 0.0030 2.9144 150.1945

ASN_190@O ILE_193@H ILE_193@N 3 0.0030 2.9161 163.8320

GLU_374@OE1 ARG_382@HH11 ARG_382@NH1 3 0.0030 2.9194 156.9360

THR_394@O ASN_398@HD22 ASN_398@ND2 3 0.0030 2.9196 168.2316

TYR_383@OH LYS_373@HZ3 LYS_373@NZ 3 0.0030 2.9225 145.8661

ASP_255@OD1 ASP_255@H ASP_255@N 3 0.0030 2.9235 137.2919

SER_26@OG GLN_1@HE21 GLN_1@NE2 3 0.0030 2.9239 160.8577

ASN_313@OD1 THR_419@H THR_419@N 3 0.0030 2.9244 151.3524

TYR_243@OH LYS_251@HZ3 LYS_251@NZ 3 0.0030 2.9261 149.3071

SER_172@O SER_172@HG SER_172@OG 3 0.0030 2.9286 139.3494

SER_129@OG ARG_127@HE ARG_127@NE 3 0.0030 2.9338 161.0814

HIE_225@NE2 SER_289@HG SER_289@OG 3 0.0030 2.9351 142.6187

ASN_99@O ASN_99@HD22 ASN_99@ND2 3 0.0030 2.9379 143.6976

SER_128@O LYS_131@H LYS_131@N 3 0.0030 2.9402 168.0642

LYS_169@O GLN_117@H GLN_117@N 3 0.0030 2.9413 152.5712

THR_103@O THR_103@HG1 THR_103@OG1 3 0.0030 2.9426 146.0225

SER_25@O GLN_1@H3 GLN_1@N 3 0.0030 2.9434 144.0944

ASN_280@ND2 ASN_280@H ASN_280@N 3 0.0030 2.9464 139.3361

PHE_97@O TYR_296@H TYR_296@N 3 0.0030 2.9493 160.8822

ASN_115@O GLN_117@HE21 GLN_117@NE2 3 0.0030 2.9525 146.3909

LEU_110@O ARG_109@HE ARG_109@NE 3 0.0030 2.9532 147.4380

GLN_408@OE1 TYR_410@HH TYR_410@OH 2 0.0020 2.6243 154.9788

SER_2@O SER_2@HG SER_2@OG 2 0.0020 2.6688 139.2423

THR_103@O SER_2@HG SER_2@OG 2 0.0020 2.6701 167.1646

GLU_319@OE2 THR_343@HG1 THR_343@OG1 2 0.0020 2.6703 161.5525

ASN_171@O SER_170@HG SER_170@OG 2 0.0020 2.6791 163.2649

GLU_70@OE2 ARG_21@HH21 ARG_21@NH2 2 0.0020 2.6821 142.8114

ASP_162@OD2 LYS_169@HZ3 LYS_169@NZ 2 0.0020 2.6828 149.3016

ASP_224@O LYS_267@HZ2 LYS_267@NZ 2 0.0020 2.6967 151.2533

GLU_310@O ARG_210@HH12 ARG_210@NH1 2 0.0020 2.7160 153.6400

GLU_213@OE1 LYS_214@HZ1 LYS_214@NZ 2 0.0020 2.7183 150.2368

SER_180@O SER_180@HG SER_180@OG 2 0.0020 2.7210 137.9332

SER_191@O SER_191@HG SER_191@OG 2 0.0020 2.7272 149.6804

ASP_311@OD2 LYS_314@HZ2 LYS_314@NZ 2 0.0020 2.7292 149.5814

ASN_415@OD1 SER_413@HG SER_413@OG 2 0.0020 2.7302 144.7507

GLU_327@OE1 TRP_396@HE1 TRP_396@NE1 2 0.0020 2.7396 168.8947

GLU_218@O SER_203@HG SER_203@OG 2 0.0020 2.7399 172.2718

GLU_319@OE2 LYS_131@HZ3 LYS_131@NZ 2 0.0020 2.7420 163.0595

ASP_140@OD2 ASP_140@H ASP_140@N 2 0.0020 2.7429 136.2868

GLY_254@O ILE_256@H ILE_256@N 2 0.0020 2.7430 136.6677

ASP_255@OD2 LYS_101@HZ1 LYS_101@NZ 2 0.0020 2.7474 159.3615

GLU_252@OE1 TYR_246@HH TYR_246@OH 2 0.0020 2.7476 154.2750

GLN_202@O THR_201@HG1 THR_201@OG1 2 0.0020 2.7478 163.3277

GLU_252@OE2 TYR_246@HH TYR_246@OH 2 0.0020 2.7487 160.7050

GLU_433@OE2 TRP_435@HE1 TRP_435@NE1 2 0.0020 2.7499 174.6266

SER_431@OG ARG_404@HH12 ARG_404@NH1 2 0.0020 2.7506 148.9194

GLU_14@OE2 MET_82@H MET_82@N 2 0.0020 2.7519 147.0740

GLU_265@OE1 ARG_269@HH22 ARG_269@NH2 2 0.0020 2.7562 152.4268

ALA_185@O ASN_189@HD21 ASN_189@ND2 2 0.0020 2.7568 163.9904

ASP_255@OD2 GLN_1@H1 GLN_1@N 2 0.0020 2.7603 170.2221

ALA_187@O ASN_189@HD22 ASN_189@ND2 2 0.0020 2.7656 161.3967

GLU_252@OE1 LYS_253@HZ1 LYS_253@NZ 2 0.0020 2.7662 145.4393

ILE_256@O LYS_253@HZ2 LYS_253@NZ 2 0.0020 2.7692 154.6176

SER_381@O LYS_373@HZ1 LYS_373@NZ 2 0.0020 2.7725 145.2612

ASP_138@OD1 LYS_335@HZ3 LYS_335@NZ 2 0.0020 2.7749 149.6971

ASN_379@OD1 LYS_314@HZ2 LYS_314@NZ 2 0.0020 2.7780 149.0275

GLU_374@OE1 LYS_131@HZ1 LYS_131@NZ 2 0.0020 2.7796 160.5470

GLU_319@OE1 LYS_131@HZ1 LYS_131@NZ 2 0.0020 2.7803 157.9790

SER_146@OG LYS_149@HZ2 LYS_149@NZ 2 0.0020 2.7815 166.6137

LYS_68@O THR_71@HG1 THR_71@OG1 2 0.0020 2.7843 166.9006

LEU_274@O LYS_214@HZ2 LYS_214@NZ 2 0.0020 2.7871 158.8527

ARG_232@O ARG_240@HH21 ARG_240@NH2 2 0.0020 2.7883 146.5762

ASP_140@OD1 SER_141@H SER_141@N 2 0.0020 2.7889 144.2065

ASP_380@OD2 LYS_373@HZ1 LYS_373@NZ 2 0.0020 2.7899 155.5787

SER_11@OG THR_111@HG1 THR_111@OG1 2 0.0020 2.7905 145.8724

ASN_144@OD1 LYS_158@HZ1 LYS_158@NZ 2 0.0020 2.7943 156.7079

GLU_329@OE2 THR_337@H THR_337@N 2 0.0020 2.7975 145.5160

ASP_138@OD1 LYS_335@HZ2 LYS_335@NZ 2 0.0020 2.7998 153.7651

GLU_195@OE2 ASN_190@H ASN_190@N 2 0.0020 2.8002 148.3336

SER_128@O SER_128@HG SER_128@OG 2 0.0020 2.8033 137.9897

SER_26@O VAL_28@H VAL_28@N 2 0.0020 2.8091 143.8533

GLU_70@OE2 HIE_74@HE2 HIE_74@NE2 2 0.0020 2.8099 163.3713

SER_11@OG LYS_158@HZ3 LYS_158@NZ 2 0.0020 2.8119 156.9114

GLY_238@O ARG_240@H ARG_240@N 2 0.0020 2.8131 143.4328

SER_2@OG SER_25@HG SER_25@OG 2 0.0020 2.8132 143.0513

SER_413@OG GLU_414@H GLU_414@N 2 0.0020 2.8140 137.9126

THR_304@OG1 SER_204@HG SER_204@OG 2 0.0020 2.8165 148.0528

SER_387@OG ARG_388@H ARG_388@N 2 0.0020 2.8190 140.3244

GLY_42@O GLN_41@HE22 GLN_41@NE2 2 0.0020 2.8190 150.5243

THR_343@OG1 LYS_131@HZ3 LYS_131@NZ 2 0.0020 2.8196 157.5591

VAL_208@O LYS_209@HZ2 LYS_209@NZ 2 0.0020 2.8232 156.2744

ASP_311@OD1 ARG_210@HE ARG_210@NE 2 0.0020 2.8274 145.1162

SER_146@OG LYS_149@HZ1 LYS_149@NZ 2 0.0020 2.8286 143.8027

SER_326@OG GLU_327@H GLU_327@N 2 0.0020 2.8309 136.0258

ASP_196@OD2 GLN_142@HE21 GLN_142@NE2 2 0.0020 2.8313 157.7617

GLU_218@OE1 ARG_269@HH22 ARG_269@NH2 2 0.0020 2.8325 152.8612

GLU_433@OE1 TRP_435@HE1 TRP_435@NE1 2 0.0020 2.8348 171.8798

TYR_298@OH LYS_101@H LYS_101@N 2 0.0020 2.8390 136.1568

PRO_371@O GLN_370@HE21 GLN_370@NE2 2 0.0020 2.8431 147.2793

SER_128@OG SER_180@HG SER_180@OG 2 0.0020 2.8457 150.1318

LEU_306@O THR_307@HG1 THR_307@OG1 2 0.0020 2.8467 150.8040

GLN_1@OE1 GLN_1@H2 GLN_1@N 2 0.0020 2.8488 138.6582

ARG_205@O ARG_305@HH21 ARG_305@NH2 2 0.0020 2.8491 162.2094

GLU_265@O LYS_249@HZ3 LYS_249@NZ 2 0.0020 2.8492 162.2457

SER_27@O LYS_68@HZ1 LYS_68@NZ 2 0.0020 2.8496 163.4771

GLN_41@OE1 LYS_199@HZ2 LYS_199@NZ 2 0.0020 2.8502 147.1259

ASN_40@OD1 ARG_205@HH12 ARG_205@NH1 2 0.0020 2.8518 139.7903

PHE_66@O THR_54@HG1 THR_54@OG1 2 0.0020 2.8521 164.0883

GLU_374@OE1 LYS_131@HZ2 LYS_131@NZ 2 0.0020 2.8527 144.0681

ASP_138@N THR_137@HG1 THR_137@OG1 2 0.0020 2.8543 136.8898

ALA_52@O THR_54@HG1 THR_54@OG1 2 0.0020 2.8553 157.2302

SER_129@O SER_129@HG SER_129@OG 2 0.0020 2.8554 136.2142

GLN_1@OE1 GLN_1@H3 GLN_1@N 2 0.0020 2.8555 136.5740

LYS_424@O GLN_428@HE21 GLN_428@NE2 2 0.0020 2.8559 146.3459

SER_141@OG GLN_142@HE21 GLN_142@NE2 2 0.0020 2.8565 161.6932

GLY_436@O ARG_437@HE ARG_437@NE 2 0.0020 2.8572 162.2240

GLU_310@OE2 ASP_311@H ASP_311@N 2 0.0020 2.8572 156.5433

PRO_119@O ASN_118@HD22 ASN_118@ND2 2 0.0020 2.8574 147.1627

ASN_60@OD1 LYS_77@HZ1 LYS_77@NZ 2 0.0020 2.8583 138.3468

VAL_200@O LYS_199@HZ1 LYS_199@NZ 2 0.0020 2.8605 154.5067

ASN_379@O LYS_314@HZ3 LYS_314@NZ 2 0.0020 2.8617 154.9896

SER_25@OG GLN_1@H2 GLN_1@N 2 0.0020 2.8619 152.3288

GLU_87@OE1 GLN_37@HE22 GLN_37@NE2 2 0.0020 2.8636 155.3899

GLY_58@O LYS_57@HZ3 LYS_57@NZ 2 0.0020 2.8638 154.0616

TYR_296@OH GLN_44@HE21 GLN_44@NE2 2 0.0020 2.8676 160.2840

LEU_274@O LYS_214@HZ1 LYS_214@NZ 2 0.0020 2.8696 144.6124

ARG_164@O MET_166@H MET_166@N 2 0.0020 2.8698 153.0956

ARG_404@O GLN_406@HE21 GLN_406@NE2 2 0.0020 2.8720 160.1033

ALA_393@O PHE_395@H PHE_395@N 2 0.0020 2.8736 155.0816

HIE_332@O GLN_334@HE21 GLN_334@NE2 2 0.0020 2.8739 153.6075

SER_302@OG ARG_205@HH21 ARG_205@NH2 2 0.0020 2.8741 149.3301

ASN_118@O ASN_118@HD22 ASN_118@ND2 2 0.0020 2.8752 140.9745

ASN_99@OD1 ASN_99@H ASN_99@N 2 0.0020 2.8762 136.9752

ASN_178@OD1 LYS_179@H LYS_179@N 2 0.0020 2.8774 143.7353

SER_302@O CYX_287@H CYX_287@N 2 0.0020 2.8798 156.4899

SER_69@OG GLU_70@H GLU_70@N 2 0.0020 2.8808 139.6089

ASP_181@OD1 PHE_182@H PHE_182@N 2 0.0020 2.8819 141.9821

ASN_178@O LYS_179@HZ2 LYS_179@NZ 2 0.0020 2.8869 160.1694

ASP_421@OD2 GLN_420@HE21 GLN_420@NE2 2 0.0020 2.8873 154.0149

GLY_105@O GLY_107@H GLY_107@N 2 0.0020 2.8887 137.2545

ASN_379@OD1 ASP_380@H ASP_380@N 2 0.0020 2.8896 143.3597

THR_4@OG1 GLN_5@H GLN_5@N 2 0.0020 2.8898 140.7867

SER_386@O ARG_388@HH21 ARG_388@NH2 2 0.0020 2.8923 154.1602

THR_159@O THR_159@HG1 THR_159@OG1 2 0.0020 2.8939 143.3012

GLY_58@O PHE_62@H PHE_62@N 2 0.0020 2.8945 156.7493

HIE_402@ND1 PHE_403@H PHE_403@N 2 0.0020 2.8956 135.4856

ASN_190@OD1 ASN_189@HD22 ASN_189@ND2 2 0.0020 2.8975 150.0491

LYS_158@O LYS_158@HZ2 LYS_158@NZ 2 0.0020 2.8989 145.3899

GLU_265@O LYS_266@HZ1 LYS_266@NZ 2 0.0020 2.9007 142.9958

GLU_226@OE2 THR_292@H THR_292@N 2 0.0020 2.9017 170.4954

ASP_196@OD2 ASN_190@HD21 ASN_190@ND2 2 0.0020 2.9017 161.1759

ASN_280@ND2 SER_278@HG SER_278@OG 2 0.0020 2.9042 158.8099

ASP_368@OD2 ARG_388@HH11 ARG_388@NH1 2 0.0020 2.9046 155.9635

SER_302@OG ARG_205@HH11 ARG_205@NH1 2 0.0020 2.9058 148.0282

ASN_186@O ASN_189@HD21 ASN_189@ND2 2 0.0020 2.9080 153.1082

ILE_330@O THR_333@H THR_333@N 2 0.0020 2.9108 151.6747

SER_366@O ARG_388@HH21 ARG_388@NH2 2 0.0020 2.9113 153.7879

ASP_140@O THR_143@HG1 THR_143@OG1 2 0.0020 2.9132 154.3526

TYR_124@OH THR_333@HG1 THR_333@OG1 2 0.0020 2.9140 139.7835

VAL_322@O ARG_127@HH21 ARG_127@NH2 2 0.0020 2.9158 152.6338

ASP_368@O GLN_370@H GLN_370@N 2 0.0020 2.9158 141.3646

SER_172@O ARG_388@HH21 ARG_388@NH2 2 0.0020 2.9158 166.1433

ASN_99@ND2 GLY_293@H GLY_293@N 2 0.0020 2.9172 147.7957

GLU_351@OE2 LEU_352@H LEU_352@N 2 0.0020 2.9180 151.8334

SER_8@O ARG_109@HH21 ARG_109@NH2 2 0.0020 2.9183 149.1502

ASP_380@O ARG_382@HH21 ARG_382@NH2 2 0.0020 2.9206 149.2266

SER_283@OG ARG_232@HH21 ARG_232@NH2 2 0.0020 2.9243 137.4478

GLU_195@O PHE_198@H PHE_198@N 2 0.0020 2.9283 156.6377

GLU_374@O GLN_375@HE22 GLN_375@NE2 2 0.0020 2.9286 152.8886

TYR_383@OH LYS_373@HZ2 LYS_373@NZ 2 0.0020 2.9292 141.1019

ASP_130@OD2 ARG_127@HE ARG_127@NE 2 0.0020 2.9294 155.3805

HIE_362@ND1 HIE_362@H HIE_362@N 2 0.0020 2.9310 144.0607

THR_103@O GLN_1@HE21 GLN_1@NE2 2 0.0020 2.9326 154.3784

ASP_157@OD2 GLN_147@H GLN_147@N 2 0.0020 2.9329 163.2711

SER_83@OG ASN_60@HD21 ASN_60@ND2 2 0.0020 2.9346 145.7949

THR_394@OG1 GLN_334@HE21 GLN_334@NE2 2 0.0020 2.9362 155.5182

ASP_196@OD2 PHE_198@H PHE_198@N 2 0.0020 2.9386 149.3372

GLN_420@NE2 ASN_313@HD21 ASN_313@ND2 2 0.0020 2.9390 144.3782

HIE_9@ND1 HIE_9@H HIE_9@N 2 0.0020 2.9407 140.0380

ASP_255@OD1 GLN_1@HE21 GLN_1@NE2 2 0.0020 2.9423 155.7281

SER_431@OG ARG_404@HE ARG_404@NE 2 0.0020 2.9432 160.6823

ASN_280@O THR_282@H THR_282@N 2 0.0020 2.9460 146.9390

ASP_421@OD1 ARG_422@HE ARG_422@NE 2 0.0020 2.9504 140.9410

TYR_35@OH TYR_298@H TYR_298@N 2 0.0020 2.9556 163.3559

TYR_88@OH TYR_38@HH TYR_38@OH 2 0.0020 2.9563 144.1609

SER_384@O SER_384@HG SER_384@OG 2 0.0020 2.9565 141.4014

GLN_1@OE1 GLN_1@H1 GLN_1@N 2 0.0020 2.9582 147.3705

SER_366@OG ARG_388@HH12 ARG_388@NH1 2 0.0020 2.9598 172.8559

GLN_125@OE1 ARG_127@H ARG_127@N 2 0.0020 2.9648 161.7460

HIE_402@O ARG_404@H ARG_404@N 2 0.0020 2.9650 146.9100

PRO_399@O ASN_401@H ASN_401@N 2 0.0020 2.9692 143.6005

HIE_349@ND1 ARG_305@HH11 ARG_305@NH1 2 0.0020 2.9734 154.8736

TYR_260@OH GLN_281@HE21 GLN_281@NE2 2 0.0020 2.9740 165.2912

ASP_196@OD1 PHE_198@H PHE_198@N 2 0.0020 2.9777 154.2831

HIE_9@O SER_8@HG SER_8@OG 2 0.0020 2.9798 167.1423

SER_278@OG GLN_281@H GLN_281@N 2 0.0020 2.9813 147.3186

SER_151@OG GLN_375@HE22 GLN_375@NE2 2 0.0020 2.9827 159.4595

SER_11@OG HIE_9@HE2 HIE_9@NE2 2 0.0020 2.9837 164.3885

TRP_355@NE1 GLN_406@HE21 GLN_406@NE2 2 0.0020 2.9884 153.9567

GLN_147@NE2 GLN_370@HE22 GLN_370@NE2 2 0.0020 2.9949 153.5140

GLN_1@OE1 SER_27@HG SER_27@OG 1 0.0010 2.5278 164.2595

THR_103@OG1 SER_2@HG SER_2@OG 1 0.0010 2.5379 168.0969

ASP_181@OD2 SER_180@HG SER_180@OG 1 0.0010 2.5441 173.4211

ASP_150@OD2 SER_148@HG SER_148@OG 1 0.0010 2.5561 168.1653

SER_8@O SER_8@HG SER_8@OG 1 0.0010 2.5696 143.9912

PHE_182@O SER_128@HG SER_128@OG 1 0.0010 2.6125 167.9478

ASP_152@O LYS_179@HZ2 LYS_179@NZ 1 0.0010 2.6139 154.2688

GLU_329@OE2 TYR_124@HH TYR_124@OH 1 0.0010 2.6178 160.2441

GLN_281@OE1 TYR_260@HH TYR_260@OH 1 0.0010 2.6220 160.1451

ASP_140@OD2 ASN_115@HD22 ASN_115@ND2 1 0.0010 2.6362 153.8470

ASP_368@OD1 SER_386@HG SER_386@OG 1 0.0010 2.6390 152.4106

GLU_265@OE1 ARG_269@HH21 ARG_269@NH2 1 0.0010 2.6401 158.8805

GLY_254@O LYS_253@HZ3 LYS_253@NZ 1 0.0010 2.6416 150.3686

HIE_81@O SER_83@H SER_83@N 1 0.0010 2.6436 152.2535

GLU_417@OE1 ARG_210@HH11 ARG_210@NH1 1 0.0010 2.6699 166.1887

SER_2@OG SER_26@HG SER_26@OG 1 0.0010 2.6772 161.4755

GLU_195@O THR_197@HG1 THR_197@OG1 1 0.0010 2.6878 175.8507

LEU_207@O THR_307@HG1 THR_307@OG1 1 0.0010 2.6881 159.1759

THR_367@O THR_159@HG1 THR_159@OG1 1 0.0010 2.6922 159.3836

ASN_40@O LYS_199@HZ3 LYS_199@NZ 1 0.0010 2.6949 135.9047

ASN_190@O ASN_189@HD22 ASN_189@ND2 1 0.0010 2.6953 142.0914

SER_331@O SER_331@HG SER_331@OG 1 0.0010 2.6957 141.7073

GLU_218@OE1 ARG_269@HH12 ARG_269@NH1 1 0.0010 2.6968 154.3231

GLU_213@OE1 LYS_209@HZ3 LYS_209@NZ 1 0.0010 2.6998 144.8978

GLN_1@OE1 LYS_101@HZ3 LYS_101@NZ 1 0.0010 2.7028 148.6125

PRO_371@O LYS_373@HZ1 LYS_373@NZ 1 0.0010 2.7031 162.6058

ASP_380@OD1 LYS_373@HZ1 LYS_373@NZ 1 0.0010 2.7035 161.2203

SER_271@O SER_271@HG SER_271@OG 1 0.0010 2.7040 144.8535

GLN_125@NE2 SER_128@HG SER_128@OG 1 0.0010 2.7044 148.8373

ASP_196@OD1 GLN_142@HE21 GLN_142@NE2 1 0.0010 2.7105 166.7802

GLU_87@OE1 ARG_109@HH22 ARG_109@NH2 1 0.0010 2.7120 151.6842

SER_128@O ASP_130@H ASP_130@N 1 0.0010 2.7120 146.0531

ASP_255@OD1 THR_103@HG1 THR_103@OG1 1 0.0010 2.7137 157.0936

GLU_374@OE2 TRP_176@HE1 TRP_176@NE1 1 0.0010 2.7158 148.7896

GLU_94@OE2 GLN_1@H2 GLN_1@N 1 0.0010 2.7193 148.2198

ASP_380@OD1 LYS_373@HZ3 LYS_373@NZ 1 0.0010 2.7253 152.4411

GLU_65@O SER_72@HG SER_72@OG 1 0.0010 2.7263 160.2568

SER_148@O ASP_150@H ASP_150@N 1 0.0010 2.7315 147.9588

GLU_94@OE1 GLN_1@H1 GLN_1@N 1 0.0010 2.7327 152.8360

GLY_7@O THR_106@HG1 THR_106@OG1 1 0.0010 2.7343 158.9945

GLU_319@OE2 LYS_131@HZ1 LYS_131@NZ 1 0.0010 2.7346 137.4536

GLU_360@O LYS_359@HZ1 LYS_359@NZ 1 0.0010 2.7377 147.6468

GLU_327@OE2 ARG_437@HH11 ARG_437@NH1 1 0.0010 2.7379 164.8901

ASN_379@OD1 LYS_373@HZ1 LYS_373@NZ 1 0.0010 2.7389 148.4107

LEU_6@O ARG_21@HH22 ARG_21@NH2 1 0.0010 2.7397 153.7651

SER_276@OG ALA_277@H ALA_277@N 1 0.0010 2.7406 137.4376

ASN_40@OD1 GLY_303@H GLY_303@N 1 0.0010 2.7413 162.0075

SER_128@O SER_129@HG SER_129@OG 1 0.0010 2.7424 162.4236

GLU_268@OE2 GLN_221@HE21 GLN_221@NE2 1 0.0010 2.7445 136.8711

ASP_222@OD1 LYS_199@HZ1 LYS_199@NZ 1 0.0010 2.7460 149.9313

SER_129@O SER_132@HG SER_132@OG 1 0.0010 2.7467 136.6142

TRP_354@O SER_353@HG SER_353@OG 1 0.0010 2.7495 144.8144

GLU_275@O ALA_277@H ALA_277@N 1 0.0010 2.7497 141.7844

GLU_70@OE2 GLU_70@H GLU_70@N 1 0.0010 2.7501 141.8761

GLU_268@OE1 GLN_221@HE21 GLN_221@NE2 1 0.0010 2.7511 175.3586

ASP_140@OD2 ASN_115@HD21 ASN_115@ND2 1 0.0010 2.7555 154.7852

ASN_144@OD1 LYS_158@HZ3 LYS_158@NZ 1 0.0010 2.7557 147.9697

ASP_167@OD2 LYS_169@HZ2 LYS_169@NZ 1 0.0010 2.7564 169.3658

ASN_398@OD1 ARG_400@HH21 ARG_400@NH2 1 0.0010 2.7598 143.3144

GLY_297@O THR_299@HG1 THR_299@OG1 1 0.0010 2.7630 153.3860

PHE_300@O LYS_199@HZ3 LYS_199@NZ 1 0.0010 2.7637 154.4635

ASP_167@OD2 LYS_169@HZ3 LYS_169@NZ 1 0.0010 2.7640 135.6549

ASN_186@OD1 LYS_179@HZ2 LYS_179@NZ 1 0.0010 2.7648 154.9628

SER_180@O SER_128@HG SER_128@OG 1 0.0010 2.7673 159.5588

LYS_249@O LYS_249@HZ3 LYS_249@NZ 1 0.0010 2.7686 161.1159

ASP_348@OD2 GLN_370@HE22 GLN_370@NE2 1 0.0010 2.7716 148.1490

ASN_379@OD1 ASN_280@HD21 ASN_280@ND2 1 0.0010 2.7741 166.0146

ASP_140@O LYS_158@HZ2 LYS_158@NZ 1 0.0010 2.7755 172.9728

GLN_375@NE2 SER_151@HG SER_151@OG 1 0.0010 2.7766 174.8793

GLN_420@OE1 ASN_313@HD21 ASN_313@ND2 1 0.0010 2.7790 175.1875

GLU_195@OE2 GLN_142@HE21 GLN_142@NE2 1 0.0010 2.7794 161.8632

ASN_379@O LYS_314@HZ1 LYS_314@NZ 1 0.0010 2.7805 169.2556

SER_245@O ARG_264@HH12 ARG_264@NH1 1 0.0010 2.7813 139.3249

SER_302@OG GLN_202@H GLN_202@N 1 0.0010 2.7819 179.0431

ARG_127@NH2 LYS_131@H LYS_131@N 1 0.0010 2.7835 141.7223

ASN_178@OD1 TRP_176@HE1 TRP_176@NE1 1 0.0010 2.7852 150.4777

GLN_117@OE1 LYS_169@HZ3 LYS_169@NZ 1 0.0010 2.7853 160.2438

ASN_401@OD1 ASN_398@HD22 ASN_398@ND2 1 0.0010 2.7866 162.1175

GLU_319@OE2 LYS_131@HZ2 LYS_131@NZ 1 0.0010 2.7882 157.5525

PRO_325@O ARG_437@HH12 ARG_437@NH1 1 0.0010 2.7904 166.2806

ILE_256@O GLU_258@H GLU_258@N 1 0.0010 2.7905 139.6003

GLN_397@OE1 ARG_437@HH12 ARG_437@NH1 1 0.0010 2.7923 135.2091

GLU_87@OE2 GLN_233@HE22 GLN_233@NE2 1 0.0010 2.7935 136.5081

PRO_376@O LYS_373@HZ2 LYS_373@NZ 1 0.0010 2.7941 144.6407

ASN_401@OD1 ASN_357@HD22 ASN_357@ND2 1 0.0010 2.7947 145.2372

GLU_268@OE1 LYS_267@H LYS_267@N 1 0.0010 2.7951 143.1012

GLU_265@OE1 ARG_269@HH11 ARG_269@NH1 1 0.0010 2.7954 164.8580

GLU_195@O GLN_142@HE22 GLN_142@NE2 1 0.0010 2.7954 153.7800

ASP_380@OD1 LYS_373@HZ2 LYS_373@NZ 1 0.0010 2.7956 171.9730

PHE_198@OXT VAL_123@H VAL_123@N 1 0.0010 2.7957 162.1275

ASP_150@O TYR_154@HH TYR_154@OH 1 0.0010 2.7976 142.5957

VAL_322@O ARG_127@HH12 ARG_127@NH1 1 0.0010 2.8001 153.4156

ASP_150@OD1 LYS_149@HZ3 LYS_149@NZ 1 0.0010 2.8002 151.5857

SER_263@OG ARG_264@H ARG_264@N 1 0.0010 2.8003 145.3406

LEU_352@O SER_353@HG SER_353@OG 1 0.0010 2.8016 157.0631

THR_143@OG1 LYS_158@HZ1 LYS_158@NZ 1 0.0010 2.8033 138.6290

ASP_130@O LYS_131@HZ2 LYS_131@NZ 1 0.0010 2.8052 143.5354

ASP_255@OD2 GLN_1@HE21 GLN_1@NE2 1 0.0010 2.8064 160.5053

SER_146@O SER_148@H SER_148@N 1 0.0010 2.8070 135.0328

ASN_186@OD1 LYS_179@HZ3 LYS_179@NZ 1 0.0010 2.8071 169.2703

PHE_300@O LYS_199@HZ2 LYS_199@NZ 1 0.0010 2.8088 164.2725

ASP_348@OD2 TYR_383@HH TYR_383@OH 1 0.0010 2.8107 136.9063

ASN_40@O ASN_40@HD22 ASN_40@ND2 1 0.0010 2.8108 141.3246

SER_13@OG GLU_14@H GLU_14@N 1 0.0010 2.8115 138.7899

ASP_157@OD2 GLN_147@HE22 GLN_147@NE2 1 0.0010 2.8147 158.1607

GLU_319@OE1 LYS_131@HZ2 LYS_131@NZ 1 0.0010 2.8148 149.9820

THR_54@OG1 LEU_55@H LEU_55@N 1 0.0010 2.8148 137.5455

GLU_417@O ARG_210@HH21 ARG_210@NH2 1 0.0010 2.8165 157.3037

GLN_397@NE2 ARG_437@HE ARG_437@NE 1 0.0010 2.8166 147.4840

ARG_305@O THR_307@HG1 THR_307@OG1 1 0.0010 2.8175 149.2546

THR_143@O THR_143@HG1 THR_143@OG1 1 0.0010 2.8197 146.5354

LYS_158@O THR_159@HG1 THR_159@OG1 1 0.0010 2.8207 149.8710

ASN_186@O ASN_189@H ASN_189@N 1 0.0010 2.8224 152.2098

ASP_181@OD2 ASN_186@HD21 ASN_186@ND2 1 0.0010 2.8225 157.1439

THR_143@OG1 ASN_144@H ASN_144@N 1 0.0010 2.8265 139.0257

ASN_415@O LYS_209@HZ3 LYS_209@NZ 1 0.0010 2.8268 150.6408

GLU_265@OE2 ARG_269@HH21 ARG_269@NH2 1 0.0010 2.8269 167.1507

ALA_438@O ARG_437@HE ARG_437@NE 1 0.0010 2.8271 151.0964

SER_129@OG ARG_127@HH21 ARG_127@NH2 1 0.0010 2.8282 140.3971

ASP_196@OD1 ASN_190@H ASN_190@N 1 0.0010 2.8294 159.2459

GLU_360@O LYS_359@HZ2 LYS_359@NZ 1 0.0010 2.8310 168.5986

ASP_368@OD1 THR_159@HG1 THR_159@OG1 1 0.0010 2.8316 142.3330

GLU_265@OE2 ARG_264@H ARG_264@N 1 0.0010 2.8318 155.8652

GLY_436@O ALA_438@H ALA_438@N 1 0.0010 2.8356 135.1240

GLU_327@OE2 SER_331@HG SER_331@OG 1 0.0010 2.8358 177.0279

ASP_140@O GLN_142@H GLN_142@N 1 0.0010 2.8361 138.5002

ASN_99@OD1 GLY_293@H GLY_293@N 1 0.0010 2.8364 164.6822

LEU_19@O ARG_21@HH12 ARG_21@NH1 1 0.0010 2.8369 157.2856

GLY_61@O LYS_57@HZ2 LYS_57@NZ 1 0.0010 2.8374 144.0729

LYS_251@O LYS_251@HZ1 LYS_251@NZ 1 0.0010 2.8379 155.9312

GLU_87@OE1 ARG_109@H ARG_109@N 1 0.0010 2.8388 137.8884

GLU_195@OE1 ASN_190@H ASN_190@N 1 0.0010 2.8399 151.7479

ASN_144@OD1 SER_146@HG SER_146@OG 1 0.0010 2.8437 164.0699

ASP_311@OD2 ARG_210@HH11 ARG_210@NH1 1 0.0010 2.8447 161.5035

SER_72@OG ASN_23@HD22 ASN_23@ND2 1 0.0010 2.8499 159.4757

ASP_150@O VAL_153@H VAL_153@N 1 0.0010 2.8499 157.5972

TYR_246@O ARG_264@HH12 ARG_264@NH1 1 0.0010 2.8515 141.8859

PHE_62@O LYS_57@HZ2 LYS_57@NZ 1 0.0010 2.8516 164.6059

MET_166@O PHE_168@H PHE_168@N 1 0.0010 2.8522 141.8454

GLU_213@OE2 LYS_214@HZ2 LYS_214@NZ 1 0.0010 2.8536 135.4782

GLU_374@OE1 ARG_382@HE ARG_382@NE 1 0.0010 2.8538 141.7264

GLU_374@OE1 ARG_382@HH21 ARG_382@NH2 1 0.0010 2.8542 154.8723

GLN_1@OE1 TYR_31@H TYR_31@N 1 0.0010 2.8576 164.5363

GLU_310@OE1 ASN_379@HD22 ASN_379@ND2 1 0.0010 2.8584 164.9375

GLU_100@OE1 THR_292@HG1 THR_292@OG1 1 0.0010 2.8595 155.3910

GLY_15@O SER_79@H SER_79@N 1 0.0010 2.8595 136.7818

THR_4@OG1 ASN_23@HD21 ASN_23@ND2 1 0.0010 2.8596 142.1786

ALA_185@O ALA_187@H ALA_187@N 1 0.0010 2.8597 135.2833

ALA_438@O ARG_437@HH21 ARG_437@NH2 1 0.0010 2.8617 163.6674

SER_95@O PHE_97@H PHE_97@N 1 0.0010 2.8623 146.2671

SER_83@O ALA_85@H ALA_85@N 1 0.0010 2.8623 137.3380

SER_165@OG MET_166@H MET_166@N 1 0.0010 2.8630 138.0894

VAL_3@O GLN_5@H GLN_5@N 1 0.0010 2.8641 138.1556

ASP_368@OD1 THR_156@HG1 THR_156@OG1 1 0.0010 2.8646 149.1983

GLN_334@O LYS_335@HZ1 LYS_335@NZ 1 0.0010 2.8653 151.3041

ASN_60@O PHE_62@H PHE_62@N 1 0.0010 2.8654 138.3715

VAL_145@O SER_146@HG SER_146@OG 1 0.0010 2.8662 152.9747

SER_132@OG PHE_182@H PHE_182@N 1 0.0010 2.8664 141.1438

ASP_348@OD2 ARG_305@HH21 ARG_305@NH2 1 0.0010 2.8680 143.7471

SER_148@O LYS_149@HZ3 LYS_149@NZ 1 0.0010 2.8683 150.9948

ARG_400@O ARG_400@HE ARG_400@NE 1 0.0010 2.8684 166.8968

ARG_437@O GLN_397@HE22 GLN_397@NE2 1 0.0010 2.8686 151.9562

ALA_377@O GLN_375@HE21 GLN_375@NE2 1 0.0010 2.8719 155.6834

GLN_1@NE2 THR_103@HG1 THR_103@OG1 1 0.0010 2.8723 149.7956

THR_50@O ALA_52@H ALA_52@N 1 0.0010 2.8735 135.2715

SER_289@OG THR_299@HG1 THR_299@OG1 1 0.0010 2.8741 136.0355

SER_392@OG GLN_334@HE22 GLN_334@NE2 1 0.0010 2.8744 138.7687

SER_72@OG LYS_67@HZ1 LYS_67@NZ 1 0.0010 2.8745 141.2360

ASP_162@OD2 LYS_169@HZ2 LYS_169@NZ 1 0.0010 2.8755 150.6644

THR_50@OG1 SER_51@H SER_51@N 1 0.0010 2.8761 136.7408

LEU_6@O THR_108@HG1 THR_108@OG1 1 0.0010 2.8770 149.1022

ASP_380@OD2 ASN_379@HD21 ASN_379@ND2 1 0.0010 2.8779 169.2441

GLU_360@OE2 GLN_406@HE22 GLN_406@NE2 1 0.0010 2.8781 145.6530

GLU_87@OE1 GLN_233@HE21 GLN_233@NE2 1 0.0010 2.8786 172.0297

GLN_142@O ASN_144@HD21 ASN_144@ND2 1 0.0010 2.8801 143.8942

GLN_44@OE1 ASN_60@HD21 ASN_60@ND2 1 0.0010 2.8826 162.6775

ASP_222@OD1 HIE_225@HE2 HIE_225@NE2 1 0.0010 2.8828 153.4713

GLY_61@O LYS_57@HZ1 LYS_57@NZ 1 0.0010 2.8843 137.8612

PRO_399@O HIE_402@HE2 HIE_402@NE2 1 0.0010 2.8845 135.3720

ASP_311@OD1 ARG_210@HH11 ARG_210@NH1 1 0.0010 2.8884 144.1857

ILE_155@O GLN_147@HE22 GLN_147@NE2 1 0.0010 2.8892 152.1686

LEU_272@O SER_271@HG SER_271@OG 1 0.0010 2.8898 146.7963

ASN_40@O GLN_41@HE22 GLN_41@NE2 1 0.0010 2.8901 171.8532

GLU_265@OE2 ARG_269@HH22 ARG_269@NH2 1 0.0010 2.8901 144.1862

SER_290@OG ASN_99@HD22 ASN_99@ND2 1 0.0010 2.8903 144.5272

LYS_149@O LYS_149@HZ2 LYS_149@NZ 1 0.0010 2.8906 148.0997

HIE_81@ND1 LYS_77@HZ2 LYS_77@NZ 1 0.0010 2.8913 144.9891

ASP_130@O LYS_179@H LYS_179@N 1 0.0010 2.8924 148.9475

SER_191@O ASN_144@HD21 ASN_144@ND2 1 0.0010 2.8927 137.0264

ARG_437@O ARG_437@HH11 ARG_437@NH1 1 0.0010 2.8929 145.8630

SER_276@OG GLN_281@HE22 GLN_281@NE2 1 0.0010 2.8934 145.4383

ALA_85@O ARG_109@HE ARG_109@NE 1 0.0010 2.8940 144.9294

GLU_327@OE2 TRP_396@HE1 TRP_396@NE1 1 0.0010 2.8962 148.6533

ASN_144@OD1 LYS_158@HZ2 LYS_158@NZ 1 0.0010 2.8978 172.4340

ASP_84@OD1 ASN_60@HD22 ASN_60@ND2 1 0.0010 2.8979 141.9632

HIE_332@ND1 ASN_118@HD21 ASN_118@ND2 1 0.0010 2.8993 142.2522

TYR_243@OH LYS_253@HZ3 LYS_253@NZ 1 0.0010 2.9000 141.6206

GLU_195@OE1 PHE_198@H PHE_198@N 1 0.0010 2.9008 161.7084

GLU_329@OE1 ARG_390@HH21 ARG_390@NH2 1 0.0010 2.9029 163.6623

GLU_374@OE1 LYS_131@HZ3 LYS_131@NZ 1 0.0010 2.9033 149.3991

VAL_220@O THR_201@HG1 THR_201@OG1 1 0.0010 2.9058 153.4396

LEU_6@O ARG_21@HH11 ARG_21@NH1 1 0.0010 2.9058 163.1570

PHE_182@O GLN_125@HE22 GLN_125@NE2 1 0.0010 2.9059 137.4769

GLU_195@O ASN_190@HD21 ASN_190@ND2 1 0.0010 2.9066 142.9961

GLY_212@O LYS_214@H LYS_214@N 1 0.0010 2.9067 159.9021

GLN_233@O GLN_233@HE22 GLN_233@NE2 1 0.0010 2.9068 137.3019

LYS_149@O LYS_149@HZ3 LYS_149@NZ 1 0.0010 2.9071 168.1289

GLN_281@OE1 ASN_280@HD22 ASN_280@ND2 1 0.0010 2.9081 151.2486

ALA_52@O THR_54@H THR_54@N 1 0.0010 2.9092 144.8027

ASN_178@O LYS_131@HZ3 LYS_131@NZ 1 0.0010 2.9095 157.4484

TRP_355@NE1 GLN_406@HE22 GLN_406@NE2 1 0.0010 2.9106 176.1900

SER_283@O ARG_305@HE ARG_305@NE 1 0.0010 2.9124 137.1524

SER_278@OG GLN_281@HE22 GLN_281@NE2 1 0.0010 2.9128 138.0119

GLN_397@O GLN_397@HE22 GLN_397@NE2 1 0.0010 2.9131 135.8540

ASN_144@O SER_146@H SER_146@N 1 0.0010 2.9136 147.1116

SER_151@OG ASP_152@H ASP_152@N 1 0.0010 2.9137 135.8488

TYR_154@OH ASN_178@HD22 ASN_178@ND2 1 0.0010 2.9143 143.9804

HIE_81@NE2 SER_79@HG SER_79@OG 1 0.0010 2.9145 145.9336

GLU_360@OE1 GLN_406@HE22 GLN_406@NE2 1 0.0010 2.9150 157.1469

ASP_348@O ARG_305@HH12 ARG_305@NH1 1 0.0010 2.9157 136.5237

GLN_5@O THR_4@HG1 THR_4@OG1 1 0.0010 2.9176 156.1879

TYR_124@OH HIE_332@HE2 HIE_332@NE2 1 0.0010 2.9178 137.8537

ASN_401@OD1 HIE_402@H HIE_402@N 1 0.0010 2.9184 139.2347

PRO_30@O LEU_32@H LEU_32@N 1 0.0010 2.9191 151.4657

GLU_14@OE2 HIE_81@HE2 HIE_81@NE2 1 0.0010 2.9197 162.2541

SER_261@OG LYS_251@HZ3 LYS_251@NZ 1 0.0010 2.9228 139.9160

THR_143@OG1 LYS_158@HZ2 LYS_158@NZ 1 0.0010 2.9234 138.0940

HIE_81@ND1 LYS_77@HZ1 LYS_77@NZ 1 0.0010 2.9249 152.1989

GLN_408@OE1 PHE_409@H PHE_409@N 1 0.0010 2.9262 147.7621

SER_72@OG LYS_67@HZ2 LYS_67@NZ 1 0.0010 2.9269 143.9177

GLN_125@OE1 VAL_133@H VAL_133@N 1 0.0010 2.9273 163.5283

SER_148@O SER_148@HG SER_148@OG 1 0.0010 2.9274 139.2317

ASP_130@O LYS_131@HZ1 LYS_131@NZ 1 0.0010 2.9277 165.7750

THR_156@O SER_146@H SER_146@N 1 0.0010 2.9293 139.2141

GLY_301@O GLY_42@H GLY_42@N 1 0.0010 2.9302 155.1806

GLU_87@OE2 GLN_233@HE21 GLN_233@NE2 1 0.0010 2.9313 159.0464

LEU_161@O MET_163@H MET_163@N 1 0.0010 2.9333 140.4798

ASN_40@ND2 ARG_205@HH22 ARG_205@NH2 1 0.0010 2.9337 149.2321

SER_141@OG GLN_142@H GLN_142@N 1 0.0010 2.9342 141.6803

SER_95@OG GLU_100@H GLU_100@N 1 0.0010 2.9346 156.9404

GLN_41@O GLN_44@HE22 GLN_44@NE2 1 0.0010 2.9353 156.2594

ASN_415@O GLU_417@H GLU_417@N 1 0.0010 2.9363 138.3165

GLU_94@OE1 GLN_1@H2 GLN_1@N 1 0.0010 2.9372 138.1607

ASN_99@ND2 LEU_294@H LEU_294@N 1 0.0010 2.9384 158.4888

SER_381@OG GLU_374@H GLU_374@N 1 0.0010 2.9392 140.7903

ASP_167@O LYS_169@HZ3 LYS_169@NZ 1 0.0010 2.9398 145.8736

GLN_125@NE2 CYX_184@H CYX_184@N 1 0.0010 2.9400 137.6026

LYS_67@O SER_72@HG SER_72@OG 1 0.0010 2.9403 135.5486

ARG_205@NH1 GLY_303@H GLY_303@N 1 0.0010 2.9419 135.0925

ASN_40@OD1 ARG_205@HH11 ARG_205@NH1 1 0.0010 2.9421 163.3182

ARG_437@NH1 ALA_438@H ALA_438@N 1 0.0010 2.9424 154.4298

GLU_324@OE2 ARG_437@HH21 ARG_437@NH2 1 0.0010 2.9432 147.5672

LYS_131@O VAL_133@H VAL_133@N 1 0.0010 2.9435 137.3938

SER_191@O ILE_193@H ILE_193@N 1 0.0010 2.9443 140.3372

THR_106@O GLY_238@H GLY_238@N 1 0.0010 2.9448 137.7191

SER_8@O ARG_109@HE ARG_109@NE 1 0.0010 2.9460 146.1614

GLU_213@OE2 LYS_209@HZ3 LYS_209@NZ 1 0.0010 2.9461 169.0375

ASN_118@OD1 ASN_118@H ASN_118@N 1 0.0010 2.9477 140.2860

SER_271@N TRP_230@HE1 TRP_230@NE1 1 0.0010 2.9488 172.1918

SER_172@O ARG_388@HH22 ARG_388@NH2 1 0.0010 2.9490 173.3023

PHE_198@O ASN_190@HD22 ASN_190@ND2 1 0.0010 2.9492 156.6956

TYR_296@OH HIE_225@HE2 HIE_225@NE2 1 0.0010 2.9512 163.3057

GLY_303@O GLN_202@HE21 GLN_202@NE2 1 0.0010 2.9518 135.1720

ASP_162@OD2 ARG_164@H ARG_164@N 1 0.0010 2.9521 152.2832

SER_363@O ARG_164@H ARG_164@N 1 0.0010 2.9534 152.9430

GLN_420@NE2 ASP_421@H ASP_421@N 1 0.0010 2.9538 139.4367

CYX_184@O ASN_186@H ASN_186@N 1 0.0010 2.9548 145.5766

TYR_49@OH ALA_53@H ALA_53@N 1 0.0010 2.9557 148.8875

SER_276@OG LYS_214@HZ2 LYS_214@NZ 1 0.0010 2.9562 169.5610

GLU_329@OE1 SER_326@H SER_326@N 1 0.0010 2.9563 165.5013

SER_263@OG LYS_249@HZ2 LYS_249@NZ 1 0.0010 2.9568 160.9657

SER_172@OG ARG_388@HH12 ARG_388@NH1 1 0.0010 2.9578 146.5456

ARG_400@NH1 ASN_398@HD21 ASN_398@ND2 1 0.0010 2.9580 163.7803

SER_72@OG ASN_23@HD21 ASN_23@ND2 1 0.0010 2.9600 144.2831

VAL_28@O GLN_1@HE21 GLN_1@NE2 1 0.0010 2.9618 136.3202

GLN_420@NE2 ARG_422@HH21 ARG_422@NH2 1 0.0010 2.9623 162.9089

GLU_87@OE1 THR_108@H THR_108@N 1 0.0010 2.9628 135.5291

ASN_280@OD1 GLN_281@HE22 GLN_281@NE2 1 0.0010 2.9638 156.5917

HIE_402@ND1 TRP_435@HE1 TRP_435@NE1 1 0.0010 2.9647 160.4793

ARG_240@NH2 ASP_234@H ASP_234@N 1 0.0010 2.9657 142.4713

GLN_5@NE2 THR_108@HG1 THR_108@OG1 1 0.0010 2.9662 143.7970

GLN_397@NE2 ARG_437@HH12 ARG_437@NH1 1 0.0010 2.9664 147.4713

ASP_152@O LYS_179@HZ1 LYS_179@NZ 1 0.0010 2.9665 143.5875

ILE_330@O HIE_332@H HIE_332@N 1 0.0010 2.9667 142.8807

GLN_44@NE2 GLN_41@HE22 GLN_41@NE2 1 0.0010 2.9671 153.2262

ASP_157@OD1 GLN_147@HE22 GLN_147@NE2 1 0.0010 2.9682 139.8669

VAL_28@O TYR_24@HH TYR_24@OH 1 0.0010 2.9685 156.5665

GLN_428@NE2 ILE_429@H ILE_429@N 1 0.0010 2.9692 141.7327

PHE_66@O LYS_67@HZ2 LYS_67@NZ 1 0.0010 2.9719 149.9070

PHE_97@O LYS_48@HZ1 LYS_48@NZ 1 0.0010 2.9723 157.4324

THR_106@OG1 GLN_5@H GLN_5@N 1 0.0010 2.9724 136.0960

SER_2@O SER_26@H SER_26@N 1 0.0010 2.9734 137.7756

ARG_205@NH1 TYR_206@HH TYR_206@OH 1 0.0010 2.9735 151.7940

PRO_194@O ASN_190@HD21 ASN_190@ND2 1 0.0010 2.9751 138.8394

LEU_6@O ARG_21@HE ARG_21@NE 1 0.0010 2.9752 135.8325

GLN_147@O SER_146@HG SER_146@OG 1 0.0010 2.9753 158.3042

GLN_41@OE1 GLN_44@HE21 GLN_44@NE2 1 0.0010 2.9756 141.2136

ASN_60@OD1 LYS_77@HZ2 LYS_77@NZ 1 0.0010 2.9766 138.6424

LYS_149@O LYS_149@HZ1 LYS_149@NZ 1 0.0010 2.9771 146.2686

ASN_186@ND2 ALA_183@H ALA_183@N 1 0.0010 2.9774 162.7534

TYR_206@OH ARG_205@HE ARG_205@NE 1 0.0010 2.9803 165.5798

GLU_65@O LYS_67@H LYS_67@N 1 0.0010 2.9804 137.3561

GLU_327@OE1 GLN_397@HE21 GLN_397@NE2 1 0.0010 2.9804 146.4220

ARG_400@NH2 ASN_398@HD21 ASN_398@ND2 1 0.0010 2.9806 168.9646

GLU_374@OE2 LYS_131@HZ2 LYS_131@NZ 1 0.0010 2.9808 150.5380

GLN_125@NE2 SER_132@HG SER_132@OG 1 0.0010 2.9808 161.5754

TYR_383@OH LYS_373@HZ1 LYS_373@NZ 1 0.0010 2.9811 152.5547

GLN_281@NE2 SER_276@HG SER_276@OG 1 0.0010 2.9837 139.0683

ALA_377@O GLN_375@HE22 GLN_375@NE2 1 0.0010 2.9855 153.0334

SER_381@O SER_381@HG SER_381@OG 1 0.0010 2.9857 135.6008

HIE_74@NE2 ARG_21@HH11 ARG_21@NH1 1 0.0010 2.9860 144.5749

ASN_144@O ASN_144@HD22 ASN_144@ND2 1 0.0010 2.9863 150.5901

SER_276@OG LYS_214@HZ1 LYS_214@NZ 1 0.0010 2.9865 138.4762

HIE_225@N GLN_221@HE21 GLN_221@NE2 1 0.0010 2.9866 147.3937

SER_180@OG LYS_131@H LYS_131@N 1 0.0010 2.9871 145.8870

ASN_178@O LYS_131@HZ2 LYS_131@NZ 1 0.0010 2.9872 153.7133

GLU_195@O THR_197@H THR_197@N 1 0.0010 2.9883 137.1685

GLU_329@O LYS_335@H LYS_335@N 1 0.0010 2.9889 165.1998

ASN_379@OD1 LYS_314@HZ1 LYS_314@NZ 1 0.0010 2.9892 146.5490

SER_177@OG SER_132@H SER_132@N 1 0.0010 2.9895 147.0834

GLU_87@OE1 GLN_233@HE22 GLN_233@NE2 1 0.0010 2.9897 145.7744

ASP_157@N GLN_147@HE21 GLN_147@NE2 1 0.0010 2.9899 156.0575

SER_413@OG LYS_209@HZ2 LYS_209@NZ 1 0.0010 2.9908 163.8879

ASN_227@ND2 ASN_227@H ASN_227@N 1 0.0010 2.9925 147.2583

ALA_64@O PHE_66@H PHE_66@N 1 0.0010 2.9939 135.5622

ASP_196@OD1 ASN_190@HD21 ASN_190@ND2 1 0.0010 2.9940 167.8482

GLN_221@NE2 HIE_225@H HIE_225@N 1 0.0010 2.9941 147.5627

PHE_409@O GLN_408@HE22 GLN_408@NE2 1 0.0010 2.9941 156.4879

SER_263@OG GLU_265@H GLU_265@N 1 0.0010 2.9942 139.0537

THR_111@O THR_111@HG1 THR_111@OG1 1 0.0010 2.9956 136.0021

THR_307@OG1 ARG_305@HH12 ARG_305@NH1 1 0.0010 2.9971 136.8319

SER_25@O GLN_1@HE21 GLN_1@NE2 1 0.0010 2.9973 141.7014

GLN_142@NE2 GLU_195@H GLU_195@N 1 0.0010 2.9973 140.2077

ILE_116@O ASN_118@H ASN_118@N 1 0.0010 2.9983 137.3204

THR_71@OG1 ASN_23@HD21 ASN_23@ND2 1 0.0010 2.9985 159.0303

ASN_186@O ASN_186@HD22 ASN_186@ND2 1 0.0010 2.9991 140.3744

GLU_265@OE2 LYS_266@HZ3 LYS_266@NZ 1 0.0010 2.9996 144.2222
