# Supplementary material for: Computational discovery of binding mode of anti-TRBC1 antibody and predicted key amino acids of TRBC1
Source: Sci Rep. 2022 Feb 2;12:1760. doi: 10.1038/s41598-022-05742-6 (PMC8810837; doi:10.1038/s41598-022-05742-6)
Supplement: Supplementary file 2 — Supplementary Information 2. [file 41598_2022_5742_MOESM2_ESM.docx]

**TRBC2-H-bond**

#Acceptor DonorH Donor Frames Frac AvgDist AvgAng

TYR_300@O TYR_99@HH TYR_99@OH 978 0.9780 2.7005 164.3622

PRO_209@O THR_308@HG1 THR_308@OG1 975 0.9750 2.6853 163.6112

ASP_81@O TYR_85@HH TYR_85@OH 964 0.9640 2.6998 165.0582

ASP_90@O THR_93@HG1 THR_93@OG1 954 0.9540 2.7327 163.0380

PRO_8@O THR_105@HG1 THR_105@OG1 948 0.9480 2.7258 164.7497

PRO_194@O THR_197@HG1 THR_197@OG1 939 0.9390 2.7384 162.5446

ASN_31@OD1 TYR_48@HH TYR_48@OH 922 0.9220 2.7144 163.9338

GLU_333@OE2 THR_341@HG1 THR_341@OG1 920 0.9200 2.7252 154.8521

GLN_302@OE1 TYR_236@HH TYR_236@OH 912 0.9120 2.7347 159.8869

ASP_287@O TYR_291@HH TYR_291@OH 906 0.9060 2.7361 164.9481

ALA_294@O TYR_234@H TYR_234@N 905 0.9050 2.8392 158.7973

THR_96@O TYR_234@HH TYR_234@OH 903 0.9030 2.7459 165.6857

VAL_343@O PHE_327@H PHE_327@N 900 0.9000 2.8491 163.0412

PHE_349@O TYR_387@H TYR_387@N 899 0.8990 2.8519 162.5990

GLU_301@OE1 SER_296@HG SER_296@OG 898 0.8980 2.6496 162.2330

ASP_372@OD2 SER_390@H SER_390@N 896 0.8960 2.8280 157.8724

ASP_287@OD2 ARG_265@HH12 ARG_265@NH1 895 0.8950 2.8505 155.0491

GLU_269@O SER_276@HG SER_276@OG 892 0.8920 2.7508 160.4929

ARG_237@O GLU_245@H GLU_245@N 889 0.8890 2.8460 159.4833

LEU_233@O PHE_250@H PHE_250@N 882 0.8820 2.8510 160.4271

VAL_84@O GLN_37@H GLN_37@N 876 0.8760 2.8330 159.5196

ASP_287@OD2 GLU_284@H GLU_284@N 870 0.8700 2.8506 164.4365

GLU_333@O THR_337@HG1 THR_337@OG1 866 0.8660 2.7425 160.6520

GLU_333@OE1 THR_341@H THR_341@N 861 0.8610 2.8359 158.3867

LEU_393@O LEU_342@H LEU_342@N 856 0.8560 2.8554 159.8377

THR_313@O ARG_216@H ARG_216@N 847 0.8470 2.8533 161.3107

ASP_152@O SER_177@HG SER_177@OG 844 0.8440 2.7229 161.4021

ARG_408@O TRP_359@H TRP_359@N 833 0.8330 2.8552 155.4001

PHE_86@O PHE_35@H PHE_35@N 829 0.8290 2.8650 159.0016

ALA_17@O SER_77@HG SER_77@OG 827 0.8270 2.7594 160.5372

GLU_245@O ARG_237@H ARG_237@N 824 0.8240 2.8694 157.1552

THR_308@O TYR_291@H TYR_291@N 821 0.8210 2.8629 161.7233

SER_77@O GLY_16@H GLY_16@N 819 0.8190 2.8536 159.0747

SER_19@O ILE_74@H ILE_74@N 817 0.8170 2.8643 159.3552

ARG_426@O TRP_422@HE1 TRP_422@NE1 814 0.8140 2.8392 158.1843

LEU_72@O THR_20@HG1 THR_20@OG1 806 0.8060 2.7619 159.3964

SER_70@O CYX_23@H CYX_23@N 805 0.8050 2.8596 158.5112

GLU_333@OE2 ARG_394@HH21 ARG_394@NH2 800 0.8000 2.8568 147.9005

HIE_230@ND1 SER_295@HG SER_295@OG 799 0.7990 2.8063 163.1627

SER_60@O TYR_73@H TYR_73@N 799 0.7990 2.8597 158.8389

TYR_121@O LEU_135@H LEU_135@N 798 0.7980 2.8679 161.3116

ASP_157@O THR_156@HG1 THR_156@OG1 795 0.7950 2.7762 162.1606

GLU_333@O THR_337@H THR_337@N 791 0.7910 2.8720 156.2957

MET_290@O GLN_238@H GLN_238@N 790 0.7900 2.8515 155.7804

GLN_285@O SER_288@HG SER_288@OG 789 0.7890 2.7896 161.7738

SER_391@O CYX_344@H CYX_344@N 789 0.7890 2.8684 162.4709

TRP_34@O LEU_46@H LEU_46@N 785 0.7850 2.8671 157.2042

GLU_331@O SER_335@HG SER_335@OG 783 0.7830 2.7523 162.4187

VAL_133@O LEU_123@H LEU_123@N 774 0.7740 2.8851 162.2949

THR_79@O SER_82@HG SER_82@OG 773 0.7730 2.7792 161.6587

ASP_90@OD1 ASN_31@HD22 ASN_31@ND2 772 0.7720 2.8614 164.7042

GLN_218@O THR_283@HG1 THR_283@OG1 771 0.7710 2.7903 163.4033

VAL_434@O VAL_411@H VAL_411@N 771 0.7710 2.8765 161.9762

ILE_49@O THR_63@HG1 THR_63@OG1 765 0.7650 2.7988 162.2028

LEU_13@O LEU_110@H LEU_110@N 762 0.7620 2.8662 162.9277

CYX_87@O GLY_102@H GLY_102@N 761 0.7610 2.8601 162.5657

GLN_207@O ARG_210@HH12 ARG_210@NH1 761 0.7610 2.8722 156.1396

GLU_301@OE1 GLU_301@H GLU_301@N 757 0.7570 2.7612 144.7436

GLY_368@O ARG_394@H ARG_394@N 757 0.7570 2.8723 162.2956

THR_105@O TYR_85@H TYR_85@N 757 0.7570 2.8793 161.8970

PHE_407@O ALA_438@H ALA_438@N 753 0.7530 2.8778 153.9537

TYR_85@O GLN_6@HE21 GLN_6@NE2 751 0.7510 2.8571 155.2710

ALA_438@O PHE_407@H PHE_407@N 745 0.7450 2.8714 158.3100

ASP_372@OD1 ARG_392@HH12 ARG_392@NH1 743 0.7430 2.8629 155.7220

LEU_107@O GLY_83@H GLY_83@N 742 0.7420 2.8763 160.0105

GLY_299@O GLN_302@HE22 GLN_302@NE2 734 0.7340 2.8747 164.0621

ARG_386@O GLU_378@H GLU_378@N 733 0.7330 2.8692 163.3999

GLN_432@O PHE_413@H PHE_413@N 732 0.7320 2.8768 160.8034

ILE_44@O HIE_36@H HIE_36@N 732 0.7320 2.8826 160.9190

GLU_333@OE2 ARG_394@HE ARG_394@NE 730 0.7300 2.8746 150.7533

LEU_110@O GLU_15@H GLU_15@N 729 0.7290 2.8686 159.0760

LEU_278@O PHE_222@H PHE_222@N 726 0.7260 2.8782 161.2198

LEU_292@O TYR_236@H TYR_236@N 724 0.7240 2.8701 155.1598

CYX_370@O ARG_392@H ARG_392@N 722 0.7220 2.8788 157.5883

GLN_302@O GLN_33@HE21 GLN_33@NE2 721 0.7210 2.8591 157.0896

HIE_36@O ILE_44@H ILE_44@N 719 0.7190 2.8629 157.9111

LEU_389@O ALA_346@H ALA_346@N 718 0.7180 2.8825 162.1097

GLU_355@O GLN_412@H GLN_412@N 717 0.7170 2.8801 161.9945

ASN_405@O GLY_440@H GLY_440@N 716 0.7160 2.8734 159.1040

ALA_175@O CYX_134@H CYX_134@N 716 0.7160 2.8834 162.6639

GLU_257@O THR_248@HG1 THR_248@OG1 715 0.7150 2.7488 158.4817

THR_248@O TRP_235@H TRP_235@N 713 0.7130 2.8785 159.2146

GLY_307@O ARG_210@HH11 ARG_210@NH1 712 0.7120 2.8647 152.5550

ALA_173@O PHE_136@H PHE_136@N 712 0.7120 2.8908 161.6340

LEU_59@O ASN_56@H ASN_56@N 705 0.7050 2.8688 160.4243

GLN_242@O GLN_238@HE22 GLN_238@NE2 701 0.7010 2.8665 160.1343

ALA_346@O LEU_389@H LEU_389@N 701 0.7010 2.8863 160.6087

THR_62@O LEU_71@H LEU_71@N 699 0.6990 2.8880 157.5174

ASP_90@OD2 ASN_30@H ASN_30@N 698 0.6980 2.8761 160.5281

GLN_78@O ASP_81@H ASP_81@N 695 0.6950 2.8856 159.3743

TRP_359@O ARG_408@H ARG_408@N 695 0.6950 2.8877 160.0950

SER_274@O ARG_270@HE ARG_270@NE 695 0.6950 2.8927 160.3411

VAL_89@O ILE_100@H ILE_100@N 694 0.6940 2.8893 163.6203

PHE_399@O ASN_405@HD22 ASN_405@ND2 690 0.6900 2.8691 156.3499

GLY_83@O LEU_107@H LEU_107@N 686 0.6860 2.8815 158.2217

PHE_168@O MET_163@H MET_163@N 685 0.6850 2.8791 160.7181

ALA_88@O GLN_33@H GLN_33@N 683 0.6830 2.8983 161.0373

ASN_219@OD1 ARG_282@H ARG_282@N 682 0.6820 2.8723 162.0931

GLN_37@OE1 GLN_238@HE21 GLN_238@NE2 680 0.6800 2.8565 157.5366

LYS_363@O VAL_360@H VAL_360@N 677 0.6770 2.8851 161.0184

ALA_17@O SER_77@H SER_77@N 675 0.6750 2.8726 157.6329

TYR_236@O LEU_292@H LEU_292@N 675 0.6750 2.8838 163.4632

ASP_90@OD2 ALA_92@H ALA_92@N 674 0.6740 2.8764 156.4505

LEU_135@O TYR_121@H TYR_121@N 667 0.6670 2.8952 161.0821

TRP_176@O TYR_154@H TYR_154@N 667 0.6670 2.8953 161.7554

ASN_18@OD1 SER_76@H SER_76@N 665 0.6650 2.8704 162.4278

LYS_54@O ALA_61@H ALA_61@N 658 0.6580 2.8893 157.4174

LEU_376@O ALA_388@H ALA_388@N 653 0.6530 2.8676 151.1943

ARG_265@O GLN_281@H GLN_281@N 650 0.6500 2.8811 160.9474

SER_391@OG TRP_358@HE1 TRP_358@NE1 647 0.6470 2.8874 157.0580

SER_276@O CYX_224@H CYX_224@N 646 0.6460 2.8872 155.5357

TRP_235@O LEU_247@H LEU_247@N 645 0.6450 2.8819 151.3020

SER_267@O GLU_279@H GLU_279@N 641 0.6410 2.8747 161.6923

LEU_21@O LEU_72@H LEU_72@N 641 0.6410 2.8970 162.8729

TYR_234@O ALA_294@H ALA_294@N 640 0.6400 2.8932 157.6742

GLN_33@O ALA_88@H ALA_88@N 637 0.6370 2.8831 153.6074

ALA_397@O GLN_401@H GLN_401@N 634 0.6340 2.8796 157.1821

LEU_247@O SER_259@H SER_259@N 634 0.6340 2.8875 159.6794

GLY_52@O THR_63@H THR_63@N 633 0.6330 2.8849 161.9647

GLU_5@O ASN_24@H ASN_24@N 628 0.6280 2.8868 159.8125

CYX_134@O ALA_175@H ALA_175@N 627 0.6270 2.8935 156.7423

GLN_238@OE1 GLN_37@HE21 GLN_37@NE2 622 0.6220 2.8695 163.7058

VAL_319@O ARG_426@HH12 ARG_426@NH1 621 0.6210 2.8792 154.2225

ASN_190@OD1 SER_191@HG SER_191@OG 618 0.6180 2.8075 160.6989

VAL_395@O ALA_340@H ALA_340@N 618 0.6180 2.8689 151.9025

TYR_69@O VAL_64@H VAL_64@N 617 0.6170 2.8877 160.3058

GLU_323@O THR_347@H THR_347@N 616 0.6160 2.8935 159.7441

PHE_136@O ALA_173@H ALA_173@N 614 0.6140 2.9040 155.4425

THR_347@O GLU_323@H GLU_323@N 613 0.6130 2.8908 164.2294

ASP_125@O ARG_124@HE ARG_124@NE 598 0.5980 2.8761 149.3315

ALA_289@O LEU_310@H LEU_310@N 597 0.5970 2.8968 161.3570

ARG_22@O SER_7@H SER_7@N 592 0.5920 2.8883 160.3335

GLU_301@OE2 GLY_298@H GLY_298@N 589 0.5890 2.8697 159.1447

TYR_291@O GLN_207@HE21 GLN_207@NE2 589 0.5890 2.8764 156.5460

VAL_220@O ILE_280@H ILE_280@N 588 0.5880 2.8962 161.5825

ASN_231@O ASN_252@H ASN_252@N 584 0.5840 2.8862 157.8158

ALA_436@O CYX_409@H CYX_409@N 579 0.5790 2.9007 164.5096

ASP_372@OD2 SER_390@HG SER_390@OG 578 0.5780 2.7935 163.9565

LYS_212@O THR_311@H THR_311@N 577 0.5770 2.8858 155.1543

ASN_361@OD1 HIE_406@H HIE_406@N 576 0.5760 2.8771 160.7637

VAL_430@O GLN_432@HE22 GLN_432@NE2 575 0.5750 2.8733 162.4483

PRO_429@O LEU_416@H LEU_416@N 574 0.5740 2.8976 162.9240

ASP_287@OD1 ARG_265@HH22 ARG_265@NH2 572 0.5720 2.8925 151.9885

GLN_6@OE1 CYX_87@H CYX_87@N 569 0.5690 2.8817 157.1718

SER_357@O GLN_410@H GLN_410@N 567 0.5670 2.9015 157.7113

PHE_35@O PHE_86@H PHE_86@N 565 0.5650 2.9041 164.2247

ASP_125@O ARG_124@HH21 ARG_124@NH2 561 0.5610 2.8774 147.1830

CYX_344@O SER_391@H SER_391@N 561 0.5610 2.8946 155.2908

ASP_162@O GLY_368@H GLY_368@N 560 0.5600 2.8353 148.1997

SER_206@O ASP_225@H ASP_225@N 559 0.5590 2.8834 156.3615

ALA_254@O GLN_251@H GLN_251@N 558 0.5580 2.8932 159.1681

ALA_183@O ASN_186@H ASN_186@N 557 0.5570 2.8966 155.4497

HIE_353@O TYR_414@H TYR_414@N 556 0.5560 2.9009 162.3189

ARG_124@O VAL_328@H VAL_328@N 555 0.5550 2.9043 158.2862

VAL_326@O SER_126@H SER_126@N 553 0.5530 2.8845 155.2137

ARG_210@O ARG_309@H ARG_309@N 551 0.5510 2.8922 163.6145

ARG_223@O ASN_208@H ASN_208@N 551 0.5510 2.8927 161.7875

ASN_231@O ARG_270@HH12 ARG_270@NH1 550 0.5500 2.8928 153.8265

GLN_238@O MET_290@H MET_290@N 545 0.5450 2.8867 163.4837

LEU_11@O LYS_108@H LYS_108@N 545 0.5450 2.8940 158.7447

GLU_257@O TYR_249@H TYR_249@N 542 0.5420 2.8921 155.5898

TYR_350@O PHE_320@H PHE_320@N 538 0.5380 2.9057 162.0330

ASP_181@O ASN_186@HD21 ASN_186@ND2 535 0.5350 2.8696 157.9218

SER_82@O HIE_36@HE2 HIE_36@NE2 535 0.5350 2.8729 155.4313

ILE_100@O VAL_89@H VAL_89@N 535 0.5350 2.9041 160.8265

GLN_410@O SER_357@H SER_357@N 535 0.5350 2.9077 160.0620

LEU_123@O VAL_133@H VAL_133@N 533 0.5330 2.9052 157.6285

SER_7@O ARG_22@H ARG_22@N 532 0.5320 2.9017 162.2254

ALA_340@O VAL_395@H VAL_395@N 531 0.5310 2.9086 164.9131

THR_63@OG1 SER_70@HG SER_70@OG 530 0.5300 2.8339 156.8169

PRO_118@O PHE_198@H PHE_198@N 529 0.5290 2.8993 164.0353

LYS_215@O GLN_218@H GLN_218@N 529 0.5290 2.9003 158.1161

GLU_331@O SER_335@H SER_335@N 527 0.5270 2.8894 157.9969

LEU_342@O LEU_393@H LEU_393@N 525 0.5250 2.9097 163.4451

ARG_58@O SER_75@H SER_75@N 524 0.5240 2.8949 161.2725

ASP_384@OD1 THR_313@HG1 THR_313@OG1 520 0.5200 2.7144 164.9317

THR_93@O TYR_97@H TYR_97@N 519 0.5190 2.8845 161.8652

ILE_74@O SER_19@H SER_19@N 518 0.5180 2.9024 157.1878

GLN_338@O ALA_397@H ALA_397@N 513 0.5130 2.8937 159.7479

HIE_406@O ASN_361@H ASN_361@N 510 0.5100 2.8968 162.2793

PHE_320@O TYR_350@H TYR_350@N 509 0.5090 2.8961 159.7346

HIE_230@O ARG_270@HH22 ARG_270@NH2 508 0.5080 2.8947 153.7317

ASP_138@OD2 THR_137@HG1 THR_137@OG1 507 0.5070 2.7102 165.6912

ARG_106@O LEU_11@H LEU_11@N 500 0.5000 2.8770 151.9472

ARG_309@O LYS_212@H LYS_212@N 496 0.4960 2.8811 150.4775

LEU_32@O ILE_49@H ILE_49@N 496 0.4960 2.9089 162.3668

CYX_293@O GLY_305@H GLY_305@N 494 0.4940 2.8808 160.2492

PHE_47@O TRP_34@H TRP_34@N 494 0.4940 2.8995 157.9031

SER_170@O LEU_161@H LEU_161@N 494 0.4940 2.9142 162.5482

GLU_301@OE1 GLY_299@H GLY_299@N 491 0.4910 2.8277 147.7427

GLN_410@OE1 ARG_408@HH11 ARG_408@NH1 491 0.4910 2.8811 154.3210

GLU_378@O TYR_154@HH TYR_154@OH 490 0.4900 2.7367 162.9313

PHE_198@O VAL_120@H VAL_120@N 486 0.4860 2.9033 157.0449

ASP_420@OD1 SER_417@HG SER_417@OG 483 0.4830 2.6897 164.3561

LYS_131@O ASP_125@H ASP_125@N 482 0.4820 2.8866 156.4670

ASP_138@OD1 ARG_394@HH12 ARG_394@NH1 476 0.4760 2.8863 155.0530

ASP_81@OD1 ARG_58@HH12 ARG_58@NH1 475 0.4750 2.8581 155.3599

ASP_138@OD2 ARG_394@HH12 ARG_394@NH1 475 0.4750 2.8831 153.5328

ILE_280@O VAL_220@H VAL_220@N 475 0.4750 2.9056 158.7991

SER_172@OG ARG_392@HE ARG_392@NE 474 0.4740 2.9148 152.0464

TYR_291@O THR_308@H THR_308@N 473 0.4730 2.8942 150.3980

GLU_437@O SER_126@HG SER_126@OG 469 0.4690 2.7423 160.9504

GLU_364@OE2 GLU_364@H GLU_364@N 469 0.4690 2.8300 151.4491

ASP_81@OD1 GLN_78@H GLN_78@N 468 0.4680 2.8486 163.2840

LEU_71@O THR_62@H THR_62@N 466 0.4660 2.9048 158.9953

ASP_81@OD2 ARG_58@HH22 ARG_58@NH2 463 0.4630 2.8899 151.9416

SER_82@OG VAL_109@H VAL_109@N 463 0.4630 2.9090 158.2412

ASP_140@OD1 THR_143@HG1 THR_143@OG1 460 0.4600 2.7085 163.6735

THR_308@OG1 GLN_207@HE22 GLN_207@NE2 460 0.4600 2.8817 156.1364

ALA_119@O THR_137@H THR_137@N 460 0.4600 2.9131 159.2028

GLN_218@O THR_283@H THR_283@N 454 0.4540 2.9005 161.5653

PRO_351@O HIE_353@H HIE_353@N 449 0.4490 2.8805 144.6989

GLY_41@O GLN_37@HE22 GLN_37@NE2 449 0.4490 2.8868 160.0694

ASN_38@O GLY_41@H GLY_41@N 449 0.4490 2.8943 152.9157

SER_70@OG TRP_34@HE1 TRP_34@NE1 449 0.4490 2.9159 159.2875

ASN_252@O ARG_270@HH11 ARG_270@NH1 447 0.4470 2.8835 148.0404

SER_26@OG GLN_3@H GLN_3@N 443 0.4430 2.9131 159.2344

ASP_420@OD2 SER_417@HG SER_417@OG 442 0.4420 2.6859 163.8591

GLN_33@OE1 ASN_45@HD21 ASN_45@ND2 442 0.4420 2.8642 151.6638

CYX_23@O SER_70@H SER_70@N 442 0.4420 2.8977 155.0938

ASP_138@OD1 THR_137@HG1 THR_137@OG1 441 0.4410 2.7111 165.6984

ALA_325@O LEU_345@H LEU_345@N 440 0.4400 2.9071 161.0789

GLU_253@OE1 GLU_253@H GLU_253@N 432 0.4320 2.8192 150.0800

TYR_154@O TRP_176@H TRP_176@N 427 0.4270 2.9144 155.9781

LEU_161@O SER_170@H SER_170@N 426 0.4260 2.9102 159.0262

ASP_157@OD2 LYS_158@H LYS_158@N 425 0.4250 2.8132 145.1153

THR_283@O GLY_217@H GLY_217@N 423 0.4230 2.8550 148.9160

ASP_81@OD1 ARG_58@HH22 ARG_58@NH2 421 0.4210 2.8961 151.0817

ASN_24@O GLU_5@H GLU_5@N 419 0.4190 2.8898 153.9863

PHE_222@O LEU_278@H LEU_278@N 419 0.4190 2.9137 160.2764

ALA_61@O LYS_54@H LYS_54@N 418 0.4180 2.9067 158.2294

THR_156@OG1 VAL_174@H VAL_174@N 418 0.4180 2.9190 162.4936

TYR_85@O THR_105@H THR_105@N 413 0.4130 2.8993 152.6165

VAL_174@O THR_156@H THR_156@N 410 0.4100 2.9067 163.9701

GLU_284@O ASP_287@H ASP_287@N 410 0.4100 2.9083 157.8778

GLN_42@O ASN_38@H ASN_38@N 408 0.4080 2.9017 159.5905

CYX_224@O SER_276@H SER_276@N 408 0.4080 2.9081 159.9613

ALA_183@O ALA_187@H ALA_187@N 406 0.4060 2.8786 152.5664

ASP_140@OD2 THR_143@HG1 THR_143@OG1 404 0.4040 2.7283 162.5984

ASN_171@O ASP_138@H ASP_138@N 404 0.4040 2.9018 152.8953

LEU_416@O THR_431@H THR_431@N 403 0.4030 2.8614 151.9206

TYR_249@O LEU_256@H LEU_256@N 403 0.4030 2.8794 154.2301

ASP_81@OD2 ARG_58@HH12 ARG_58@NH1 402 0.4020 2.8587 154.2644

THR_93@OG1 LYS_98@H LYS_98@N 400 0.4000 2.9081 156.3897

ASP_420@OD1 SER_417@H SER_417@N 397 0.3970 2.8589 158.7481

THR_214@O THR_313@H THR_313@N 392 0.3920 2.9131 158.2935

GLU_269@O THR_277@H THR_277@N 392 0.3920 2.9171 157.5489

GLU_314@OE2 ARG_216@HE ARG_216@NE 390 0.3900 2.8802 157.0410

ASP_287@OD2 ARG_265@HH22 ARG_265@NH2 388 0.3880 2.9055 145.9779

GLU_253@OE2 GLU_253@H GLU_253@N 387 0.3870 2.8163 149.5906

GLN_37@O VAL_84@H VAL_84@N 386 0.3860 2.9035 157.9892

ASP_81@OD2 GLN_78@H GLN_78@N 383 0.3830 2.8503 163.0791

THR_137@O ALA_119@H ALA_119@N 383 0.3830 2.9172 159.8732

ARG_270@O GLY_273@H GLY_273@N 379 0.3790 2.9032 155.6418

SER_357@OG TRP_359@HE1 TRP_359@NE1 379 0.3790 2.9063 154.4934

GLU_314@OE1 ARG_216@HH21 ARG_216@NH2 378 0.3780 2.8752 154.9479

GLU_364@OE1 GLU_364@H GLU_364@N 377 0.3770 2.8354 152.1229

ASP_402@OD2 ARG_404@HE ARG_404@NE 376 0.3760 2.8755 155.1576

ASP_420@OD2 SER_417@H SER_417@N 374 0.3740 2.8652 158.1812

SER_191@OG SER_146@H SER_146@N 374 0.3740 2.9129 162.0558

TRP_400@O ARG_441@H ARG_441@N 369 0.3690 2.8923 153.0972

SER_75@O ARG_58@HH11 ARG_58@NH1 368 0.3680 2.9066 155.1661

GLN_207@OE1 CYX_293@H CYX_293@N 365 0.3650 2.8911 160.4133

ASP_90@OD2 ASN_31@H ASN_31@N 362 0.3620 2.9137 162.3141

PHE_188@O SER_191@H SER_191@N 359 0.3590 2.9007 155.6482

ASP_384@OD2 THR_313@HG1 THR_313@OG1 356 0.3560 2.7096 163.8974

GLU_195@OE2 GLU_195@H GLU_195@N 350 0.3500 2.8245 150.8148

ASN_405@OD1 ASN_361@HD21 ASN_361@ND2 349 0.3490 2.8845 161.3105

GLU_229@OE1 GLU_229@H GLU_229@N 348 0.3480 2.8325 155.2048

LYS_428@O VAL_430@H VAL_430@N 348 0.3480 2.8668 143.2250

ASN_38@OD1 GLN_42@H GLN_42@N 347 0.3470 2.9057 154.9870

THR_105@OG1 GLN_6@HE22 GLN_6@NE2 346 0.3460 2.9070 157.8508

GLN_379@O SER_385@HG SER_385@OG 345 0.3450 2.7610 154.6522

GLU_378@OE1 ARG_386@HE ARG_386@NE 345 0.3450 2.8877 156.2541

ASN_208@O ARG_223@H ARG_223@N 344 0.3440 2.9123 163.1030

VAL_411@O VAL_434@H VAL_434@N 344 0.3440 2.9194 161.5062

CYX_409@O ALA_436@H ALA_436@N 342 0.3420 2.9219 159.4584

ASN_31@O ASP_90@H ASP_90@N 341 0.3410 2.9183 161.4204

CYX_370@O SER_391@HG SER_391@OG 339 0.3390 2.7477 152.4806

GLU_195@OE1 GLU_195@H GLU_195@N 339 0.3390 2.8309 151.2306

GLU_418@OE2 GLU_418@H GLU_418@N 339 0.3390 2.8350 152.2858

GLU_418@OE1 GLU_418@H GLU_418@N 338 0.3380 2.8338 151.1739

GLU_331@OE1 GLU_331@H GLU_331@N 337 0.3370 2.8204 151.0834

LEU_297@O ARG_232@HH11 ARG_232@NH1 337 0.3370 2.8847 157.8400

SER_288@OG VAL_312@H VAL_312@N 337 0.3370 2.9096 152.3452

ASN_231@OD1 GLN_251@HE22 GLN_251@NE2 335 0.3350 2.8584 160.6792

PHE_250@O LEU_233@H LEU_233@N 335 0.3350 2.9204 162.9687

SER_172@O CYX_159@H CYX_159@N 332 0.3320 2.9139 156.4597

THR_371@OG1 SER_391@HG SER_391@OG 331 0.3310 2.8320 158.3334

LYS_108@O LEU_13@H LEU_13@N 330 0.3300 2.9011 158.4690

GLY_95@O TYR_249@HH TYR_249@OH 329 0.3290 2.7813 157.6096

ASP_157@OD1 LYS_158@H LYS_158@N 329 0.3290 2.8189 143.8858

GLU_437@OE1 HIE_406@HE2 HIE_406@NE2 327 0.3270 2.8402 161.2638

THR_311@O THR_214@H THR_214@N 326 0.3260 2.9004 154.2086

GLU_314@OE1 ARG_216@HE ARG_216@NE 325 0.3250 2.8855 156.8316

GLN_281@O ARG_265@HH11 ARG_265@NH1 325 0.3250 2.9227 158.6683

GLN_412@O GLU_355@H GLU_355@N 323 0.3230 2.9028 155.8135

PRO_9@O ARG_106@H ARG_106@N 321 0.3210 2.9072 161.4751

LEU_316@O VAL_319@H VAL_319@N 320 0.3200 2.9164 159.3213

GLU_229@OE2 GLU_229@H GLU_229@N 319 0.3190 2.8404 155.1683

SER_276@OG TRP_235@HE1 TRP_235@NE1 319 0.3190 2.9142 161.0590

ASP_138@OD2 ARG_394@HH22 ARG_394@NH2 318 0.3180 2.8875 152.6852

ASP_125@OD1 LYS_127@H LYS_127@N 317 0.3170 2.8857 163.2134

ALA_332@O HIE_336@H HIE_336@N 316 0.3160 2.8835 153.9504

TYR_73@O SER_60@H SER_60@N 316 0.3160 2.9147 157.1560

VAL_319@O ARG_426@HH22 ARG_426@NH2 311 0.3110 2.8946 148.1426

SER_263@O PHE_266@H PHE_266@N 302 0.3020 2.9030 156.3009

PHE_246@O SER_259@HG SER_259@OG 300 0.3000 2.7860 160.1590

ASP_27@OD2 SER_28@H SER_28@N 300 0.3000 2.8232 145.8353

ASP_225@O SER_206@H SER_206@N 297 0.2970 2.9130 162.9262

GLU_378@OE2 ARG_386@HE ARG_386@NE 296 0.2960 2.8906 156.4223

ARG_392@O CYX_370@H CYX_370@N 295 0.2950 2.9194 156.0419

THR_248@OG1 GLN_255@HE22 GLN_255@NE2 294 0.2940 2.9029 153.2065

ASP_384@OD2 TYR_350@HH TYR_350@OH 292 0.2920 2.7045 158.3610

THR_277@O GLU_269@H GLU_269@N 290 0.2900 2.9180 156.4626

TYR_97@O GLN_302@HE21 GLN_302@NE2 288 0.2880 2.8773 147.7294

ASP_140@OD2 THR_143@H THR_143@N 287 0.2870 2.8364 153.7821

SER_170@OG ARG_394@HH11 ARG_394@NH1 287 0.2870 2.9204 146.8062

ILE_227@O HIE_230@H HIE_230@N 286 0.2860 2.9127 159.3107

ARG_232@O SER_296@H SER_296@N 286 0.2860 2.9253 155.4106

ASP_90@O SER_91@HG SER_91@OG 285 0.2850 2.7537 157.0144

GLU_378@OE2 ARG_386@HH21 ARG_386@NH2 285 0.2850 2.8843 152.6265

GLN_3@O SER_26@H SER_26@N 284 0.2840 2.9270 158.6918

ASP_402@OD1 ARG_404@HH21 ARG_404@NH2 280 0.2800 2.8785 152.8425

ASP_140@OD1 THR_143@H THR_143@N 277 0.2770 2.8454 151.6957

GLU_314@OE2 ARG_216@HH21 ARG_216@NH2 276 0.2760 2.8736 154.3107

ASP_138@OD1 ARG_394@HH22 ARG_394@NH2 276 0.2760 2.8853 152.6285

THR_313@OG1 ASN_318@HD22 ASN_318@ND2 276 0.2760 2.9019 156.9113

ARG_68@O PHE_25@H PHE_25@N 276 0.2760 2.9057 159.1235

SER_296@O ARG_232@H ARG_232@N 276 0.2760 2.9216 159.3325

GLU_279@O SER_267@H SER_267@N 273 0.2730 2.9083 159.6042

ASP_225@OD2 ARG_223@HH21 ARG_223@NH2 272 0.2720 2.8779 156.0239

SER_396@O PHE_399@H PHE_399@N 272 0.2720 2.8971 147.7361

ASN_56@O LEU_59@H LEU_59@N 272 0.2720 2.9037 156.4902

GLU_421@OE1 GLU_421@H GLU_421@N 269 0.2690 2.8347 154.2039

GLU_437@OE2 HIE_406@HE2 HIE_406@NE2 267 0.2670 2.8375 160.4006

ASP_225@OD1 ARG_223@HH21 ARG_223@NH2 267 0.2670 2.8723 155.2672

SER_132@OG GLN_122@HE22 GLN_122@NE2 266 0.2660 2.9038 155.9695

TRP_358@O VAL_365@H VAL_365@N 265 0.2650 2.8869 154.5352

ASP_157@OD1 GLN_374@HE21 GLN_374@NE2 264 0.2640 2.8635 161.0907

GLU_331@OE2 GLU_331@H GLU_331@N 262 0.2620 2.8221 151.7394

GLU_421@OE2 GLU_421@H GLU_421@N 262 0.2620 2.8319 154.5569

GLU_378@OE1 ARG_386@HH21 ARG_386@NH2 262 0.2620 2.8822 152.5485

LEU_416@O THR_431@HG1 THR_431@OG1 259 0.2590 2.8471 162.6881

SER_94@O THR_96@HG1 THR_96@OG1 258 0.2580 2.8178 161.3685

CYX_159@O SER_172@HG SER_172@OG 258 0.2580 2.8226 150.8733

PHE_327@O VAL_343@H VAL_343@N 258 0.2580 2.9215 165.0644

GLU_229@O ASN_231@H ASN_231@N 255 0.2550 2.8689 143.4064

SER_274@OG PHE_275@H PHE_275@N 254 0.2540 2.8833 139.1997

ASN_38@OD1 TRP_40@H TRP_40@N 254 0.2540 2.9038 147.0529

ASP_402@OD2 ARG_404@HH21 ARG_404@NH2 253 0.2530 2.8837 150.7294

TRP_400@O ASP_402@H ASP_402@N 253 0.2530 2.8969 146.4107

VAL_64@O GLU_67@H GLU_67@N 250 0.2500 2.8914 146.8571

PHE_413@O GLN_432@H GLN_432@N 245 0.2450 2.9236 155.5791

THR_103@OG1 GLN_6@H GLN_6@N 243 0.2430 2.9154 155.2610

PRO_194@O THR_197@H THR_197@N 240 0.2400 2.9093 153.6196

ASP_402@OD1 ARG_404@HE ARG_404@NE 235 0.2350 2.8777 153.5670

SER_76@O GLN_78@HE22 GLN_78@NE2 228 0.2280 2.8832 160.1199

CYX_159@O SER_172@H SER_172@N 227 0.2270 2.8924 147.5622

ARG_58@O SER_75@HG SER_75@OG 225 0.2250 2.7730 160.1493

TYR_303@O SER_295@H SER_295@N 225 0.2250 2.9242 154.7883

ASP_352@O VAL_354@H VAL_354@N 223 0.2230 2.8911 144.1377

HIE_366@ND1 SER_367@HG SER_367@OG 222 0.2220 2.8531 159.4160

SER_146@O ASN_190@HD21 ASN_190@ND2 220 0.2200 2.8691 148.0687

ASN_186@OD1 ALA_183@H ALA_183@N 220 0.2200 2.9113 156.5651

ARG_404@O ASN_361@HD21 ASN_361@ND2 219 0.2190 2.8822 158.2184

THR_311@OG1 HIE_353@HE2 HIE_353@NE2 219 0.2190 2.8879 147.5912

GLU_269@OE1 THR_277@HG1 THR_277@OG1 218 0.2180 2.7011 158.1888

GLU_279@OE2 SER_267@HG SER_267@OG 217 0.2170 2.6833 161.8282

LEU_345@O ALA_325@H ALA_325@N 216 0.2160 2.9115 153.3314

ASP_90@O THR_93@H THR_93@N 216 0.2160 2.9225 158.8665

ASP_372@OD1 SER_390@HG SER_390@OG 215 0.2150 2.8053 146.5496

LEU_72@O LEU_21@H LEU_21@N 215 0.2150 2.9213 159.2042

ASP_27@OD1 SER_28@H SER_28@N 214 0.2140 2.8296 145.3696

THR_239@O GLN_242@H GLN_242@N 214 0.2140 2.9157 154.5698

GLU_253@OE1 ASN_252@HD22 ASN_252@ND2 212 0.2120 2.8478 155.1233

GLY_204@O ILE_227@H ILE_227@N 209 0.2090 2.8974 154.2039

GLY_286@O ARG_237@HH22 ARG_237@NH2 207 0.2070 2.8922 151.5289

GLU_418@OE2 ASN_419@HD22 ASN_419@ND2 204 0.2040 2.8484 161.5139

ALA_111@O SER_141@HG SER_141@OG 203 0.2030 2.7437 160.7670

VAL_205@O THR_203@HG1 THR_203@OG1 202 0.2020 2.7230 160.0407

GLU_253@OE2 ASN_252@HD22 ASN_252@ND2 201 0.2010 2.8511 154.4984

ALA_65@O ARG_68@H ARG_68@N 200 0.2000 2.9070 148.3170

VAL_326@O SER_126@HG SER_126@OG 199 0.1990 2.8077 158.6430

ASN_144@OD1 VAL_145@H VAL_145@N 199 0.1990 2.8750 145.5442

ASP_225@OD2 ARG_223@HE ARG_223@NE 199 0.1990 2.8922 158.2519

VAL_360@O LYS_363@H LYS_363@N 199 0.1990 2.9182 156.8093

ASP_402@OD2 ARG_404@H ARG_404@N 197 0.1970 2.9027 159.2911

ASP_384@OD1 TYR_350@HH TYR_350@OH 196 0.1960 2.7006 158.4411

GLU_67@OE2 THR_66@HG1 THR_66@OG1 194 0.1940 2.7212 163.7146

PHE_25@O ARG_68@HE ARG_68@NE 192 0.1920 2.9009 153.5609

ASP_152@OD1 VAL_153@H VAL_153@N 189 0.1890 2.8258 144.0992

GLU_314@O LYS_215@HZ2 LYS_215@NZ 187 0.1870 2.8206 157.3028

ASP_225@OD1 ARG_223@HE ARG_223@NE 185 0.1850 2.8808 157.0478

ASP_130@OD2 SER_128@HG SER_128@OG 183 0.1830 2.6485 162.5864

ASP_125@OD2 LYS_127@H LYS_127@N 183 0.1830 2.8887 160.9970

GLU_314@O LYS_215@HZ1 LYS_215@NZ 182 0.1820 2.8151 157.0139

ALA_388@O LEU_376@H LEU_376@N 182 0.1820 2.9034 154.1322

ILE_334@O GLN_338@H GLN_338@N 181 0.1810 2.8860 144.3248

GLN_379@O LEU_382@H LEU_382@N 180 0.1800 2.9150 157.7738

TYR_387@O GLY_348@H GLY_348@N 178 0.1780 2.8734 144.2797

ASP_140@OD2 GLN_142@H GLN_142@N 178 0.1780 2.8814 149.9605

GLU_257@OE2 LYS_258@H LYS_258@N 176 0.1760 2.8740 150.9401

ALA_111@O ILE_113@H ILE_113@N 175 0.1750 2.8611 143.8278

THR_93@O THR_96@H THR_96@N 175 0.1750 2.9170 150.3920

GLU_355@OE2 TYR_414@HH TYR_414@OH 173 0.1730 2.6884 164.4534

GLU_269@OE2 THR_277@HG1 THR_277@OG1 173 0.1730 2.7263 157.4151

GLU_378@O SER_151@HG SER_151@OG 173 0.1730 2.7437 160.5637

ASP_27@O ARG_68@HH21 ARG_68@NH2 172 0.1720 2.8629 147.3326

THR_337@OG1 LYS_339@H LYS_339@N 172 0.1720 2.9282 152.6855

ASP_125@OD1 SER_128@HG SER_128@OG 170 0.1700 2.7221 162.2506

ILE_334@O GLN_338@HE22 GLN_338@NE2 169 0.1690 2.8653 155.2913

SER_390@O ASP_372@H ASP_372@N 169 0.1690 2.9293 160.9172

ARG_216@O LYS_215@HZ1 LYS_215@NZ 167 0.1670 2.8305 155.7525

GLN_251@O ALA_254@H ALA_254@N 167 0.1670 2.9154 151.1862

ASN_112@OD1 GLN_114@HE22 GLN_114@NE2 164 0.1640 2.8621 159.3306

ASP_152@OD2 VAL_153@H VAL_153@N 162 0.1620 2.8210 143.3044

GLU_418@OE1 ASN_419@HD22 ASN_419@ND2 162 0.1620 2.8472 162.0846

GLU_378@OE1 ARG_386@H ARG_386@N 162 0.1620 2.8891 163.0710

ASP_125@OD2 SER_128@HG SER_128@OG 161 0.1610 2.7339 161.6552

GLU_355@OE1 TYR_414@HH TYR_414@OH 157 0.1570 2.6929 163.9515

GLN_207@OE1 GLY_307@H GLY_307@N 156 0.1560 2.8661 144.2618

THR_156@O GLN_147@HE22 GLN_147@NE2 156 0.1560 2.8833 161.7922

ASP_138@O ASP_117@H ASP_117@N 154 0.1540 2.8867 153.2281

LEU_382@O SER_385@HG SER_385@OG 153 0.1530 2.8117 157.0565

GLU_314@O LYS_215@HZ3 LYS_215@NZ 153 0.1530 2.8224 158.3514

GLU_15@O ASN_112@HD21 ASN_112@ND2 153 0.1530 2.8702 154.0249

ASP_140@O ASN_171@HD22 ASN_171@ND2 153 0.1530 2.8901 148.9304

ASP_402@O ASN_405@H ASN_405@N 153 0.1530 2.9275 160.2939

GLN_122@OE1 CYX_184@H CYX_184@N 150 0.1500 2.8911 155.2444

ASP_140@OD1 GLN_142@H GLN_142@N 149 0.1490 2.8964 149.2967

GLU_67@OE1 THR_66@HG1 THR_66@OG1 148 0.1480 2.7267 165.4847

GLN_424@OE1 THR_423@HG1 THR_423@OG1 143 0.1430 2.7677 157.1193

ARG_216@O LYS_215@HZ2 LYS_215@NZ 143 0.1430 2.8291 154.3510

GLU_257@OE1 LYS_258@H LYS_258@N 141 0.1410 2.8723 150.6905

ASP_10@OD1 ARG_106@HE ARG_106@NE 141 0.1410 2.8945 155.8590

GLN_379@OE1 ALA_381@H ALA_381@N 141 0.1410 2.9041 156.7739

ASP_315@OD2 LYS_317@HZ3 LYS_317@NZ 140 0.1400 2.8288 155.5712

MET_166@O PHE_168@H PHE_168@N 138 0.1380 2.8700 143.3946

ARG_216@O LYS_215@HZ3 LYS_215@NZ 137 0.1370 2.8305 155.5636

THR_62@O SER_70@HG SER_70@OG 136 0.1360 2.7942 148.5637

GLN_207@O ARG_210@HH22 ARG_210@NH2 136 0.1360 2.9051 145.9925

GLN_285@O SER_288@H SER_288@N 135 0.1350 2.9297 158.0271

ASP_181@OD2 LYS_179@HZ2 LYS_179@NZ 134 0.1340 2.8188 155.3739

PHE_182@O GLN_122@HE21 GLN_122@NE2 133 0.1330 2.8902 157.4050

ASP_157@OD2 GLN_374@HE21 GLN_374@NE2 131 0.1310 2.8628 159.7672

GLY_241@O ARG_106@HH21 ARG_106@NH2 131 0.1310 2.8698 155.6128

SER_191@O ILE_193@H ILE_193@N 129 0.1290 2.8953 146.5580

LEU_261@O SER_263@H SER_263@N 128 0.1280 2.8746 143.5690

SER_148@OG ILE_155@H ILE_155@N 128 0.1280 2.9300 159.4537

ASN_318@OD1 LYS_317@HZ3 LYS_317@NZ 127 0.1270 2.8432 158.0715

GLN_424@O ARG_426@H ARG_426@N 127 0.1270 2.8811 147.7578

ILE_155@O SER_148@H SER_148@N 127 0.1270 2.9106 159.6582

ASP_181@OD2 LYS_179@HZ1 LYS_179@NZ 126 0.1260 2.8167 156.2687

ASP_315@OD2 LYS_317@HZ1 LYS_317@NZ 126 0.1260 2.8240 156.5829

ASP_315@OD1 LYS_317@HZ2 LYS_317@NZ 125 0.1250 2.8278 154.8742

GLU_279@OE1 GLN_281@HE22 GLN_281@NE2 125 0.1250 2.8515 159.5119

ASN_186@O ASN_189@H ASN_189@N 125 0.1250 2.9226 157.0430

PRO_50@O SER_51@HG SER_51@OG 123 0.1230 2.7130 157.2237

ASP_181@OD2 LYS_179@HZ3 LYS_179@NZ 123 0.1230 2.8234 154.8801

THR_103@O ARG_106@HH22 ARG_106@NH2 123 0.1230 2.8899 149.7705

GLU_279@OE2 GLN_281@HE22 GLN_281@NE2 122 0.1220 2.8631 159.5536

ASN_318@OD1 LYS_317@HZ1 LYS_317@NZ 120 0.1200 2.8230 157.7393

GLU_333@OE1 THR_341@HG1 THR_341@OG1 120 0.1200 2.8846 150.9397

TYR_73@OH ASN_18@HD22 ASN_18@ND2 120 0.1200 2.9002 153.4700

ASP_402@OD1 ARG_404@H ARG_404@N 120 0.1200 2.9006 158.0553

GLN_242@OE1 THR_239@HG1 THR_239@OG1 119 0.1190 2.7345 159.8044

VAL_109@O THR_79@HG1 THR_79@OG1 119 0.1190 2.7589 161.8705

ASP_315@OD1 ASN_318@HD21 ASN_318@ND2 119 0.1190 2.8275 153.0538

GLN_285@OE1 ASN_383@HD21 ASN_383@ND2 119 0.1190 2.8863 157.3648

THR_103@O ARG_106@HH12 ARG_106@NH1 119 0.1190 2.9080 149.3134

PHE_413@O GLN_412@HE22 GLN_412@NE2 119 0.1190 2.9177 156.1933

TYR_48@O LYS_54@HZ1 LYS_54@NZ 117 0.1170 2.8314 153.4700

THR_203@O TYR_303@HH TYR_303@OH 115 0.1150 2.7513 162.3500

ASP_315@OD1 LYS_317@HZ1 LYS_317@NZ 115 0.1150 2.8182 153.6052

ASP_420@O LYS_428@HZ3 LYS_428@NZ 115 0.1150 2.8282 156.5484

ASP_315@O ASN_318@H ASN_318@N 115 0.1150 2.9239 160.9452

ASN_318@OD1 LYS_317@HZ2 LYS_317@NZ 114 0.1140 2.8274 158.4053

GLN_251@OE1 ARG_232@HE ARG_232@NE 114 0.1140 2.8650 150.4124

ASP_162@OD1 ARG_164@HH12 ARG_164@NH1 114 0.1140 2.8692 153.9179

THR_431@OG1 GLY_415@H GLY_415@N 114 0.1140 2.9114 153.1703

ASP_420@O LYS_428@HZ1 LYS_428@NZ 113 0.1130 2.8386 154.3358

ASN_144@OD1 GLN_147@HE21 GLN_147@NE2 113 0.1130 2.8829 158.8889

SER_172@OG ARG_392@HH21 ARG_392@NH2 113 0.1130 2.9172 145.7488

ASP_315@O ASN_318@HD22 ASN_318@ND2 112 0.1120 2.8525 153.8792

ASP_130@OD1 SER_128@HG SER_128@OG 111 0.1110 2.6500 163.0190

ASP_27@OD2 SER_26@HG SER_26@OG 111 0.1110 2.6931 165.1131

GLN_42@OE1 ASN_38@HD21 ASN_38@ND2 110 0.1100 2.8853 162.6752

GLU_364@OE2 LYS_363@HZ3 LYS_363@NZ 109 0.1090 2.8304 153.9840

GLU_378@OE2 ARG_386@H ARG_386@N 109 0.1090 2.8704 162.7831

GLY_1@O GLN_3@HE21 GLN_3@NE2 108 0.1080 2.8783 155.4574

PRO_306@O GLN_42@HE21 GLN_42@NE2 108 0.1080 2.8918 159.9877

SER_396@O TRP_400@H TRP_400@N 108 0.1080 2.9324 155.8910

CYX_409@O SER_435@HG SER_435@OG 107 0.1070 2.7402 162.4398

ASP_420@O LYS_428@HZ2 LYS_428@NZ 107 0.1070 2.8263 154.4361

ALA_92@O SER_94@H SER_94@N 107 0.1070 2.8917 143.2483

ASP_181@OD1 LYS_179@HZ3 LYS_179@NZ 106 0.1060 2.8094 155.5870

GLU_437@OE2 LYS_127@HZ2 LYS_127@NZ 105 0.1050 2.7998 154.2700

ASP_315@OD2 LYS_317@HZ2 LYS_317@NZ 105 0.1050 2.8117 155.9490

LYS_317@O ARG_426@HH22 ARG_426@NH2 105 0.1050 2.8649 147.5659

ASP_181@OD1 LYS_179@HZ2 LYS_179@NZ 104 0.1040 2.8236 154.2861

GLU_314@OE2 GLN_285@HE22 GLN_285@NE2 104 0.1040 2.8590 155.3946

ASP_10@OD1 ARG_106@HH21 ARG_106@NH2 104 0.1040 2.8963 150.8773

ASP_352@OD1 ASP_352@H ASP_352@N 103 0.1030 2.7648 138.7180

ASP_315@OD1 LYS_317@HZ3 LYS_317@NZ 102 0.1020 2.8217 153.0723

LYS_179@O PHE_182@H PHE_182@N 102 0.1020 2.9215 157.8853

TYR_48@O LYS_54@HZ2 LYS_54@NZ 100 0.1000 2.8315 153.6099

TYR_48@O LYS_54@HZ3 LYS_54@NZ 99 0.0990 2.8216 154.4662

ASP_315@OD2 ASN_318@HD21 ASN_318@ND2 99 0.0990 2.8277 153.4101

GLU_269@OE2 ARG_270@H ARG_270@N 99 0.0990 2.8423 158.1159

GLU_364@OE1 LYS_363@HZ1 LYS_363@NZ 99 0.0990 2.8423 154.6800

ASP_27@OD1 SER_26@HG SER_26@OG 98 0.0980 2.7002 165.1901

ASP_181@OD1 LYS_179@HZ1 LYS_179@NZ 98 0.0980 2.8169 153.2546

PHE_199@O SER_201@H SER_201@N 98 0.0980 2.8800 146.0680

SER_330@OG GLU_333@H GLU_333@N 98 0.0980 2.9303 156.7934

GLU_364@OE1 LYS_363@HZ3 LYS_363@NZ 96 0.0960 2.8275 156.2383

THR_79@O SER_82@H SER_82@N 94 0.0940 2.9279 155.5144

GLU_437@OE2 LYS_127@HZ1 LYS_127@NZ 93 0.0930 2.7985 153.7243

ASP_157@OD1 LYS_158@HZ2 LYS_158@NZ 93 0.0930 2.8136 159.2102

GLU_364@OE1 LYS_363@HZ2 LYS_363@NZ 92 0.0920 2.8386 153.6318

SER_417@O ASP_420@H ASP_420@N 92 0.0920 2.9302 160.2284

PHE_139@O ASN_171@HD22 ASN_171@ND2 91 0.0910 2.8921 159.4480

GLU_364@OE2 LYS_363@HZ2 LYS_363@NZ 90 0.0900 2.8203 156.4764

ASP_10@OD2 ARG_106@HH21 ARG_106@NH2 90 0.0900 2.8891 153.1033

ILE_213@O LYS_212@HZ3 LYS_212@NZ 89 0.0890 2.8378 152.5486

GLU_364@OE2 LYS_363@HZ1 LYS_363@NZ 89 0.0890 2.8384 154.3264

ASP_443@OD1 ARG_441@HH21 ARG_441@NH2 89 0.0890 2.8634 151.2134

ARG_68@O ASN_24@HD22 ASN_24@ND2 89 0.0890 2.8655 153.6446

ASP_162@OD2 ARG_164@HH12 ARG_164@NH1 89 0.0890 2.8765 152.9295

ASP_162@OD1 ARG_164@HH22 ARG_164@NH2 88 0.0880 2.8756 150.2981

LYS_212@O THR_311@HG1 THR_311@OG1 87 0.0870 2.8102 152.9557

THR_221@O THR_221@HG1 THR_221@OG1 87 0.0870 2.8272 141.9983

ASN_171@OD1 SER_172@H SER_172@N 87 0.0870 2.8990 140.9448

ASP_181@OD2 ASP_181@H ASP_181@N 86 0.0860 2.8174 139.7038

GLU_245@OE2 PHE_246@H PHE_246@N 86 0.0860 2.8583 151.8634

ASP_352@OD1 ARG_309@HH22 ARG_309@NH2 86 0.0860 2.8781 151.3246

SER_330@O GLU_333@H GLU_333@N 86 0.0860 2.8955 147.1813

SER_177@O LYS_179@H LYS_179@N 86 0.0860 2.9002 143.8428

ASP_130@OD2 LYS_131@HZ2 LYS_131@NZ 85 0.0850 2.8016 156.3536

GLU_437@OE1 LYS_127@HZ1 LYS_127@NZ 85 0.0850 2.8079 154.9291

GLU_245@OE1 PHE_246@H PHE_246@N 85 0.0850 2.8570 151.9292

GLY_57@O GLN_55@HE22 GLN_55@NE2 85 0.0850 2.8699 156.3096

ASP_157@OD1 LYS_158@HZ1 LYS_158@NZ 84 0.0840 2.7926 156.6427

ASP_130@OD2 LYS_131@HZ3 LYS_131@NZ 84 0.0840 2.8151 156.5777

ASP_352@OD2 ARG_309@HH12 ARG_309@NH1 84 0.0840 2.8886 154.4908

SER_77@OG SER_19@HG SER_19@OG 82 0.0820 2.8685 159.3501

LEU_46@O ASN_56@HD22 ASN_56@ND2 82 0.0820 2.8784 153.1007

ASP_140@OD1 GLN_142@HE22 GLN_142@NE2 81 0.0810 2.8797 155.7336

PRO_9@O ARG_106@HH11 ARG_106@NH1 81 0.0810 2.8981 157.2494

GLU_437@OE2 LYS_127@HZ3 LYS_127@NZ 80 0.0800 2.8196 153.5987

ILE_213@O LYS_212@HZ1 LYS_212@NZ 80 0.0800 2.8463 151.8933

ASP_140@OD2 GLN_142@HE22 GLN_142@NE2 80 0.0800 2.8901 155.3267

ASP_130@OD1 LYS_131@HZ3 LYS_131@NZ 79 0.0790 2.7841 155.6133

VAL_64@O ARG_68@H ARG_68@N 79 0.0790 2.8841 145.1700

ASP_157@OD1 LYS_158@HZ3 LYS_158@NZ 78 0.0780 2.8068 158.8646

ASP_157@OD2 LYS_158@HZ3 LYS_158@NZ 78 0.0780 2.8225 156.9822

LEU_247@O THR_248@HG1 THR_248@OG1 77 0.0770 2.7786 158.6301

ASP_157@OD2 LYS_158@HZ1 LYS_158@NZ 77 0.0770 2.8122 157.3343

ASN_144@O ILE_192@H ILE_192@N 77 0.0770 2.9085 157.6390

SER_259@O LEU_262@H LEU_262@N 77 0.0770 2.9168 152.8553

GLU_437@OE1 LYS_127@HZ2 LYS_127@NZ 76 0.0760 2.8061 153.6605

GLN_424@OE1 ARG_426@HE ARG_426@NE 75 0.0750 2.8780 149.1119

ASP_130@OD1 LYS_131@HZ1 LYS_131@NZ 74 0.0740 2.8130 155.3071

THR_337@O LYS_339@HZ3 LYS_339@NZ 74 0.0740 2.8543 153.9202

ALA_65@O ARG_68@HH11 ARG_68@NH1 74 0.0740 2.8974 152.6339

THR_63@O GLY_52@H GLY_52@N 74 0.0740 2.9016 149.7456

LEU_310@O ALA_289@H ALA_289@N 74 0.0740 2.9301 145.6531

GLU_15@OE1 ASN_112@H ASN_112@N 73 0.0730 2.8725 162.0894

ASN_38@OD1 GLY_41@H GLY_41@N 73 0.0730 2.8789 146.9241

GLN_251@OE1 ARG_232@HH21 ARG_232@NH2 73 0.0730 2.8815 147.8997

ASP_157@OD2 LYS_158@HZ2 LYS_158@NZ 72 0.0720 2.8025 157.3108

ASP_420@OD2 LYS_212@HZ1 LYS_212@NZ 72 0.0720 2.8239 154.0694

THR_337@O LYS_339@HZ1 LYS_339@NZ 72 0.0720 2.8514 153.7237

GLY_298@O ARG_232@HH11 ARG_232@NH1 72 0.0720 2.8638 158.0326

ALA_187@O ASN_190@HD21 ASN_190@ND2 72 0.0720 2.8710 147.0462

ASP_443@OD1 ARG_441@HE ARG_441@NE 72 0.0720 2.8867 155.3503

ASP_443@OD2 ARG_441@HH21 ARG_441@NH2 72 0.0720 2.8867 150.8743

LEU_247@O SER_259@HG SER_259@OG 71 0.0710 2.7820 161.6303

ASP_196@OD1 ASP_196@H ASP_196@N 71 0.0710 2.7914 139.0240

ILE_213@O LYS_212@HZ2 LYS_212@NZ 71 0.0710 2.8427 151.0951

GLU_15@OE1 ASN_112@HD22 ASN_112@ND2 71 0.0710 2.8472 151.6166

GLU_314@OE1 GLN_285@HE22 GLN_285@NE2 71 0.0710 2.8676 156.1513

GLN_218@OE1 LYS_215@H LYS_215@N 71 0.0710 2.8999 165.1828

GLU_279@OE1 SER_267@HG SER_267@OG 70 0.0700 2.7021 162.5870

ASP_225@O SER_206@HG SER_206@OG 70 0.0700 2.8139 155.5990

ASP_130@OD2 LYS_131@HZ1 LYS_131@NZ 70 0.0700 2.8177 154.9277

ASN_24@OD1 ARG_22@HE ARG_22@NE 70 0.0700 2.8765 156.2577

ASP_443@OD2 ARG_441@HE ARG_441@NE 70 0.0700 2.8780 155.5999

TYR_97@O TYR_99@H TYR_99@N 70 0.0700 2.9051 142.9949

ASN_190@O SER_146@HG SER_146@OG 69 0.0690 2.7726 159.2770

GLU_229@OE2 SER_228@HG SER_228@OG 67 0.0670 2.7116 160.3766

GLU_229@OE1 SER_228@HG SER_228@OG 67 0.0670 2.7684 158.8534

ALA_111@O GLN_14@HE22 GLN_14@NE2 67 0.0670 2.8870 159.4574

ASP_10@OD2 ARG_106@HE ARG_106@NE 66 0.0660 2.8655 156.4842

LYS_98@O SER_91@HG SER_91@OG 65 0.0650 2.8004 155.7537

GLU_437@OE1 LYS_127@HZ3 LYS_127@NZ 65 0.0650 2.8043 152.7487

GLN_147@OE1 GLN_147@H GLN_147@N 65 0.0650 2.8588 149.0105

TYR_249@OH ARG_232@HH11 ARG_232@NH1 65 0.0650 2.9140 149.2890

ASP_162@OD2 ARG_164@HH22 ARG_164@NH2 64 0.0640 2.8882 150.8328

ASP_130@OD1 LYS_131@HZ2 LYS_131@NZ 63 0.0630 2.8084 154.4971

ASP_150@OD1 LYS_149@HZ2 LYS_149@NZ 63 0.0630 2.8102 156.9418

ASP_420@OD2 LYS_212@HZ2 LYS_212@NZ 63 0.0630 2.8151 154.3106

ASP_420@OD2 LYS_212@HZ3 LYS_212@NZ 63 0.0630 2.8178 156.2280

GLU_418@O LYS_428@HZ3 LYS_428@NZ 63 0.0630 2.8306 149.5420

ASP_167@OD2 LYS_169@HZ2 LYS_169@NZ 62 0.0620 2.8076 154.8572

ASP_181@OD1 ASP_181@H ASP_181@N 62 0.0620 2.8228 139.0601

ALA_61@O THR_62@HG1 THR_62@OG1 62 0.0620 2.8585 157.7327

SER_330@O ILE_334@H ILE_334@N 62 0.0620 2.9428 165.3990

ASP_167@OD1 LYS_169@HZ1 LYS_169@NZ 61 0.0610 2.8165 155.7739

ASP_150@OD1 LYS_149@HZ1 LYS_149@NZ 61 0.0610 2.8173 157.1060

VAL_220@O THR_221@HG1 THR_221@OG1 61 0.0610 2.8204 155.4866

GLU_15@OE2 ASN_112@HD22 ASN_112@ND2 61 0.0610 2.8632 154.5296

ILE_213@O THR_214@HG1 THR_214@OG1 61 0.0610 2.8650 155.4837

SER_296@OG GLY_299@H GLY_299@N 61 0.0610 2.9113 143.7699

ASP_125@O SER_128@H SER_128@N 61 0.0610 2.9182 157.5872

ASP_352@OD2 ASP_352@H ASP_352@N 60 0.0600 2.7683 139.2997

THR_103@O THR_103@HG1 THR_103@OG1 60 0.0600 2.7818 142.0615

ASN_231@OD1 GLN_251@HE21 GLN_251@NE2 60 0.0600 2.8410 154.1414

ASP_352@OD2 ARG_309@HH22 ARG_309@NH2 60 0.0600 2.8892 150.7563

SER_177@OG LYS_179@H LYS_179@N 60 0.0600 2.9406 159.1118

ASP_167@OD2 LYS_169@HZ1 LYS_169@NZ 59 0.0590 2.8172 154.8813

VAL_145@O GLN_147@HE21 GLN_147@NE2 59 0.0590 2.8551 146.4524

THR_337@O LYS_339@HZ2 LYS_339@NZ 59 0.0590 2.8554 152.2925

GLU_245@OE2 ARG_237@HE ARG_237@NE 59 0.0590 2.8703 154.5638

ASP_150@OD1 LYS_149@HZ3 LYS_149@NZ 58 0.0580 2.7999 158.6470

ASP_125@OD1 SER_128@H SER_128@N 58 0.0580 2.8504 151.6979

TRP_439@O ARG_441@HH11 ARG_441@NH1 58 0.0580 2.8732 156.1258

ARG_441@O ASP_443@H ASP_443@N 58 0.0580 2.8809 145.3005

ASN_24@OD1 ARG_22@HH11 ARG_22@NH1 58 0.0580 2.8851 152.9869

GLN_147@OE1 ASN_144@HD21 ASN_144@ND2 58 0.0580 2.8859 160.3170

ASN_252@OD1 ASN_231@HD21 ASN_231@ND2 57 0.0570 2.8599 160.1171

VAL_4@O THR_103@H THR_103@N 57 0.0570 2.9470 152.0132

ASP_130@OD2 ASP_130@H ASP_130@N 56 0.0560 2.8152 138.8768

ASN_252@OD1 GLN_251@HE22 GLN_251@NE2 56 0.0560 2.8797 159.5885

LEU_297@O ASN_231@HD21 ASN_231@ND2 56 0.0560 2.8873 149.1664

ASP_167@OD1 LYS_169@HZ3 LYS_169@NZ 55 0.0550 2.8091 156.8683

ASP_150@OD2 SER_151@H SER_151@N 55 0.0550 2.8300 146.2479

ASN_112@OD1 GLN_14@HE22 GLN_14@NE2 55 0.0550 2.8732 156.0936

GLY_298@O TYR_300@H TYR_300@N 55 0.0550 2.9132 144.3283

GLU_418@O LYS_428@HZ1 LYS_428@NZ 54 0.0540 2.8482 151.0227

ASP_117@OD1 HIE_336@HE2 HIE_336@NE2 54 0.0540 2.8552 155.3587

ASP_167@OD2 LYS_169@HZ3 LYS_169@NZ 53 0.0530 2.7947 157.4732

ASP_167@OD1 LYS_169@HZ2 LYS_169@NZ 53 0.0530 2.8162 156.3711

ASP_443@OD1 ARG_441@HH12 ARG_441@NH1 53 0.0530 2.8640 154.8648

ASP_140@O THR_143@HG1 THR_143@OG1 52 0.0520 2.7157 161.0514

ARG_260@O SER_263@HG SER_263@OG 52 0.0520 2.7448 158.7397

ASP_117@OD2 HIE_336@HE2 HIE_336@NE2 52 0.0520 2.8452 156.5783

ASP_352@OD1 ARG_309@HH12 ARG_309@NH1 52 0.0520 2.8798 150.3108

LEU_123@O SER_132@HG SER_132@OG 51 0.0510 2.7149 158.7432

ASP_425@OD1 ASP_425@H ASP_425@N 51 0.0510 2.7686 139.5170

MET_163@O ARG_164@HH11 ARG_164@NH1 51 0.0510 2.8854 150.6591

ASP_443@O ARG_441@HE ARG_441@NE 50 0.0500 2.8768 155.1512

TRP_40@O GLN_42@HE22 GLN_42@NE2 50 0.0500 2.8830 161.5828

SER_295@O TYR_303@H TYR_303@N 50 0.0500 2.9397 163.4796

GLU_418@O LYS_428@HZ2 LYS_428@NZ 49 0.0490 2.8428 153.7700

GLN_14@OE1 GLN_114@HE22 GLN_114@NE2 48 0.0480 2.8894 155.6321

SER_76@O ARG_58@HH11 ARG_58@NH1 48 0.0480 2.8896 144.2113

ASP_425@OD2 ASP_425@H ASP_425@N 47 0.0470 2.8037 139.3990

ALA_332@O SER_335@HG SER_335@OG 47 0.0470 2.8073 153.0940

GLU_5@OE2 GLN_3@HE21 GLN_3@NE2 47 0.0470 2.8292 155.7118

ASN_112@O LYS_169@HZ1 LYS_169@NZ 47 0.0470 2.8519 151.9526

GLN_218@OE1 ASN_219@H ASN_219@N 47 0.0470 2.8929 158.3213

GLU_5@OE2 SER_7@HG SER_7@OG 46 0.0460 2.6835 161.0891

ASP_420@OD1 LYS_212@HZ1 LYS_212@NZ 46 0.0460 2.8214 151.6614

ASN_112@O LYS_169@HZ2 LYS_169@NZ 46 0.0460 2.8222 153.2139

GLU_437@O LYS_127@HZ3 LYS_127@NZ 46 0.0460 2.8376 147.8026

ASP_372@OD1 ARG_392@HH22 ARG_392@NH2 46 0.0460 2.8848 150.9544

ALA_397@O GLN_401@HE22 GLN_401@NE2 46 0.0460 2.9068 157.0731

ASP_90@OD1 ASN_31@H ASN_31@N 46 0.0460 2.9068 148.8215

SER_7@OG ARG_22@HH11 ARG_22@NH1 46 0.0460 2.9408 155.5648

GLU_5@OE2 ARG_22@HH12 ARG_22@NH1 45 0.0450 2.8456 153.1631

SER_191@OG SER_146@HG SER_146@OG 45 0.0450 2.8598 157.6764

ILE_113@O GLN_14@HE21 GLN_14@NE2 45 0.0450 2.8737 157.4540

THR_53@OG1 GLN_55@HE21 GLN_55@NE2 45 0.0450 2.9076 150.9787

PHE_413@O GLY_415@H GLY_415@N 45 0.0450 2.9166 146.3676

ASP_196@OD2 ASP_196@H ASP_196@N 44 0.0440 2.7910 139.5184

ARG_441@O GLN_401@HE22 GLN_401@NE2 44 0.0440 2.8699 158.9004

ASP_443@O ARG_441@HH21 ARG_441@NH2 44 0.0440 2.8872 149.7952

GLN_3@OE1 SER_26@HG SER_26@OG 43 0.0430 2.7291 158.3482

GLU_378@O GLN_379@HE22 GLN_379@NE2 43 0.0430 2.8975 154.8247

ASP_90@OD1 SER_94@HG SER_94@OG 42 0.0420 2.7534 158.8107

ASP_130@OD1 ASP_130@H ASP_130@N 42 0.0420 2.7977 138.7284

ASP_420@OD1 LYS_212@HZ2 LYS_212@NZ 42 0.0420 2.8420 154.0905

LEU_310@O THR_311@HG1 THR_311@OG1 42 0.0420 2.8454 161.9821

ASP_125@OD2 LYS_127@HZ1 LYS_127@NZ 42 0.0420 2.8552 154.2332

GLU_15@OE2 ASN_112@H ASN_112@N 42 0.0420 2.8598 163.4674

ASN_112@O GLN_114@HE22 GLN_114@NE2 42 0.0420 2.8928 160.7737

GLN_424@OE1 THR_423@H THR_423@N 42 0.0420 2.9092 155.2829

SER_274@O ARG_270@HH21 ARG_270@NH2 42 0.0420 2.9182 144.8620

GLN_142@OE1 SER_141@HG SER_141@OG 41 0.0410 2.7293 160.0354

LYS_54@O SER_60@HG SER_60@OG 41 0.0410 2.7523 159.5761

GLU_5@OE1 GLN_3@HE21 GLN_3@NE2 41 0.0410 2.8304 160.4515

GLN_424@OE1 ARG_426@HH21 ARG_426@NH2 41 0.0410 2.8627 147.9186

SER_77@O GLN_78@HE22 GLN_78@NE2 41 0.0410 2.8670 153.3813

THR_66@O ARG_68@HH11 ARG_68@NH1 41 0.0410 2.8752 150.5735

ARG_265@O GLN_281@HE22 GLN_281@NE2 41 0.0410 2.9094 154.3249

GLU_437@OE2 GLU_437@H GLU_437@N 40 0.0400 2.8024 147.0859

SER_385@O LYS_377@HZ2 LYS_377@NZ 40 0.0400 2.8461 147.8946

ASP_264@OD1 ARG_265@H ARG_265@N 40 0.0400 2.8463 149.0634

PHE_25@O ARG_68@HH11 ARG_68@NH1 40 0.0400 2.8622 151.9789

PRO_375@O GLN_374@HE22 GLN_374@NE2 40 0.0400 2.9010 161.8055

SER_385@OG GLN_379@H GLN_379@N 40 0.0400 2.9313 152.1250

VAL_64@O TYR_69@H TYR_69@N 40 0.0400 2.9362 164.1000

GLU_437@O LYS_127@HZ2 LYS_127@NZ 39 0.0390 2.8176 149.3631

SER_94@O THR_96@H THR_96@N 39 0.0390 2.8749 143.2217

ASP_443@OXT ARG_441@HE ARG_441@NE 39 0.0390 2.8814 154.7597

ASN_361@O ARG_408@HH21 ARG_408@NH2 39 0.0390 2.8816 148.1212

ALA_397@O TRP_400@H TRP_400@N 39 0.0390 2.9043 144.0482

ASP_117@OD2 ASP_117@H ASP_117@N 38 0.0380 2.7890 139.4668

ASP_420@OD1 LYS_212@HZ3 LYS_212@NZ 38 0.0380 2.8165 152.4017

ASN_144@OD1 LYS_158@HZ3 LYS_158@NZ 38 0.0380 2.8248 151.6377

THR_62@OG1 THR_53@HG1 THR_53@OG1 38 0.0380 2.8257 160.4813

GLN_424@OE1 GLN_424@H GLN_424@N 38 0.0380 2.8581 147.7495

GLU_15@OE1 GLN_78@HE21 GLN_78@NE2 38 0.0380 2.8600 158.4571

THR_423@O GLN_424@HE22 GLN_424@NE2 38 0.0380 2.8737 156.6262

GLU_421@OE2 TRP_422@H TRP_422@N 38 0.0380 2.8863 151.8126

SER_385@O LYS_377@HZ1 LYS_377@NZ 37 0.0370 2.8193 148.6338

GLU_257@OE2 ARG_260@HH21 ARG_260@NH2 37 0.0370 2.8712 153.3055

GLN_410@OE1 ARG_408@HE ARG_408@NE 37 0.0370 2.8940 153.5287

GLY_217@O ARG_282@HH11 ARG_282@NH1 37 0.0370 2.8943 156.4170

ASP_315@OD1 LYS_317@H LYS_317@N 37 0.0370 2.9051 165.2248

GLN_55@OE1 SER_60@HG SER_60@OG 36 0.0360 2.7303 159.2641

VAL_153@O SER_148@HG SER_148@OG 36 0.0360 2.7536 160.3368

ASN_144@OD1 LYS_158@HZ2 LYS_158@NZ 36 0.0360 2.8322 152.2985

ASN_112@O LYS_169@HZ3 LYS_169@NZ 36 0.0360 2.8386 151.7657

GLY_217@O ARG_282@HE ARG_282@NE 36 0.0360 2.8705 149.8520

GLU_5@OE2 ASN_24@HD22 ASN_24@ND2 36 0.0360 2.8738 160.5297

ASP_443@OXT ARG_441@HH21 ARG_441@NH2 36 0.0360 2.8747 151.7682

GLU_301@OE2 LEU_297@H LEU_297@N 36 0.0360 2.8936 150.7397

SER_141@OG GLN_14@HE22 GLN_14@NE2 36 0.0360 2.9074 151.6553

THR_283@OG1 GLY_217@H GLY_217@N 36 0.0360 2.9344 149.8191

GLU_437@OE1 GLU_437@H GLU_437@N 35 0.0350 2.8403 144.6793

ASP_443@OD2 ARG_441@HH12 ARG_441@NH1 35 0.0350 2.8646 153.6450

ARG_260@O LEU_262@H LEU_262@N 35 0.0350 2.8823 145.4242

SER_335@O GLN_338@HE21 GLN_338@NE2 35 0.0350 2.8865 147.8610

ASP_162@OD1 ARG_164@HH21 ARG_164@NH2 35 0.0350 2.8895 153.0484

SER_172@O ARG_392@HH21 ARG_392@NH2 35 0.0350 2.8916 145.1888

ALA_111@O GLN_14@HE21 GLN_14@NE2 35 0.0350 2.8960 151.3467

PHE_25@O ASP_27@H ASP_27@N 35 0.0350 2.8983 143.9600

GLU_437@O LYS_127@HZ1 LYS_127@NZ 34 0.0340 2.8092 147.1675

SER_385@O LYS_377@HZ3 LYS_377@NZ 34 0.0340 2.8282 145.6364

SER_259@O LYS_258@HZ3 LYS_258@NZ 34 0.0340 2.8346 152.4592

ASN_144@OD1 LYS_158@HZ1 LYS_158@NZ 34 0.0340 2.8523 153.3505

ASP_150@OD1 SER_151@H SER_151@N 34 0.0340 2.8573 148.9383

ASP_162@OD2 ARG_164@HE ARG_164@NE 34 0.0340 2.8621 152.1497

SER_417@OG ASN_419@H ASN_419@N 34 0.0340 2.9341 147.1157

PHE_266@O SER_267@HG SER_267@OG 33 0.0330 2.7728 154.7600

ASP_150@OD2 LYS_149@HZ2 LYS_149@NZ 33 0.0330 2.8495 157.4757

ASP_125@OD1 LYS_127@HZ2 LYS_127@NZ 33 0.0330 2.8651 150.5706

SER_128@O ARG_124@HH21 ARG_124@NH2 33 0.0330 2.8678 144.7900

GLN_302@O ASN_45@HD22 ASN_45@ND2 33 0.0330 2.8823 145.7043

ASN_24@OD1 ARG_22@HH21 ARG_22@NH2 33 0.0330 2.8893 146.4022

GLU_257@OE2 ARG_260@HH12 ARG_260@NH1 33 0.0330 2.8925 152.2281

SER_141@OG ASN_171@HD21 ASN_171@ND2 33 0.0330 2.9069 158.4180

ASP_315@OD2 LYS_317@H LYS_317@N 33 0.0330 2.9181 162.8851

ASP_117@OD1 ASP_117@H ASP_117@N 32 0.0320 2.7888 139.1845

ASP_150@OD2 LYS_149@HZ3 LYS_149@NZ 32 0.0320 2.7941 160.1652

THR_53@OG1 THR_62@HG1 THR_62@OG1 32 0.0320 2.8358 158.6436

GLU_269@OE1 ARG_270@H ARG_270@N 32 0.0320 2.8516 155.5156

GLU_245@OE2 ARG_237@HH21 ARG_237@NH2 32 0.0320 2.8806 148.0230

GLN_6@OE1 GLY_104@H GLY_104@N 32 0.0320 2.9256 141.7725

GLU_333@O HIE_336@H HIE_336@N 32 0.0320 2.9539 145.6941

ASP_138@OD1 SER_170@HG SER_170@OG 31 0.0310 2.6274 163.2307

THR_423@O THR_423@HG1 THR_423@OG1 31 0.0310 2.7804 140.4959

GLU_301@OE2 GLU_301@H GLU_301@N 31 0.0310 2.7995 151.1511

GLY_415@O THR_431@HG1 THR_431@OG1 31 0.0310 2.8008 148.3758

ASN_186@O ASN_189@HD22 ASN_189@ND2 31 0.0310 2.8487 161.0787

GLY_95@O ARG_232@HH12 ARG_232@NH1 31 0.0310 2.8630 147.7297

THR_53@O THR_53@HG1 THR_53@OG1 31 0.0310 2.8643 142.3317

ASP_443@OD1 ARG_441@HH11 ARG_441@NH1 31 0.0310 2.8658 151.9063

GLU_331@OE2 ARG_441@HH12 ARG_441@NH1 31 0.0310 2.8697 154.3262

SER_335@O GLN_338@HE22 GLN_338@NE2 31 0.0310 2.8739 149.0206

GLU_5@OE1 ARG_22@HH22 ARG_22@NH2 31 0.0310 2.8891 153.8665

ASP_125@OD2 SER_128@H SER_128@N 31 0.0310 2.8895 153.2721

SER_390@OG ARG_392@HH11 ARG_392@NH1 31 0.0310 2.9025 140.3631

PRO_200@O SER_201@HG SER_201@OG 30 0.0300 2.7829 156.0781

ILE_155@O SER_148@HG SER_148@OG 30 0.0300 2.7841 152.5826

ASP_425@OD2 ARG_426@H ARG_426@N 30 0.0300 2.8217 140.4664

GLU_284@OE2 ARG_216@HH11 ARG_216@NH1 30 0.0300 2.8761 150.8636

ASP_162@OD2 ARG_164@HH21 ARG_164@NH2 30 0.0300 2.8785 147.7190

GLU_229@O ASN_231@HD22 ASN_231@ND2 30 0.0300 2.8827 152.4479

ASP_81@OD1 THR_80@HG1 THR_80@OG1 29 0.0290 2.8183 154.5908

ASP_443@OD1 GLN_401@HE21 GLN_401@NE2 29 0.0290 2.8367 157.9012

GLY_217@O ARG_282@HH21 ARG_282@NH2 29 0.0290 2.8776 148.1301

ASN_231@OD1 ARG_232@HE ARG_232@NE 29 0.0290 2.8870 148.0572

LEU_310@O ARG_309@HH11 ARG_309@NH1 29 0.0290 2.9068 152.7928

LEU_240@O GLN_242@HE22 GLN_242@NE2 29 0.0290 2.9130 160.2339

PHE_199@O SER_201@HG SER_201@OG 28 0.0280 2.8270 157.6309

ASP_425@OD1 ARG_426@H ARG_426@N 28 0.0280 2.8414 142.5016

ASP_125@OD2 LYS_127@HZ2 LYS_127@NZ 28 0.0280 2.8686 157.5426

ASN_231@OD1 ARG_232@HH11 ARG_232@NH1 28 0.0280 2.8993 153.5977

ASP_140@O THR_143@H THR_143@N 28 0.0280 2.9254 160.0460

GLY_16@O SER_76@HG SER_76@OG 27 0.0270 2.7975 156.1406

ASP_125@OD2 LYS_127@HZ3 LYS_127@NZ 27 0.0270 2.8128 154.9906

GLN_410@OE1 ARG_408@HH21 ARG_408@NH2 27 0.0270 2.8973 145.9044

SER_128@O LYS_131@H LYS_131@N 27 0.0270 2.9204 161.5488

ASP_264@OD2 ASP_264@H ASP_264@N 26 0.0260 2.8123 138.5675

THR_53@O LYS_54@HZ2 LYS_54@NZ 26 0.0260 2.8139 159.8074

ASP_372@O THR_371@HG1 THR_371@OG1 26 0.0260 2.8204 162.3668

THR_143@O LYS_158@HZ1 LYS_158@NZ 26 0.0260 2.8393 153.0044

THR_143@O LYS_158@HZ3 LYS_158@NZ 26 0.0260 2.8519 157.4175

ASN_112@OD1 GLN_114@HE21 GLN_114@NE2 26 0.0260 2.8755 157.6463

ALA_268@O GLN_255@HE21 GLN_255@NE2 26 0.0260 2.8770 151.5129

GLU_5@OE2 ARG_22@HH22 ARG_22@NH2 26 0.0260 2.8807 147.6821

GLU_257@OE1 ARG_260@HH12 ARG_260@NH1 26 0.0260 2.8810 154.4522

ASN_112@O GLN_114@H GLN_114@N 26 0.0260 2.8824 145.4687

ASP_81@O HIE_36@HE2 HIE_36@NE2 26 0.0260 2.8939 143.3920

LYS_258@O ARG_260@HH11 ARG_260@NH1 26 0.0260 2.8973 148.8021

VAL_145@O GLN_147@H GLN_147@N 26 0.0260 2.9022 142.6469

SER_126@O ARG_441@HH22 ARG_441@NH2 26 0.0260 2.9138 158.3108

GLN_412@OE1 TYR_414@HH TYR_414@OH 25 0.0250 2.7427 156.3324

SER_91@O SER_91@HG SER_91@OG 25 0.0250 2.7788 140.4778

SER_259@O LYS_258@HZ2 LYS_258@NZ 25 0.0250 2.8326 155.9665

GLN_147@O LYS_149@HZ3 LYS_149@NZ 25 0.0250 2.8474 156.0481

GLU_245@OE1 ARG_237@HE ARG_237@NE 25 0.0250 2.8661 157.8408

GLU_5@OE1 ASN_24@HD22 ASN_24@ND2 25 0.0250 2.8679 163.5287

GLU_323@OE2 VAL_324@H VAL_324@N 25 0.0250 2.8724 149.0902

GLU_284@OE1 ARG_216@HH11 ARG_216@NH1 25 0.0250 2.8804 150.9290

GLU_421@OE1 TRP_422@H TRP_422@N 25 0.0250 2.8832 152.2552

ASP_443@OD1 ARG_441@HH22 ARG_441@NH2 25 0.0250 2.8839 153.0477

ASP_443@OD2 ARG_441@HH22 ARG_441@NH2 25 0.0250 2.9047 152.1720

ASP_125@OD2 LYS_131@HZ2 LYS_131@NZ 24 0.0240 2.7876 160.2300

ASP_443@OD1 GLN_401@HE22 GLN_401@NE2 24 0.0240 2.8293 157.9233

ASP_125@OD1 LYS_127@HZ3 LYS_127@NZ 24 0.0240 2.8510 153.6596

ASP_150@OD2 LYS_149@HZ1 LYS_149@NZ 24 0.0240 2.8512 155.3233

GLN_147@O LYS_149@HZ1 LYS_149@NZ 24 0.0240 2.8528 150.8838

ASP_443@OD2 GLN_401@HE22 GLN_401@NE2 24 0.0240 2.8698 159.6001

GLN_432@OE1 ILE_433@H ILE_433@N 24 0.0240 2.8745 151.6826

ASP_384@OD1 GLN_285@HE21 GLN_285@NE2 24 0.0240 2.8800 152.2707

VAL_312@O GLN_285@HE22 GLN_285@NE2 24 0.0240 2.8874 161.5687

GLU_331@OE1 ARG_124@HH22 ARG_124@NH2 24 0.0240 2.9015 150.5739

SER_267@OG GLN_281@HE21 GLN_281@NE2 24 0.0240 2.9256 156.2550

GLN_14@OE1 SER_141@HG SER_141@OG 23 0.0230 2.7799 160.2301

THR_398@OG1 SER_396@HG SER_396@OG 23 0.0230 2.8140 154.0896

ASP_384@O ASN_318@HD21 ASN_318@ND2 23 0.0230 2.8186 164.0616

ASN_190@OD1 SER_146@HG SER_146@OG 23 0.0230 2.8250 154.6381

GLN_255@OE1 LYS_258@HZ3 LYS_258@NZ 23 0.0230 2.8366 152.2682

SER_396@OG THR_398@HG1 THR_398@OG1 23 0.0230 2.8433 157.9270

GLY_57@O GLN_55@HE21 GLN_55@NE2 23 0.0230 2.8610 155.3147

ARG_260@O ARG_260@HH11 ARG_260@NH1 23 0.0230 2.8619 160.7495

GLN_281@OE1 ARG_282@HH12 ARG_282@NH1 23 0.0230 2.8709 144.4180

GLU_257@OE1 ARG_260@HH21 ARG_260@NH2 23 0.0230 2.8725 148.4545

GLU_331@OE2 ARG_124@HH22 ARG_124@NH2 23 0.0230 2.8866 157.5821

GLU_323@OE1 VAL_324@H VAL_324@N 23 0.0230 2.8939 151.6504

PRO_380@O LEU_382@H LEU_382@N 23 0.0230 2.8952 145.9826

GLU_301@OE1 GLY_298@H GLY_298@N 23 0.0230 2.8991 147.6162

ASP_384@O ASN_318@HD22 ASN_318@ND2 23 0.0230 2.9168 158.5337

GLN_379@OE1 SER_151@HG SER_151@OG 22 0.0220 2.7600 156.6639

SER_128@O SER_128@HG SER_128@OG 22 0.0220 2.7610 142.4718

ASP_125@OD2 LYS_131@HZ1 LYS_131@NZ 22 0.0220 2.7840 162.1095

ASP_125@OD1 LYS_131@HZ3 LYS_131@NZ 22 0.0220 2.7944 164.5859

GLU_301@OE2 GLY_299@H GLY_299@N 22 0.0220 2.8164 151.2308

ASP_264@OD1 ASP_264@H ASP_264@N 22 0.0220 2.8213 138.7753

ILE_113@O GLN_114@HE22 GLN_114@NE2 22 0.0220 2.8413 155.5273

GLN_14@OE1 GLN_142@HE22 GLN_142@NE2 22 0.0220 2.8764 155.9104

GLN_401@OE1 ARG_441@HE ARG_441@NE 22 0.0220 2.8930 152.7894

SER_141@OG GLN_14@HE21 GLN_14@NE2 22 0.0220 2.8970 155.3753

ARG_22@O ARG_22@HH11 ARG_22@NH1 22 0.0220 2.9015 154.7154

PHE_47@O ASN_45@HD21 ASN_45@ND2 22 0.0220 2.9238 152.3705

THR_431@O THR_431@HG1 THR_431@OG1 21 0.0210 2.8092 142.0444

ASP_425@OD1 GLN_424@HE22 GLN_424@NE2 21 0.0210 2.8662 163.0286

ASP_125@OD1 LYS_127@HZ1 LYS_127@NZ 21 0.0210 2.8663 154.7345

GLU_331@OE2 ARG_124@HH12 ARG_124@NH1 21 0.0210 2.8747 154.3480

THR_143@O VAL_145@H VAL_145@N 21 0.0210 2.8902 143.5620

GLU_301@O SER_296@HG SER_296@OG 20 0.0200 2.7469 157.2866

GLN_114@OE1 GLN_14@HE21 GLN_14@NE2 20 0.0200 2.8285 157.6251

GLU_421@OE1 GLN_218@HE22 GLN_218@NE2 20 0.0200 2.8403 157.9673

GLY_104@O ARG_106@HH11 ARG_106@NH1 20 0.0200 2.8531 152.8519

SER_417@O LYS_428@HZ2 LYS_428@NZ 20 0.0200 2.8668 156.8911

GLN_78@OE1 ARG_58@HH21 ARG_58@NH2 20 0.0200 2.8731 143.1815

ASN_208@OD1 ARG_223@HH11 ARG_223@NH1 20 0.0200 2.8755 157.7566

GLU_5@OE2 ASN_24@HD21 ASN_24@ND2 20 0.0200 2.8866 163.2024

GLY_241@O GLN_242@HE22 GLN_242@NE2 20 0.0200 2.8909 154.2604

SER_126@OG LYS_127@HZ2 LYS_127@NZ 20 0.0200 2.9024 150.6279

SER_267@OG GLN_281@HE22 GLN_281@NE2 20 0.0200 2.9034 156.4100

LYS_127@O SER_128@HG SER_128@OG 19 0.0190 2.7303 157.7650

GLN_251@OE1 TYR_249@HH TYR_249@OH 19 0.0190 2.7817 152.3986

THR_53@O LYS_54@HZ1 LYS_54@NZ 19 0.0190 2.7945 156.4995

GLN_6@O THR_103@HG1 THR_103@OG1 19 0.0190 2.8087 143.7791

GLU_284@OE1 ARG_282@HH21 ARG_282@NH2 19 0.0190 2.8444 154.3660

LYS_98@O LYS_98@HZ1 LYS_98@NZ 19 0.0190 2.8581 162.4134

GLY_102@O GLY_104@H GLY_104@N 19 0.0190 2.8624 142.4182

ALA_442@O ARG_441@HE ARG_441@NE 19 0.0190 2.8634 149.2163

GLU_257@OE1 ARG_260@HH22 ARG_260@NH2 19 0.0190 2.8723 153.9185

ASN_419@OD1 LYS_212@HZ2 LYS_212@NZ 19 0.0190 2.8782 153.1644

GLY_305@O GLN_42@HE21 GLN_42@NE2 19 0.0190 2.8782 157.6378

GLU_331@OE1 ARG_441@HH12 ARG_441@NH1 19 0.0190 2.8833 152.4288

GLN_14@OE1 ILE_113@H ILE_113@N 19 0.0190 2.8948 156.3617

SER_367@OG ARG_164@H ARG_164@N 19 0.0190 2.8956 154.3232

GLU_355@OE2 LEU_356@H LEU_356@N 19 0.0190 2.9019 148.9608

GLU_245@OE1 ARG_237@HH21 ARG_237@NH2 19 0.0190 2.9148 148.1168

SER_94@OG ASN_30@HD22 ASN_30@ND2 19 0.0190 2.9239 157.4697

SER_126@OG LYS_127@HZ1 LYS_127@NZ 19 0.0190 2.9316 151.1857

ASP_152@OD2 ASP_152@H ASP_152@N 18 0.0180 2.7664 138.4839

SER_201@O SER_201@HG SER_201@OG 18 0.0180 2.7951 139.9615

ASN_419@OD1 LYS_212@HZ1 LYS_212@NZ 18 0.0180 2.8514 152.0053

ASP_384@OD2 GLN_285@HE21 GLN_285@NE2 18 0.0180 2.8549 152.3864

GLN_255@OE1 LYS_258@HZ1 LYS_258@NZ 18 0.0180 2.8584 151.1719

SER_259@O LYS_258@HZ1 LYS_258@NZ 18 0.0180 2.8689 153.2454

GLN_42@OE1 TRP_40@HE1 TRP_40@NE1 18 0.0180 2.8719 156.0903

ALA_254@O LEU_256@H LEU_256@N 18 0.0180 2.8841 141.9261

PHE_246@O ARG_237@HE ARG_237@NE 18 0.0180 2.8938 149.7776

GLN_122@O ARG_124@HH11 ARG_124@NH1 18 0.0180 2.8972 155.3549

ASP_150@OD2 ASP_152@H ASP_152@N 18 0.0180 2.9307 159.7067

TRP_34@O PHE_47@H PHE_47@N 18 0.0180 2.9425 157.8274

GLY_299@O GLN_302@H GLN_302@N 18 0.0180 2.9463 155.7920

LEU_278@O THR_221@HG1 THR_221@OG1 17 0.0170 2.7429 161.2323

SER_75@OG SER_76@HG SER_76@OG 17 0.0170 2.8066 164.5257

ASP_125@OD2 LYS_131@HZ3 LYS_131@NZ 17 0.0170 2.8226 160.2121

VAL_312@O GLN_285@HE21 GLN_285@NE2 17 0.0170 2.8282 151.9311

GLU_5@OE1 ARG_22@HH12 ARG_22@NH1 17 0.0170 2.8368 149.3827

THR_311@O THR_214@HG1 THR_214@OG1 17 0.0170 2.8413 156.9975

SER_417@O LYS_428@HZ1 LYS_428@NZ 17 0.0170 2.8494 154.6993

SER_28@O SER_28@HG SER_28@OG 17 0.0170 2.8611 143.9925

GLN_410@OE1 ARG_408@HH12 ARG_408@NH1 17 0.0170 2.8650 151.8117

ASP_140@OD2 ASP_140@H ASP_140@N 17 0.0170 2.8747 138.6983

GLN_142@OE1 GLN_114@HE21 GLN_114@NE2 17 0.0170 2.8759 162.6962

SER_170@O SER_170@HG SER_170@OG 17 0.0170 2.8857 140.1466

GLU_331@OE2 ALA_332@H ALA_332@N 17 0.0170 2.9016 145.8818

GLU_331@OE1 ALA_332@H ALA_332@N 17 0.0170 2.9018 148.2369

ASP_162@OD1 ARG_164@HE ARG_164@NE 17 0.0170 2.9029 152.2971

ALA_442@O ARG_441@HH11 ARG_441@NH1 17 0.0170 2.9140 149.3985

GLU_257@OE2 ARG_260@HH22 ARG_260@NH2 17 0.0170 2.9299 151.0933

GLU_418@OE2 ASN_419@H ASN_419@N 17 0.0170 2.9382 146.5421

GLU_378@OE2 THR_347@HG1 THR_347@OG1 16 0.0160 2.7329 155.8845

ASP_315@O THR_313@HG1 THR_313@OG1 16 0.0160 2.7487 164.4637

GLU_5@OE2 THR_103@HG1 THR_103@OG1 16 0.0160 2.7565 159.9518

ASP_125@OD1 LYS_131@HZ1 LYS_131@NZ 16 0.0160 2.8124 157.6840

GLN_147@O LYS_149@HZ2 LYS_149@NZ 16 0.0160 2.8427 153.8949

PHE_188@O ASN_190@H ASN_190@N 16 0.0160 2.8473 146.3259

LYS_158@O LYS_158@HZ3 LYS_158@NZ 16 0.0160 2.8512 150.9999

GLN_114@OE1 GLN_142@HE21 GLN_142@NE2 16 0.0160 2.8597 163.8730

GLN_114@OE1 GLN_114@H GLN_114@N 16 0.0160 2.8604 149.8801

LYS_98@O LYS_98@HZ3 LYS_98@NZ 16 0.0160 2.8632 157.7454

SER_146@O SER_146@HG SER_146@OG 16 0.0160 2.8665 142.9380

THR_143@O LYS_158@HZ2 LYS_158@NZ 16 0.0160 2.8776 157.0134

GLN_142@OE1 GLN_142@H GLN_142@N 16 0.0160 2.8793 143.4297

GLU_355@OE1 LEU_356@H LEU_356@N 16 0.0160 2.8887 150.4512

SER_128@O ASP_130@H ASP_130@N 16 0.0160 2.8887 144.7476

ILE_155@O GLN_147@HE22 GLN_147@NE2 16 0.0160 2.8935 153.1503

SER_141@OG GLN_142@HE22 GLN_142@NE2 16 0.0160 2.8950 155.8353

TRP_422@O GLN_424@H GLN_424@N 16 0.0160 2.9013 145.1816

GLU_195@O THR_197@H THR_197@N 16 0.0160 2.9044 147.4193

SER_259@OG LEU_261@H LEU_261@N 16 0.0160 2.9483 155.3107

GLU_5@OE1 SER_7@HG SER_7@OG 15 0.0150 2.7417 160.2384

ASP_264@O SER_267@HG SER_267@OG 15 0.0150 2.7453 158.6567

ALA_294@O SER_295@HG SER_295@OG 15 0.0150 2.7700 154.2501

ASP_125@OD1 LYS_131@HZ2 LYS_131@NZ 15 0.0150 2.8026 159.1851

THR_53@O LYS_54@HZ3 LYS_54@NZ 15 0.0150 2.8112 151.4287

GLN_14@OE1 GLN_114@HE21 GLN_114@NE2 15 0.0150 2.8150 149.9615

GLU_355@OE2 TRP_40@HE1 TRP_40@NE1 15 0.0150 2.8225 151.6823

GLN_255@OE1 LYS_258@HZ2 LYS_258@NZ 15 0.0150 2.8408 152.3693

GLU_15@OE2 GLN_78@HE21 GLN_78@NE2 15 0.0150 2.8512 160.1881

ASP_81@OD2 THR_80@HG1 THR_80@OG1 15 0.0150 2.8520 159.4616

GLU_301@OE1 LEU_297@H LEU_297@N 15 0.0150 2.8699 157.3030

GLY_440@O ARG_441@HE ARG_441@NE 15 0.0150 2.8744 151.9776

GLN_14@OE1 ASN_112@HD22 ASN_112@ND2 15 0.0150 2.8751 153.9555

ASP_384@OD2 GLU_314@H GLU_314@N 15 0.0150 2.8782 155.5656

GLN_3@OE1 VAL_4@H VAL_4@N 15 0.0150 2.8790 152.7043

ASP_117@O HIE_336@HE2 HIE_336@NE2 15 0.0150 2.8812 151.8421

THR_347@O THR_347@HG1 THR_347@OG1 15 0.0150 2.8887 142.9040

ASN_219@OD1 ARG_282@HH11 ARG_282@NH1 15 0.0150 2.8989 154.8329

ARG_282@O ARG_282@HE ARG_282@NE 15 0.0150 2.8994 154.4535

GLN_374@OE1 GLN_147@HE21 GLN_147@NE2 15 0.0150 2.9064 157.6829

GLU_257@OE1 ARG_260@HE ARG_260@NE 15 0.0150 2.9111 150.9183

SER_259@OG LEU_262@H LEU_262@N 15 0.0150 2.9262 150.4242

GLY_286@O ARG_237@HH12 ARG_237@NH1 15 0.0150 2.9343 155.5325

TYR_236@OH TYR_99@H TYR_99@N 15 0.0150 2.9551 159.2336

TRP_235@O THR_248@H THR_248@N 15 0.0150 2.9591 155.6166

GLU_5@OE1 THR_103@HG1 THR_103@OG1 14 0.0140 2.6921 160.1677

GLU_421@OE2 GLN_218@HE22 GLN_218@NE2 14 0.0140 2.8235 154.5752

GLU_421@OE1 GLN_218@HE21 GLN_218@NE2 14 0.0140 2.8447 153.3193

ASP_150@OD1 LYS_149@H LYS_149@N 14 0.0140 2.8564 161.8960

GLN_410@OE1 TRP_359@HE1 TRP_359@NE1 14 0.0140 2.8600 147.6604

THR_93@O GLY_95@H GLY_95@N 14 0.0140 2.8670 148.5434

LEU_262@O LYS_258@HZ1 LYS_258@NZ 14 0.0140 2.8686 149.8465

ASP_443@OD2 GLN_401@HE21 GLN_401@NE2 14 0.0140 2.8696 159.8078

ASN_419@OD1 LYS_212@HZ3 LYS_212@NZ 14 0.0140 2.8711 150.9390

TYR_303@OH HIE_230@HE2 HIE_230@NE2 14 0.0140 2.8974 144.3934

GLU_331@OE2 ARG_441@HH22 ARG_441@NH2 14 0.0140 2.9031 148.7537

TYR_300@O GLN_302@H GLN_302@N 14 0.0140 2.9156 145.4606

GLU_333@O GLN_338@H GLN_338@N 14 0.0140 2.9164 149.0797

GLU_331@OE1 ARG_124@HH12 ARG_124@NH1 14 0.0140 2.9181 150.6552

GLN_14@O ALA_17@H ALA_17@N 14 0.0140 2.9266 152.5821

PRO_306@O GLN_42@HE22 GLN_42@NE2 14 0.0140 2.9288 163.3836

GLU_245@OE2 THR_239@HG1 THR_239@OG1 13 0.0130 2.7410 149.6616

ASN_144@OD1 SER_146@HG SER_146@OG 13 0.0130 2.7655 157.4596

ASP_152@OD1 ASP_152@H ASP_152@N 13 0.0130 2.7742 137.7859

GLY_95@O THR_96@HG1 THR_96@OG1 13 0.0130 2.8012 156.9070

ASP_167@O LYS_169@HZ2 LYS_169@NZ 13 0.0130 2.8226 157.2415

SER_263@O ARG_265@H ARG_265@N 13 0.0130 2.8245 142.6556

ILE_2@O GLN_3@HE21 GLN_3@NE2 13 0.0130 2.8286 151.0810

LEU_382@O LYS_377@HZ2 LYS_377@NZ 13 0.0130 2.8448 150.1077

ALA_92@O SER_94@HG SER_94@OG 13 0.0130 2.8477 155.5332

ASP_130@OD2 LYS_131@H LYS_131@N 13 0.0130 2.8504 144.4336

ASP_425@OD2 GLN_424@HE22 GLN_424@NE2 13 0.0130 2.8540 160.9937

THR_156@O THR_156@HG1 THR_156@OG1 13 0.0130 2.8591 144.4105

GLU_314@OE2 GLN_285@HE21 GLN_285@NE2 13 0.0130 2.8684 159.5639

GLN_432@O GLN_412@HE22 GLN_412@NE2 13 0.0130 2.8750 147.8784

ILE_433@O GLN_432@HE22 GLN_432@NE2 13 0.0130 2.8754 159.6537

SER_417@O LYS_428@HZ3 LYS_428@NZ 13 0.0130 2.8801 155.7554

ASP_425@OD2 ARG_426@HE ARG_426@NE 13 0.0130 2.8822 155.0059

ALA_65@O ARG_68@HH12 ARG_68@NH1 13 0.0130 2.8852 142.5484

ASP_425@OD2 ARG_426@HH21 ARG_426@NH2 13 0.0130 2.8900 151.2324

ASN_144@OD1 ILE_192@H ILE_192@N 13 0.0130 2.9100 159.9316

ASP_150@OD1 ASP_152@H ASP_152@N 13 0.0130 2.9248 161.8494

GLN_147@O LYS_149@H LYS_149@N 13 0.0130 2.9371 144.5921

SER_396@OG PHE_399@H PHE_399@N 13 0.0130 2.9415 151.5342

ASP_225@OD2 SER_206@HG SER_206@OG 12 0.0120 2.7146 161.9966

LEU_59@O SER_60@HG SER_60@OG 12 0.0120 2.8182 152.0964

SER_148@O SER_148@HG SER_148@OG 12 0.0120 2.8228 143.1190

ASP_130@O SER_132@HG SER_132@OG 12 0.0120 2.8290 162.3295

GLN_142@O ASN_144@HD22 ASN_144@ND2 12 0.0120 2.8568 148.8874

THR_239@O THR_239@HG1 THR_239@OG1 12 0.0120 2.8584 142.3479

GLN_285@OE1 GLN_285@H GLN_285@N 12 0.0120 2.8592 145.0515

ASP_402@OD2 ASN_405@H ASN_405@N 12 0.0120 2.8617 153.1131

THR_277@O THR_277@HG1 THR_277@OG1 12 0.0120 2.8640 142.0832

GLN_251@OE1 ASN_231@HD21 ASN_231@ND2 12 0.0120 2.8678 162.5576

GLY_241@O ARG_106@HE ARG_106@NE 12 0.0120 2.8782 148.0853

GLN_78@OE1 THR_79@H THR_79@N 12 0.0120 2.8796 151.6623

LEU_43@O GLN_42@HE22 GLN_42@NE2 12 0.0120 2.8900 162.3243

ASP_90@OD1 THR_93@H THR_93@N 12 0.0120 2.8942 164.8397

GLU_418@OE1 ASN_419@H ASN_419@N 12 0.0120 2.9015 147.4188

ASP_150@O ASP_152@H ASP_152@N 12 0.0120 2.9040 146.3451

SER_126@OG LYS_127@HZ3 LYS_127@NZ 12 0.0120 2.9046 153.0473

TYR_249@OH ARG_232@HE ARG_232@NE 12 0.0120 2.9108 149.8847

GLU_257@OE2 ARG_260@HE ARG_260@NE 12 0.0120 2.9142 151.2457

GLY_440@O ARG_441@HH21 ARG_441@NH2 12 0.0120 2.9164 144.6128

ARG_164@O ARG_164@HE ARG_164@NE 12 0.0120 2.9182 151.4446

ASN_18@O SER_19@HG SER_19@OG 11 0.0110 2.8024 152.3439

GLY_1@O SER_26@HG SER_26@OG 11 0.0110 2.8150 149.8372

LYS_272@O LYS_272@HZ1 LYS_272@NZ 11 0.0110 2.8177 154.0437

LYS_98@O LYS_98@HZ2 LYS_98@NZ 11 0.0110 2.8190 158.8546

ASP_81@OD2 SER_77@HG SER_77@OG 11 0.0110 2.8210 163.0462

ASP_443@OD2 ASP_443@H ASP_443@N 11 0.0110 2.8247 139.2386

GLN_401@OE1 ARG_441@HH21 ARG_441@NH2 11 0.0110 2.8395 143.6560

GLU_331@OE1 ARG_441@HH22 ARG_441@NH2 11 0.0110 2.8492 150.3166

SER_141@O LYS_158@HZ3 LYS_158@NZ 11 0.0110 2.8503 150.0322

LEU_261@O ARG_237@HH22 ARG_237@NH2 11 0.0110 2.8511 155.3170

GLY_298@O ARG_232@HE ARG_232@NE 11 0.0110 2.8525 153.4559

GLU_15@OE1 LYS_169@HZ2 LYS_169@NZ 11 0.0110 2.8552 153.0156

GLU_421@OE2 GLN_218@HE21 GLN_218@NE2 11 0.0110 2.8571 155.8322

GLU_5@OE1 ASN_24@HD21 ASN_24@ND2 11 0.0110 2.8862 165.4265

ASP_140@OD1 ASP_140@H ASP_140@N 11 0.0110 2.8879 138.2685

LEU_382@O LYS_377@HZ3 LYS_377@NZ 11 0.0110 2.8900 151.2602

SER_128@OG LYS_131@HZ1 LYS_131@NZ 11 0.0110 2.8913 152.4950

SER_151@OG GLN_379@HE21 GLN_379@NE2 11 0.0110 2.8934 151.7669

SER_75@O SER_75@HG SER_75@OG 11 0.0110 2.8939 140.4029

PHE_246@O ARG_237@HH21 ARG_237@NH2 11 0.0110 2.8999 146.8340

SER_180@O PHE_182@H PHE_182@N 11 0.0110 2.9005 143.9352

GLU_15@O ALA_17@H ALA_17@N 11 0.0110 2.9148 142.5807

ASP_150@OD2 ASP_150@H ASP_150@N 10 0.0100 2.7609 138.1226

SER_75@O SER_76@HG SER_76@OG 10 0.0100 2.8059 157.6069

THR_93@O THR_96@HG1 THR_96@OG1 10 0.0100 2.8088 153.1248

GLU_15@OE1 LYS_169@HZ3 LYS_169@NZ 10 0.0100 2.8260 154.6990

ASP_157@OD1 GLN_147@HE22 GLN_147@NE2 10 0.0100 2.8441 149.6323

GLN_14@OE1 GLN_142@HE21 GLN_142@NE2 10 0.0100 2.8466 154.0245

SER_94@O SER_94@HG SER_94@OG 10 0.0100 2.8614 141.9381

ASP_315@OD2 LEU_316@H LEU_316@N 10 0.0100 2.8656 139.9226

ASN_219@OD1 ARG_282@HH21 ARG_282@NH2 10 0.0100 2.8721 148.6088

TRP_439@O ARG_441@HH21 ARG_441@NH2 10 0.0100 2.8806 160.0464

ASP_181@O ASN_186@HD22 ASN_186@ND2 10 0.0100 2.8847 160.1686

GLU_245@OE1 ARG_237@HH11 ARG_237@NH1 10 0.0100 2.8859 157.7292

SER_385@OG LYS_377@HZ3 LYS_377@NZ 10 0.0100 2.8897 147.6853

SER_148@O GLN_147@HE22 GLN_147@NE2 10 0.0100 2.8967 155.8341

SER_128@OG LYS_131@HZ2 LYS_131@NZ 10 0.0100 2.9039 146.0442

ASN_361@O LYS_363@HZ1 LYS_363@NZ 10 0.0100 2.9041 150.9415

VAL_89@O SER_91@H SER_91@N 10 0.0100 2.9056 142.9338

VAL_4@O GLN_6@H GLN_6@N 10 0.0100 2.9066 143.6348

GLU_355@OE1 TRP_40@HE1 TRP_40@NE1 10 0.0100 2.9097 149.0305

ILE_192@O ASN_144@H ASN_144@N 10 0.0100 2.9302 159.0176

GLN_379@OE1 LEU_382@H LEU_382@N 10 0.0100 2.9314 154.6452

SER_417@OG ASP_420@H ASP_420@N 10 0.0100 2.9527 142.2189

GLU_314@N GLN_285@HE21 GLN_285@NE2 10 0.0100 2.9532 145.0777

GLU_269@OE1 SER_267@HG SER_267@OG 9 0.0090 2.6550 162.5973

GLU_301@OE2 SER_296@HG SER_296@OG 9 0.0090 2.6724 157.6707

ASP_130@OD2 SER_129@HG SER_129@OG 9 0.0090 2.7508 153.5600

ASP_130@OD1 SER_129@HG SER_129@OG 9 0.0090 2.7591 161.7151

LEU_382@O LYS_377@HZ1 LYS_377@NZ 9 0.0090 2.7862 147.3808

GLU_257@OE1 LYS_98@HZ1 LYS_98@NZ 9 0.0090 2.7901 158.5312

ASN_18@OD1 SER_75@HG SER_75@OG 9 0.0090 2.7986 158.8202

GLU_364@O LYS_363@HZ2 LYS_363@NZ 9 0.0090 2.8070 154.3430

LYS_272@O SER_274@HG SER_274@OG 9 0.0090 2.8149 163.7157

LEU_262@O LYS_258@HZ2 LYS_258@NZ 9 0.0090 2.8162 154.8528

ASP_443@OXT GLN_401@HE22 GLN_401@NE2 9 0.0090 2.8226 162.4013

ASN_56@O ASN_56@HD22 ASN_56@ND2 9 0.0090 2.8452 143.7314

ASP_157@OD1 ASN_144@HD21 ASN_144@ND2 9 0.0090 2.8453 159.7975

ASP_264@OD2 ARG_265@H ARG_265@N 9 0.0090 2.8456 149.5571

ASP_352@O ARG_309@HH22 ARG_309@NH2 9 0.0090 2.8460 148.5371

GLU_378@OE1 GLN_379@HE22 GLN_379@NE2 9 0.0090 2.8500 157.8684

GLU_15@OE1 LYS_169@HZ1 LYS_169@NZ 9 0.0090 2.8564 159.4476

GLU_279@OE2 GLN_281@HE21 GLN_281@NE2 9 0.0090 2.8647 155.9627

ARG_441@O ARG_441@HE ARG_441@NE 9 0.0090 2.8708 150.2595

ASN_383@OD1 ASP_384@H ASP_384@N 9 0.0090 2.8913 139.7992

LEU_262@O LYS_258@HZ3 LYS_258@NZ 9 0.0090 2.8979 152.3691

GLN_410@OE1 ARG_408@HH22 ARG_408@NH2 9 0.0090 2.9042 152.8879

CYX_184@O ALA_187@H ALA_187@N 9 0.0090 2.9064 164.6443

SER_128@O ARG_124@HE ARG_124@NE 9 0.0090 2.9086 145.5933

TYR_387@O PHE_349@H PHE_349@N 9 0.0090 2.9286 155.6133

MET_163@O MET_166@H MET_166@N 9 0.0090 2.9326 156.4509

ASN_190@ND2 SER_146@HG SER_146@OG 9 0.0090 2.9555 155.3186

GLU_437@OE1 SER_435@HG SER_435@OG 8 0.0080 2.6644 160.1290

ASP_162@OD2 ASP_162@H ASP_162@N 8 0.0080 2.7257 137.9538

GLU_257@OE2 LYS_98@HZ2 LYS_98@NZ 8 0.0080 2.7484 159.6940

GLU_245@OE1 THR_239@HG1 THR_239@OG1 8 0.0080 2.7687 163.5497

ASP_150@OD1 ASP_150@H ASP_150@N 8 0.0080 2.7822 137.0826

GLN_55@OE1 THR_53@HG1 THR_53@OG1 8 0.0080 2.7924 162.7648

PRO_271@O LYS_272@HZ1 LYS_272@NZ 8 0.0080 2.8004 157.6556

ASP_81@OD1 SER_77@HG SER_77@OG 8 0.0080 2.8017 157.0730

ASP_315@OD1 GLN_424@HE21 GLN_424@NE2 8 0.0080 2.8079 160.7086

GLU_15@OE2 LYS_169@HZ2 LYS_169@NZ 8 0.0080 2.8212 151.0047

ASN_361@O LYS_363@HZ3 LYS_363@NZ 8 0.0080 2.8403 154.3557

GLN_55@OE1 GLY_57@H GLY_57@N 8 0.0080 2.8424 158.6775

SER_141@O LYS_158@HZ2 LYS_158@NZ 8 0.0080 2.8486 142.7485

GLN_142@OE1 GLN_14@HE21 GLN_14@NE2 8 0.0080 2.8500 154.0006

GLN_147@O ASN_190@HD22 ASN_190@ND2 8 0.0080 2.8501 146.3390

ASP_384@OD1 GLU_314@H GLU_314@N 8 0.0080 2.8511 158.2935

GLU_364@O LYS_363@HZ1 LYS_363@NZ 8 0.0080 2.8552 150.9260

ASP_443@OD2 ARG_441@HH11 ARG_441@NH1 8 0.0080 2.8585 151.6477

ASP_425@OD1 ARG_426@HH21 ARG_426@NH2 8 0.0080 2.8623 156.6912

THR_311@O THR_311@HG1 THR_311@OG1 8 0.0080 2.8699 146.6605

ARG_106@O ARG_106@HE ARG_106@NE 8 0.0080 2.8705 158.2217

GLU_364@O LYS_363@HZ3 LYS_363@NZ 8 0.0080 2.8715 155.5877

PRO_403@O ARG_404@HE ARG_404@NE 8 0.0080 2.8776 145.8624

THR_239@O GLN_238@HE22 GLN_238@NE2 8 0.0080 2.8792 143.1527

GLU_15@O ASN_112@HD22 ASN_112@ND2 8 0.0080 2.8941 157.7467

ASP_352@OD2 ARG_309@HH21 ARG_309@NH2 8 0.0080 2.9044 162.7098

SER_385@OG LYS_377@HZ1 LYS_377@NZ 8 0.0080 2.9046 148.8142

CYX_370@O ARG_392@HH21 ARG_392@NH2 8 0.0080 2.9062 151.2232

SER_26@O ARG_68@HH11 ARG_68@NH1 8 0.0080 2.9191 148.1555

ALA_187@O ASN_189@H ASN_189@N 8 0.0080 2.9418 146.8918

ASP_225@OD1 SER_206@HG SER_206@OG 7 0.0070 2.6707 161.6175

GLU_421@OE2 LYS_215@HZ2 LYS_215@NZ 7 0.0070 2.7332 153.2575

GLU_15@OE2 LYS_169@HZ3 LYS_169@NZ 7 0.0070 2.7831 156.0289

ASP_162@OD1 ASP_162@H ASP_162@N 7 0.0070 2.7854 138.2839

ASP_315@OD2 GLN_424@HE21 GLN_424@NE2 7 0.0070 2.7971 156.3601

TRP_422@O THR_423@HG1 THR_423@OG1 7 0.0070 2.7978 147.9036

THR_248@O THR_248@HG1 THR_248@OG1 7 0.0070 2.8258 141.4484

GLU_279@OE1 GLN_281@HE21 GLN_281@NE2 7 0.0070 2.8293 157.3544

ASP_402@OD1 ASN_405@H ASN_405@N 7 0.0070 2.8370 155.8388

ASN_231@OD1 ASN_252@HD21 ASN_252@ND2 7 0.0070 2.8422 151.2406

LYS_158@O LYS_158@HZ2 LYS_158@NZ 7 0.0070 2.8451 159.2879

SER_165@OG MET_166@H MET_166@N 7 0.0070 2.8497 141.1560

ASP_157@OD1 GLN_374@HE22 GLN_374@NE2 7 0.0070 2.8523 164.9400

ASN_115@O ASP_117@H ASP_117@N 7 0.0070 2.8534 144.2073

GLN_251@OE1 ASN_252@HD22 ASN_252@ND2 7 0.0070 2.8555 149.9242

GLU_331@OE1 ARG_441@HH21 ARG_441@NH2 7 0.0070 2.8635 151.4461

GLU_245@OE2 ARG_237@HH11 ARG_237@NH1 7 0.0070 2.8668 154.6947

ASP_157@OD2 GLN_374@HE22 GLN_374@NE2 7 0.0070 2.8668 164.6301

THR_221@O ARG_223@HH12 ARG_223@NH1 7 0.0070 2.8669 143.5781

GLY_95@O ARG_232@HH21 ARG_232@NH2 7 0.0070 2.8698 147.0102

ARG_282@O ARG_265@HH11 ARG_265@NH1 7 0.0070 2.8741 140.7625

THR_103@O GLN_242@HE21 GLN_242@NE2 7 0.0070 2.8744 161.8316

ASP_443@O GLN_401@HE22 GLN_401@NE2 7 0.0070 2.8785 155.7427

SER_129@O LYS_131@H LYS_131@N 7 0.0070 2.8826 140.6243

LEU_110@O GLN_14@HE22 GLN_14@NE2 7 0.0070 2.8875 152.5546

GLN_42@O GLN_37@HE22 GLN_37@NE2 7 0.0070 2.8903 150.4446

PRO_9@O ARG_106@HE ARG_106@NE 7 0.0070 2.8908 150.9186

GLN_242@OE1 GLY_243@H GLY_243@N 7 0.0070 2.8972 151.7978

ASN_208@OD1 ARG_210@HH22 ARG_210@NH2 7 0.0070 2.9091 150.5108

ALA_185@O ASN_189@HD21 ASN_189@ND2 7 0.0070 2.9106 148.0997

GLY_273@O ARG_270@HH21 ARG_270@NH2 7 0.0070 2.9169 142.7757

LEU_13@O GLN_14@HE22 GLN_14@NE2 7 0.0070 2.9205 148.1273

THR_341@OG1 ARG_394@HE ARG_394@NE 7 0.0070 2.9299 147.2882

VAL_29@O ASN_30@HD22 ASN_30@ND2 7 0.0070 2.9313 156.0845

GLY_95@O ARG_232@HE ARG_232@NE 7 0.0070 2.9326 152.1185

SER_7@OG ARG_22@HE ARG_22@NE 7 0.0070 2.9428 147.9412

GLU_301@OE1 TYR_303@HH TYR_303@OH 6 0.0060 2.5788 162.4854

GLU_257@OE2 LYS_98@HZ1 LYS_98@NZ 6 0.0060 2.7351 156.2047

ARG_232@O SER_295@HG SER_295@OG 6 0.0060 2.7620 164.8559

GLU_378@OE1 GLN_379@H GLN_379@N 6 0.0060 2.7978 144.6901

ASP_167@O LYS_169@HZ1 LYS_169@NZ 6 0.0060 2.8089 159.2374

GLU_195@OE1 ASP_196@H ASP_196@N 6 0.0060 2.8157 150.4625

ASP_315@OD1 LYS_215@HZ3 LYS_215@NZ 6 0.0060 2.8175 161.8223

GLU_269@OE2 SER_267@HG SER_267@OG 6 0.0060 2.8226 163.9396

PRO_271@O LYS_272@HZ3 LYS_272@NZ 6 0.0060 2.8233 153.9277

LYS_272@O LYS_272@HZ2 LYS_272@NZ 6 0.0060 2.8240 147.9596

LYS_272@O LYS_272@HZ3 LYS_272@NZ 6 0.0060 2.8289 156.9462

LYS_158@O LYS_158@HZ1 LYS_158@NZ 6 0.0060 2.8299 146.1891

ASP_443@OD1 ASP_443@H ASP_443@N 6 0.0060 2.8347 142.2982

ASP_117@OD2 ASN_115@HD21 ASN_115@ND2 6 0.0060 2.8359 156.9281

ASP_287@O ALA_289@H ALA_289@N 6 0.0060 2.8363 143.1296

GLN_251@OE1 ASN_231@HD22 ASN_231@ND2 6 0.0060 2.8410 156.0309

GLU_355@OE1 GLN_412@HE22 GLN_412@NE2 6 0.0060 2.8440 155.9670

ASP_402@O ARG_404@H ARG_404@N 6 0.0060 2.8491 140.1302

LEU_240@O GLN_242@HE21 GLN_242@NE2 6 0.0060 2.8530 142.5767

THR_214@OG1 LYS_215@H LYS_215@N 6 0.0060 2.8574 137.8590

ASN_115@O ASN_115@HD22 ASN_115@ND2 6 0.0060 2.8575 144.6294

GLU_284@OE1 GLN_285@H GLN_285@N 6 0.0060 2.8598 151.9294

ASP_167@O LYS_169@HZ3 LYS_169@NZ 6 0.0060 2.8603 145.3036

SER_128@OG LYS_131@HZ3 LYS_131@NZ 6 0.0060 2.8603 150.2544

GLU_314@OE1 GLN_285@HE21 GLN_285@NE2 6 0.0060 2.8605 162.2759

LYS_317@O ARG_426@HH21 ARG_426@NH2 6 0.0060 2.8615 137.8785

ASP_130@OD1 LYS_131@H LYS_131@N 6 0.0060 2.8619 142.0866

HIE_336@O GLN_338@HE21 GLN_338@NE2 6 0.0060 2.8628 149.1362

PRO_380@O LYS_377@HZ3 LYS_377@NZ 6 0.0060 2.8640 151.9399

GLN_114@OE1 ASN_115@H ASN_115@N 6 0.0060 2.8648 161.7420

GLN_114@OE1 ASN_112@HD21 ASN_112@ND2 6 0.0060 2.8657 155.8833

THR_203@O VAL_205@H VAL_205@N 6 0.0060 2.8662 143.9940

ASN_112@OD1 ILE_113@H ILE_113@N 6 0.0060 2.8686 145.4651

GLN_374@O GLN_374@HE22 GLN_374@NE2 6 0.0060 2.8701 143.5162

GLN_281@OE1 ARG_282@HH22 ARG_282@NH2 6 0.0060 2.8746 143.4522

ASP_130@O LYS_131@HZ1 LYS_131@NZ 6 0.0060 2.8768 149.0485

ASP_157@OD2 GLN_147@HE22 GLN_147@NE2 6 0.0060 2.8848 162.5060

GLY_241@O ARG_106@HH22 ARG_106@NH2 6 0.0060 2.8850 155.0051

GLN_14@OE1 GLN_14@H GLN_14@N 6 0.0060 2.8867 146.4143

HIE_366@O ARG_164@HH12 ARG_164@NH1 6 0.0060 2.8965 149.5929

GLN_281@OE1 ASN_219@HD21 ASN_219@ND2 6 0.0060 2.8969 142.4057

SER_367@OG ARG_164@HE ARG_164@NE 6 0.0060 2.8988 148.1953

ALA_268@O GLN_255@HE22 GLN_255@NE2 6 0.0060 2.9011 153.2899

ILE_113@O GLN_14@HE22 GLN_14@NE2 6 0.0060 2.9058 155.7213

ASN_30@O ASN_30@HD22 ASN_30@ND2 6 0.0060 2.9059 147.8453

SER_148@O GLN_147@HE21 GLN_147@NE2 6 0.0060 2.9060 147.9572

ALA_442@O GLN_401@HE22 GLN_401@NE2 6 0.0060 2.9107 156.8625

THR_214@OG1 LYS_212@HZ2 LYS_212@NZ 6 0.0060 2.9115 157.0300

SER_141@O GLN_14@HE21 GLN_14@NE2 6 0.0060 2.9181 153.5765

SER_206@OG ASP_225@H ASP_225@N 6 0.0060 2.9308 154.5955

ARG_232@O SER_296@HG SER_296@OG 6 0.0060 2.9355 150.2855

SER_128@OG ASP_130@H ASP_130@N 6 0.0060 2.9377 147.7440

SER_28@OG ARG_68@HH21 ARG_68@NH2 6 0.0060 2.9387 148.7066

TRP_40@O ARG_309@HH21 ARG_309@NH2 6 0.0060 2.9483 148.9200

TYR_97@OH GLY_95@H GLY_95@N 6 0.0060 2.9708 149.3893

GLU_437@OE2 SER_435@HG SER_435@OG 5 0.0050 2.6458 167.2308

ASP_264@OD1 SER_263@HG SER_263@OG 5 0.0050 2.6718 171.7777

ASN_178@OD1 TYR_154@HH TYR_154@OH 5 0.0050 2.6814 166.2903

GLU_323@OE1 THR_347@HG1 THR_347@OG1 5 0.0050 2.6916 161.2461

SER_129@O SER_129@HG SER_129@OG 5 0.0050 2.7349 141.8265

ASP_315@OD1 LEU_316@H LEU_316@N 5 0.0050 2.7430 141.6441

GLU_257@OE1 LYS_98@HZ3 LYS_98@NZ 5 0.0050 2.7531 157.6676

ASN_318@O ASN_318@HD22 ASN_318@ND2 5 0.0050 2.7594 146.1704

VAL_174@O THR_156@HG1 THR_156@OG1 5 0.0050 2.7597 153.1980

ASP_162@OD1 SER_367@HG SER_367@OG 5 0.0050 2.7780 157.5236

ASP_315@OD1 LYS_215@HZ2 LYS_215@NZ 5 0.0050 2.7803 153.9811

ASP_315@OD2 LYS_215@HZ2 LYS_215@NZ 5 0.0050 2.7829 157.8474

ASN_190@OD1 ASN_190@H ASN_190@N 5 0.0050 2.7833 137.5364

GLU_323@O THR_347@HG1 THR_347@OG1 5 0.0050 2.8001 148.7577

GLU_257@OE1 LYS_98@HZ2 LYS_98@NZ 5 0.0050 2.8033 150.9014

GLU_418@OE1 LYS_428@HZ1 LYS_428@NZ 5 0.0050 2.8045 154.6773

ASN_361@O LYS_363@HZ2 LYS_363@NZ 5 0.0050 2.8092 154.8807

ASP_425@O TRP_422@HE1 TRP_422@NE1 5 0.0050 2.8269 141.1165

HIE_366@O ARG_164@HH22 ARG_164@NH2 5 0.0050 2.8369 147.5399

GLY_52@O THR_53@HG1 THR_53@OG1 5 0.0050 2.8412 152.5196

SER_141@O LYS_158@HZ1 LYS_158@NZ 5 0.0050 2.8419 162.8070

SER_19@O SER_19@HG SER_19@OG 5 0.0050 2.8430 139.8763

ASP_384@OD2 ASN_383@HD21 ASN_383@ND2 5 0.0050 2.8430 142.1410

ASN_208@OD1 ARG_223@HE ARG_223@NE 5 0.0050 2.8481 150.5614

VAL_145@O GLN_147@HE22 GLN_147@NE2 5 0.0050 2.8543 147.1949

GLU_378@OE2 GLY_348@H GLY_348@N 5 0.0050 2.8568 144.0092

SER_146@OG LYS_149@HZ3 LYS_149@NZ 5 0.0050 2.8577 153.2980

ASP_372@O GLN_374@H GLN_374@N 5 0.0050 2.8577 149.2940

GLU_5@OE1 GLN_6@H GLN_6@N 5 0.0050 2.8578 145.8566

ASN_30@OD1 ASN_31@HD21 ASN_31@ND2 5 0.0050 2.8601 141.7617

GLU_284@OE2 ARG_282@HH11 ARG_282@NH1 5 0.0050 2.8605 151.7821

GLU_15@OE2 LYS_169@HZ1 LYS_169@NZ 5 0.0050 2.8639 156.5005

GLU_364@OE1 HIE_366@HE2 HIE_366@NE2 5 0.0050 2.8642 155.3404

ASN_219@O GLN_218@HE22 GLN_218@NE2 5 0.0050 2.8657 165.3418

GLU_253@OE2 ALA_254@H ALA_254@N 5 0.0050 2.8672 143.5970

HIE_366@ND1 HIE_366@H HIE_366@N 5 0.0050 2.8674 139.0084

GLY_440@O ALA_442@H ALA_442@N 5 0.0050 2.8685 150.2951

GLU_5@OE1 GLU_5@H GLU_5@N 5 0.0050 2.8693 143.9982

ASN_252@OD1 ASN_231@HD22 ASN_231@ND2 5 0.0050 2.8712 170.2171

ASN_24@O ASN_24@HD22 ASN_24@ND2 5 0.0050 2.8722 147.3829

PRO_271@O LYS_272@HZ2 LYS_272@NZ 5 0.0050 2.8730 153.6173

LEU_59@O GLN_55@HE22 GLN_55@NE2 5 0.0050 2.8743 147.9039

ASP_352@O ARG_309@HH12 ARG_309@NH1 5 0.0050 2.8745 148.1609

SER_26@O ARG_68@HH21 ARG_68@NH2 5 0.0050 2.8764 155.3438

GLN_147@O SER_146@HG SER_146@OG 5 0.0050 2.8770 162.8576

ASP_384@OD1 ASN_383@HD22 ASN_383@ND2 5 0.0050 2.8771 150.1343

LYS_215@O THR_214@HG1 THR_214@OG1 5 0.0050 2.8824 159.9306

ASP_27@O VAL_29@H VAL_29@N 5 0.0050 2.8829 145.8810

SER_19@OG THR_20@H THR_20@N 5 0.0050 2.8861 136.3473

GLU_253@OE1 ALA_254@H ALA_254@N 5 0.0050 2.8948 141.4737

TRP_40@O GLN_42@HE21 GLN_42@NE2 5 0.0050 2.8952 146.2241

ASP_10@OD1 LEU_11@H LEU_11@N 5 0.0050 2.8962 138.6703

ASP_425@OD1 ARG_426@HE ARG_426@NE 5 0.0050 2.8973 158.5960

ASN_112@O GLN_114@HE21 GLN_114@NE2 5 0.0050 2.8998 157.4268

SER_367@OG ARG_164@HH21 ARG_164@NH2 5 0.0050 2.9002 144.3789

LEU_11@O ARG_106@HH21 ARG_106@NH2 5 0.0050 2.9019 143.9049

SER_129@OG ARG_124@HH12 ARG_124@NH1 5 0.0050 2.9043 141.9493

LEU_382@O SER_385@H SER_385@N 5 0.0050 2.9101 150.6290

ASP_162@OD1 ARG_164@HH11 ARG_164@NH1 5 0.0050 2.9195 163.0062

GLN_251@OE1 ARG_232@HH11 ARG_232@NH1 5 0.0050 2.9226 155.7938

LYS_258@O ARG_260@HE ARG_260@NE 5 0.0050 2.9226 150.5963

GLU_331@OE1 ARG_441@HH11 ARG_441@NH1 5 0.0050 2.9228 146.2217

HIE_336@O GLN_338@HE22 GLN_338@NE2 5 0.0050 2.9233 151.5256

LYS_258@O ARG_260@HH12 ARG_260@NH1 5 0.0050 2.9262 150.4348

SER_141@OG GLN_142@HE21 GLN_142@NE2 5 0.0050 2.9299 147.8343

SER_385@OG LYS_377@HZ2 LYS_377@NZ 5 0.0050 2.9325 151.0724

GLN_147@OE1 GLN_374@HE22 GLN_374@NE2 5 0.0050 2.9346 167.5271

ASP_443@O GLN_401@HE21 GLN_401@NE2 5 0.0050 2.9350 160.8560

GLN_401@OE1 ARG_441@HH11 ARG_441@NH1 5 0.0050 2.9360 152.6404

SER_151@OG GLN_379@HE22 GLN_379@NE2 5 0.0050 2.9452 154.3734

GLN_6@NE2 THR_105@H THR_105@N 5 0.0050 2.9577 158.7188

ASN_361@O LYS_363@H LYS_363@N 5 0.0050 2.9615 140.0287

PHE_399@O ASP_402@H ASP_402@N 5 0.0050 2.9702 152.5937

SER_396@OG THR_398@H THR_398@N 5 0.0050 2.9793 155.6245

GLU_323@OE2 THR_347@HG1 THR_347@OG1 4 0.0040 2.6581 156.6782

GLU_15@OE2 THR_79@HG1 THR_79@OG1 4 0.0040 2.6610 155.6514

SER_76@O SER_76@HG SER_76@OG 4 0.0040 2.6630 140.5721

GLU_418@OE2 LYS_428@HZ3 LYS_428@NZ 4 0.0040 2.7476 156.7743

GLU_279@OE2 THR_221@HG1 THR_221@OG1 4 0.0040 2.7506 163.1981

SER_151@OG TYR_154@HH TYR_154@OH 4 0.0040 2.7645 156.3078

GLN_78@OE1 THR_79@HG1 THR_79@OG1 4 0.0040 2.7670 161.1321

ASN_30@OD1 SER_94@HG SER_94@OG 4 0.0040 2.7706 157.2631

ASP_27@OD2 SER_28@HG SER_28@OG 4 0.0040 2.7810 160.6136

ARG_386@O SER_385@HG SER_385@OG 4 0.0040 2.7861 159.3523

ASP_130@O LYS_131@HZ3 LYS_131@NZ 4 0.0040 2.8006 154.6014

HIE_211@O LYS_212@HZ2 LYS_212@NZ 4 0.0040 2.8007 150.2854

ASP_352@OD1 GLN_374@HE21 GLN_374@NE2 4 0.0040 2.8012 157.8472

GLU_378@OE2 TYR_154@HH TYR_154@OH 4 0.0040 2.8056 153.2973

GLU_331@OE2 ARG_441@HH11 ARG_441@NH1 4 0.0040 2.8069 144.4957

LYS_149@O LYS_149@HZ3 LYS_149@NZ 4 0.0040 2.8097 159.7165

GLU_279@OE1 GLU_279@H GLU_279@N 4 0.0040 2.8219 150.3683

GLU_437@OE2 ARG_408@HH11 ARG_408@NH1 4 0.0040 2.8235 145.6085

TYR_73@O SER_60@HG SER_60@OG 4 0.0040 2.8343 155.7717

GLN_255@OE1 GLN_255@H GLN_255@N 4 0.0040 2.8453 157.5246

LYS_258@O LYS_258@HZ1 LYS_258@NZ 4 0.0040 2.8521 156.1796

GLU_195@OE2 ASP_196@H ASP_196@N 4 0.0040 2.8570 150.8532

TYR_303@OH GLY_204@H GLY_204@N 4 0.0040 2.8619 149.9381

GLY_1@O GLN_3@HE22 GLN_3@NE2 4 0.0040 2.8621 145.9923

THR_239@OG1 LEU_240@H LEU_240@N 4 0.0040 2.8631 140.3004

ASP_443@OXT GLN_401@HE21 GLN_401@NE2 4 0.0040 2.8639 157.6061

GLU_418@OE1 LYS_428@HZ2 LYS_428@NZ 4 0.0040 2.8649 162.4879

GLU_284@OE2 ARG_282@HH21 ARG_282@NH2 4 0.0040 2.8659 155.9698

ASN_189@O ASN_189@HD22 ASN_189@ND2 4 0.0040 2.8680 146.1314

GLU_284@OE2 GLN_285@H GLN_285@N 4 0.0040 2.8688 156.2967

THR_203@O THR_203@HG1 THR_203@OG1 4 0.0040 2.8695 140.8340

PRO_373@O GLN_374@HE22 GLN_374@NE2 4 0.0040 2.8734 155.0444

GLN_255@OE1 GLU_257@H GLU_257@N 4 0.0040 2.8766 141.7351

GLU_355@OE2 GLN_412@HE22 GLN_412@NE2 4 0.0040 2.8771 164.3231

LYS_258@O LYS_258@HZ3 LYS_258@NZ 4 0.0040 2.8794 156.5445

ASP_402@OD1 ARG_404@HH11 ARG_404@NH1 4 0.0040 2.8809 154.8872

GLU_314@OE2 LYS_215@HZ2 LYS_215@NZ 4 0.0040 2.8832 151.1477

ASN_190@OD1 SER_191@H SER_191@N 4 0.0040 2.8860 142.5118

THR_248@OG1 TYR_249@H TYR_249@N 4 0.0040 2.8860 136.7814

GLN_147@OE1 LYS_149@HZ2 LYS_149@NZ 4 0.0040 2.8871 156.5265

ASP_150@O LYS_149@H LYS_149@N 4 0.0040 2.8874 149.7970

LYS_149@O GLN_379@HE21 GLN_379@NE2 4 0.0040 2.8921 150.5498

HIE_366@ND1 ARG_164@HH12 ARG_164@NH1 4 0.0040 2.8930 142.1252

GLU_331@OE1 ARG_441@HE ARG_441@NE 4 0.0040 2.8943 151.2796

ASN_383@OD1 GLN_285@HE21 GLN_285@NE2 4 0.0040 2.9003 164.0784

GLN_424@O TRP_422@HE1 TRP_422@NE1 4 0.0040 2.9028 140.7164

GLU_437@OE1 ARG_408@HE ARG_408@NE 4 0.0040 2.9038 153.3924

GLN_55@O LYS_54@HZ1 LYS_54@NZ 4 0.0040 2.9043 160.3419

ASN_190@O SER_191@HG SER_191@OG 4 0.0040 2.9094 151.9005

SER_435@OG ARG_408@HH11 ARG_408@NH1 4 0.0040 2.9132 147.7804

GLN_147@OE1 SER_148@H SER_148@N 4 0.0040 2.9142 141.1080

GLN_424@OE1 ASP_425@H ASP_425@N 4 0.0040 2.9148 152.3151

GLN_142@OE1 GLN_14@HE22 GLN_14@NE2 4 0.0040 2.9160 157.1536

ASP_140@O GLN_142@H GLN_142@N 4 0.0040 2.9191 142.5999

GLN_412@OE1 PHE_413@H PHE_413@N 4 0.0040 2.9192 144.9807

CYX_184@O ASN_186@H ASN_186@N 4 0.0040 2.9209 138.8412

ASP_264@OD1 ARG_282@HH22 ARG_282@NH2 4 0.0040 2.9223 160.9634

GLY_440@O ARG_441@HH11 ARG_441@NH1 4 0.0040 2.9248 153.2481

ASP_264@O PHE_266@H PHE_266@N 4 0.0040 2.9256 139.7230

CYX_159@O ARG_392@HH21 ARG_392@NH2 4 0.0040 2.9280 141.1620

SER_129@OG ARG_124@HH22 ARG_124@NH2 4 0.0040 2.9318 141.0233

PHE_266@O ALA_268@H ALA_268@N 4 0.0040 2.9346 142.5700

TYR_291@OH ARG_237@HH11 ARG_237@NH1 4 0.0040 2.9389 158.9788

THR_239@OG1 ARG_237@HH12 ARG_237@NH1 4 0.0040 2.9424 152.0500

SER_330@OG ALA_332@H ALA_332@N 4 0.0040 2.9463 154.6915

MET_163@O ARG_164@HH21 ARG_164@NH2 4 0.0040 2.9496 146.8769

SER_141@OG ILE_113@H ILE_113@N 4 0.0040 2.9514 162.7857

TYR_249@O GLU_257@H GLU_257@N 4 0.0040 2.9515 156.8624

GLN_401@OE1 ASP_443@H ASP_443@N 4 0.0040 2.9527 145.7417

ILE_113@O SER_141@H SER_141@N 4 0.0040 2.9548 160.0788

GLU_331@OE2 ARG_441@HH21 ARG_441@NH2 4 0.0040 2.9694 150.1582

GLU_378@OE1 TYR_154@HH TYR_154@OH 3 0.0030 2.6712 158.9075

ASP_150@O SER_148@HG SER_148@OG 3 0.0030 2.6937 149.9690

ASN_56@O ARG_58@H ARG_58@N 3 0.0030 2.7276 143.0103

ASP_125@OD2 SER_126@HG SER_126@OG 3 0.0030 2.7348 162.6194

GLN_114@OE1 LYS_169@HZ1 LYS_169@NZ 3 0.0030 2.7395 148.3427

ASP_315@OD1 LYS_215@HZ1 LYS_215@NZ 3 0.0030 2.7437 158.3783

GLU_314@OE1 LYS_215@HZ3 LYS_215@NZ 3 0.0030 2.7533 152.7333

ASP_264@OD2 SER_263@HG SER_263@OG 3 0.0030 2.7554 164.3855

GLY_299@O SER_296@HG SER_296@OG 3 0.0030 2.7623 166.9396

PHE_188@O SER_191@HG SER_191@OG 3 0.0030 2.7631 151.7231

ASP_125@OD2 SER_126@H SER_126@N 3 0.0030 2.7643 137.5468

GLU_5@OE2 GLU_5@H GLU_5@N 3 0.0030 2.7681 143.1529

ASN_419@OD1 SER_417@HG SER_417@OG 3 0.0030 2.7759 160.5379

SER_60@O SER_60@HG SER_60@OG 3 0.0030 2.7795 143.5934

GLU_257@OE1 LYS_258@HZ1 LYS_258@NZ 3 0.0030 2.7795 163.0163

ASP_225@OD1 ARG_223@HH12 ARG_223@NH1 3 0.0030 2.7844 143.9056

SER_128@O SER_129@HG SER_129@OG 3 0.0030 2.7868 156.5659

ALA_346@O THR_347@HG1 THR_347@OG1 3 0.0030 2.7964 150.9278

GLU_418@OE2 LYS_428@HZ2 LYS_428@NZ 3 0.0030 2.7966 152.2795

ASN_38@O TRP_40@H TRP_40@N 3 0.0030 2.7968 142.1532

ASP_315@OD2 LYS_215@HZ1 LYS_215@NZ 3 0.0030 2.7977 158.2473

GLU_437@OE1 ARG_408@HH21 ARG_408@NH2 3 0.0030 2.7997 149.6953

ASN_178@OD1 LYS_131@HZ1 LYS_131@NZ 3 0.0030 2.8028 151.3794

ASP_162@OD2 ARG_164@HH11 ARG_164@NH1 3 0.0030 2.8164 149.0497

SER_151@O TYR_154@HH TYR_154@OH 3 0.0030 2.8230 151.1242

GLN_147@OE1 LYS_149@HZ3 LYS_149@NZ 3 0.0030 2.8268 165.7049

ALA_65@O ARG_68@HH22 ARG_68@NH2 3 0.0030 2.8332 139.8030

LYS_258@O ARG_260@HH21 ARG_260@NH2 3 0.0030 2.8347 154.3879

GLU_284@OE1 ARG_282@HH12 ARG_282@NH1 3 0.0030 2.8356 149.4745

ASN_115@OD1 GLN_114@HE22 GLN_114@NE2 3 0.0030 2.8405 156.7023

ASP_162@O ARG_164@HH21 ARG_164@NH2 3 0.0030 2.8414 150.4166

ASP_152@O LYS_179@HZ1 LYS_179@NZ 3 0.0030 2.8436 155.2045

LYS_258@O LYS_258@HZ2 LYS_258@NZ 3 0.0030 2.8438 150.5461

GLU_257@O GLN_255@HE22 GLN_255@NE2 3 0.0030 2.8446 143.1939

GLN_401@OE1 ARG_441@HH12 ARG_441@NH1 3 0.0030 2.8459 151.6659

ASN_144@O SER_146@HG SER_146@OG 3 0.0030 2.8464 162.9105

GLU_245@OE2 THR_239@H THR_239@N 3 0.0030 2.8491 146.2826

VAL_205@O GLN_207@H GLN_207@N 3 0.0030 2.8507 142.9096

ASN_252@OD1 GLN_251@HE21 GLN_251@NE2 3 0.0030 2.8517 149.2493

PRO_380@O LYS_377@HZ2 LYS_377@NZ 3 0.0030 2.8522 148.8046

ALA_442@O ARG_441@HH21 ARG_441@NH2 3 0.0030 2.8551 139.9937

ASN_219@OD1 ARG_282@HH22 ARG_282@NH2 3 0.0030 2.8563 145.3047

GLN_114@OE1 LYS_169@HZ3 LYS_169@NZ 3 0.0030 2.8578 152.5554

ASP_402@OD2 ARG_404@HH11 ARG_404@NH1 3 0.0030 2.8584 149.4770

LYS_149@O LYS_149@HZ1 LYS_149@NZ 3 0.0030 2.8588 146.9249

GLY_298@O ARG_232@HH21 ARG_232@NH2 3 0.0030 2.8624 150.5329

SER_259@O SER_259@HG SER_259@OG 3 0.0030 2.8625 141.8953

ASN_383@OD1 ASN_383@H ASN_383@N 3 0.0030 2.8629 137.2701

GLU_437@OE1 ARG_408@HH11 ARG_408@NH1 3 0.0030 2.8670 151.3092

HIE_211@O LYS_212@HZ3 LYS_212@NZ 3 0.0030 2.8679 153.0436

ASP_162@O ARG_164@HH12 ARG_164@NH1 3 0.0030 2.8729 142.9340

ASP_117@OD1 ASN_115@HD21 ASN_115@ND2 3 0.0030 2.8731 158.6189

GLN_42@O ILE_44@H ILE_44@N 3 0.0030 2.8734 143.9559

ILE_113@O ASN_115@H ASN_115@N 3 0.0030 2.8737 141.1613

PRO_375@O GLN_147@HE21 GLN_147@NE2 3 0.0030 2.8765 150.3896

PRO_403@O ARG_404@HH21 ARG_404@NH2 3 0.0030 2.8769 142.1323

GLU_5@OE2 GLN_3@HE22 GLN_3@NE2 3 0.0030 2.8776 164.7077

GLY_104@O ARG_106@HH12 ARG_106@NH1 3 0.0030 2.8810 136.9627

GLU_269@OE1 GLU_269@H GLU_269@N 3 0.0030 2.8821 144.2775

ASP_130@O LYS_131@HZ2 LYS_131@NZ 3 0.0030 2.8822 147.6865

ASP_162@O SER_367@HG SER_367@OG 3 0.0030 2.8830 154.0932

GLU_279@OE1 THR_221@HG1 THR_221@OG1 3 0.0030 2.8874 150.5731

VAL_360@O LYS_363@HZ2 LYS_363@NZ 3 0.0030 2.8899 158.1498

TYR_291@OH ARG_237@HH21 ARG_237@NH2 3 0.0030 2.8899 145.8456

ALA_381@O GLN_379@HE21 GLN_379@NE2 3 0.0030 2.8910 155.6438

ASN_186@OD1 ASN_189@HD21 ASN_189@ND2 3 0.0030 2.8940 161.2669

GLU_437@OE2 ARG_408@HH21 ARG_408@NH2 3 0.0030 2.8994 153.3940

ALA_332@O SER_335@H SER_335@N 3 0.0030 2.8997 151.0419

THR_239@OG1 GLN_242@HE22 GLN_242@NE2 3 0.0030 2.9017 170.5579

ASN_24@OD1 ARG_22@HH12 ARG_22@NH1 3 0.0030 2.9065 145.3924

GLN_147@OE1 LYS_149@HZ1 LYS_149@NZ 3 0.0030 2.9071 160.8183

SER_76@OG SER_75@HG SER_75@OG 3 0.0030 2.9102 167.3797

GLU_257@OE2 LYS_98@HZ3 LYS_98@NZ 3 0.0030 2.9112 171.4251

LYS_149@O LYS_149@HZ2 LYS_149@NZ 3 0.0030 2.9140 168.7762

ASN_219@OD1 ARG_282@HE ARG_282@NE 3 0.0030 2.9144 164.8235

GLU_314@OE1 LYS_215@HZ2 LYS_215@NZ 3 0.0030 2.9155 158.1362

ASN_112@OD1 GLN_142@HE21 GLN_142@NE2 3 0.0030 2.9161 163.5748

GLU_279@OE1 ASN_219@HD22 ASN_219@ND2 3 0.0030 2.9169 164.2677

ARG_260@O ARG_260@HE ARG_260@NE 3 0.0030 2.9170 147.1713

GLY_362@O LYS_363@HZ1 LYS_363@NZ 3 0.0030 2.9179 159.8811

ASP_10@OD1 ARG_106@HH22 ARG_106@NH2 3 0.0030 2.9185 147.7737

ASN_24@OD1 GLN_3@HE22 GLN_3@NE2 3 0.0030 2.9206 165.0420

ASP_125@O ARG_124@HH11 ARG_124@NH1 3 0.0030 2.9263 165.2010

ARG_282@O ARG_282@HH11 ARG_282@NH1 3 0.0030 2.9287 150.8772

THR_423@OG1 GLN_424@HE22 GLN_424@NE2 3 0.0030 2.9320 153.5691

SER_201@OG ALA_185@H ALA_185@N 3 0.0030 2.9323 153.4620

GLN_424@OE1 ARG_426@H ARG_426@N 3 0.0030 2.9342 159.6741

SER_357@OG GLN_410@HE22 GLN_410@NE2 3 0.0030 2.9361 155.5684

ASP_125@OD1 SER_126@HG SER_126@OG 3 0.0030 2.9405 148.1185

ARG_164@O ARG_164@HH11 ARG_164@NH1 3 0.0030 2.9407 153.5451

CYX_370@O ARG_392@HE ARG_392@NE 3 0.0030 2.9413 152.8290

GLY_348@O ARG_386@HH11 ARG_386@NH1 3 0.0030 2.9427 147.0385

GLN_114@NE2 ASN_112@HD21 ASN_112@ND2 3 0.0030 2.9514 143.5908

GLU_15@OE2 THR_79@H THR_79@N 3 0.0030 2.9518 153.0375

SER_259@OG ARG_237@HH21 ARG_237@NH2 3 0.0030 2.9528 142.3601

GLN_410@NE2 TRP_359@HE1 TRP_359@NE1 3 0.0030 2.9623 157.3062

THR_20@OG1 ARG_22@HH21 ARG_22@NH2 3 0.0030 2.9654 138.7038

GLY_305@O GLY_307@H GLY_307@N 3 0.0030 2.9688 138.6249

TYR_154@OH ASN_178@HD21 ASN_178@ND2 3 0.0030 2.9688 152.3455

THR_214@OG1 LYS_212@HZ3 LYS_212@NZ 3 0.0030 2.9743 145.8600

VAL_89@O SER_91@HG SER_91@OG 3 0.0030 2.9766 148.6506

PRO_403@O ARG_404@HH11 ARG_404@NH1 3 0.0030 2.9784 147.7571

GLU_279@O GLN_281@HE21 GLN_281@NE2 3 0.0030 2.9820 153.5335

SER_151@O SER_151@HG SER_151@OG 2 0.0020 2.6868 136.1782

ASN_171@O THR_137@HG1 THR_137@OG1 2 0.0020 2.6877 168.7373

LEU_261@O SER_259@HG SER_259@OG 2 0.0020 2.7002 161.8119

ASP_152@O LYS_179@HZ3 LYS_179@NZ 2 0.0020 2.7018 158.6105

GLN_122@OE1 SER_132@HG SER_132@OG 2 0.0020 2.7098 152.0811

ASP_125@O SER_129@HG SER_129@OG 2 0.0020 2.7213 175.3061

GLN_379@O LYS_377@HZ2 LYS_377@NZ 2 0.0020 2.7216 145.1074

ASP_352@OD2 TYR_387@HH TYR_387@OH 2 0.0020 2.7237 155.3393

GLN_55@O LYS_54@HZ2 LYS_54@NZ 2 0.0020 2.7263 156.6528

GLN_379@O LYS_377@HZ1 LYS_377@NZ 2 0.0020 2.7284 152.5632

ASP_162@OD2 SER_367@HG SER_367@OG 2 0.0020 2.7311 153.8297

ASP_352@OD1 TYR_387@HH TYR_387@OH 2 0.0020 2.7352 158.1450

GLU_378@OE1 THR_347@HG1 THR_347@OG1 2 0.0020 2.7426 148.7032

CYX_370@O THR_371@HG1 THR_371@OG1 2 0.0020 2.7486 140.8947

ASN_189@OD1 ASN_190@H ASN_190@N 2 0.0020 2.7513 138.3482

GLN_218@OE1 THR_214@HG1 THR_214@OG1 2 0.0020 2.7541 168.7190

ASP_315@OD1 GLN_424@HE22 GLN_424@NE2 2 0.0020 2.7570 148.1690

GLU_257@OE2 LYS_258@HZ3 LYS_258@NZ 2 0.0020 2.7603 157.1549

ALA_332@O TYR_121@HH TYR_121@OH 2 0.0020 2.7649 159.2942

GLN_255@OE1 THR_248@HG1 THR_248@OG1 2 0.0020 2.7669 157.1536

GLY_362@O LYS_363@HZ3 LYS_363@NZ 2 0.0020 2.7713 152.3685

GLU_418@OE2 LYS_428@HZ1 LYS_428@NZ 2 0.0020 2.7728 154.9958

SER_295@O SER_296@HG SER_296@OG 2 0.0020 2.7796 159.3967

ASN_178@O ASN_178@HD22 ASN_178@ND2 2 0.0020 2.7881 139.2584

GLY_204@O THR_203@HG1 THR_203@OG1 2 0.0020 2.7904 152.7067

GLY_307@O THR_308@HG1 THR_308@OG1 2 0.0020 2.7950 152.9216

VAL_324@O LYS_127@HZ2 LYS_127@NZ 2 0.0020 2.7972 158.1138

GLN_255@O LYS_258@HZ1 LYS_258@NZ 2 0.0020 2.8070 143.5081

GLU_437@OE1 TRP_439@HE1 TRP_439@NE1 2 0.0020 2.8081 145.8194

ASP_402@O ASN_405@HD22 ASN_405@ND2 2 0.0020 2.8085 152.0817

ASP_425@OD2 GLN_424@HE21 GLN_424@NE2 2 0.0020 2.8093 152.2829

PRO_202@O SER_201@HG SER_201@OG 2 0.0020 2.8118 160.9160

SER_177@OG LYS_179@HZ3 LYS_179@NZ 2 0.0020 2.8124 147.7400

GLN_3@O GLN_3@HE21 GLN_3@NE2 2 0.0020 2.8176 143.1477

PHE_266@O LYS_258@HZ2 LYS_258@NZ 2 0.0020 2.8200 157.2338

ASP_443@O ARG_441@HH12 ARG_441@NH1 2 0.0020 2.8208 164.0198

ASP_384@O ARG_386@HH21 ARG_386@NH2 2 0.0020 2.8230 153.4638

PRO_202@OXT ARG_124@HH12 ARG_124@NH1 2 0.0020 2.8233 156.5658

SER_141@OG ASN_171@HD22 ASN_171@ND2 2 0.0020 2.8235 139.2742

LEU_262@O ARG_260@HH21 ARG_260@NH2 2 0.0020 2.8237 143.6429

THR_66@O ARG_68@HH21 ARG_68@NH2 2 0.0020 2.8256 150.4955

PRO_375@O GLN_374@HE21 GLN_374@NE2 2 0.0020 2.8261 143.8763

ASP_425@O ARG_426@HH21 ARG_426@NH2 2 0.0020 2.8266 141.6033

GLU_333@OE2 THR_341@H THR_341@N 2 0.0020 2.8283 139.4519

ASP_162@O ARG_164@H ARG_164@N 2 0.0020 2.8327 141.2521

ASP_443@OXT ARG_441@HH22 ARG_441@NH2 2 0.0020 2.8333 146.6582

GLU_5@OE2 VAL_4@H VAL_4@N 2 0.0020 2.8351 150.9241

GLN_374@OE1 ARG_106@HH22 ARG_106@NH2 2 0.0020 2.8352 152.2698

GLN_424@NE2 THR_423@HG1 THR_423@OG1 2 0.0020 2.8361 146.7143

GLU_15@OE1 THR_79@H THR_79@N 2 0.0020 2.8374 162.1937

GLU_269@OE2 GLU_269@H GLU_269@N 2 0.0020 2.8374 141.1630

VAL_133@O GLN_122@HE22 GLN_122@NE2 2 0.0020 2.8385 148.1076

ILE_74@O SER_19@HG SER_19@OG 2 0.0020 2.8388 153.5899

THR_239@OG1 ARG_237@HH11 ARG_237@NH1 2 0.0020 2.8402 147.5080

PRO_202@OXT ARG_124@HH22 ARG_124@NH2 2 0.0020 2.8405 146.1680

ASP_315@O LYS_317@H LYS_317@N 2 0.0020 2.8421 138.1074

GLN_338@O LYS_339@HZ2 LYS_339@NZ 2 0.0020 2.8429 153.6872

ASP_150@O SER_151@HG SER_151@OG 2 0.0020 2.8429 158.3312

ASP_167@OD1 ARG_164@HH12 ARG_164@NH1 2 0.0020 2.8435 161.3656

ASN_186@OD1 LYS_179@HZ1 LYS_179@NZ 2 0.0020 2.8438 148.9893

GLU_421@OE2 LYS_215@HZ1 LYS_215@NZ 2 0.0020 2.8448 148.8050

ASN_252@OD1 GLU_253@H GLU_253@N 2 0.0020 2.8458 138.4087

PHE_266@O LYS_258@HZ3 LYS_258@NZ 2 0.0020 2.8468 145.7724

ASP_264@OD2 ARG_282@HH22 ARG_282@NH2 2 0.0020 2.8503 160.0956

THR_137@O PHE_139@H PHE_139@N 2 0.0020 2.8515 143.0513

LYS_212@O THR_214@HG1 THR_214@OG1 2 0.0020 2.8526 156.9367

GLN_114@OE1 ASN_112@HD22 ASN_112@ND2 2 0.0020 2.8546 136.1639

GLU_314@OE1 LYS_215@HZ1 LYS_215@NZ 2 0.0020 2.8557 150.7012

ASP_225@OD2 ARG_223@HH12 ARG_223@NH1 2 0.0020 2.8557 149.7708

ASP_372@OD2 ARG_392@HH12 ARG_392@NH1 2 0.0020 2.8572 142.6546

TYR_387@OH LYS_377@HZ3 LYS_377@NZ 2 0.0020 2.8590 155.5541

ASP_384@OD1 ASN_383@HD21 ASN_383@ND2 2 0.0020 2.8590 155.6897

GLN_147@O GLN_147@HE22 GLN_147@NE2 2 0.0020 2.8592 149.1597

SER_75@OG TYR_73@HH TYR_73@OH 2 0.0020 2.8594 155.7913

GLN_424@OE1 LYS_317@HZ3 LYS_317@NZ 2 0.0020 2.8598 162.2060

GLN_255@O GLN_255@HE22 GLN_255@NE2 2 0.0020 2.8609 158.7136

ASP_352@OD2 GLN_374@HE21 GLN_374@NE2 2 0.0020 2.8652 160.2375

ALA_427@O ARG_426@HH11 ARG_426@NH1 2 0.0020 2.8665 149.8689

GLN_338@O LYS_339@HZ1 LYS_339@NZ 2 0.0020 2.8694 158.1020

GLN_242@OE1 GLY_104@H GLY_104@N 2 0.0020 2.8698 144.3420

ARG_216@O ARG_216@HH11 ARG_216@NH1 2 0.0020 2.8713 154.4052

ARG_237@O ARG_237@HH11 ARG_237@NH1 2 0.0020 2.8719 158.9362

LEU_416@O LYS_428@HZ1 LYS_428@NZ 2 0.0020 2.8744 169.4026

GLY_104@O THR_103@HG1 THR_103@OG1 2 0.0020 2.8746 159.4666

GLN_281@OE1 ARG_282@HH21 ARG_282@NH2 2 0.0020 2.8750 150.2500

GLU_245@OE2 GLN_242@HE22 GLN_242@NE2 2 0.0020 2.8750 155.7103

GLN_142@O ASN_144@H ASN_144@N 2 0.0020 2.8756 142.8260

GLU_67@OE2 GLU_67@H GLU_67@N 2 0.0020 2.8781 150.4214

GLY_41@O GLN_42@HE22 GLN_42@NE2 2 0.0020 2.8792 173.4978

ASN_208@OD1 ARG_223@HH21 ARG_223@NH2 2 0.0020 2.8801 163.4056

ASP_443@OXT ARG_441@HH12 ARG_441@NH1 2 0.0020 2.8805 148.0970

LYS_317@O ARG_426@HH12 ARG_426@NH1 2 0.0020 2.8805 138.1444

GLY_95@O GLN_251@HE22 GLN_251@NE2 2 0.0020 2.8809 146.6792

ALA_185@O ASN_189@H ASN_189@N 2 0.0020 2.8819 149.9894

GLU_301@OE1 TYR_300@H TYR_300@N 2 0.0020 2.8827 149.4037

MET_163@O ARG_164@HE ARG_164@NE 2 0.0020 2.8834 157.6919

THR_283@O THR_283@HG1 THR_283@OG1 2 0.0020 2.8837 141.1180

ASP_384@OD2 ASN_383@HD22 ASN_383@ND2 2 0.0020 2.8850 158.7160

SER_267@OG ALA_268@H ALA_268@N 2 0.0020 2.8854 137.7326

GLU_5@OE1 GLN_3@HE22 GLN_3@NE2 2 0.0020 2.8858 159.8196

SER_296@O ASN_231@H ASN_231@N 2 0.0020 2.8858 145.5988

ASP_384@OD2 ASN_318@HD22 ASN_318@ND2 2 0.0020 2.8861 145.5775

ASP_117@OD1 ASN_115@HD22 ASN_115@ND2 2 0.0020 2.8874 159.2839

ASP_287@O ARG_237@HH21 ARG_237@NH2 2 0.0020 2.8886 155.2840

LEU_107@O ARG_106@HH11 ARG_106@NH1 2 0.0020 2.8890 153.0080

LEU_356@O SER_357@HG SER_357@OG 2 0.0020 2.8900 152.6408

GLU_245@OE1 THR_239@H THR_239@N 2 0.0020 2.8925 167.5372

GLU_245@OE2 ARG_237@HH12 ARG_237@NH1 2 0.0020 2.8928 157.6767

PRO_9@O ARG_106@HH21 ARG_106@NH2 2 0.0020 2.8936 141.5397

ASP_152@OD2 LYS_179@HZ3 LYS_179@NZ 2 0.0020 2.8964 157.3834

THR_20@OG1 ARG_22@HH11 ARG_22@NH1 2 0.0020 2.8971 146.4385

GLY_241@O ARG_106@HH12 ARG_106@NH1 2 0.0020 2.8981 154.0126

SER_28@OG ARG_68@HE ARG_68@NE 2 0.0020 2.8986 145.0998

SER_126@O ARG_441@HH12 ARG_441@NH1 2 0.0020 2.9012 138.3324

GLU_437@OE2 ARG_408@HE ARG_408@NE 2 0.0020 2.9024 151.9215

SER_94@OG ASN_31@HD21 ASN_31@ND2 2 0.0020 2.9029 150.8471

LYS_149@O GLN_379@HE22 GLN_379@NE2 2 0.0020 2.9032 154.1678

ASP_402@OD1 ASN_405@HD21 ASN_405@ND2 2 0.0020 2.9065 145.8474

GLN_401@OE1 ARG_441@HH22 ARG_441@NH2 2 0.0020 2.9078 148.0206

TYR_291@OH ARG_237@HE ARG_237@NE 2 0.0020 2.9095 150.6172

GLN_338@O LYS_339@HZ3 LYS_339@NZ 2 0.0020 2.9099 140.0570

SER_94@OG ASN_30@HD21 ASN_30@ND2 2 0.0020 2.9107 142.5100

GLU_378@OE2 GLN_379@HE22 GLN_379@NE2 2 0.0020 2.9161 171.7255

ASP_10@OD2 LEU_11@H LEU_11@N 2 0.0020 2.9181 140.1733

GLU_284@OE1 ARG_282@HH11 ARG_282@NH1 2 0.0020 2.9182 165.2747

ASP_315@OD2 LYS_215@HZ3 LYS_215@NZ 2 0.0020 2.9184 153.1588

SER_128@O LYS_131@HZ3 LYS_131@NZ 2 0.0020 2.9187 146.4175

PRO_380@O LYS_377@HZ1 LYS_377@NZ 2 0.0020 2.9187 152.8557

SER_146@OG ASN_144@HD21 ASN_144@ND2 2 0.0020 2.9205 163.2706

ALA_185@O ASN_189@HD22 ASN_189@ND2 2 0.0020 2.9210 154.6432

THR_80@O SER_82@H SER_82@N 2 0.0020 2.9212 147.8900

GLN_374@O GLN_147@HE21 GLN_147@NE2 2 0.0020 2.9217 147.5393

ASP_150@OD2 LYS_149@H LYS_149@N 2 0.0020 2.9221 153.4828

TRP_40@O ARG_309@HH22 ARG_309@NH2 2 0.0020 2.9224 138.7539

SER_141@O GLN_142@HE22 GLN_142@NE2 2 0.0020 2.9242 156.0389

GLU_331@OE2 ARG_441@HE ARG_441@NE 2 0.0020 2.9245 151.4827

TYR_414@OH GLN_412@HE22 GLN_412@NE2 2 0.0020 2.9247 167.8109

GLU_378@O SER_151@H SER_151@N 2 0.0020 2.9270 147.5795

ASN_178@OD1 LYS_131@HZ3 LYS_131@NZ 2 0.0020 2.9272 159.0356

HIE_366@ND1 ARG_164@HH22 ARG_164@NH2 2 0.0020 2.9308 149.0148

GLN_242@NE2 THR_239@HG1 THR_239@OG1 2 0.0020 2.9310 151.3976

ASP_264@OD2 ARG_260@HH22 ARG_260@NH2 2 0.0020 2.9318 147.8629

THR_221@O ARG_223@HH22 ARG_223@NH2 2 0.0020 2.9319 142.4232

ASP_425@O ARG_426@HE ARG_426@NE 2 0.0020 2.9322 156.2639

ASP_181@O LYS_179@HZ3 LYS_179@NZ 2 0.0020 2.9347 141.5165

GLN_122@O ARG_124@HH21 ARG_124@NH2 2 0.0020 2.9360 143.4393

ASP_162@O ARG_164@HE ARG_164@NE 2 0.0020 2.9360 149.5470

SER_146@OG ASN_190@HD22 ASN_190@ND2 2 0.0020 2.9391 148.0182

PHE_25@O ARG_68@HH21 ARG_68@NH2 2 0.0020 2.9412 142.2481

LEU_310@O ARG_309@HE ARG_309@NE 2 0.0020 2.9438 141.6014

SER_263@OG ARG_265@H ARG_265@N 2 0.0020 2.9447 157.5352

GLY_217@O GLN_218@HE22 GLN_218@NE2 2 0.0020 2.9457 154.6057

GLN_114@OE1 ILE_113@H ILE_113@N 2 0.0020 2.9533 145.6878

THR_239@OG1 ARG_237@HH21 ARG_237@NH2 2 0.0020 2.9540 167.1640

THR_371@O THR_371@HG1 THR_371@OG1 2 0.0020 2.9541 144.6848

GLN_401@O ARG_441@HH21 ARG_441@NH2 2 0.0020 2.9552 164.3734

SER_417@OG ASN_419@HD22 ASN_419@ND2 2 0.0020 2.9556 149.1348

GLN_379@O ALA_381@H ALA_381@N 2 0.0020 2.9557 142.8327

ASP_10@OD1 ARG_106@HH12 ARG_106@NH1 2 0.0020 2.9589 155.9303

GLN_33@NE2 GLN_302@H GLN_302@N 2 0.0020 2.9622 135.5505

ASN_190@ND2 ASN_190@H ASN_190@N 2 0.0020 2.9622 139.8519

SER_259@O LEU_261@H LEU_261@N 2 0.0020 2.9662 147.4749

GLU_331@O ILE_334@H ILE_334@N 2 0.0020 2.9668 142.6066

HIE_366@ND1 ARG_164@HH11 ARG_164@NH1 2 0.0020 2.9676 140.7390

SER_417@OG LYS_212@HZ1 LYS_212@NZ 2 0.0020 2.9814 138.4449

SER_435@OG GLN_410@HE22 GLN_410@NE2 2 0.0020 2.9849 148.7766

SER_60@OG GLN_55@HE22 GLN_55@NE2 2 0.0020 2.9869 158.1884

SER_28@OG ARG_68@HH22 ARG_68@NH2 2 0.0020 2.9940 159.2857

GLU_355@OE1 SER_357@HG SER_357@OG 1 0.0010 2.5231 147.2009

GLU_301@OE2 TYR_303@HH TYR_303@OH 1 0.0010 2.5481 176.7864

SER_146@OG SER_191@HG SER_191@OG 1 0.0010 2.5484 169.1953

VAL_109@O LYS_108@HZ2 LYS_108@NZ 1 0.0010 2.5784 136.1087

GLN_218@OE1 LYS_215@HZ1 LYS_215@NZ 1 0.0010 2.5889 140.7627

SER_367@O SER_367@HG SER_367@OG 1 0.0010 2.5984 154.7368

GLU_279@OE2 THR_277@HG1 THR_277@OG1 1 0.0010 2.5993 151.7511

GLN_281@OE1 SER_267@HG SER_267@OG 1 0.0010 2.6084 159.3128

ASP_138@OD2 SER_170@HG SER_170@OG 1 0.0010 2.6099 165.0657

GLU_279@OE2 ASN_219@HD22 ASN_219@ND2 1 0.0010 2.6172 159.1648

ASP_162@OD1 THR_79@HG1 THR_79@OG1 1 0.0010 2.6217 143.3993

ASN_419@O ASN_419@HD22 ASN_419@ND2 1 0.0010 2.6304 138.2382

ASN_18@OD1 TYR_73@HH TYR_73@OH 1 0.0010 2.6308 152.9798

HIE_366@O SER_367@HG SER_367@OG 1 0.0010 2.6370 153.5602

ASP_384@OD1 ASP_384@H ASP_384@N 1 0.0010 2.6447 136.3851

ASP_264@OD1 LYS_258@HZ3 LYS_258@NZ 1 0.0010 2.6537 152.3462

THR_203@OG1 SER_206@HG SER_206@OG 1 0.0010 2.6593 143.0063

LYS_377@O TYR_154@HH TYR_154@OH 1 0.0010 2.6698 168.7711

GLU_331@OE1 SER_330@HG SER_330@OG 1 0.0010 2.6756 142.3799

VAL_312@O TYR_350@HH TYR_350@OH 1 0.0010 2.6848 148.1152

ASN_24@OD1 SER_26@HG SER_26@OG 1 0.0010 2.6875 146.3224

SER_94@O TYR_97@HH TYR_97@OH 1 0.0010 2.6897 155.8865

GLU_5@O ASN_24@HD22 ASN_24@ND2 1 0.0010 2.6968 136.1273

ASN_231@OD1 ASN_252@HD22 ASN_252@ND2 1 0.0010 2.6980 144.7850

GLU_421@O LYS_428@HZ2 LYS_428@NZ 1 0.0010 2.6983 156.3621

GLN_251@OE1 ARG_232@HH12 ARG_232@NH1 1 0.0010 2.7055 164.7997

GLN_14@O SER_77@HG SER_77@OG 1 0.0010 2.7058 151.2201

LEU_256@O LYS_258@HZ2 LYS_258@NZ 1 0.0010 2.7082 165.1057

SER_141@O SER_141@HG SER_141@OG 1 0.0010 2.7098 139.3188

GLN_3@OE1 GLN_3@H GLN_3@N 1 0.0010 2.7157 135.8176

VAL_434@O SER_435@HG SER_435@OG 1 0.0010 2.7199 144.5530

ASN_383@O SER_385@HG SER_385@OG 1 0.0010 2.7204 164.9928

ASN_112@OD1 LYS_169@HZ2 LYS_169@NZ 1 0.0010 2.7216 138.7884

LYS_377@O GLN_379@H GLN_379@N 1 0.0010 2.7259 137.9964

ASN_178@OD1 LYS_179@HZ2 LYS_179@NZ 1 0.0010 2.7266 155.9414

VAL_4@O THR_103@HG1 THR_103@OG1 1 0.0010 2.7289 174.8130

ASN_112@O ASN_112@HD22 ASN_112@ND2 1 0.0010 2.7400 145.2001

ASP_152@OD1 LYS_179@HZ2 LYS_179@NZ 1 0.0010 2.7412 173.8390

ASP_264@OD2 LYS_258@HZ3 LYS_258@NZ 1 0.0010 2.7439 141.5606

GLN_114@OE1 LYS_169@HZ2 LYS_169@NZ 1 0.0010 2.7464 146.9993

VAL_145@O LYS_158@HZ2 LYS_158@NZ 1 0.0010 2.7468 144.8868

GLU_437@OE2 ARG_408@HH12 ARG_408@NH1 1 0.0010 2.7490 140.8708

ASP_315@OD2 ASP_315@H ASP_315@N 1 0.0010 2.7504 135.8601

ASN_56@OD1 LYS_54@HZ2 LYS_54@NZ 1 0.0010 2.7539 139.4177

LYS_179@O ASP_181@H ASP_181@N 1 0.0010 2.7541 154.2729

GLU_421@OE1 LYS_215@HZ2 LYS_215@NZ 1 0.0010 2.7543 168.0972

SER_126@O LYS_127@HZ2 LYS_127@NZ 1 0.0010 2.7554 148.5842

GLN_379@OE1 SER_151@H SER_151@N 1 0.0010 2.7558 137.5311

GLU_364@OE2 HIE_366@HE2 HIE_366@NE2 1 0.0010 2.7560 142.3908

TYR_303@OH THR_203@H2 THR_203@N 1 0.0010 2.7577 167.0892

THR_20@OG1 LEU_21@H LEU_21@N 1 0.0010 2.7584 138.9661

ASN_208@OD1 ARG_223@HH12 ARG_223@NH1 1 0.0010 2.7621 158.8070

ASP_27@OD1 GLY_1@H1 GLY_1@N 1 0.0010 2.7694 144.0391

LEU_256@O LYS_258@HZ1 LYS_258@NZ 1 0.0010 2.7730 150.5652

ASP_150@O GLN_379@HE21 GLN_379@NE2 1 0.0010 2.7731 147.4661

ASP_117@OD2 ASN_115@HD22 ASN_115@ND2 1 0.0010 2.7732 141.2369

PHE_168@O LYS_169@HZ1 LYS_169@NZ 1 0.0010 2.7749 170.3777

LEU_261@O SER_263@HG SER_263@OG 1 0.0010 2.7755 169.7495

GLN_285@OE1 GLU_314@H GLU_314@N 1 0.0010 2.7770 141.9703

GLN_142@OE1 SER_141@H SER_141@N 1 0.0010 2.7807 143.3193

GLU_284@OE2 ARG_282@HH12 ARG_282@NH1 1 0.0010 2.7828 140.9557

ASP_167@OD1 ARG_164@HH22 ARG_164@NH2 1 0.0010 2.7833 144.0692

SER_148@O LYS_149@HZ3 LYS_149@NZ 1 0.0010 2.7843 172.7184

ASN_383@OD1 LYS_377@HZ2 LYS_377@NZ 1 0.0010 2.7862 140.6888

ILE_227@O GLU_229@H GLU_229@N 1 0.0010 2.7926 149.7634

GLN_424@OE1 LYS_317@HZ1 LYS_317@NZ 1 0.0010 2.7929 169.6879

VAL_360@O GLY_362@H GLY_362@N 1 0.0010 2.7966 137.6895

ASP_443@OD1 ALA_442@H ALA_442@N 1 0.0010 2.7975 158.5758

THR_239@OG1 ARG_237@HH22 ARG_237@NH2 1 0.0010 2.7980 150.6319

TYR_249@O GLN_255@HE22 GLN_255@NE2 1 0.0010 2.7986 161.5437

ASP_152@OD1 LYS_179@HZ1 LYS_179@NZ 1 0.0010 2.7989 139.0356

ALA_397@O PHE_399@H PHE_399@N 1 0.0010 2.8023 147.1337

THR_239@O GLY_241@H GLY_241@N 1 0.0010 2.8026 137.1418

SER_76@O GLN_78@HE21 GLN_78@NE2 1 0.0010 2.8039 151.5087

ASP_181@OD2 PHE_182@H PHE_182@N 1 0.0010 2.8044 147.9893

ASN_178@O LYS_179@HZ2 LYS_179@NZ 1 0.0010 2.8058 176.8948

SER_51@OG GLY_52@H GLY_52@N 1 0.0010 2.8083 135.9114

SER_128@O LYS_131@HZ1 LYS_131@NZ 1 0.0010 2.8084 153.9561

LEU_382@O ASP_384@H ASP_384@N 1 0.0010 2.8107 148.3638

GLN_122@OE1 ARG_124@HH11 ARG_124@NH1 1 0.0010 2.8118 157.4297

GLN_281@OE1 ARG_282@HE ARG_282@NE 1 0.0010 2.8122 166.2050

GLU_418@O ASP_420@H ASP_420@N 1 0.0010 2.8157 147.0974

ALA_65@O SER_28@HG SER_28@OG 1 0.0010 2.8171 141.7309

ASP_352@OD1 ARG_309@HH21 ARG_309@NH2 1 0.0010 2.8204 171.9070

SER_51@O LYS_54@HZ3 LYS_54@NZ 1 0.0010 2.8209 169.4430

SER_288@O ARG_309@HE ARG_309@NE 1 0.0010 2.8210 149.8065

LEU_256@O LYS_258@HZ3 LYS_258@NZ 1 0.0010 2.8212 165.9349

GLN_242@OE1 ARG_106@HH21 ARG_106@NH2 1 0.0010 2.8227 145.3406

ASN_190@O LYS_149@HZ1 LYS_149@NZ 1 0.0010 2.8233 155.6651

ASP_372@OD2 ARG_392@HH22 ARG_392@NH2 1 0.0010 2.8238 154.5769

GLN_424@OE1 LYS_317@HZ2 LYS_317@NZ 1 0.0010 2.8252 175.4141

ASP_225@OD1 ARG_223@HH22 ARG_223@NH2 1 0.0010 2.8290 148.0237

SER_259@O SER_263@HG SER_263@OG 1 0.0010 2.8297 159.8970

SER_385@OG ARG_386@H ARG_386@N 1 0.0010 2.8309 135.7067

PRO_375@O LYS_377@HZ1 LYS_377@NZ 1 0.0010 2.8317 164.1616

GLU_257@OE1 LYS_258@HZ2 LYS_258@NZ 1 0.0010 2.8326 153.6633

GLU_253@O GLN_255@HE22 GLN_255@NE2 1 0.0010 2.8336 174.3942

ASN_186@OD1 ASN_189@HD22 ASN_189@ND2 1 0.0010 2.8345 174.0213

ASN_419@O LYS_212@HZ1 LYS_212@NZ 1 0.0010 2.8356 139.3020

ASN_144@O SER_146@H SER_146@N 1 0.0010 2.8375 142.8677

VAL_360@O LYS_363@HZ3 LYS_363@NZ 1 0.0010 2.8384 150.2841

ASP_443@OD2 ALA_442@H ALA_442@N 1 0.0010 2.8403 170.0731

GLU_378@OE2 GLN_379@HE21 GLN_379@NE2 1 0.0010 2.8412 146.0693

THR_214@OG1 LYS_212@HZ1 LYS_212@NZ 1 0.0010 2.8412 166.2293

ASP_315@OD2 GLN_424@HE22 GLN_424@NE2 1 0.0010 2.8434 175.4736

ASN_189@OD1 ASN_186@HD22 ASN_186@ND2 1 0.0010 2.8439 160.1012

ILE_74@O SER_77@HG SER_77@OG 1 0.0010 2.8449 139.2420

ASP_10@OD2 ASP_10@H ASP_10@N 1 0.0010 2.8449 135.9184

SER_146@OG LYS_149@HZ1 LYS_149@NZ 1 0.0010 2.8454 141.9007

TRP_439@O ARG_441@HE ARG_441@NE 1 0.0010 2.8455 138.4277

ASN_186@O ASN_186@HD22 ASN_186@ND2 1 0.0010 2.8457 136.8457

VAL_324@O LYS_127@HZ1 LYS_127@NZ 1 0.0010 2.8500 150.2078

ASP_152@OD2 LYS_179@HZ2 LYS_179@NZ 1 0.0010 2.8523 168.5349

ASP_152@O LYS_179@HZ2 LYS_179@NZ 1 0.0010 2.8527 149.2006

SER_396@O SER_396@HG SER_396@OG 1 0.0010 2.8534 141.3851

PRO_329@O TRP_400@HE1 TRP_400@NE1 1 0.0010 2.8536 141.2531

GLN_142@OE1 ASN_115@HD21 ASN_115@ND2 1 0.0010 2.8548 159.6785

GLU_421@O LYS_428@HZ1 LYS_428@NZ 1 0.0010 2.8560 147.7041

GLN_410@O SER_357@HG SER_357@OG 1 0.0010 2.8572 138.1814

GLY_204@O SER_295@HG SER_295@OG 1 0.0010 2.8574 140.4563

ARG_232@NH1 ASN_231@HD22 ASN_231@ND2 1 0.0010 2.8583 143.6230

PHE_25@O ASN_24@HD21 ASN_24@ND2 1 0.0010 2.8597 136.2310

SER_151@O LYS_179@HZ2 LYS_179@NZ 1 0.0010 2.8601 138.0858

PRO_375@O LYS_377@HZ2 LYS_377@NZ 1 0.0010 2.8602 137.3713

GLU_378@OE1 ARG_386@HH12 ARG_386@NH1 1 0.0010 2.8605 159.5394

ASP_420@O TRP_422@H TRP_422@N 1 0.0010 2.8612 155.8284

GLN_142@O LYS_158@HZ3 LYS_158@NZ 1 0.0010 2.8614 139.6412

GLU_421@OE2 LYS_215@HZ3 LYS_215@NZ 1 0.0010 2.8643 175.3227

VAL_360@O LYS_363@HZ1 LYS_363@NZ 1 0.0010 2.8648 156.3584

ASP_27@OD1 GLY_1@H3 GLY_1@N 1 0.0010 2.8653 175.4212

GLU_314@OE2 LYS_215@HZ3 LYS_215@NZ 1 0.0010 2.8657 170.6610

SER_126@O ARG_124@HH21 ARG_124@NH2 1 0.0010 2.8672 135.1140

GLN_218@OE1 ARG_282@HH21 ARG_282@NH2 1 0.0010 2.8704 140.6121

ARG_22@O SER_7@HG SER_7@OG 1 0.0010 2.8755 174.3234

GLN_3@O GLU_5@H GLU_5@N 1 0.0010 2.8767 145.8909

SER_146@OG GLN_147@H GLN_147@N 1 0.0010 2.8784 136.5164

ALA_427@O TRP_422@HE1 TRP_422@NE1 1 0.0010 2.8795 136.6311

ASP_425@OD1 GLN_424@HE21 GLN_424@NE2 1 0.0010 2.8798 148.2261

ASP_140@OD2 ASN_115@HD22 ASN_115@ND2 1 0.0010 2.8813 152.7979

ASN_189@OD1 ASN_190@HD22 ASN_190@ND2 1 0.0010 2.8817 157.8948

ASP_152@OD2 LYS_179@HZ1 LYS_179@NZ 1 0.0010 2.8819 159.0595

GLU_418@OE2 ASN_419@HD21 ASN_419@ND2 1 0.0010 2.8820 136.2968

ASP_443@OXT ARG_441@HH11 ARG_441@NH1 1 0.0010 2.8834 168.6635

CYX_224@O ARG_223@HH11 ARG_223@NH1 1 0.0010 2.8837 144.4143

LEU_262@O ARG_260@HH22 ARG_260@NH2 1 0.0010 2.8869 140.7542

GLY_83@O ARG_106@HH11 ARG_106@NH1 1 0.0010 2.8877 140.3420

VAL_109@O LYS_108@HZ1 LYS_108@NZ 1 0.0010 2.8884 163.4754

GLU_437@OE2 TRP_439@HE1 TRP_439@NE1 1 0.0010 2.8892 152.7210

GLU_301@O GLN_33@HE21 GLN_33@NE2 1 0.0010 2.8933 144.0139

SER_128@OG SER_129@H SER_129@N 1 0.0010 2.8945 139.2277

THR_66@O ARG_68@HE ARG_68@NE 1 0.0010 2.8950 154.1390

ASP_384@OD1 ASN_318@HD22 ASN_318@ND2 1 0.0010 2.8959 154.9811

ASP_90@OD1 ASN_30@HD22 ASN_30@ND2 1 0.0010 2.8961 160.5935

GLN_251@NE2 ARG_232@HE ARG_232@NE 1 0.0010 2.8978 138.4686

SER_177@OG LYS_179@HZ1 LYS_179@NZ 1 0.0010 2.8980 145.7652

GLN_285@NE2 ASN_383@HD21 ASN_383@ND2 1 0.0010 2.9006 142.5364

TRP_40@O GLN_42@H GLN_42@N 1 0.0010 2.9009 137.8282

GLU_5@OE2 GLN_6@H GLN_6@N 1 0.0010 2.9039 142.2221

GLN_3@OE1 GLY_1@H2 GLY_1@N 1 0.0010 2.9058 142.9004

THR_239@O GLN_242@HE22 GLN_242@NE2 1 0.0010 2.9069 167.5012

TYR_387@O THR_347@HG1 THR_347@OG1 1 0.0010 2.9082 156.8921

PHE_182@O GLN_122@HE22 GLN_122@NE2 1 0.0010 2.9083 158.5357

ASN_144@OD1 GLN_147@HE22 GLN_147@NE2 1 0.0010 2.9088 146.0159

ASP_402@OD2 ASN_405@HD21 ASN_405@ND2 1 0.0010 2.9095 136.5200

ASP_264@OD2 ARG_260@HH12 ARG_260@NH1 1 0.0010 2.9095 136.5690

THR_197@O PHE_199@H PHE_199@N 1 0.0010 2.9100 151.9827

GLN_285@O GLN_285@HE22 GLN_285@NE2 1 0.0010 2.9107 135.1857

LYS_317@O LYS_317@HZ3 LYS_317@NZ 1 0.0010 2.9117 154.1466

ALA_442@O GLN_401@HE21 GLN_401@NE2 1 0.0010 2.9128 152.6209

GLN_218@NE2 ASN_219@H ASN_219@N 1 0.0010 2.9136 152.7585

ASN_208@O ARG_223@HH11 ARG_223@NH1 1 0.0010 2.9137 150.7043

SER_435@OG ARG_408@HH22 ARG_408@NH2 1 0.0010 2.9138 147.1581

GLU_378@OE1 ARG_386@HH22 ARG_386@NH2 1 0.0010 2.9138 147.3497

GLN_379@O LYS_377@HZ3 LYS_377@NZ 1 0.0010 2.9143 143.1723

GLU_245@OE1 ARG_237@HH22 ARG_237@NH2 1 0.0010 2.9144 136.8930

GLN_78@OE1 ARG_58@HE ARG_58@NE 1 0.0010 2.9147 137.0060

VAL_145@O LYS_158@HZ3 LYS_158@NZ 1 0.0010 2.9152 135.4343

ASP_425@OD1 ARG_426@HH12 ARG_426@NH1 1 0.0010 2.9160 141.1673

ASP_372@OD2 ASP_372@H ASP_372@N 1 0.0010 2.9179 135.7002

GLN_338@OE1 THR_398@HG1 THR_398@OG1 1 0.0010 2.9179 169.8504

THR_105@O LEU_107@H LEU_107@N 1 0.0010 2.9181 141.3446

CYX_23@O ASN_24@HD22 ASN_24@ND2 1 0.0010 2.9186 149.7311

LEU_123@O GLN_122@HE22 GLN_122@NE2 1 0.0010 2.9187 166.3219

ALA_185@O PHE_188@H PHE_188@N 1 0.0010 2.9207 161.1552

THR_431@O GLN_432@HE22 GLN_432@NE2 1 0.0010 2.9241 167.0420

ARG_124@NH1 SER_129@HG SER_129@OG 1 0.0010 2.9250 135.9395

SER_129@OG ARG_124@HH21 ARG_124@NH2 1 0.0010 2.9252 144.7171

GLN_218@OE1 LYS_215@HZ3 LYS_215@NZ 1 0.0010 2.9273 137.2351

SER_51@O LYS_54@HZ1 LYS_54@NZ 1 0.0010 2.9279 157.4990

ILE_433@O GLN_432@HE21 GLN_432@NE2 1 0.0010 2.9281 141.3630

SER_132@O GLN_122@HE22 GLN_122@NE2 1 0.0010 2.9282 146.8566

GLN_42@OE1 GLN_42@H GLN_42@N 1 0.0010 2.9283 147.7070

SER_396@OG SER_165@HG SER_165@OG 1 0.0010 2.9331 154.0871

GLN_374@OE1 LYS_108@HZ1 LYS_108@NZ 1 0.0010 2.9332 167.9772

ILE_280@O GLN_281@HE22 GLN_281@NE2 1 0.0010 2.9333 157.6269

GLN_142@OE1 ASN_144@HD21 ASN_144@ND2 1 0.0010 2.9333 166.5230

GLN_242@O THR_239@H THR_239@N 1 0.0010 2.9335 175.5932

ASP_167@OD1 ARG_164@HH11 ARG_164@NH1 1 0.0010 2.9347 150.9213

SER_417@OG LYS_212@HZ3 LYS_212@NZ 1 0.0010 2.9370 140.9175

GLU_418@OE1 LYS_428@HZ3 LYS_428@NZ 1 0.0010 2.9374 162.8960

LYS_272@O SER_274@H SER_274@N 1 0.0010 2.9383 148.5567

THR_103@O GLY_243@H GLY_243@N 1 0.0010 2.9391 150.2226

SER_77@OG GLY_16@H GLY_16@N 1 0.0010 2.9408 139.5464

THR_63@O ALA_65@H ALA_65@N 1 0.0010 2.9413 140.9850

ASN_231@ND2 ASN_252@HD21 ASN_252@ND2 1 0.0010 2.9431 137.4563

ASN_252@ND2 ASN_231@HD21 ASN_231@ND2 1 0.0010 2.9432 146.5265

ASN_383@O ASN_383@HD22 ASN_383@ND2 1 0.0010 2.9437 139.0611

GLU_279@O GLN_281@HE22 GLN_281@NE2 1 0.0010 2.9450 150.0536

ASP_150@OD2 VAL_153@H VAL_153@N 1 0.0010 2.9473 151.6840

ASP_264@OD2 ARG_282@HH21 ARG_282@NH2 1 0.0010 2.9482 142.5816

SER_128@OG LYS_131@H LYS_131@N 1 0.0010 2.9493 153.6326

SER_128@OG LYS_127@HZ1 LYS_127@NZ 1 0.0010 2.9503 164.7178

ASP_157@OD1 GLN_147@HE21 GLN_147@NE2 1 0.0010 2.9508 156.8565

GLN_114@O GLN_114@HE22 GLN_114@NE2 1 0.0010 2.9510 135.8290

ASP_125@OD1 SER_126@H SER_126@N 1 0.0010 2.9535 139.9565

LYS_317@O LYS_317@HZ1 LYS_317@NZ 1 0.0010 2.9537 150.7722

SER_263@OG ARG_260@HH21 ARG_260@NH2 1 0.0010 2.9547 157.5243

HIE_230@ND1 ILE_227@H ILE_227@N 1 0.0010 2.9553 158.5597

ARG_392@O TRP_358@HE1 TRP_358@NE1 1 0.0010 2.9569 140.1908

GLN_207@O ASN_208@HD22 ASN_208@ND2 1 0.0010 2.9574 144.4323

SER_177@OG LYS_179@HZ2 LYS_179@NZ 1 0.0010 2.9579 139.3310

TYR_121@OH HIE_336@HE2 HIE_336@NE2 1 0.0010 2.9579 142.2763

PRO_202@O ARG_124@HH22 ARG_124@NH2 1 0.0010 2.9598 145.7968

ASP_27@O ARG_68@HE ARG_68@NE 1 0.0010 2.9606 137.5775

ASN_419@OD1 ASN_419@H ASN_419@N 1 0.0010 2.9606 137.9456

ASP_81@N THR_80@HG1 THR_80@OG1 1 0.0010 2.9609 136.4058

THR_221@O ARG_223@HH11 ARG_223@NH1 1 0.0010 2.9610 138.0456

LEU_297@O ARG_232@HH21 ARG_232@NH2 1 0.0010 2.9617 143.4034

ASN_112@ND2 GLN_114@HE21 GLN_114@NE2 1 0.0010 2.9619 152.2177

SER_435@OG ARG_408@HH12 ARG_408@NH1 1 0.0010 2.9628 141.3352

ASP_264@OD2 ARG_282@HH12 ARG_282@NH1 1 0.0010 2.9635 158.7253

ASP_420@OD1 GLN_218@HE21 GLN_218@NE2 1 0.0010 2.9645 149.4863

THR_221@O ARG_223@HH21 ARG_223@NH2 1 0.0010 2.9655 144.2218

ASP_167@OD2 ARG_164@HH22 ARG_164@NH2 1 0.0010 2.9656 142.9569

SER_128@O ASP_125@H ASP_125@N 1 0.0010 2.9657 143.2101

TYR_154@OH SER_151@HG SER_151@OG 1 0.0010 2.9671 163.5029

ASP_162@O ARG_164@HH22 ARG_164@NH2 1 0.0010 2.9671 139.0389

GLU_15@OE2 ASN_112@HD21 ASN_112@ND2 1 0.0010 2.9675 139.5956

THR_79@OG1 THR_80@H THR_80@N 1 0.0010 2.9686 136.6721

GLN_114@OE1 GLN_14@HE22 GLN_14@NE2 1 0.0010 2.9691 144.1051

ASN_144@O ASN_144@HD22 ASN_144@ND2 1 0.0010 2.9701 138.1623

ASP_150@O TYR_154@HH TYR_154@OH 1 0.0010 2.9720 141.1488

SER_263@OG ARG_265@HE ARG_265@NE 1 0.0010 2.9720 137.0953

GLU_67@O ARG_68@HE ARG_68@NE 1 0.0010 2.9739 150.4080

SER_367@OG ARG_164@HH11 ARG_164@NH1 1 0.0010 2.9747 144.8277

SER_7@O ARG_22@HH11 ARG_22@NH1 1 0.0010 2.9753 145.7477

ASN_186@O ASN_189@HD21 ASN_189@ND2 1 0.0010 2.9760 157.5894

GLN_33@OE1 GLN_302@H GLN_302@N 1 0.0010 2.9767 154.8718

TRP_40@O ARG_309@HH12 ARG_309@NH1 1 0.0010 2.9772 135.5581

SER_141@O GLN_14@HE22 GLN_14@NE2 1 0.0010 2.9780 149.2183

GLN_424@NE2 GLN_424@H GLN_424@N 1 0.0010 2.9794 145.0899

ALA_187@O ASN_190@HD22 ASN_190@ND2 1 0.0010 2.9826 145.0314

ASP_443@O ARG_441@HH11 ARG_441@NH1 1 0.0010 2.9832 149.2859

THR_283@OG1 THR_214@HG1 THR_214@OG1 1 0.0010 2.9835 168.7767

TRP_439@O ARG_441@HH12 ARG_441@NH1 1 0.0010 2.9843 172.2580

THR_66@O ARG_68@HH12 ARG_68@NH1 1 0.0010 2.9848 137.3371

TRP_40@NE1 ASN_38@HD21 ASN_38@ND2 1 0.0010 2.9848 148.8119

GLN_142@O GLN_142@HE22 GLN_142@NE2 1 0.0010 2.9849 148.4814

GLN_379@OE1 ARG_386@H ARG_386@N 1 0.0010 2.9859 165.8766

TYR_387@OH LYS_377@HZ2 LYS_377@NZ 1 0.0010 2.9863 141.6978

ASP_425@OD1 ARG_426@HH22 ARG_426@NH2 1 0.0010 2.9864 150.5502

TYR_69@OH ARG_22@HE ARG_22@NE 1 0.0010 2.9865 149.4316

SER_26@O SER_26@HG SER_26@OG 1 0.0010 2.9873 136.0846

LEU_261@O ARG_237@HH12 ARG_237@NH1 1 0.0010 2.9877 153.2706

PRO_306@O ARG_210@HH11 ARG_210@NH1 1 0.0010 2.9887 145.6872

GLN_147@OE1 GLN_374@HE21 GLN_374@NE2 1 0.0010 2.9888 141.7007

ASN_112@OD1 GLN_114@H GLN_114@N 1 0.0010 2.9890 154.7832

GLN_374@NE2 GLN_374@H GLN_374@N 1 0.0010 2.9890 143.2382

ASN_318@ND2 ASP_315@H ASP_315@N 1 0.0010 2.9891 168.3582

LEU_297@O ARG_232@HE ARG_232@NE 1 0.0010 2.9894 139.6006

SER_26@OG ASP_27@H ASP_27@N 1 0.0010 2.9901 135.7100

GLU_245@OE1 ARG_237@HH12 ARG_237@NH1 1 0.0010 2.9910 137.4463

ASP_152@O ASN_178@H ASN_178@N 1 0.0010 2.9911 156.5144

ASN_231@O ARG_270@HH22 ARG_270@NH2 1 0.0010 2.9916 135.8824

SER_141@OG GLN_114@HE21 GLN_114@NE2 1 0.0010 2.9917 155.2950

ARG_265@O SER_267@HG SER_267@OG 1 0.0010 2.9923 169.5274

SER_296@O SER_296@HG SER_296@OG 1 0.0010 2.9924 140.8127

ASP_167@OD2 ARG_164@HH11 ARG_164@NH1 1 0.0010 2.9932 145.4969

ASP_352@OD1 ARG_309@HE ARG_309@NE 1 0.0010 2.9938 141.0588

THR_313@OG1 ASP_315@H ASP_315@N 1 0.0010 2.9939 166.7359

ASN_231@ND2 ASN_231@H ASN_231@N 1 0.0010 2.9948 136.0189

GLU_301@OE2 TYR_300@H TYR_300@N 1 0.0010 2.9956 144.1335

LYS_363@O LYS_363@HZ3 LYS_363@NZ 1 0.0010 2.9957 156.0697

THR_398@O ASP_402@H ASP_402@N 1 0.0010 2.9959 138.3082

ARG_232@NH1 GLN_251@HE21 GLN_251@NE2 1 0.0010 2.9961 153.8756

TYR_154@OH GLN_379@HE22 GLN_379@NE2 1 0.0010 2.9980 142.5778

TYR_387@OH LYS_377@HZ1 LYS_377@NZ 1 0.0010 2.9992 157.5108

GLN_281@NE2 ASN_219@HD21 ASN_219@ND2 1 0.0010 2.9997 176.3034
